# An Atom-Economical Method for the Formation of Amidopyrroles Exploiting the Self-Assembled Resorcinarene Capsule

**DOI:** 10.1021/acs.orglett.0c00529

**Published:** 2020-03-16

**Authors:** Pellegrino La Manna, Carmen Talotta, Margherita De Rosa, Annunziata Soriente, Carmine Gaeta, Placido Neri

**Affiliations:** Laboratory of Supramolecular Chemistry, Dipartimento di Chimica e Biologia “A. Zambelli”, Università di Salerno, Via Giovanni Paolo II 132, I-84084 Fisciano, Salerno, Italy

## Abstract

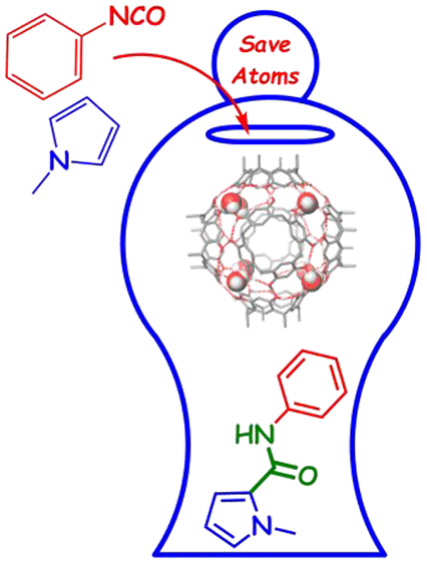

Here is reported the first example of an organocatalyzed coupling between pyrrole and isocyanates in a nanoconfined space. The hexameric resorcinarene capsule **C** is able to catalyze the direct coupling between isocyanates and pyrroles to give amidopyrroles with excellent yields and selectivities. The reaction catalyzed by **C** prevents the use of expensive and poorly atom-economical reagents. As in natural enzymes, the cavity of **C** is able to discriminate between isomeric substrates.

Amide linkages are a bedrock in living systems where they are continuously formed by complex natural catalytic systems such as ribosomes.^1^ Additionally, amide bond formation can be considered as one of the most exploited reactions in the synthesis of drug candidates,^2^ host systems for anion recognition,^3^ and materials of industrial relevance.^1^ The amide bond is also found in amidopyrroles, which play important roles in medicinal chemistry, where they act as anticancer agents. Thus, distamycin A and its derivatives act as inhibitors of DNA ligases, which are considered as druggable targets in cancer therapy.^4^ Amidopyrroles such as *N*-benzyl- and *N*-propargyl-1*H*-pyrrole-2-carboxamides show MAO inhibition activity.^5^

Traditionally, amide bond formation is obtained by acylation of amines with carboxylic acids activated by means of coupling reagents (e.g., DCC).^1,2^ This strategy uses expensive reagents that show poor atom economy and for these reasons is considered nonsustainable.^6^ On the other hand, biocatalyzed amide-bond-forming reactions are considered as highly sustainable.^6^ In fact, biocatalysts work under mild conditions with excellent stereo- and regioselectivity, avoiding poorly atom-economical reagents. In the last decades, scientists have invested considerable efforts to reduce the gap between artificial catalytic systems and their natural counterparts.^7^

In the past few years, several research groups have focused their attention on the self-assembled resorcinarene capsule **C** (Figure 1), which shows an internal cavity reminiscent of natural enzyme pockets.^8,9^**C** is formed by six resorcinarenes **1** and eight water molecules to give a self-assembled structure sealed by 60 H-bonds with the water molecules occupying the corners (Figure 1).^9^ This self-assembled capsule shows intriguing features that make it particularly adapt for enzyme mimicry:^8^ (a) it presents a π-electron-rich cavity of 1375 Å^3^ that can act as an enzyme pocket; (b) the inner cavity is able to recognize neutral and cationic species and stabilize transition states thanks to secondary interactions; (c) it behaves as a mild Brønsted acid with a p*K*_a_ value of about 5.5–6.0; (d) its inner cavity can establish H-bonding interactions with hosted molecules thanks to the presence of bridging water molecules with H-bond-donating free valence (Figure 1).^8^ These catalytic features have been exploited with amazing results in the literature.^8,10,11^

**Figure 1:**
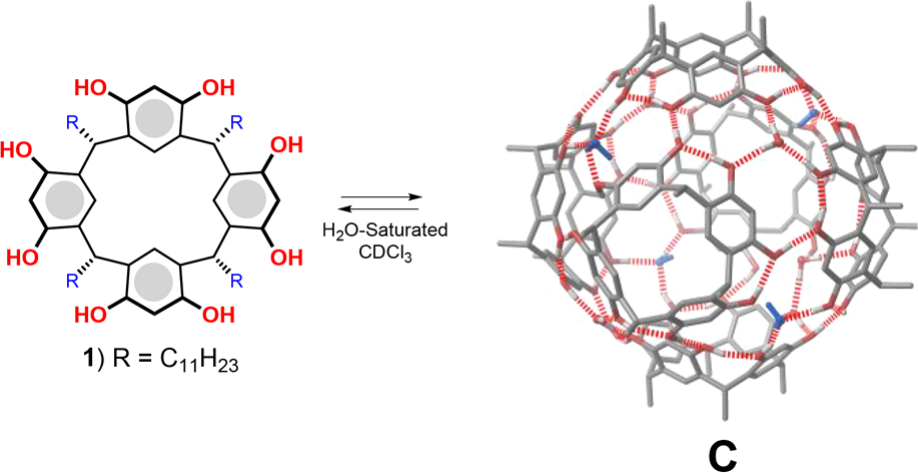
*C*-undecylresorcin[4]arene **1** self-assembles to form the hexameric resorcinarene capsule **C** in the presence of H_2_O-saturated CDCl_3_. In blue are shown the bridging water molecules with H-bond-donating free valence toward the center of the cavity of **C**.

With regard to the synthesis of amidopyrrole derivatives, an interesting work reported by Neumann in 1990^12^ showed that isocyanates can be considered as a useful vector for amide linkage. The authors reported the reaction of trialkylstannyl-substituted aromatic and heterocyclic compounds with aryl isocyanates in the presence of aluminum trichloride to give *N*-aryl-substituted amides.^12^ In a similar vein, in 1988 Katritzky^13^ reported the formation of amidopyrroles by reaction of C-lithiated pyrroles with isocyanates. More recently, the formation of amidopyrroles by rhenium-catalyzed insertion of isocyanates into C–H bonds of heteroaromatic compounds was reported.^14^ However, all of these strategies showed poor atom economy because of the reagents necessary for the activation of the pyrrole nucleophiles. To the best of our knowledge, no examples have been reported in the literature regarding the organocatalyzed formation of amidopyrroles from pyrroles and isocyanates. Prompted by these considerations, we decided to explore the organocatalyzed, atom-economical formation of amidopyrroles by confinement of pyrrole and isocyanate inside capsule **C**.^15^ As a preliminary step, we started our investigation by testing the reaction between *N*-methylpyrrole (**2a**) and phenyl isocyanate (**3a**) (Table 1). Their reaction in the presence of 26 mol % capsule **C** at 50 °C for 40 h in water-saturated CDCl_3_ afforded **4aa** in 99% yield (Table 1, entry 2).

**Table 1:**
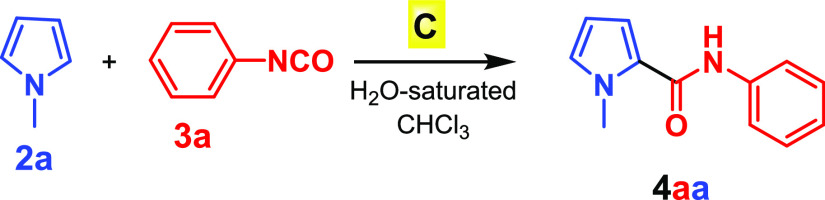
Optimization of the Reaction Conditions for the Coupling of **2a** with **3a**^a^

entry	capsule (mol %)^b^	**2a**/**3a**	*T* (°C)	*t* (h)	yield (%)^c^
1	–d	4	50	40	–
**2**	**26**	**4**	**50**	**40**	**99**^e^^,^^f^
3	15	4	50	40	5
4	40	4	50	40	85
5	26	2	50	40	38
6	26	1	50	40	11
7	26	4	50	40	–g
8	26	4	50	40	–h

a Reaction conditions: **2a** (0.59 M), **3a** (0.15 M), H_2_O-saturated CDCl_3_ (1.1 mL).

b Calculated with respect to **3a**.

c Yield of the product isolated by column chromatography.

d Only starting materials were recovered.

e The same result was obtained using H_2_O-saturated CHCl_3_.

f Experiments on the reusability of **C** under these optimized conditions were performed, giving a positive indication of the reusability of the capsule **C**. Indeed, the activity was maintained after three cycles: run 2, 90%; run 3, 75% .

g The reaction was performed in the presence of tetraethylammonium tetrafluoroborate (0.76 M).

h The reaction was carried out in the presence of DMSO (0.76 M).

The presence of **C** is mandatory in order to ensure a successful outcome of the reaction. In fact, the reaction performed under the same conditions (Table 1, entry 2) but in the absence of capsule **C** did not show any conversion of substrates **2a** and **3a** to **4aa**, even after a prolonged reaction time (Table 1, entry 1).

With these results in hand, a series of experiments were performed in order to investigate the effects of the reaction conditions on the efficiency of the reaction (Table 1, entries 3–6). When the reaction between **2a** and **3a** was performed in the presence of a lower amount of **C** (15 mol %), the product **4aa** was isolated in a very low yield (Table 1, entry 3). With 40 mol % **C**, amidopyrrole **4aa** was isolated in a slightly lower yield (85%; Table 1, entry 4) with respect to the run with 26 mol % **C**. Finally, a lowering of the reaction yield was observed when the **2a**/**3a** ratio was lowered to 2 or 1 (Table 1, entries 5 and 6). Following a standard protocol previously reported by us and others,^8,10,11^ the role of capsule **C** in the catalysis of the reaction in Table 1 was studied. When the reaction between **2a** and **3a** in the presence of **C** was performed under the same conditions but in the presence of tetraethylammonium tetrafluoroborate (**5**) (Figure 2b), a known competitive guest with high affinity for the inner cavity of **C**,^8^ then no hint of product **4aa** was detected in the reaction mixture (Table 1, entry 7). Analogously, upon addition of dimethyl sulfoxide, which can dissociate the capsule by breaking its H-bonding network, no evidence of product **4aa** was detected (Table 1, entry 8). These results confirmed that the reaction takes place inside the capsule **C** through the formation of the heterocomplex **2a+3a@C** in Figure 2.

**Figure 2:**
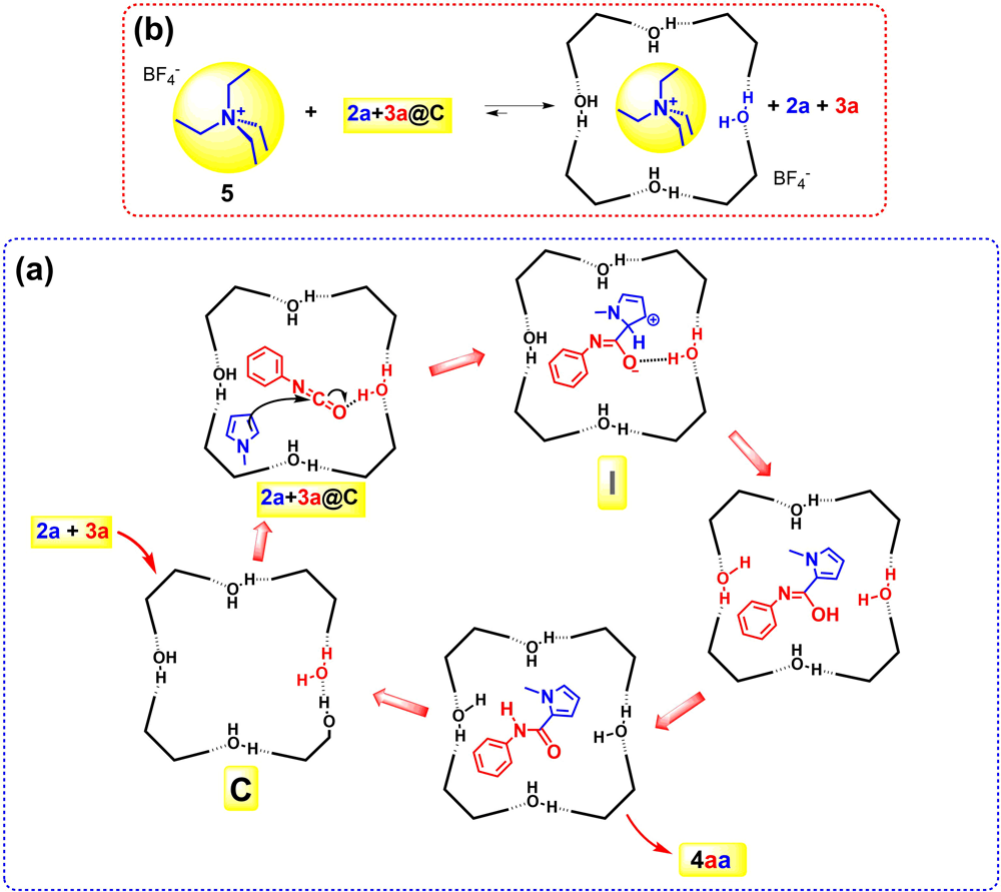
(a) Proposed mechanism for the formation of amidopyrrole **4aa** inside **C**. (b) Proposed mechanism for the competitive inhibition of **C** by tetraethylammonium tetrafluoroborate (**5**).

At this point, the scope of the reaction between pyrroles and isocyanates was studied under the optimized conditions (Table 1, entry 2) using pyrrole derivatives bearing different N substituents (Scheme 1). In accord with the nucleophilicity scale of typical π systems reported by Mayr and co-workers,^16^ the less nucleophilic unsubstituted pyrrole (**2b**) showed a lower reactivity than *N*-methylpyrrole **2a** toward isocyanate **3a**, thus requiring 72 h, rather than 40 h, to give product **4ab** in 99% yield (Scheme 1). Interestingly, other N-substituted pyrroles **2c**–**f** (Scheme 1) gave the corresponding amidopyrroles by reaction with **3a** in high yields but after longer reaction times than with **2a**. The reaction between isocyanate **3a** and *N*-phenylpyrrole (**2f**) gave the expected amidopyrrole **4af** in 74% yield after 96 h (Scheme 1), indicating in this way its lower reactivity with respect to *N*-alkyl-substituted pyrroles **2a**, **2c**, and **2d** bearing smaller N substituents. Interestingly, when the *N*-phenyl group of **2** was *para*-substituted as in **2g**–**j**, no hint of the corresponding products was detected in the reaction mixtures with **3a** (Scheme 1). When pyrrole **2k** bearing a *meta*-OMe-substitued *N*-phenyl group was used, the reaction with **3a** gave amidopyrrole **4ak** in 22% yield after 96 h. The yield increased to 99% when isomeric **2l** with the *ortho*-OMe-substituted *N*-phenyl group was used. In a similar way, when *N*-benzyl-substituted pyrroles **2m**–**r** were investigated in the reaction with **3a**, the *ortho*-substituted pyrroles **2m**, **2o**, and **2q** showed higher reactivity than the *meta*-substituted isomers **2n**, **2p**, and **2r** (Scheme 1). All of these results clearly indicated that the formation of the catalytically active heterocomplexes **2+3a@C** is favored with pyrroles **2m**–**r** bearing *ortho*- or *meta*-substituted phenyl groups with respect to the longer *para*-substituted isomers **2g**–**j**, which are more sterically demanding.^17^ Overall, these results clearly indicate that, like a natural enzyme, capsule **C** is able to discriminate the pair of substrates pyrrole/phenyl isocyanate by inclusion inside its cavity. In particular, concerning the *N*-phenyl- or *N*-benzylpyrroles, capsule **C** shows the affinity scale *ortho* > *meta* > *para* with regard to substitution.^17^ On the basis of these observations (Scheme 1), we propose the mechanism reported in Figure 2 for the formation of amidopyrrole derivatives **4** in the nanoconfined space of **C**. Initially, the heterocomplex **2+3a@C** is formed, with isocyanate **3a** H-bonded to a bridging water molecule.^18^ At this point, α-attack of the pyrrole to the H-bonded activated isocyanate **3a** occurs inside the capsule, leading to intermediate **I**, which is stabilized through H-bonding interactions. Then the rearomatization of **I** and the successive prototropic equilibrium give the final amidopyrrole product **4aa**.

**Scheme 1:**
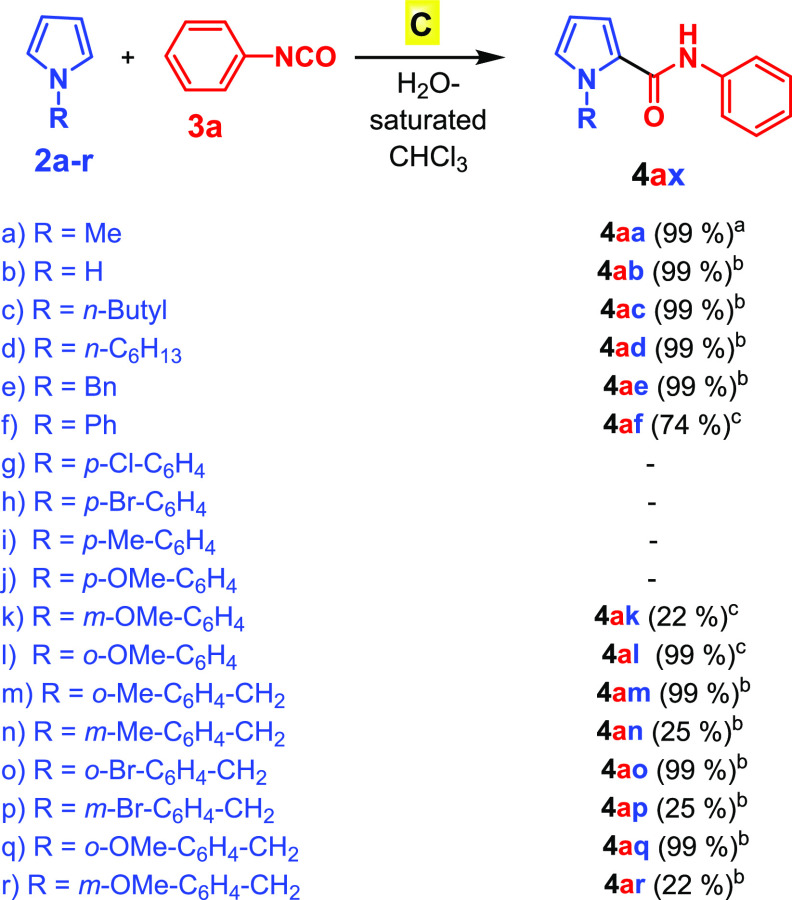
Synthesis of Amidopyrroles **4aa**–**ar** Conditions reported in Table 1, entry 2, 40 h. 72 h. 96 h. Reaction conditions: **2a**–**r** (0.59 M), **3a** (0.15 M), **C** (0.039 M), H_2_O-saturated CHCl_3_ (1.1 mL). Yields of the products isolated by column chromatography are shown. In the numbering scheme **4ax**, the blue and red letters refer to the pyrrole and isocyanate starting compounds **2a**–**r** and **3a**, respectively.

Spectroscopic evidence for the encapsulation of pyrrole **2a** inside **C** was previously reported by our group.^10^ In addition, the encapsulation of **3a** was ascertained by 2D EXSY and DOSY NMR experiments following a standard protocol previously reported by us and others.^10^ In detail, the 2D EXSY spectrum of the mixture of **3a** and **C** in water-saturated CDCl_3_ evidenced the presence of an exchange cross-peak at 3.81/7.11 ppm between the aromatic signals of isocyanate **3a** inside and outside capsule **C**, respectively (Figures S5 and S6). Furthermore, the DOSY NMR experiment (Figure S7) indicated that the aromatic protons of the encapsulated **3a**, at 3.81 ppm, showed the same diffusion coefficient as the hexameric capsule **C**. Analogously, the formation of the catalytically active **2a+3a@C** heterocomplex was ascertained by 2D EXSY and DOSY NMR experiments. In detail, an exchange cross-peak was found at 5.27/6.19 ppm (Figure S154) attributable to aromatic protons of **2a** inside and outside the capsule. Analogously, exchange cross-peaks were observed at 3.09/7.12 and 3.31/7.35 ppm that were attributable to aromatic signals of **3a** inside/outside the capsule. The generality of the procedure here described was further proved by experiments summarized in Figure 3. In fact, *ortho*- and *para*-substituted aromatic isocyanates **3b**–**i** were also able to react with *N*-methylpyrrole **2a**, leading to amidopyrroles **4(b**–**i)a** in high yields (Figure 3a). Notably, the large 1-naphthyl isocyanate (**3i**) had also no difficulty in reacting with **2a** to give product **4ia** (Figure 3a). Moreover, benzyl isocyanate (**3j**) afforded amide **4ja** in good yield. Analogously, unsubstituted pyrrole **2b** and *N*-benzylpyrrole (**2e**) gave the corresponding amidopyrroles in Figure 3b,c upon reaction with the appropriate isocyanates in the presence of capsule **C**.

**Figure 3:**
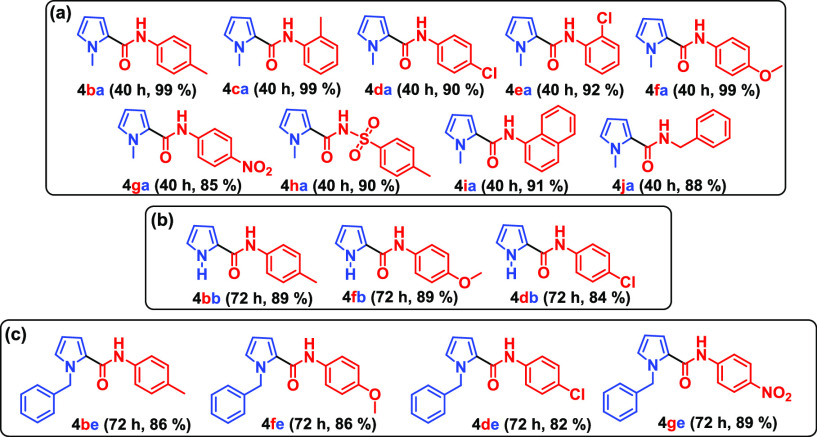
Synthesis of amidopyrroles starting from appropriate isocyanates **3b**–**j**. Reaction conditions: **2a,b,e** (0.59 M), **3b**–**j** (0.15 M), **C** (0,039 M), water-saturated CHCl_3_ (1.1 mL). The yield of the product isolated by column chromatography is given in parentheses. (a) Starting with *N*-methylpyrrole (**2a**) and appropriate isocyanates **3b**–**j**. (b) Starting with pyrrole (**2b**) and isocyanates **3b**, **3f**, and **3d**. (c) Starting with *N*-benzylpyrrole (**2e**) and isocyanates **3b**, **3f**, **3d**, and **3g**. In the numbering scheme **4xx**, the blue and red letters refer to the isocyanate and pyrrole starting compounds **2** and **3**, respectively.

These results indicated that the reaction is less affected by the changes in isocyanates **3** with respect to the substituent effects observed for pyrroles **2**. In analogy with previous results,^10,11^ this difference can be explained in the following way. As reported in Figure 2a, an isocyanate substrate **3** is involved in a strong H-bonding interaction with a bridging water molecule of the capsule, whereas pyrrole substrate **2** interacts only through weaker van der Waals-like interactions (CH−π and π–π). Therefore, the isocyanate substrate **3**, being more tightly bound, first occupies all of the needed space in the large capsule volume with no size discrimination. At this point, the more loosely bound pyrrole substrate **2** can occupy only the free space left over by **3**, which is quite smaller and hence exerts the observed size discrimination. Interestingly, isocyanates did not undergo hydrolysis under the experimental reaction conditions. This was confirmed with blank experiments performed with isocyanate substrate **3a** alone in water-saturated chloroform in the presence or absence of capsule **C** (see the Supporting Information), where hydrolysis product(s) could not be detected. This behavior can be mainly ascribed to the known low reactivity of aryl isocyanates.

In conclusion, we have here reported an example of organocatalyzed amide bond formation that exploits the nanoconfined space inside the hexameric resorcinarene capsule. Thus, amidopyrroles were obtained with excellent yields and selectivities by the direct coupling between isocyanates and pyrroles. Like an enzyme pocket, the inner cavity of the capsule is able to discriminate isomeric substrates. The strategy here described is highly sustainable and prevents the use of expensive and poorly atom-economical coupling reagents.

## Supporting Information Available

Detailed synthetic procedures, characterization of supramolecular complexes with the capsule **C**, and 1D and 2D NMR spectra of new compounds (PDF)
