# Supplementary material for: An Atom-Economical Method for the Formation of Amidopyrroles Exploiting the Self-Assembled Resorcinarene Capsule
Source: Org Lett. 2020 Mar 16;22(7):2590–4. doi: 10.1021/acs.orglett.0c00529 (PMC7997627; doi:10.1021/acs.orglett.0c00529)
Supplement: Supplementary file 1 — ol0c00529_si_001.pdf [file ol0c00529_si_001.pdf]

Supporting Information

An Atom-Economical Method for the Formation of Amidopyrroles Exploiting the Self-Assembled Resorcinarene Capsule

Pellegrino La Manna, Carmen Talotta,* Margherita De Rosa, Annunziata Soriente, Carmine Gaeta and Placido Neri*

Laboratory of Supramolecular Chemistry, Dipartimento di Chimica e Biologia, “A. Zambelli”, Università di Salerno, Via Giovanni Paolo II 132, Fisciano (SA), I-84084, Italy.

Table of Content

I. General Informations…………………………………………………………………..S3

II. General Procedure for the Amide Bond Formation between Pyrroles and Isocyanates in absence of **C (GP1)**…………...…………………………………………………….…S4

III. General Procedure for the Amide Bond Formation between Pyrroles and Isocyanates in presence of **C (GP2)**……………………………………………………...………S4

IV. Inhibition Experiments with Tetraethylammonium Tetrafluoroborate **5……………..….**S4

V. Inhibition Experiments with DMSO……………………………………..………....S7

VI. Encapsulation Experiments: NOESY and DOSY Spectra of the Mixture Phenyl isocyanate **3a** and Capsule **C**………………………….………………….……….**.**S8

VII. Experiments on the Reusability of the Capsule **C**……………………..….…..……….S11

VIII. Characterization Data of Pyrrole Derivatives **2m-n**………….……………...……….S13

IX. Characterization Data of Amides **4ac-ge**..S15

X. ^{1}H and ^{13}C NMR Spectra and 2D NMR Spectra of Pyrrole Derivatives **2m-n……**……………………..…………………………………………………..…..S27

XI. ^{1}H and ^{13}C NMR Spectra and 2D NMR Spectra of Amides **4ac-ge**…….…….…..……S37

XII. ^{1}H and ^{13}C NMR Spectra of derivatives **4ha** and **4fb**…………………………………S148

XIII. Hydrolysis test of the phenyl isocyanate **3a**……………………….…………………S152

XIV. 1 mmol Scale synthesis of **4aa** in presence of the hexameric resorcinarene capsule **C** starting by pyrrole **2a** and isocyanate **3a**……………………………………………………………..S154

XV. Encapsulation Experiments: NOESY and DOSY Spectra of the Mixture of Pyrrole **2a**, Phenyl isocyanate **3a** and Capsule **C**…………………………………..S155

XVI. References………………………….……………………………………………….…S157

I. General Informations

All chemicals were reagent grade and were used without further purification. Solvents, isocyanates **3a-j**, pyrroles **2a-b**, 2-Methylbenzyl chloride and 3-Methylbenzyl chloride were purchased from Sigma-Aldrich (Merck). Heat source of the reactions was an oil bath and reaction temperatures were measured externally; reactions were monitored by ^{1}H NMR and by TLC on Merck silica gel plates (0.25 mm) and visualized by UV light and spraying with H_2SO_4-$Ce(SO_4)_2$ or phosphomolybdic acid. Chromatography was performed on Merck silica gel (60, 40-63 µm). NMR spectra were recorded on Bruker Avance-600 spectrometer [600.13 MHz (^{1}H) and 150.03 MHz (^{13}C)] and Bruker Avance-400 spectrometer [400 (^{1}H) and 100.57 MHz (^{13}C)]. Chemical shifts were reported in ppm, relative to the residual solvent peak ($CHCl_3$: δ 7.26, $CDCl_3$: δ 77.23). Multiplicity was reported as s (singlet), d (doublet), t (triplet), dd (double of doublets), quint (quintet) and m (multiplet). Coupling contants were reported in hertz (Hz). DOSY experiments were performed on a Bruker Avance-600 spectrometer equipped with 5 mm PABBO BB|19F-1H\D Z-GRD Z114607/0109. The standard Bruker pulse program, ledbpgp2s, employing a double stimulated echo sequence and LED, bipolar gradient pulses for diffusion, and two spoil gradients were utilized. Diffusion times were 150 ms, eddy current delay was 5 ms, gradient recovery delays were 0.2 ms, and gradient pulse was 1400 ms. Individual rows of the quasi-2D diffusion databases were phased and baseline corrected. NOESY experiments were performed on Bruker Avance 600 spectrometers with a D8 value range from 100 to 900 ms. All final compounds purity was determined by elemental analysis on a Flash EA 1112 Series with Thermal Conductivity Detector, for C, H and N. Resorcinarene **1**[1] and derivatives **5**[2], **2c**[3], **2d**[4], **2e**[5], **2f**[6], **2g-j, 2l**[7], **2k**[8], **2o**[9], **2p**[10], **2q**[11] and **2r**[12] were synthesized according to literature procedures. Pyrrole derivatives **2m** and **2n** were synthesized adapting procedure reported in ref. **5** and fully characterized. Amides **4aa**[13], **4ba**[14], **4ha**[15] and **4fb**[16] are already known in literature.

II. General Procedure for the Amide Bond Formation between Pyrroles and Isocyanates in Absence of C (GP1).

In a typical experiment, pyrrole derivative **2a-r** (650.4 µmol, 4.0 equiv) was added into a 4 mL vial containing 1.1 mL of H_2O-saturated $CHCl_3$, and the solution was stirred for 2 minutes at 50 °C. Then, the appropriate isocyanate **3a-j** (162.6 µmol, 1.0 eq.) was added and the solution was vigorously stirred (1400 rpm) at 50 °C for the appropriate time. The progress of the reaction was monitored by ^1H NMR analysis by periodically sampling from the reaction system at different times.

III. General Procedure for the Amide Bond Formation between Pyrroles and Isocyanates in presence of C (GP2).

Resorcinarene **1** (282 mg, 255 µmol, 1.56 equiv) was weighed in a 4 mL vial. Then, 1.1 mL of H_2O-saturated $CHCl_3$ was added and the mixture was stirred at 50 °C for 10 min. To this clear, slight yellow solution, the appropriate derivative **2a-r** (650 µmol, 4.00 equiv) was added and the solution was stirred at 30 °C for 5 min. Then, the derivative **3a-j** (163 µmol, 1.00 equiv) was added and the reaction mixture was vigorously stirred (1400 rpm) at 50 °C for the appropriate time. The reaction was monitored by ^1H NMR analysis taking aliquots of the reaction mixture (50 µL) at various time intervals and diluting with H_2O-saturated $CDCl_3$ (0.35 mL). The reaction was stopped pouring the solution into a 50 mL Eppendorf conical tube and diluting with a 0.13 % (v/v) solution of DMSO in *n*-hexane (35 mL). The tube was placed in a freezer at −20 °C for 3 h and successively centrifugated at 1250 rpm for 8 minutes. The diluted reaction mixture was subjected three times to this process of centrifugation/dilution with *n*-hexane. Finally, the clear solution was filtered and concentrated under reduced pressure. The brown oily residue thus obtained was purified by chromatography on silica gel to afford the desired compound.

IV. Inhibition Experiments with Tetraethylammonium Tetrafluoroborate 5.

With the aim to demonstrate that the reaction between **2a** and **3a** occurs inside the resorcinarene capsule **C**, the reaction was performed in the presence of a tetralkylammonium salt since it is known in literature that tetraethylammonium tetrafluoroborate **5** is able to occupy the inner cavity of **C**, acting as an inhibitor[17]. Resorcinarene **1** (282 mg, 255 µmol, 1.56 equiv) was weighed in a 4 mL vial. Then, 1.1 mL of H_2O-saturated $CHCl_3$ was added and the mixture was stirred at 50 °C for 10 min. To this clear, slight yellow solution, the derivative **2a** (650 µmol, 4.00 equiv) was added and the

solution was stirred for 10 min. Then, the derivative **3a** (163 μmol, 1.00 eq.) was added, followed by **5** (920 mg, 424 μmol, 10 equivalents respect to **C**). As reported in **Figure S2**, no trace of product **4aa** (**Figure S1**) was detected in the reaction mixture. Only signals attributable to reactants **2a** and **3a** are visible. In absence of the guest **5**, products signals are clearly visible in the ^{1}H NMR spectrum of the crude reaction mixture (**Figure S3**). Noteworthy, spectrum of the crude mixture of the reaction between **2a** and **3a** in absence of the capsule **C** shows no trace of products (**Figure S3**), reaffirming the pivotal role played by the aggregate **C**.

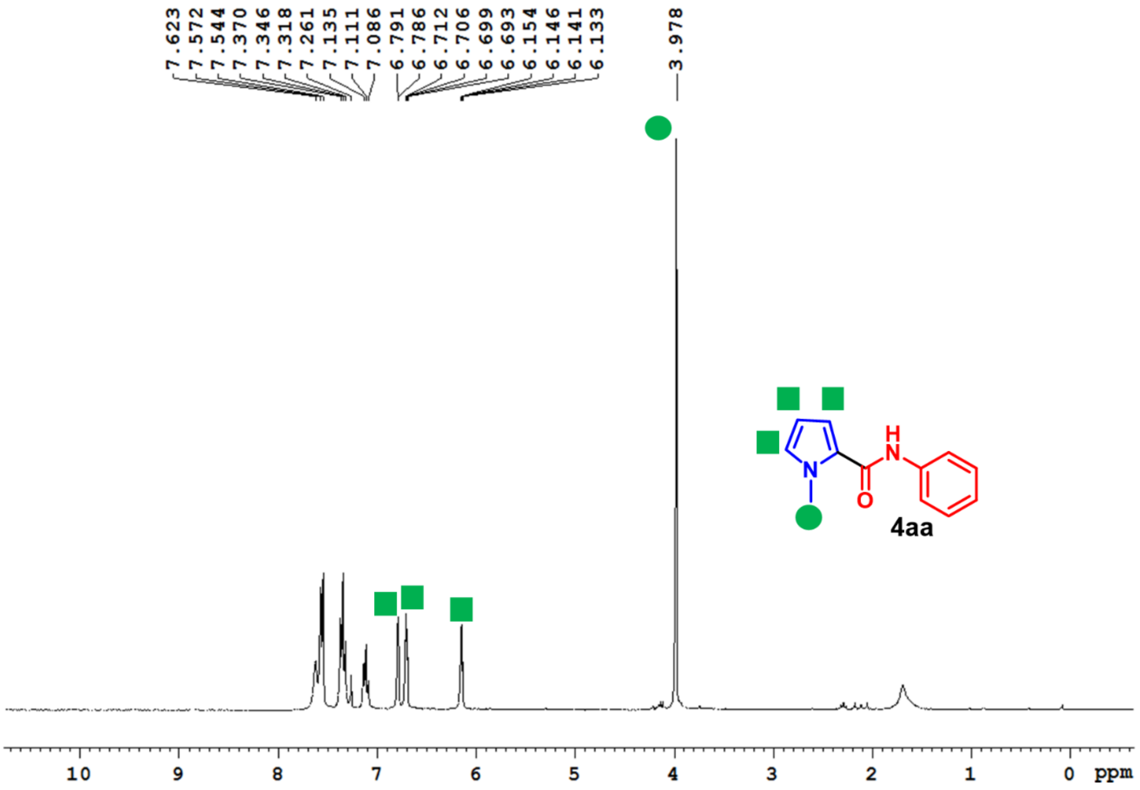

Figure S1. ^{1}H NMR (400 MHz, $CDCl_3$, 298 K) spectrum of the derivative **4aa**.

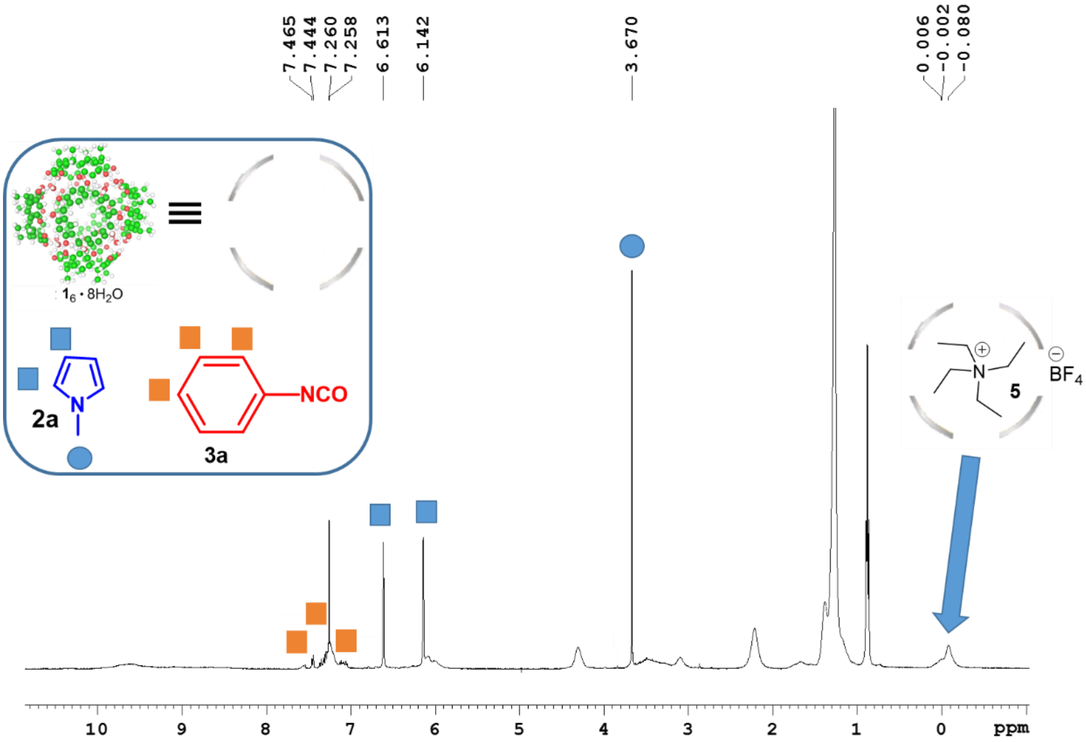

Figure S2.^{1}H NMR (400 MHz, $CDCl_3$, 298 K) spectrum of the crude mixture of the reaction between **2a** and **3a** in presence of the capsule **C** and ammonium guest **5** performed in H_2O-saturated $CHCl_3$. No trace of product is visible and signals attributable to the encapsulated ammonium salt **5** are marked with a blue arrow. Morover, signals attributable to **2a** and **3a** are indicated.

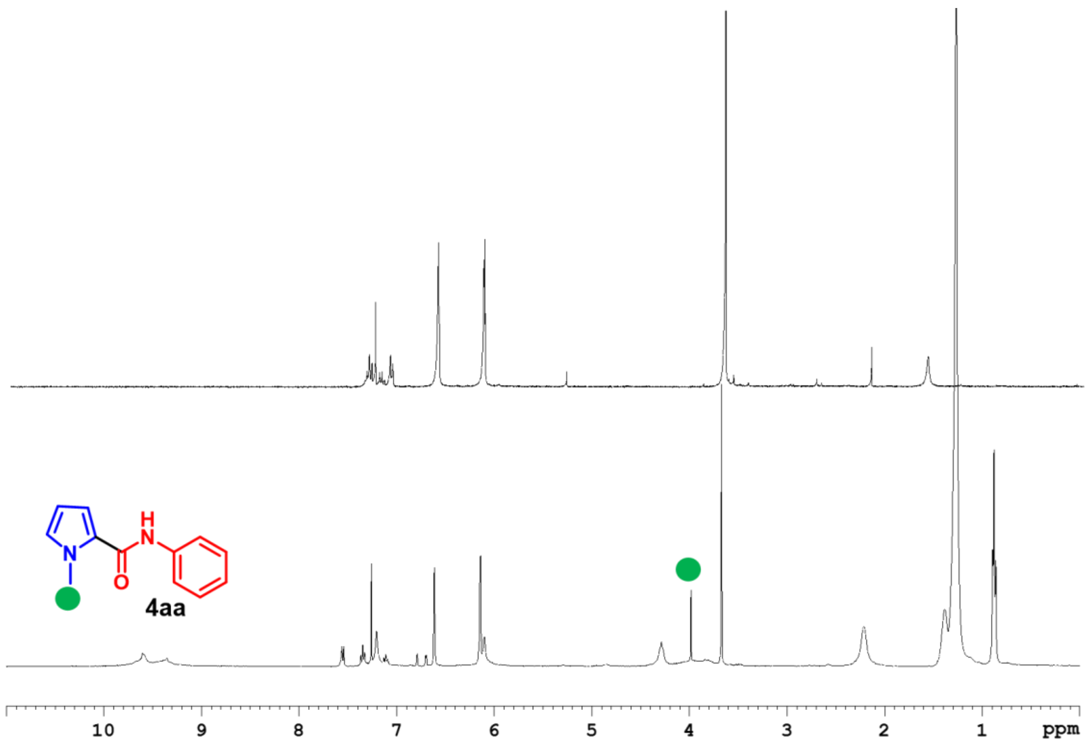

Figure S3. ^{1}H NMR (400 MHz, $CDCl_3$, 298 K) spectra of the crude reaction mixture of the *N*-methylpyrrole (**2a**) with phenyl isocyanate (**3a**) promoted by the capsule **C** (bottom) and without capsule **C** (top). In the latter case, no trace of product **4aa** is present.

V. Inhibition Experiments with DMSO.

To a solution of **2a** (650 µmol, 4.00 equiv), **3a** (163 µmol, 1.00 equiv) and **1** (282 mg, 254.7 µmol, 1.56 eq.) in 1.1 mL of H_2O-saturated $CHCl_3$, DMSO (1.12 mmol, 26.6 eq.) was added and the reaction mixture was stirred at 50°C for the appropriate time. No hint of product **4aa** was detected in the ^{1}H NMR spectrum of the reaction mixture in **Figure S4**. In fact, as it is known, H-bonds competitor solvent such as DMSO is able of destroying the resorcinarene aggregate **C** (**ref. 8b** in the manuscript).

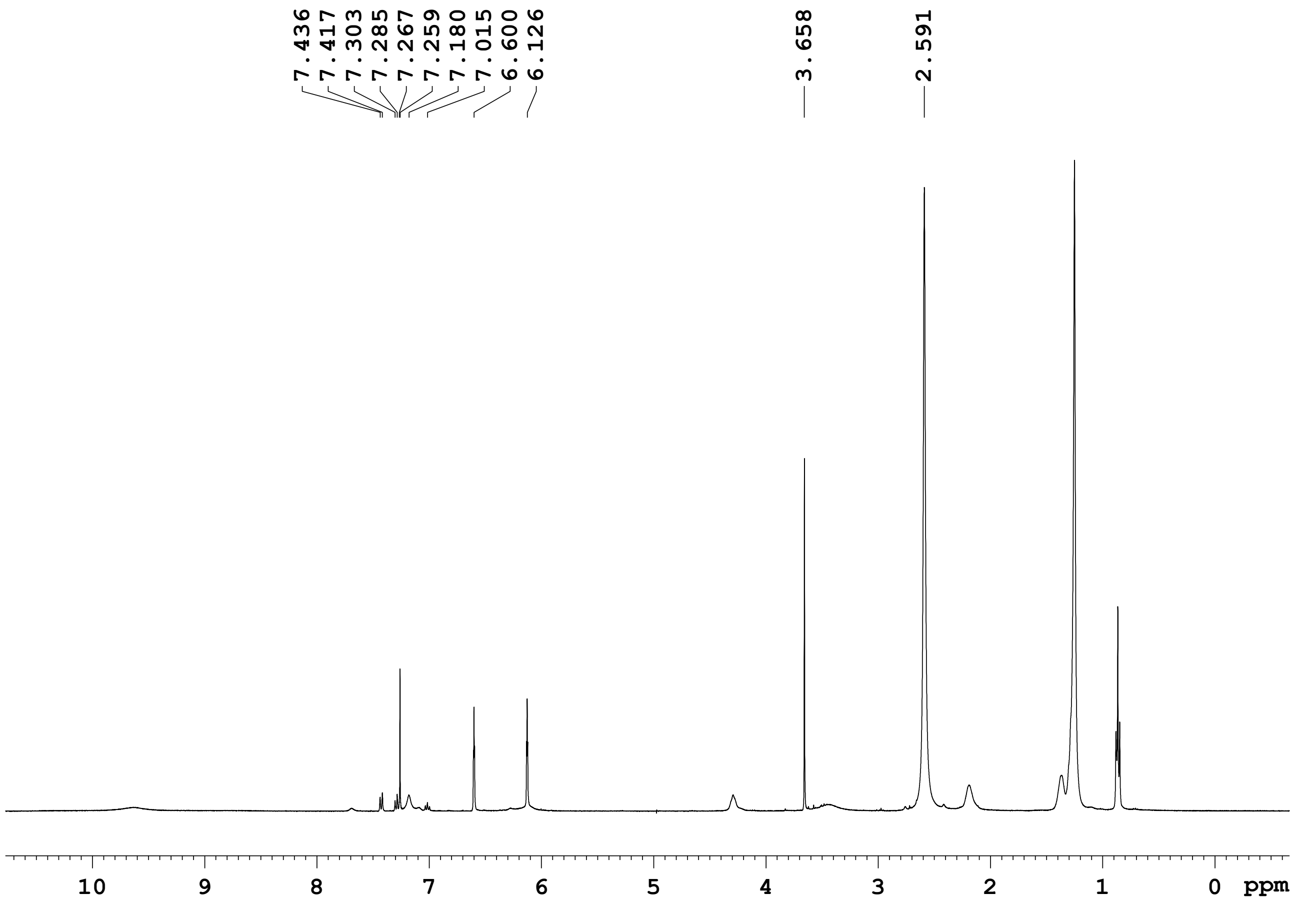

Figure S4. ^{1}H NMR (400 MHz, $CDCl_3$, 298 K) spectrum of the crude reaction mixture between **2a** and **3a** in presence of the capsule **C** and DMSO performed in H_2O-saturated $CHCl_3$.

VI. NOESY and DOSY Spectra of Encapsulation Experiments between Phenyl isocyanate 3a and capsule C.

Samples were prepared according to the following procedure: resorcinarene **1** (142 mg, 0.13 mmol) was weighed in a 4 mL vial containing 0.7 mL of H_2O-saturated $CDCl_3$ and this solution (31 mM respect to the hexameric capsule) was used for the experiments. 9.70 mg of phenyl isocyanate **3a** (116 mM) were added to the solution and this latter was stirred for 5 minutes.

In accordance with a standard protocol previously reported, evidences for the encapsulation of the reagents inside the capsule **C** were provided by 1D and 2D-NMR studies.

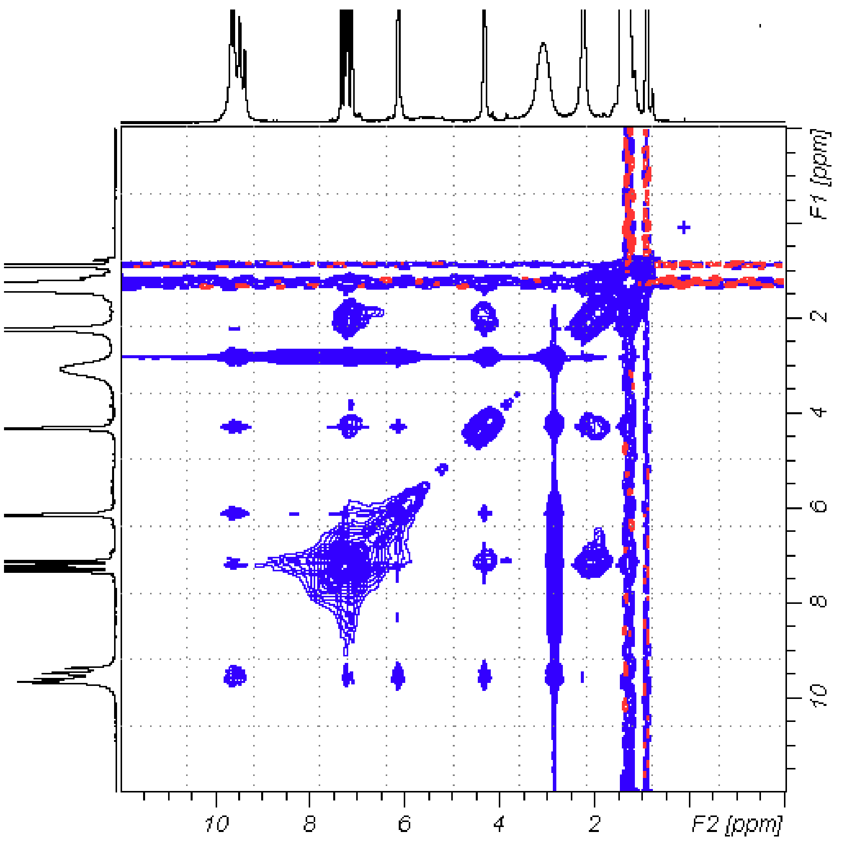

Figure S5. NOESY 2D NMR spectrum (600 MHz, $CDCl_3$, 298 K, d8 = 700 ms), of the mixture of capsule **C** and phenyl isocyanate **3a**.

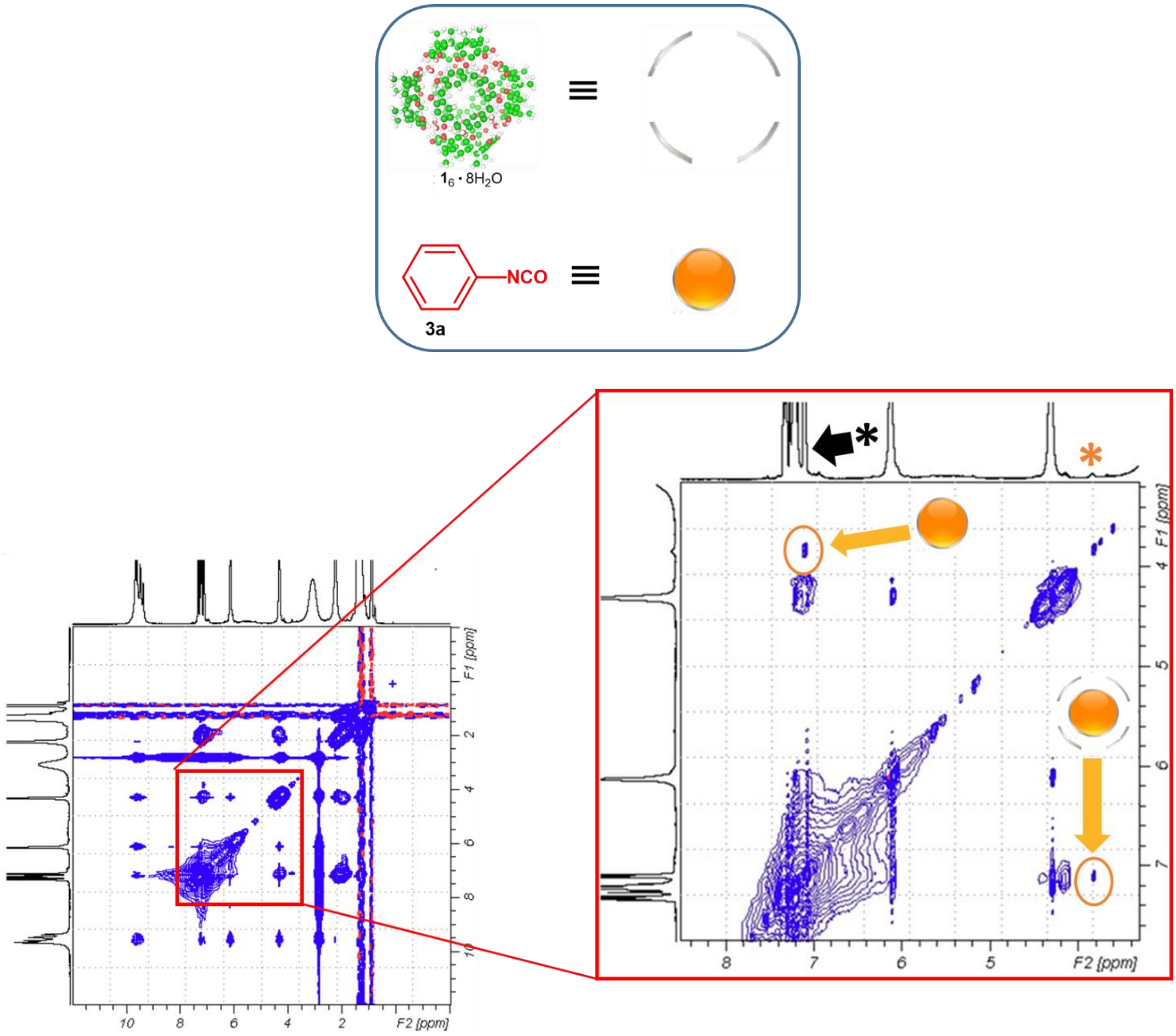

Figure S6. Relevant region of 2D NOESY NMR spectrum (600 MHz, $CDCl_3$, 298 K, d8 = 700 ms), of capsule **C** and phenyl isocyanate **3a**. Exchange cross-peak between CH aromatic proton of **3a** *outside* the capsule (indicated with a black asterisk) and *inside* the capsule (indicated with an orange asterisk) is shown.

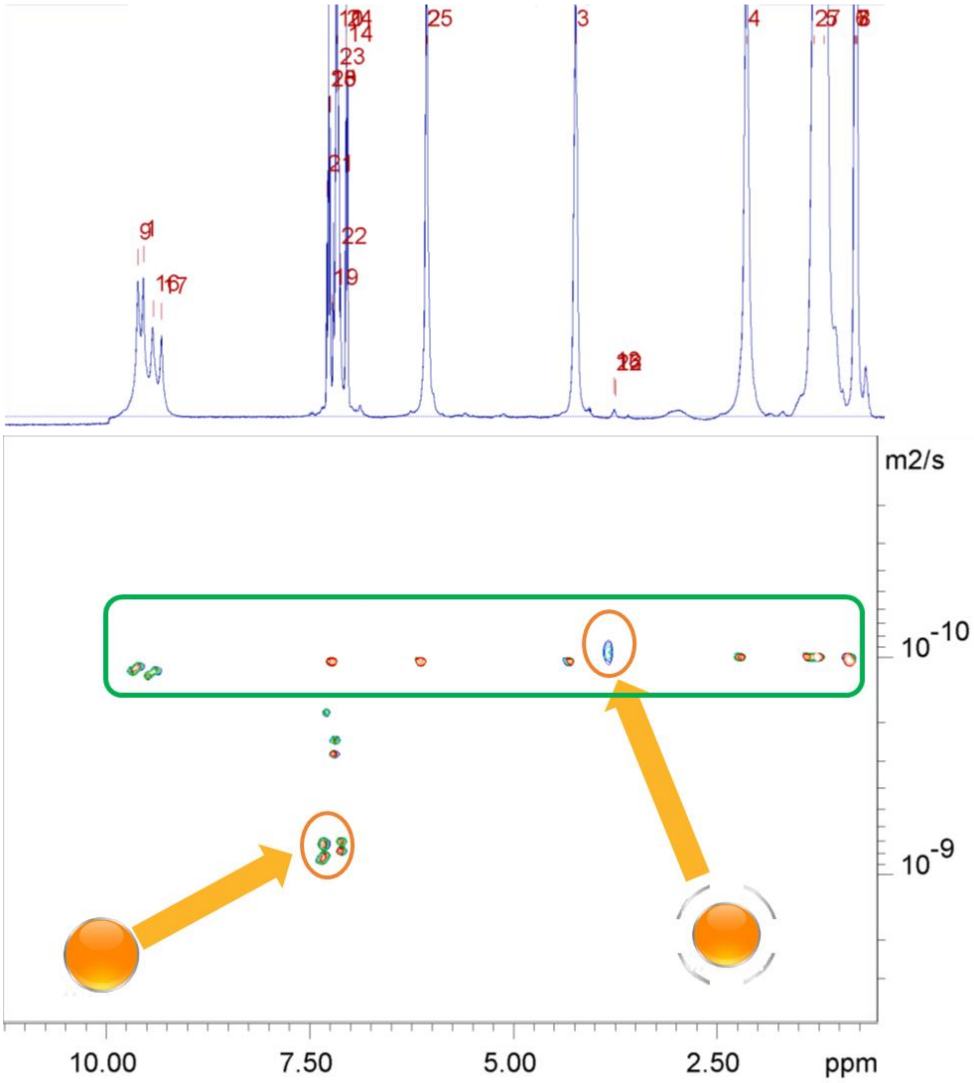

Figure S7. DOSY NMR spectrum (600 MHz, $CDCl_3$, 298 K) of the phenyl isocyanate **3a** and capsule **C**. As indicated in Figure, the signals pattern associated to the phenyl isocyanate **3a** hosted inside the resorcinarene hexameric capsule **C** show the same diffusion coefficient of the hexameric capsule.

VII. Experiments on the reusability of the capsule C

In order to investigate the reusability of **C**, the reaction between **2a** and **3a** in presence of the capsule was performed. After the workup procedure described in the **GP2**, resorcinarene precipitate was washed several times (4 x 30 mL) with deionized H_2O and centrifugated. Then, the beige solid obtained was dried in vacuo for 48 h. After this time, it was weighed in a 4 mL vial and the **GP2** was repeated. This procedure was iterated four times and results are summarized in **Table S1**. No product was obtained at the fourth cycle.

Cycle	Catalyst amount recovered (mg)	Yield of **4aa** (%)[a]
**1**	281.6 mg	99
**2**	271	90
**3**	250	75
**4**	240	0

Table S1. Results for the reusability experiments of the capsule **C** in the reaction between **2a** (0.59 M) and **3a** (0.15 M) in H_2O-saturated $CHCl_3$ (1.1 mL). [a]Isolated yields after silica gel column chromatography.

VIII. Characterization data of pyrrole derivatives 2m and 2n.

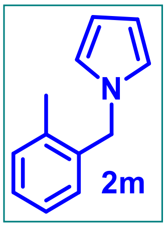

Derivative 2m. Obtained as a brownish oil adapting a procedure reported in ref. **5**. To a 100 mL round bottomed flask, finely powdered KOH (59 mmol, 4.0 equiv) and DMSO (20 mL) were added. The mixture was vigorously stirred for 30 min. Subsequently, pyrrole **2b** (15 mmol, 1.0 equiv) was added and the suspension was stirred for 30 min and cooled to 0 °C. 2-Methylbenzyl chloride (22 mmol,1.5 equiv) was then added and the reaction mixture was heated and stirred at 60 °C for 36 h. Then, the reaction mixture was poured into a flask containing 200 mL of deionized H_2O and extracted with Et_2O (4 x 40 mL). The combined organic layers were washed with LiCl-saturated H_2O solutions (3 x 20 mL), H_2O (1 x 20 mL) and then dried over Na_2SO_4. Solvent was removed under reduced pressure to give a dark brown oil which was purified by silica gel column chromatography (9/1 Hex/AcOEt) to afford **2m**, Yield: 1.53 g, 60%. **^{1}H NMR** (600 MHz, $CDCl_3$, 298 K): δ 2.47 (s, 3H, $-CH_3$), 5.21 (s, 2H, $-CH_2$), 6.43-6.44 (m, 2H, $ArH_{pyrrole}$), 6.85 (m, 2H, $ArH_{pyrrole}$), 7.08 (d, J = 7.5 Hz, 2H, ArH), 7.35-7.43 (*overlapped*, 3H, ArH). **^{13}C NMR** (150 MHz, $CDCl_3$, 298 K): δ 18.9, 51.4, 108.4, 121.1, 126.4, 127.85, 127.89, 130.4, 135.9, 136.1. Anal. Calcd for $C_{12}H_{13}N$: C 84.17, H 7.65, N 8.18. Found: C 84.19, H 7.64, N 8.17.

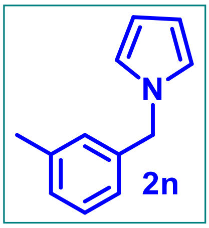

Derivative 2n. Obtained as a brownish oil following procedure reported in ref. **25**. Obtained as a brownish oil adapting a procedure reported in ref. **5**. To a 100 mL round bottomed flask, finely powdered KOH (59 mmol, 4.0 equiv) and DMSO (20 mL) were added. The mixture was vigorously stirred for 30 min. Subsequently, pyrrole **2b** (15 mmol, 1.0 equiv) was added and the suspension was stirred for another 30 min and cooled to 0 °C. 3-Methylbenzyl chloride (22 mmol,1.5 equiv) was then added and the reaction mixture was stirred and

heated at 60 °C for 36 h. Then, reaction mixture was poured into a flask containing 200 mL of deionized H_2O and extracted with Et_2O (4 x 40 mL). The combined organic layers were washed with LiCl-saturated H_2O solutions (3 x 20 mL), H_2O (1 x 20 mL) and then dried over Na_2SO_4. Solvent was removed under reduced pressure to give a dark brown oil which was purified by silica gel column chromatography (9/1 Hex/AcOEt) to afford **2n**. Yield: 1.79 g, 70 %. **^{1}H NMR** (600 MHz, $CDCl_3$, 298 K): δ 2.45 (s, 3H, -CH_3), 5.13 (s, 2H, -CH_2), 6.33 (m, 2H, $ArH_{pyrrole}$), 6.81 (m, 2H, $ArH_{pyrrole}$), 7.05 (d, J = 7.6 Hz, 1H, ArH), 7.08 (s, 1H, ArH), 7.21 (d, J = 7.5 Hz, 1H, ArH), 7.32-7.35 (m, 1H, ArH). **^{13}C NMR** (150 MHz, $CDCl_3$, 298 K): δ 21.5, 53.4, 108.6, 121.2, 124.3, 127.9, 128.5, 128.7, 138.2, 138.5. Anal. Calcd for $C_{12}H_{13}N$: C 84.17, H 7.65, N 8.18. Found: C 84.15, H 7.66, N 8.19.

IX. Characterization data for amides 4ac-ge

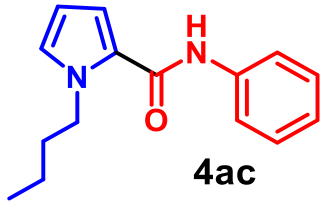

4ac

Derivative 4ac. Following the general procedure **GP2**, the pure derivative **4ac** was isolated by FC on silica gel (using as eluent hexane/ethyl acetate gradient, from 100:0 to 90:10) as a clear oil (39.0 mg, 99 % yield). **^{1}H NMR** (600 MHz, $CDCl_3$, 298 K): δ 0.93 (t, J = 7.3 Hz, 3H, CH_3), 1.33 (m, 2H, CH_2), 1.79 (m, 2H, CH_2), 4.37 (*t*, J = 7.3 Hz, 2H, NCH_2), 6.14 (dd, J_1 = 3.5 Hz, J_2 = 2.8 Hz, 1H, $ArH_{pyrrole}$), 6.70 (dd, J_1 = 3.7 Hz, J_2 = 1.1 Hz, 1H, $ArH_{pyrrole}$), 6.84 (dd, J_1 = J_2 = 1.9Hz, 1H, $ArH_{pyrrole}$), 7.11 (t, J = 7.4 Hz, 1H, ArH), 7.34 (dd, J_1 = J_2 = 7.9 Hz, 2H, ArH), 7.55 (d, J = 7.9 Hz, 2H, ArH), 7.62 (s, 1H, NH). **^{13}C NMR** (150 MHz, $CDCl_3$, 298 K): δ 13.9, 20.1, 34.1, 49.1, 107.6, 112.6, 120.2, 124.2, 125.3, 128.1, 129.2, 138.3, 160.0. Anal. Calcd for $C_{15}H_{18}N_2O$: C 74.35, H 7.49, N 11.56. Found: C 74.37, H 7.48, N 11.55.

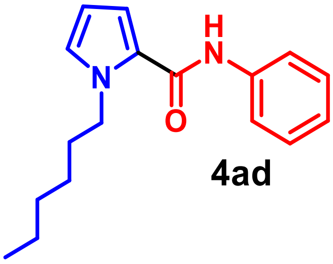

4ad

Derivative 4ad. Following the general procedure **GP2**, the derivative **4ad** was isolated by FC on silica gel (using as eluent hexane/ethyl acetate gradient, from 100:0 to 90:10) as a clear oil (43.5 mg, 99% yield). **^{1}H NMR** (600 MHz, $CDCl_3$, 298 K): δ 0.86 (t, J = 6.8 Hz, 3H, CH_3), 1.26-1.31 (*overlapped*, 6H, $NCH_2CH_2\mathit{CH_2CH_2CH_2}CH_3$), 1.79 (m, 2H, $NCH_2\mathit{CH_2}$), 4.36 (t, J = 7.3 Hz, 2H, NCH_2), 6.14 (dd, J_1 = 3.9 Hz, J_2 = 2.6 Hz, 1H, $ArH_{pyrrole}$), 6.69 (dd, J_1 = 3.9 Hz, J_2 = 1.6 Hz, 1H, $ArH_{pyrrole}$), 6.84 (dd, J_1 = 2.5 Hz, J_2 = 1.8 Hz, 1H, $ArH_{pyrrole}$), 7.11 (t, J = 7.4 Hz, 1H, ArH), 7.35 (dd, J_1 = 8.3 Hz, J_2 = 7.5 Hz, 2H, ArH), 7.55 (d, J = 7.6 Hz, 2H, ArH), 7.58 (s, -NH, 1H). **^{13}C NMR** (150 MHz, $CDCl_3$, 298 K): δ 14.2, 22.8, 26.6, 31.6, 32.0, 49.4, 107.6, 112.6, 120.2, 124.2, 125.3, 128.0, 129.2, 138.3, 160.0. Anal. Calcd for $C_{17}H_{22}N_2O$: C 75.52, H 8.20, N 10.36. Found: C 75.54, H 8.19, N 10.35.

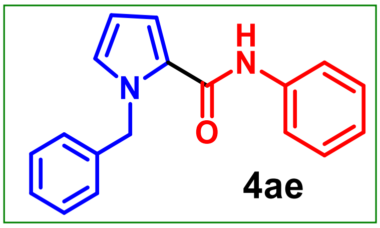

Derivative 4ae. Following the general procedure **GP2**, pure derivative **4ae** was isolated by FC on silica gel (using as eluent hexane/ethyl acetate gradient, from 100:0 to 90:10) as a white solid (44.5 mg, 99% yield). **m.p.** 115.2-116.2 °C. **^{1}H NMR** (600 MHz, $CDCl_3$, 298 K): δ 5.63 (s, 2H, NCH_2), 6.19 (dd, J_1 = 3.7 Hz, J_2 = 2.9 Hz, 1H, $ArH_{pyrrole}$), 6.73 (dd, J_1 = 3.9 Hz, J_2 = 1.4 Hz, 1H, $ArH_{pyrrole}$), 6.86 (br, 1H, $ArH_{pyrrole}$), 7.09 (t, J = 7.4 Hz, 1H, ArH), 7.15 (d, J = 7.3 Hz, 2H, ArH), 7.23-7.32 (overlapped, 5H, ArH), 7.51 (d, J = 7.9 Hz, 2H, ArH), 7.60 (br s, 1H, NH). **^{13}C NMR** (150 MHz, $CDCl_3$, 298 K): δ 52.1, 108.4, 112.9, 120.2, 124.3, 125.8, 127.4, 127.7, 128.3, 128.8, 129.2, 138.2, 138.6, 160.0. Anal. Calcd for $C_{18}H_{16}N_2O$: C 78.24, H 5.84, N 10.14. Found: C 78.22, H 5.85, N 10.15.

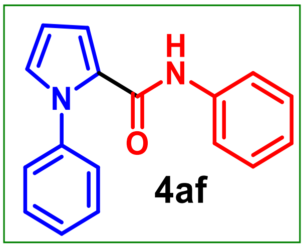

Derivative 4af. Following the general procedure **GP2**, pure derivative **4af** was isolated by FC on silica gel (using as eluent hexane/ethyl acetate gradient, from 100:0 to 90:10) as a tan solid (31.5 mg, 74 % yield). **m.p.** 114.1-115.0 °C. **^{1}H NMR** (600 MHz, $CDCl_3$, 298 K): δ 6.33 (dd, J_1 = 3.6 Hz, J_2 = 2.8 Hz, 1H, $ArH_{pyrrole}$), 6.93 (dd, J_1 = 3.8 Hz, J_2 = 1.7 Hz, 1H, $ArH_{pyrrole}$), 6.96 (dd, J_1= 2.6 Hz, J_2 = 1.8 Hz, 1H, $ArH_{pyrrole}$), 7.06 (t, J = 7.4 Hz, 1H, ArH), 7.27 (dd, J_1 = J_2 = 8.2 Hz, 2H, ArH), 7.37-7.47 (*overlapped*, 8H, ArH + NH). **^{13}C NMR** (150 MHz, $CDCl_3$, 298 K): δ 109.3, 115.0, 119.9, 124.2, 126.1, 127.6, 128.2, 128.8, 129.2, 129.3, 138.2, 140.4, 159.0. Anal. Calcd for $C_{17}H_{14}N_2O$: C 77.84, H 5.38, N 10.68. Found: C 77.86, H 5.37, N 10.67.

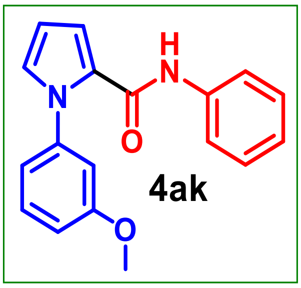

Derivative 4ak. Following the general procedure **GP2**, pure derivative **4ak** was isolated by FC on silica gel (using as eluent hexane/ethyl acetate gradient, from 100:0 to 80:20) as a white solid (10.4 mg, 22% yield). **m.p.** 139.2-139.7 °C.^{1}**H NMR** (600 MHz, $CDCl_3$, 298 K): δ 3.81 (s, 3H, OCH_3), 6.32 (dd, J_1 = 3.7 Hz, J_2 = 2.8 Hz, 1H, $ArH_{pyrrole}$), 6.91-6.98 (*overlapped*, 4H, $ArH_{pyrrole}$ + ArH), 7.06 (t, J = 7.4 Hz, 1H, ArH), 7.27-7.29 (m, 2H, ArH), 7.35 (t, J = 8.0 Hz, 2H, ArH), 7.40-7.41 (*overlapped*, 3H, ArH + NH). ^{13}**C NMR** (150 MHz, $CDCl_3$, 298 K): δ 55.7, 109.3, 112.0, 114.0, 115.3, 118.5, 119.9, 124.2, 127.7, 128.7, 129.2, 130.0, 138.2, 141.4, 158.9, 160.3. Anal. Calcd for $C_{18}H_{16}N_2O_2$: C 73.95, H 5.52, N 9.58. Found: C 73.93, H 5.53, N 9.59.

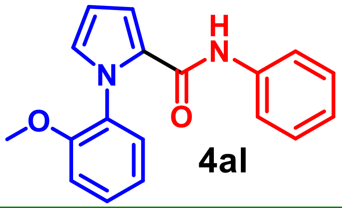

4al

Derivative 4al. Following the general procedure **GP2**, pure derivative **4al** was isolated by FC on silica gel (using as eluent hexane/ethyl acetate gradient, from 100:0 to 80:20) as a white solid (47.0 mg, 99% yield). **m.p.** 135.0-135.6°C. ^{1}**H NMR** (600 MHz, $CDCl_3$, 298 K): δ 3.74 (s, 3H, OCH_3), 6.34 (dd, J_1 = 3.8 Hz, J_2 = 2.8 Hz, 1H, $ArH_{pyrrole}$), 6.87 (dd, J_1 = 2.6 Hz, J_2= 1.7 Hz, 1H, $ArH_{pyrrole}$), 6.90 (dd, J_1 = 3.8 Hz, J_2 = 1.7 Hz, 1H, $ArH_{pyrrole}$), 7.04-7.07 (m, 2H, ArH), 7.25-7.28 (m, 2H, ArH), 7.35 (dd, J_1 = 7.7 Hz, J_2 = 1.6 Hz, 1H, ArH), 7.38 (dd, J_1 = 7.9 Jz, J_2 = 1.6 Hz, 1H, ArH), 7.41 (d, J = 7.7 Hz, 2H, ArH), 7.47 (s, 1H, NH). ^{13}**C NMR** (150 MHz, $CDCl_3$, 298 K): δ 56.1, 109.0, 112.2, 113.5, 119.8, 121.1, 124.0, 127.7, 128.3, 128.6, 129.1, 129.59, 129.63, 138.3, 154.5, 159.2. Anal. Calcd for $C_{18}H_{16}N_2O_2$: C 73.95, H 5.52, N 9.58. Found: C 73.97, H 5.51, N 9.57.

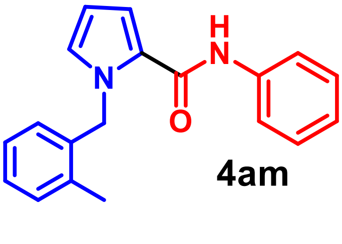

4am

Derivative 4am. Following the general procedure **GP2**, pure derivative **4am** was isolated by FC on silica gel (using as eluent hexane/ethyl acetate gradient, from 100:0 to 85:15) as a yellow solid (46.7 mg, 99% yield). **m.p.** 119.1-119.7 °C. **^{1}H NMR** (600 MHz, $CDCl_3$, 298 K): *δ* 2.31 (s, 3H, CH_3), 5.65 (s, 2H, CH_2), 6.20 (dd, J_1 = 3.8 Hz, J_2 = 2.7 Hz, 1H, $ArH_{pyrrole}$), 6.71 (m, 1H, $ArH_{pyrrole}$), 6.76 (d, *J* = 7.6, 1H, ArH), 6.79 (dd, J_1 = 3.7 Hz, J_2 = 1.5 Hz, 1H, $ArH_{pyrrole}$), 7.09-7.15 (m, 2H, ArH), 7.19-7.20 (m, 2H, ArH), 7.32-7.34 (m, 2H, ArH), 7.54 (d, *J* = 7.8 Hz, 2H, ArH), 7.68 (s, 1H, NH). **^{13}C NMR** (150 MHz, $CDCl_3$, 298 K): *δ* 19.1, 50.4, 108.2, 112.8, 120.2, 124.2, 126.1, 126.5, 127.4, 127.8, 128.0, 129.2, 130.5, 136.1, 136.5, 138.2, 160.0. Anal. Calcd for $C_{19}H_{18}N_2O$: C 78.59, H 6.25, N 9.65. Found: C 78.57, H 6.26, N 9.66.

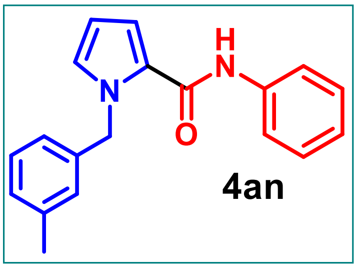

Derivative 4an. Following the general procedure **GP2**, pure derivative **4an** was isolated by FC on silica gel (using as eluent hexane/ethyl acetate gradient, from 100:0 to 85:15) as a yellow solid (11.8 mg, 25% yield). **m.p.** 120.6-121.4 °C. **^{1}H NMR** (600 MHz, $CDCl_3$, 298 K): *δ* 2.31 (s, 3H, CH_3), 5.61 (s, 2H, NCH_2), 6.20 (dd, J_1 = 3.6 Hz, J_2 = 2.8 Hz, 1H, $ArH_{pyrrole}$), 6.74 (dd, J_1 = 3.9 Hz, J_2 = 1.4 Hz, 1H, $ArH_{pyrrole}$), 6.86 (dd, J_1 = J_2 = 2.1 Hz, 1H, $ArH_{pyrrole}$), 6.96 (d, *J* = 7.2 Hz, 1H, ArH), 7.00 (s, 1H, ArH), 7.06 (d, *J* = 7.8 Hz, 1H, ArH), 7.09-7.11 (m, 1H, ArH), 7.17-7.20 (m, 1H, ArH), 7.32-7.34 (m, 2H, ArH), 7.54 (d, *J* = 8.2 Hz, 2H, ArH), 7.61 (s, 1H, NH). **^{13}C NMR** (150 MHz, $CDCl_3$, 298 K): *δ* 21.6, 52.0, 108.3, 112.9, 120.2, 124.2, 124.5, 125.8, 128.1, 128.2, 128.4, 128.7, 129.2, 138.2, 138.4, 160.0. Anal. Calcd for $C_{19}H_{18}N_2O$: C 78.59, H 6.25, N 9.65. Found: C 78.61, H 6.24, N 9.64.

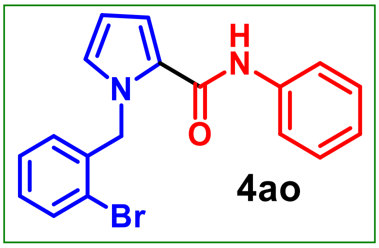

Derivative 4ao. Following the general procedure **GP2**, pure derivative **4ao** was isolated by FC on silica gel (using as eluent hexane/ethyl acetate gradient, from 100:0 to 85:15) as a brown solid (57 mg, 99% yield). **m.p.** 127.9-128.7°C. ^{1}**H NMR** (600 MHz, $CDCl_3$, 298 K): δ 5.72 (s, 2H, CH_2), 6.24 (dd, J_1 = 3.9 Hz, J_2 = 2.4 Hz, 1H, $ArH_{pyrrole}$), 6.71 (d, J = 7.2 Hz, 1H, ArH), 6.79 (dd, J_1 = 3.9 Hz, J_2 = 1.5 Hz, 1H, $ArH_{pyrrole}$), 6.85-6.86 (m, 1H, $ArH_{pyrrole}$), 7.08-7.12 (m, 2H, ArH), 7.19-7.21 (m, 1H, ArH), 7.31-7.34 (m, 1H, ArH), 7.52 (d, J = 7.9 Hz, 2H, ArH), 7.54 (d, J = 7.3 Hz, 1H, ArH), 7.62 (s, 1H, NH). ^{13}**C NMR** (150 MHz, $CDCl_3$, 298 K): δ 52.4, 108.7, 112.8, 120.2, 122.6, 123.6, 124.3, 126.1, 128.0, 128.4, 129.1, 129.3, 132.9, 138.1, 138.2, 159.7. Anal. Calcd for $C_{18}H_{15}BrN_2O$: C 60.86, H 4.26, N 7.89. Found: C 60.84, H 4.27, N 7.90.

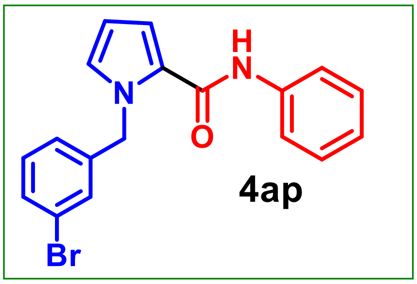

Derivative 4ap. Following the general procedure **GP2**, pure derivative **4ap** was isolated by FC on silica gel (using as eluent hexane/ethyl acetate gradient, from 100:0 to 85:15) as a white solid (14.4 mg, 25% yield). **m.p.** 126.5-127.1°C. ^{1}**H NMR** (400 MHz, $CDCl_3$, 298 K): δ 5.61 (s, 2H, CH_2), 6.23 (dd, J_1 = 3.9 Hz, J_2 = 2.8 Hz, 1H, $ArH_{pyrrole}$), 6.76 (dd, J_1 = 4.0 Hz, J_2 = 1.6 Hz, 1H, $ArH_{pyrrole}$), 6.87 (dd, J_1 = 2.5 Hz, J_2 = 1.8 Hz, 1H, $ArH_{pyrrole}$), 7-07-7.12 (m, 2H, ArH), 7.14-7.18 (m, 1H, ArH), 7.31-7.37 (*overlapped*, 4H, ArH), 7.52 (d, J = 7.7 Hz, 2H, ArH), 7.61 (s, 1H, NH). ^{13}**C NMR** (150 MHz, $CDCl_3$, 298 K): δ 51.5, 108.7, 113.1, 120.3, 122.9, 124.3, 125.6, 125.9, 128.4, 129.3, 130.1, 130.4, 130.8, 138.0, 141.0, 159.8. Anal. Calcd for $C_{18}H_{15}BrN_2O$: C 60.86, H 4.26, N 7.89. Found: C 60.88, H 4.25, N 7.88.

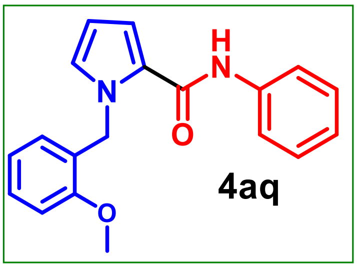

Derivative 4aq. Following the general procedure **GP2**, pure derivative **4aq** was isolated by FC on silica gel (using as eluent hexane/ethyl acetate gradient, from 100:0 to 80:20) as a grey solid (49.3 mg, 99% yield). **m.p.** 105.5-106.3 °C. **^{1}H NMR** (400 MHz, $CDCl_3$, 298 K): δ 3.83 (s, 3H, OCH_3), 5.65 (s, 2H, CH_2), 6.16 (dd, J_1 = 3.6 Hz, J_2 = 2.4 Hz, 1H, $ArH_{pyrrole}$), 6.73 (dd, J_1 = 3.8 Hz, J_2 = 1.3 Hz, 1H, $ArH_{pyrrole}$), 6.84-6.91 (*overlapped*, 4H, $ArH_{pyrrole}$ + ArH), 7.08-7.10 (m, 1H, ArH), 7.21-7.25 (m, 1H, ArH), 7.30-7.32 (m, 2H, ArH), 7.54 (d, J = 8.0 Hz, 2H, ArH), 7.65 (s, 1H, NH). **^{13}C NMR** (100 MHz, $CDCl_3$, 298 K): δ 47.1, 55.4, 108.0, 110.3, 112.6, 120.1, 120.9, 124.1, 126.0, 127.1, 128.6, 128.7, 128.9, 129.2, 138.3, 157.0, 160.1. Anal. Calcd for $C_{19}H_{18}N_2O_2$: C 74.49, H 5.92, N 9.14. Found: C 74.47, H 5.93, N 9.15.

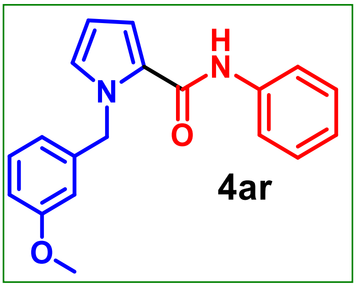

4ar

Derivative 4ar. Following the general procedure **GP2**, pure derivative **4ar** was isolated by FC on silica gel (using as eluent hexane/ethyl acetate gradient, from 100:0 to 80:20) as a white solid (10.9 mg, 22% yield). **m.p.** 111.3-112.0°C. **^{1}H NMR** (400 MHz, $CDCl_3$, 298 K): δ 3.75 (s, 3H, OCH_3), 5.62 (s, 2H, CH_2), 6.19-6.21 (m, 1H, $ArH_{pyrrole}$), 6.70 (br s, 1H, $ArH_{pyrrole}$), 6.74-6.79 (*overlapped*, 3H, ArH), 6.87 (s, 1H, ArH), 7.10 (t, J = 7.4 Hz, 1H, ArH), 7.19-7.23 (m, 1H, ArH), 7.30-7.34 (m, 2H, ArH), 7.53 (d, J = 8.4 Hz, 2H, ArH), 7.63 (s, 1H, NH). **^{13}C NMR** (100 MHz, $CDCl_3$, 298 K): δ 52.0, 55.3, 108.4, 112.92, 112.94, 113.0, 119.6, 120.1, 124.2, 125.8, 128.4, 129.2, 129.8, 138.2, 140.2, 159.9, 160.0. Anal. Calcd for $C_{19}H_{18}N_2O_2$: C 74.49, H 5.92, N 9.14. Found: C 74.51, H 5.91, N 9.13.

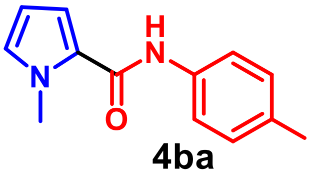

4ba

Derivative 4ba. Following the general procedure **GP2**, pure derivative **4ba** was isolated by FC on silica gel (using as eluent hexane/ethyl acetate gradient, from 100:0 to 90:10) as a

yellow oil (34.5 mg, 99% yield). **^{1}H NMR** (600 MHz, $CDCl_3$, 298 K): δ 2.33 (s, 3H, -CH_3), 3.98 (s, 3H, NCH_3), 6.13-6.14 (m, 1H, $ArH_{pyrrole}$), 6.68 (dd, J_1 = 4.2 Hz, J_2 = 1.2 Hz, 1H, $ArH_{pyrrole}$), 6.77 (br s, J = 1.8Hz, 1H, $ArH_{pyrrole}$), 7.15 (d, J = 8.4 Hz, 2H, ArH), 7.43 (d, J = 8.4 Hz, 2H, ArH), 7.53 (s, 1H, NH). **^{13}C NMR** (150 MHz, $CDCl_3$, 298 K): δ 21.1, 37.0, 107.6, 112.2, 120.3, 126.1, 128.9, 129.7, 133.9, 135.6, 160.1. Anal. Calcd for $C_{13}H_{14}N_2O$: C 72.87, H 6.59, N 13.87. Found: C 72.89, H 6.58, N 13.86.

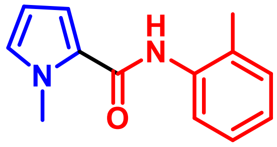

4ca

Derivative 4ca. Following the general procedure **GP2**, pure derivative **4ca** was isolated by FC on silica gel (using as eluent hexane/ethyl acetate gradient, from 100:0 to 90:10) as a glassy white solid (34.5 mg, 99% yield). **m. p.** 115.4-115.9 °C.**^{1}H NMR** (600 MHz, $CDCl_3$, 298 K): δ 2.32 (s, 3H, CH_3), 3.99 (s, 3H, NCH_3), 6.16 (dd, J_1 = 3.9 Hz, J_2 = 1.8 Hz, 1H, $ArH_{pyrrole}$), 6.70 (dd, J_1 = 3.9 Hz, J_2 = 1.8 Hz, 1H, $ArH_{pyrrole}$), 6.79-6.80 (m, 1H, $ArH_{pyrrole}$), 7.07-7.09 (m, 1H, ArH), 7.21 (d, J = 7.5 Hz, 1H, ArH), 7.23-7.25 (m, 1H, ArH), 7.46 (s, 1H, NH), 7.90 (d, J = 7.9 Hz, 1H, ArH). **^{13}C NMR** (150 MHz, $CDCl_3$, 298 K): δ 18.0, 37.0, 107.7, 112.1, 122.9, 125.0, 126.1, 127.0, 128.90, 128.93, 130.7, 136.1, 160.1. Anal. Calcd for $C_{13}H_{14}N_2O$: C 72.87, H 6.59, N 13.87. Found: C 72.85, H 6.60, N 13.88.

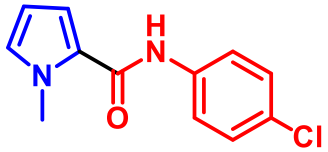

4da

Derivative 4da. Following the general procedure **GP2**, pure derivative **4da** was isolated by FC on silica gel (using as eluent hexane/ethyl acetate gradient, from 100:0 to 80:20) as a yellow solid (34.3 mg, 90% yield). **m. p.** 133.9-134.3 °C. **^{1}H NMR** (600 MHz, $CDCl_3$, 298 K): δ 3.97 (s, 3H, NCH_3), 6.14 (dd, J_1 = 4.0 Hz, J_2 = 2.6 Hz, 1H, $ArH_{pyrrole}$), 6.69 (dd, J_1 = 3.9 Hz, J_2 = 1.5 Hz, 1H, $ArH_{pyrrole}$), 6.79-6.80 (m, 1H, $ArH_{pyrrole}$), 7.30 (d, J = 8.9 Hz, 2H, ArH), 7.51 (d, J = 8.8 Hz, 2H, ArH), 7.58 (s, 1H, NH). **^{13}C NMR** (150 MHz, $CDCl_3$, 298 K): δ 37.1, 107.8,

112.6, 121.4, 125.6, 129.1, 129.2, 129.3, 136.9, 160.0. Anal. Calcd for $C_{12}H_{11}ClN_2O$: C 61.42, H 4.72, N 11.94. Found: C 61.40, H 4.73, N 11.95.

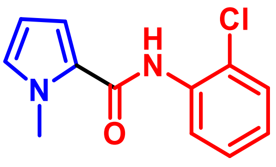

4ea

Derivative 4ea. Following the general procedure **GP2**, pure derivative **4ea** was isolated by FC on silica gel (using as eluent hexane/ethyl acetate gradient, from 100:0 to 80:20) as a white solid (35.1 mg, 92% yield). **m. p.** 101.2-102.0°C. **^{1}H NMR** (400 MHz, $CDCl_3$, 298 K): δ 3.99 (s, 3H, NCH_3), 6.16-6.18 (m, 1H, $ArH_{pyrrole}$), 6.79-6.82 (*overlapped*, 2H, $ArH_{pyrrole}$), 7.01-7.05 (m, 1H, ArH), 7.27-7.31 (m, 1H, ArH), 7.39 (d, J = 7.9 Hz, 1H, ArH), 8.20 (s, 1H, NH), 8.43 (d, J = 8.4 Hz, 1H, ArH). **^{13}C NMR** (150 MHz, $CDCl_3$, 298 K): δ 37.2, 107.9, 113.0, 121.2, 122.8, 124.3, 125.7, 127.9, 129.2, 129.5, 135.2, 159.7. Anal. Calcd for $C_{12}H_{11}ClN_2O$: C 61.42, H 4.72, N 11.94. Found: C 61.44, H 4.71, N 11.93.

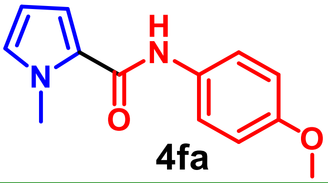

4fa

Derivative 4fa. Following the general procedure **GP2**, pure derivative **4fa** was isolated by FC on silica gel (using as eluent hexane/ethyl acetate gradient, from 100:0 to 80:20) as a white solid (37.1 mg, 99% yield). **m. p.** 112.8-113.6 °C. **^{1}H NMR** (600 MHz, $CDCl_3$, 298 K): δ 3.80 (s, 3H, OCH_3), 3.97 (s, 3H, NCH_3), 6.13 (dd, J_1 = 3.8 Hz, J_2 = 2.7 Hz, 1H, $ArH_{pyrrole}$), 6.67 (dd, J_1 = 3.8 Hz, J_2 = 1.4 Hz, 1H, $ArH_{pyrrole}$), 6.77 (br s, 1H, $ArH_{pyrrole}$), 6.88 (d, J = 8.9 Hz, 2H, ArH), 7.45 (d, J = 8.9 Hz, 2H, ArH), 7.53 (s, 1H, -NH-). **^{13}C NMR** (150 MHz, $CDCl_3$, 298 K): δ 37.0, 55.7, 107.6, 112.1, 114.4, 122.2, 126.0, 128.8, 131.2, 156.5, 160.1. Anal. Calcd for $C_{13}H_{14}N_2O_2$: C 67.81, H 6.13, N 12.17. Found: C 67.83, H 6.12, N 12.16.

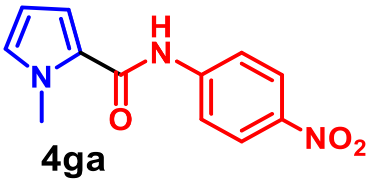

4ga

Derivative 4ga. Following the general procedure **GP2**, pure derivative **4ga** was isolated by FC on silica gel (using as eluent hexane/ethyl acetate gradient, from 100:0 to 70:30) as a yellow solid (33.9 mg, 85% yield). **m. p.** 199.7-200.2 °C. **^{1}H NMR** (600 MHz, $CDCl_3$, 298 K): δ 3.99 (s, 3H, NCH_3), 6.18 (dd, J_1 = 3.9 Hz, J_2 = 2.4 Hz, 1H, $ArH_{pyrrole}$), 6.76-6.77 (m, 1H, $ArH_{pyrrole}$), 6.85 (br s, 1H, $ArH_{pyrrole}$), 7.75 (d, J = 9.1 Hz, 2H, ArH), 7.82 (s, 1H, NH), 8.23 (d, J = 9.1 Hz, 2H, ArH). **^{13}C NMR** (150 MHz, $CDCl_3$, 298 K): δ 37.3, 108.2, 113.5, 119.1, 125.0, 125.4, 130.3, 143.4, 144.4, 159.7. Anal. Calcd for $C_{12}H_{11}N_3O_3$: C 58.77, H 4.52, N 17.13. Found: C 58.79, H 4.51, N 17.12.

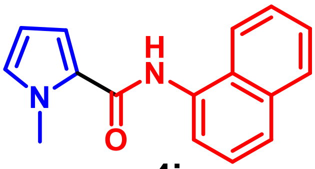

4ia

Derivative 4ia. Following the general procedure **GP2**, pure derivative **4ia** was isolated by FC on silica gel (using as eluent hexane/ethyl acetate gradient, from 100:0 to 90:10) as a white solid (37.0 mg, 91% yield). **m. p.** 117.1-117.6 °C. **^{1}H NMR** (600 MHz, $CDCl_3$, 298 K): δ 4.00 (s, 3H, NCH_3), 6.20 (dd, J_1 = 3.9 Hz, J_2 = 2.6 Hz, 1H, $ArH_{pyrrole}$), 6.82-6.83 (m, 1H, $ArH_{pyrrole}$), 6.86 (dd, J_1 = 3.9 Hz, J_2 = 1.5 Hz, $ArH_{pyrrole}$), 7.48-7.54 (*overlapped*, 3H, $ArH_{naphtyl}$), 7.72 (d, J = 8.22 Hz, 1H, $ArH_{naphtyl}$), 7.88-7.91 (*overlapped*, 2H, $ArH_{naphtyl}$), 7.95 (d, J = 7.44 Hz, 1H, $ArH_{naphtyl}$), 8.02 (s, 1H, NH). **^{13}C NMR** (150 MHz, $CDCl_3$, 298 K): δ 37.1, 107.8, 112.5, 121.0, 121.2, 125.8, 125. 88, 125.93, 126.2, 126.5, 127.7, 128.9, 129.0, 132.6, 134.4, 160.6. Anal. Calcd for $C_{16}H_{14}N_2O$: C 76.78, H 5.64, N 11.19. Found: C 76.80, H 5.63, N 11.18.

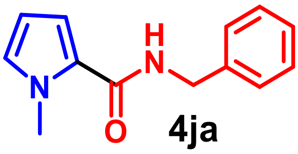

4ja

Derivative 4ja. Following the general procedure **GP2**, pure derivative **4ja** was isolated by FC on silica gel (using as eluent hexane/ethyl acetate gradient, from 100:0 to 95:5) as a

white solid (30.6 mg, 88% yield). **m. p.** 119.2-120.1°C. **^{1}H NMR** (600 MHz, $CDCl_3$, 298 K): δ 3.97 (s, 3H, NCH_3), 4.58 (d, J = 6 Hz, 2H, CH_2), 6.08 (dd, J_1 = 3.8 Hz, J_2 = 2.6 Hz, 1H, $ArH_{pyrrole}$), 6.13 (s, 1H, -NH-), 6.53 (dd, J_1 = 3.9 Hz, J_2 = 1,6 Hz, 1H, $ArH_{pyrrole}$), 6.72-6.73 (m, 1H, $ArH_{pyrrole}$), 7.28-7.30 (m, 1H, ArH), 7.34-7.35 (*overlapped*, 4H, ArH). **^{13}C NMR** (150 MHz, $CDCl_3$, 298 K): δ 37.0, 43.5, 107.4, 111.7, 125.8, 127.7, 128.0, 128.3, 129.0, 138.8, 162.0. Anal. Calcd for $C_{13}H_{14}N_2O$: C 72.87, H 6.59, N 13.07. Found: C 72.85, H 6.60, N 13.08.

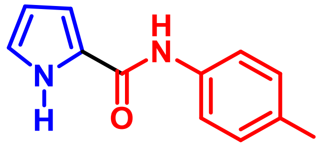

4bb

Derivative 4bb. Following the general procedure **GP2**, pure derivative **4bb** was isolated by FC on silica gel (using as eluent hexane/ethyl acetate gradient, from 100:0 to 80:20) as a yellow oil (29.0 mg, 89% yield). **^{1}H NMR** (600 MHz, $CDCl_3$, 298 K): δ 2.33 (s, 3H, CH_3), 6.29 (dd, J_1 = 6.3 Hz, J_2 = 3.0 Hz, 1H, $ArH_{pyrrole}$), 6.68 (br s, 1H, $ArH_{pyrrole}$), 6,98 (dd, J_1 = 3.8 Hz, J_2 = 2.6 Hz, 1H, $ArH_{pyrrole}$), 7.16 (d, J = 8.7 Hz, 2H, ArH), 7.46 (d, J = 8.4 Hz, 2H, ArH), 7.50 (s, 1H, NH). **^{13}C NMR** (100 MHz, $CDCl_3$, 298 K): δ 21.1, 109.4, 110.4, 120.3, 122.4, 126.3, 129.8, 134.1, 135.3, 159.1. Anal. Calcd for $C_{12}H_{12}N_2O$: C 71.98, H 6.04, N 13.99. Found: C 72.00, H 6.03, N 13.98.

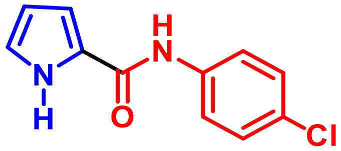

4db

Derivative 4db. Following the general procedure **GP2**, pure derivative **4db** was isolated by FC on silica gel (using as eluent hexane/ethyl acetate gradient, from 100:0 to 70:30) as a white solid (30.1 mg, 84% yield). **m.p.** 127.1-128.0°C. **^{1}H NMR** (400 MHz, $CDCl_3$, 298 K): δ 6.31 (dd, J_1 = 5.6 Hz, J_2 = 2.7 Hz, 1H, $ArH_{pyrrole}$), 6.71 (br s, 1H, $ArH_{pyrrole}$), 7.00 (br s, 1H, $ArH_{pyrrole}$), 7.32 (d, J = 8.8 Hz, 2H, ArH), 7.55 (s, 1H, NH), 7.56 (d, J = 8.8 Hz, 2H, ArH), 9.50 (s, 1H, $NH_{pyrrole}$). **^{13}C NMR** (150 MHz, $CDCl_3$, 298 K): δ 109.8, 110.6, 121.4, 122.7, 126.0, 129.3, 129.4, 136.6, 159.0. Anal. Calcd for $C_{11}H_9ClN_2O$: C 59.88, H 4.11, N 12.70. Found: C 59.90, H 4.10, N 12.69.

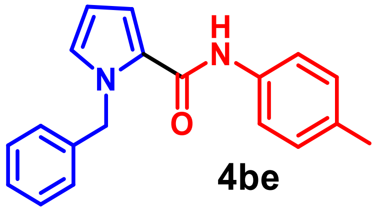

4be

Derivative 4be. Following the general procedure **GP2**, pure derivative **4fb** was isolated by FC on silica gel (using as eluent hexane/ethyl acetate gradient, from 100:0 to 70:30) as a white solid (40.6 mg, 86% yield). **m.p.** 125.4-126.2°C. **^{1}H NMR** (600 MHz, $CDCl_3$, 298 K): δ 2.33 (s, 3H,-CH_3), 5.65 (s, 2H, -CH_2), 6.21 (dd, J_1 = 3.8 Hz, J_2 = 2.7 Hz, 1H, $ArH_{pyrrole}$), 6.74 (dd, J_1 = 3.8 Hz, J_2 = 1.6 Hz, 1H, $ArH_{pyrrole}$), 6.87 (dd, J_1 = 2.4 Hz, J_2 = 1.6 Hz, 1H, $ArH_{pyrrole}$), 7.14 (d, J = 8.1 Hz, 2H, ArH), 7.19 (d, J = 8.3 Hz, 2H, ArH), 7.23-7.33 (*overlapped*, 3H, ArH), 7.43 (d, J = 8.5 Hz ,2H, ArH), 7.60 (s, 1H, NH). **^{13}C NMR** (100 MHz, $CDCl_3$, 298 K): δ 21.0, 52.0, 108.3, 112.7, 120.3, 125.9, 127.4, 127.6, 128.1, 128.8, 129.7, 133.8, 135.6, 138.6, 159.9. Anal. Calcd for $C_{19}H_{18}N_2O$: C 78.59, H 6.25, N 9.65. Found: C 78.57, H 6.26, N 9.66.

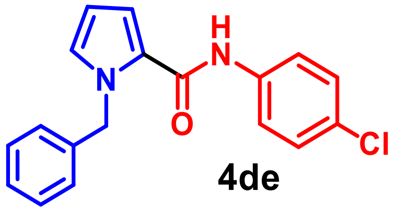

4de

Derivative 4de. Following the general procedure **GP2**, pure derivative **4de** was isolated by FC on silica gel (using as eluent hexane/ethyl acetate gradient, from 100:0 to 90:10) as a white solid (41.4 mg, 82% yield). **m.p.** 132.0-132.8°C. **^{1}H NMR** (600 MHz, $CDCl_3$, 298 K): δ 5.63 (s, 2H, CH_2), 6.20-6.21 (m, 1H, $ArH_{pyrrole}$), 6.74-6.75 (m, 1H, $ArH_{pyrrole}$), 6.89 (br s, 1H, $ArH_{pyrrole}$), 7.16 (d, J =7.4 Hz, 2H, ArH), 7.25-7.31 (*overlapped*, 4H, ArH), 7.47 (d, J = 8.7 Hz, 2H, ArH), 7.60 (s, 1H, NH). **^{13}C NMR** (150 MHz, $CDCl_3$, 298 K): δ 52.1, 108.4, 113.2, 121.4, 125.5, 127.3, 127.7, 128.6, 128.8, 129.1, 129.2, 136.8, 138.5, 159.8. Anal. Calcd for $C_{18}H_{15}ClN_2O$: C 69.57, H 4.87, N 9.01. Found: C 69.55, H 4.88, N 9.02.

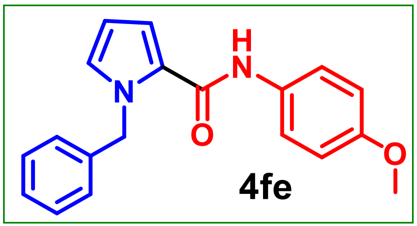

4fe

Derivative 4fe. Following the general procedure **GP2**, pure derivative **4fe** was isolated by FC on silica gel (using as eluent hexane/ethyl acetate gradient, from 100:0 to 80:20) as a white solid (42.8 mg, 86% yield). **m.p.** 137.1-138.0°C. **^{1}H NMR** (400 MHz, $CDCl_3$, 298 K): δ 3.79 (s, 3H, OCH_3), 5.63 (s, 2H, NCH_2), 6.18-6.20 (m, 1H, $ArH_{pyrrole}$), 6.71 (dd, J_1 = 3.9 Hz, J_2 = 1.4 Hz, 1H, $ArH_{pyrrole}$), 6.85-6.87 (*overlapped*, ArH + $ArH_{pyrrole}$, 3H), 7.16 (d, J = 7.0 Hz, 2H, ArH), 7.23-7.31 (*overlapped*, 3H, ArH), 7.42 (d, J = 8.9 Hz, 2H, ArH), 7.50 (s, 1H, NH). **^{13}C NMR** (150 MHz, $CDCl_3$, 298 K): δ 52.0, 55.7, 108.3, 112.6, 114.4, 122.3, 125.9, 127.4, 127.6, 128.0, 128.8, 131.2, 138.6, 156.6, 160.0. Anal. Calcd for $C_{19}H_{18}N_2O_2$: C 74.49, H 5.92, N 9.14. Found: C 74.51, H 5.91, N 9.13.

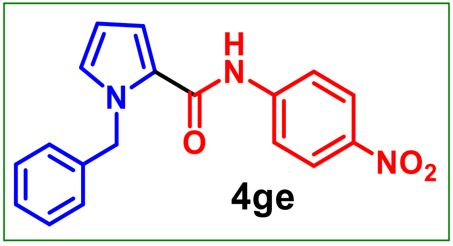

4ge

Derivative 4ge. Following the general procedure **GP2**, pure derivative **4ge** was isolated by FC on silica gel (using as eluent hexane/ethyl acetate gradient, from 100:0 to 80:20) as a yellow solid (46.5 mg, 89% yield). **m.p.** 168.4-169.2 °C. **^{1}H NMR** (400 MHz, $CDCl_3$, 298 K): δ 5.63 (s, 2H, CH_2), 6.24 (dd, J_1 = 4.1 Hz, J_2 = 2.7 Hz, 1H, $ArH_{pyrrole}$), 6.82 (dd, J_1 = 4.0 Hz, J_2 = 1.6 Hz, 1H, $ArH_{pyrrole}$), 6.94 (dd, J_1 = 2.5 Hz, J_2 = 1.6 Hz, 1H, $ArH_{pyrrole}$), 7.14 (d, J = 7.6 Hz, 2H, ArH), 7.24-7.33 (*overlapped*, 3H, ArH), 7.71 (d, J = 9.2 Hz, 2H, ArH), 7.86 (s, 1H, NH), 8.20 (d, J = 9.2 Hz, 2H, ArH). **^{13}C NMR** (100 MHz, $CDCl_3$, 298 K): δ 52.4, 108.8, 114.1, 119.2, 124.8, 125.3, 127.3, 127.8, 128.9, 129.6, 138.2, 143.4, 144.3, 159.5. Anal. Calcd for $C_{18}H_{15}N_3O_3$: C 67.28, H 4.71, N 13.08. Found: C 67.30, H 4.70, N 13.07.

X. ^{1}H and ^{13}C NMR Spectra and 2D NMR Spectra of Pyrrole Derivatives 2m-n.

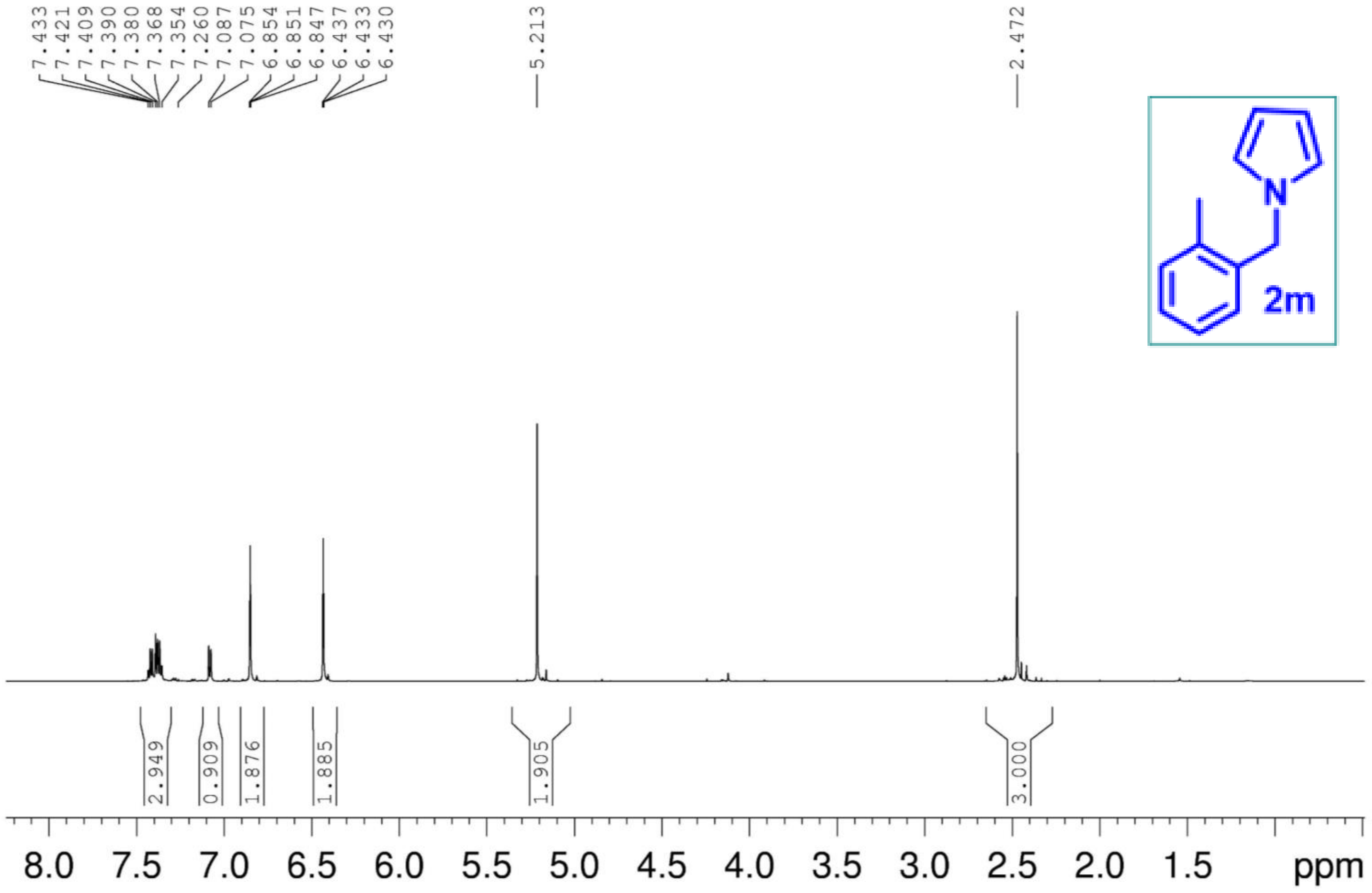

0

Figure S8. ^{1}H NMR spectrum (600 MHz, $CDCl_3$, 298 K) of the derivative **2m**.

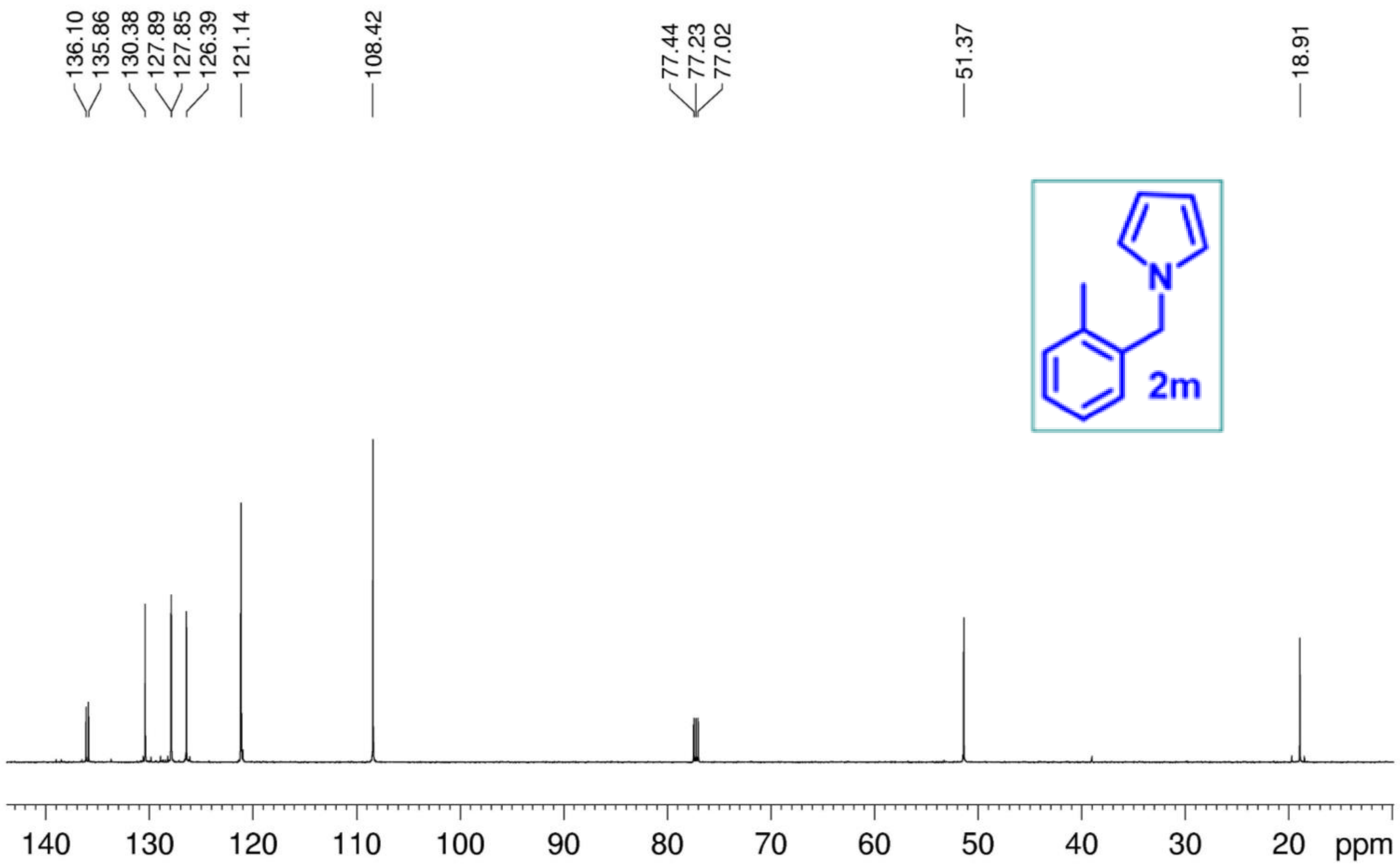

Figure S9. ^{13}C NMR spectrum (150 MHz, $CDCl_3$, 298 K) of the derivative **2m**.

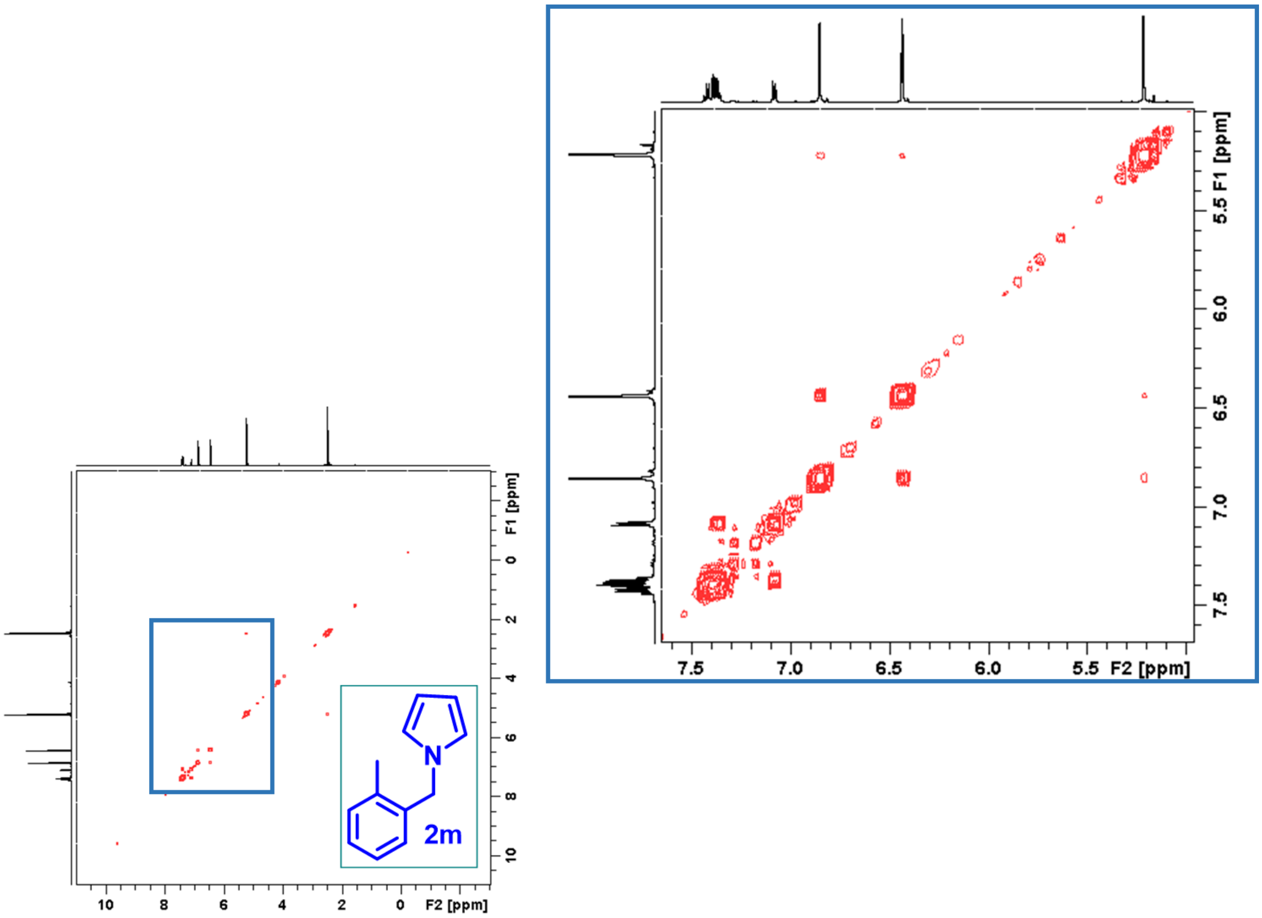

Figure S10. COSY NMR spectrum (600 MHz, $CDCl_3$, 298 K) of the derivative **2m**.

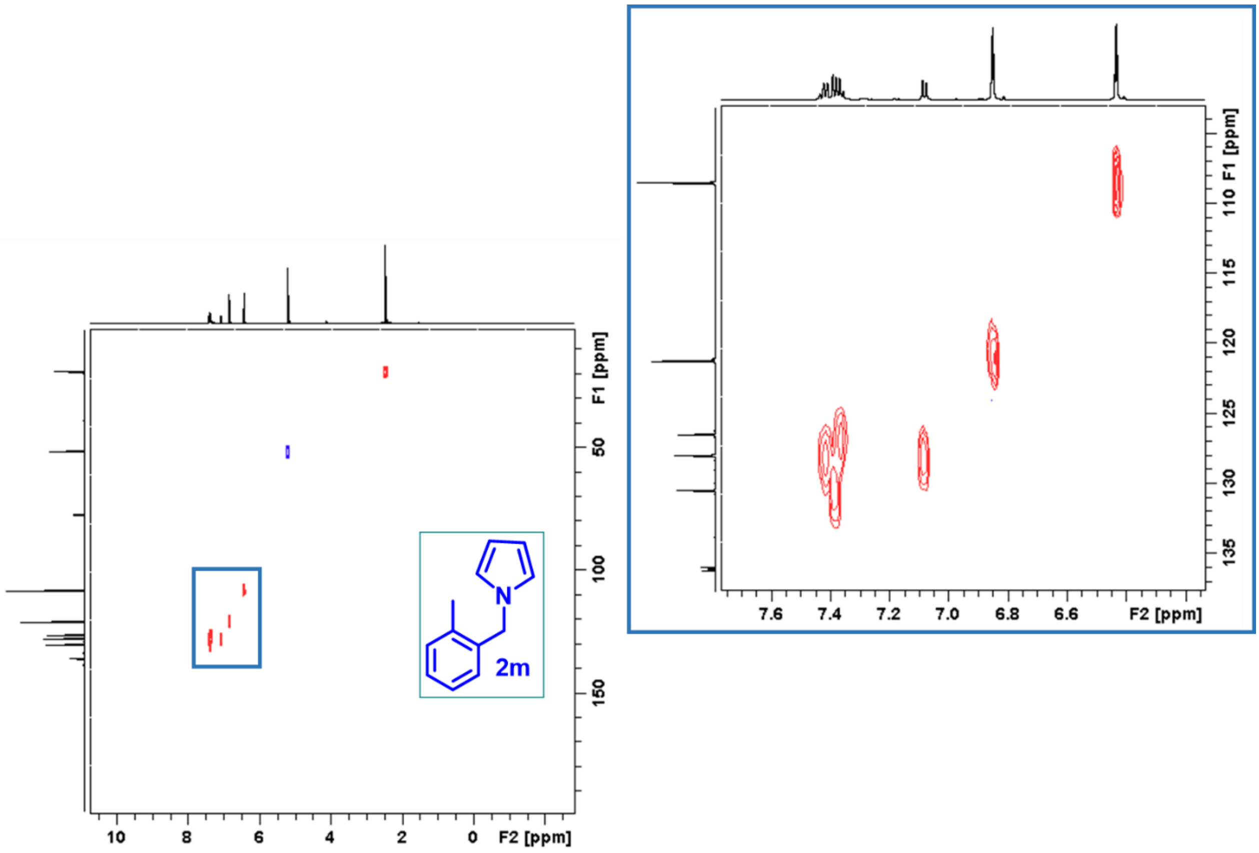

Figure S11. HSQC NMR spectrum (600 MHz, $CDCl_3$, 298 K) of the derivative **2m**.

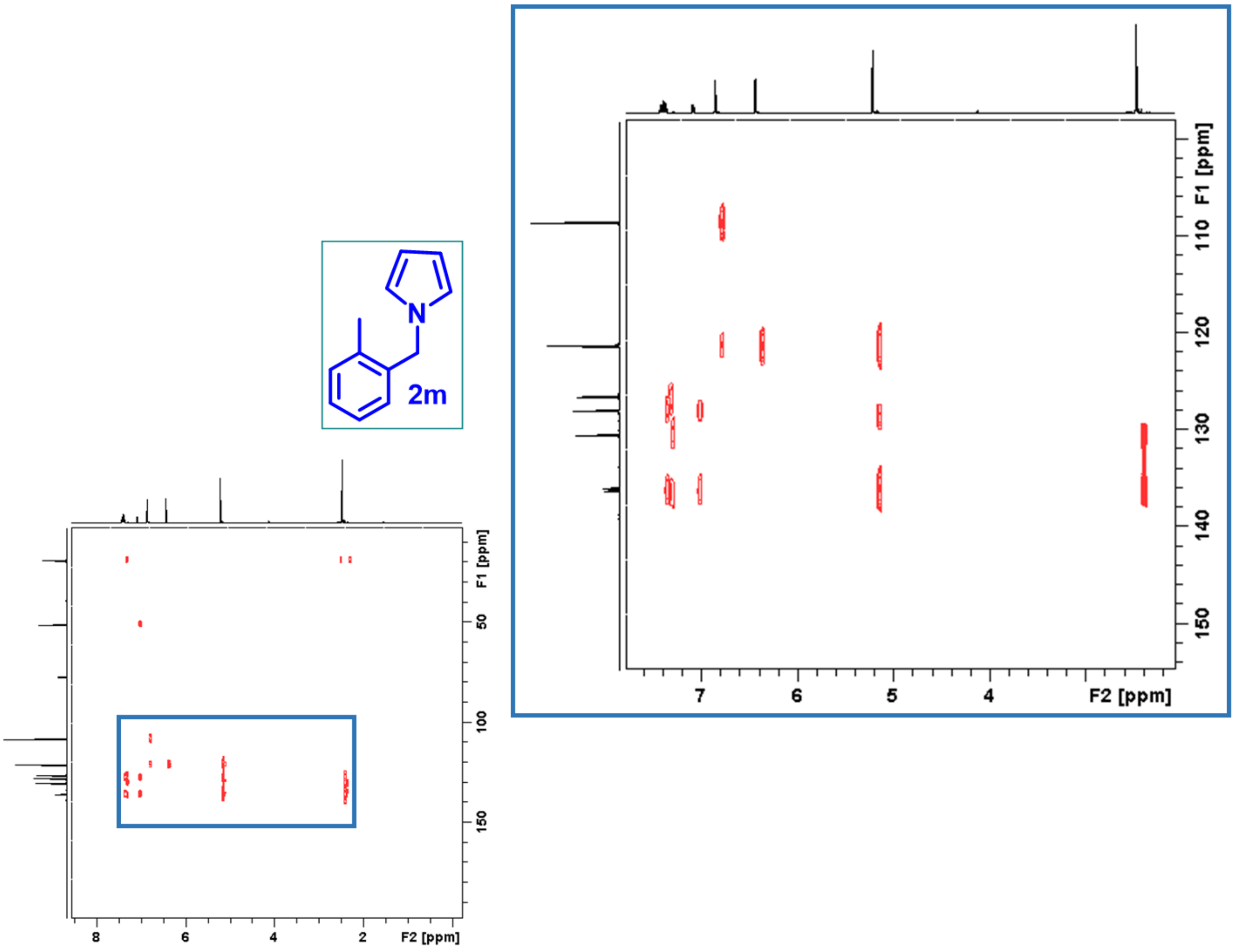

Figure S12. HMBC NMR spectrum (600 MHz, $CDCl_3$, 298 K) of the derivative **2m**.

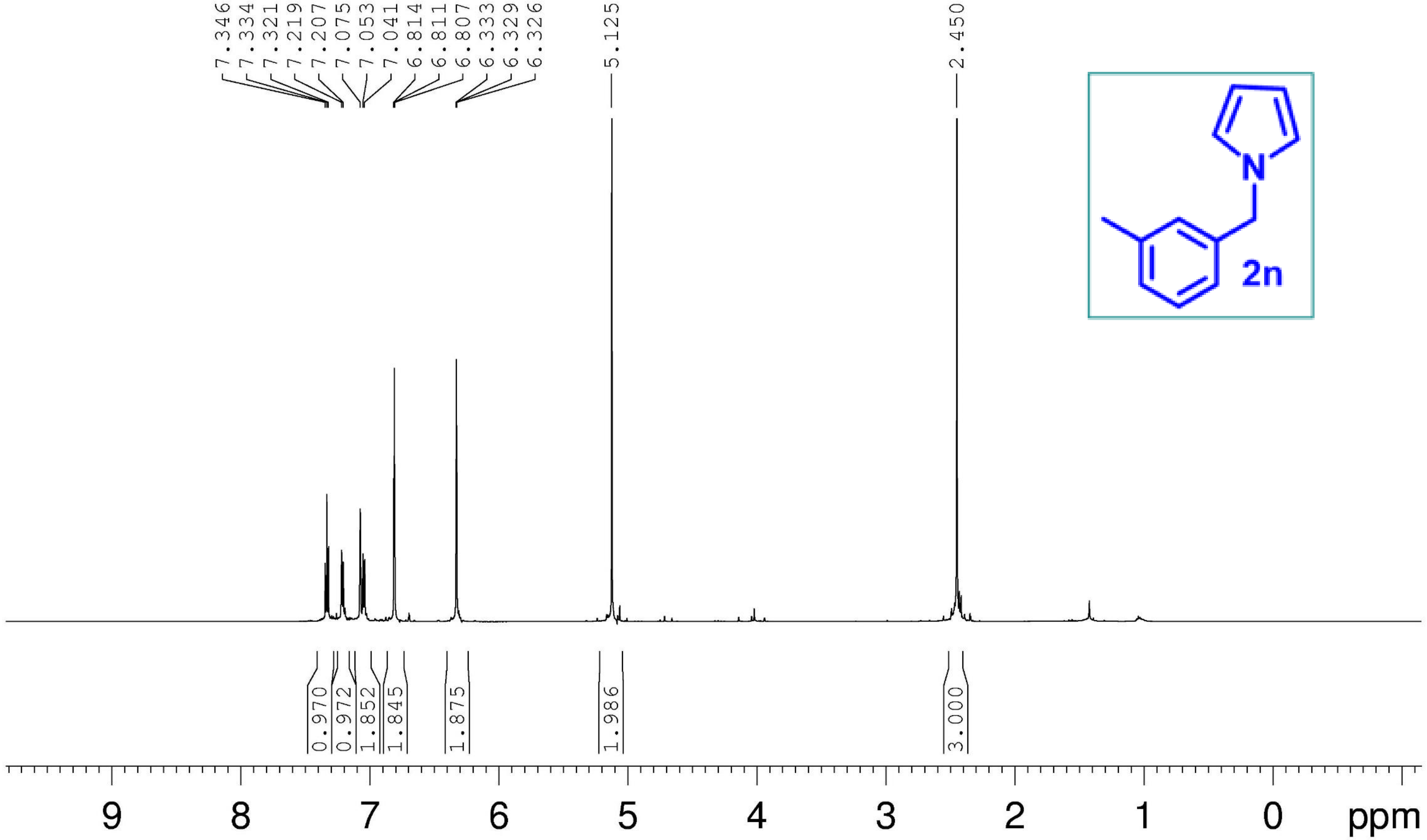

Figure S13. ^{1}H NMR spectrum (600 MHz, $CDCl_3$, 298 K) of the derivative **2n**.

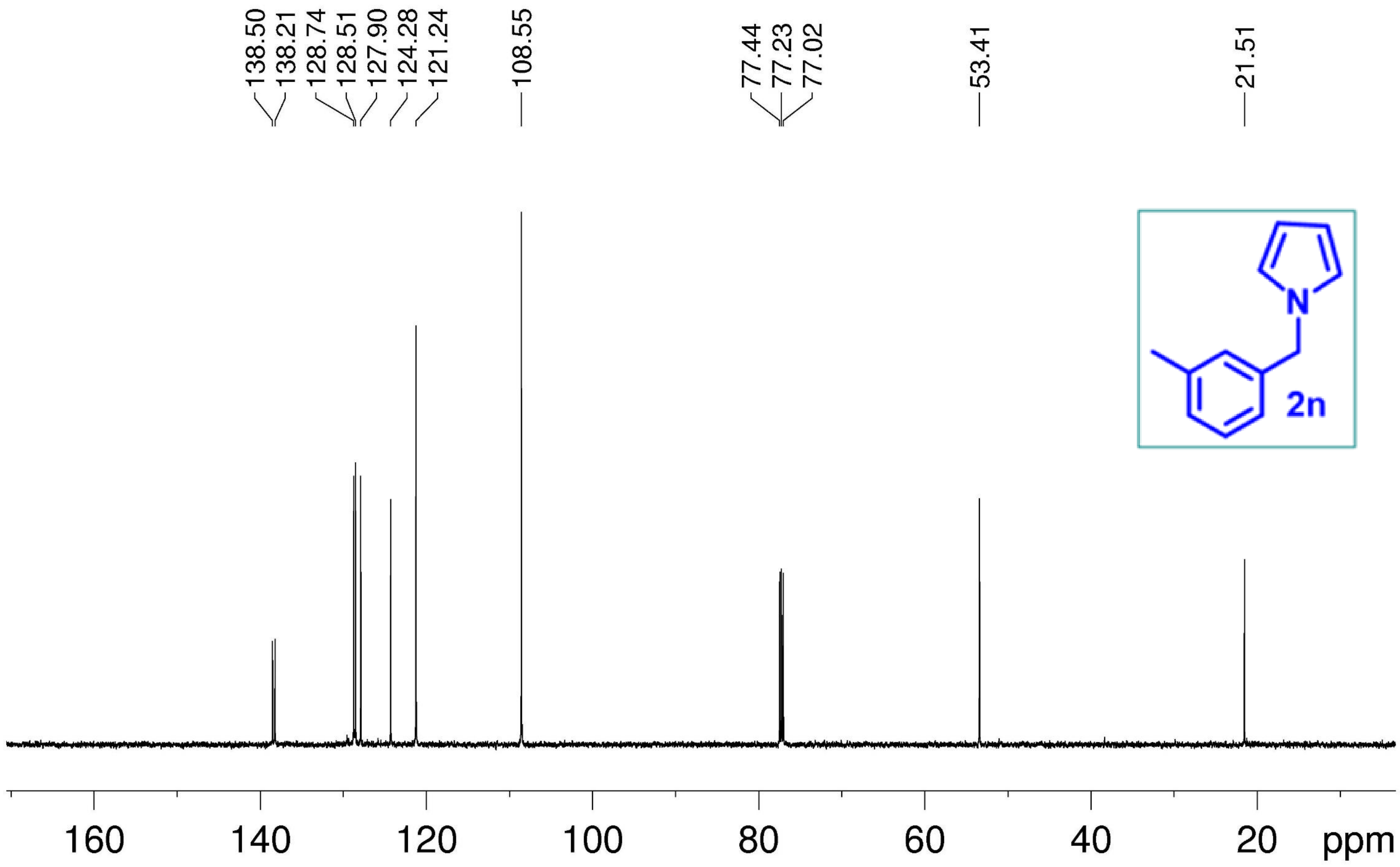

Figure S14. ^{13}C NMR spectrum (150 MHz, $CDCl_3$, 298 K) of the derivative **2n**.

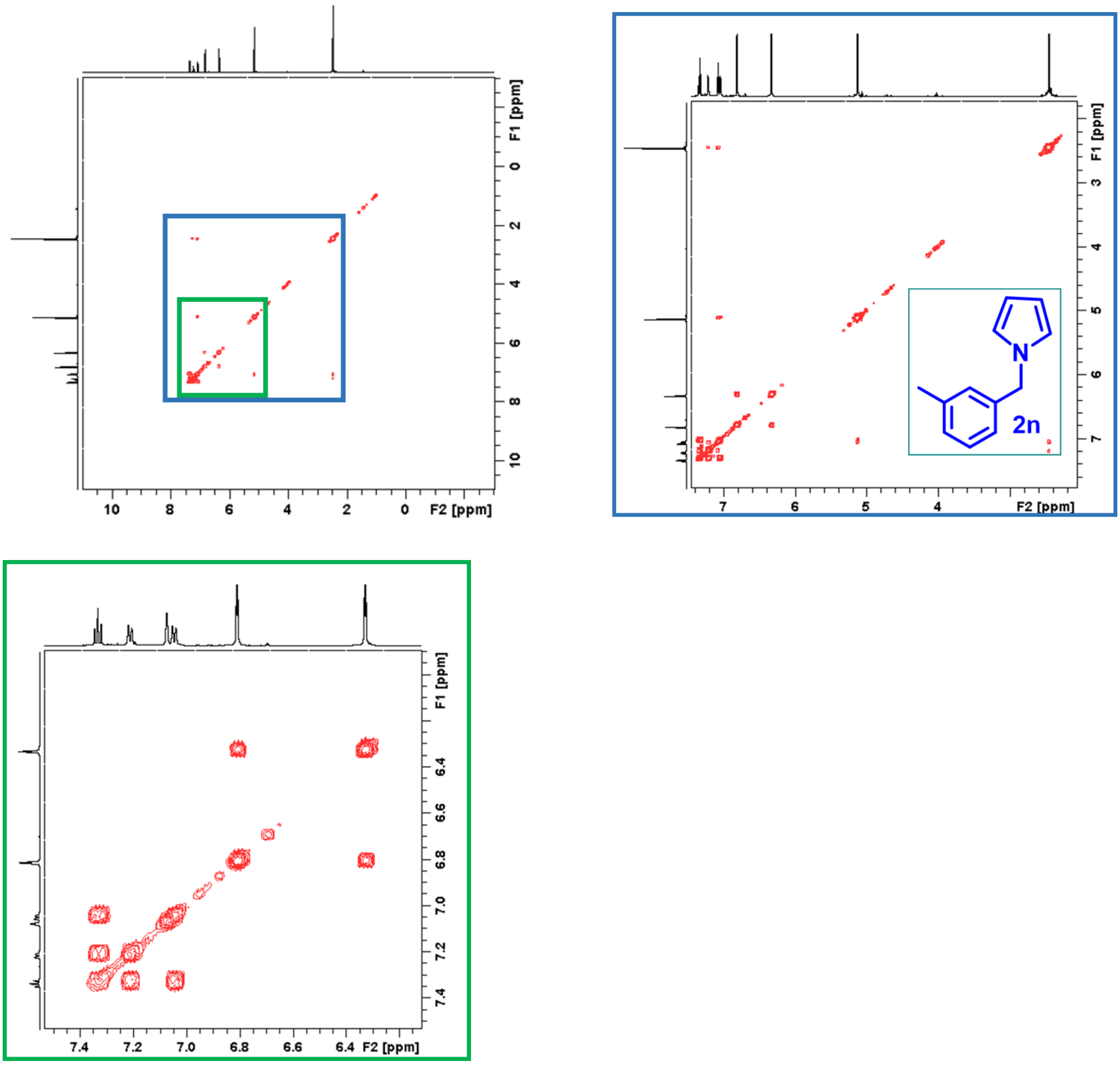

Figure S15. COSY NMR spectrum (600 MHz, $CDCl_3$, 298 K) of the derivative **2n**. Expansions of the significant parts of the spectrum in blue and green squares are visible.

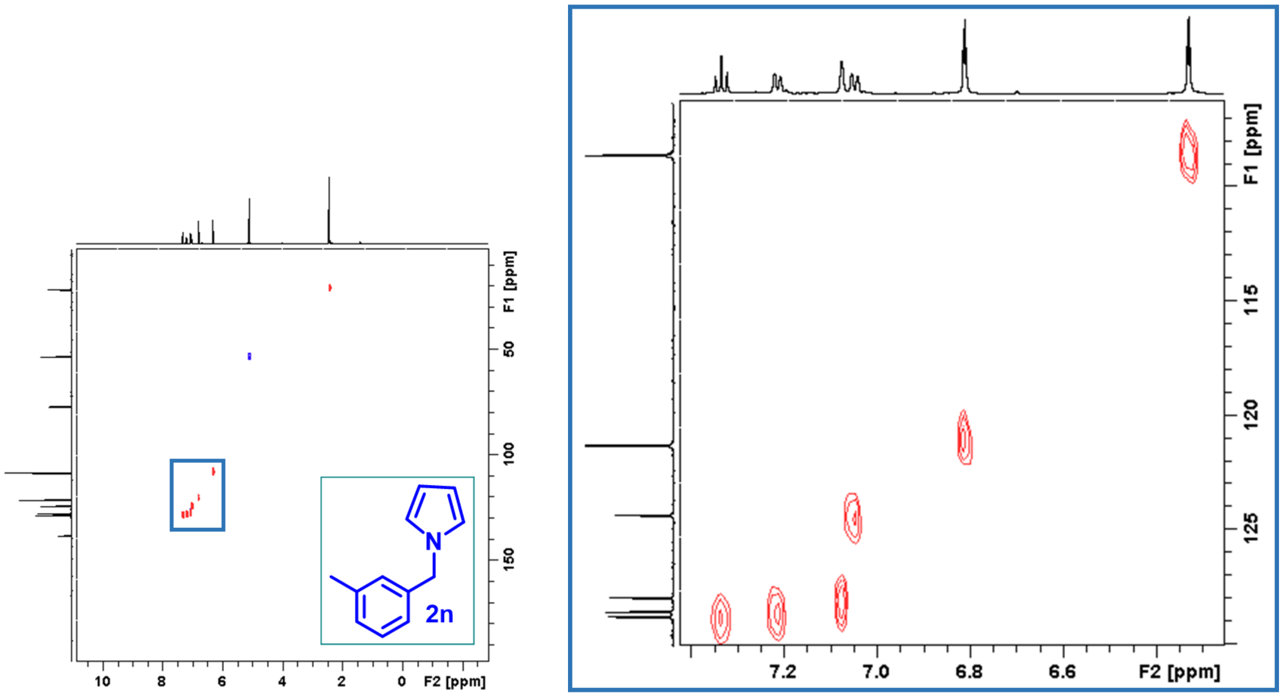

Figure S16. HSQC NMR spectrum (600 MHZ, $CDCl_3$, 298 K) of the derivative **2n**. Expansion of the significant part of the spectrum is visibile in the blue square.

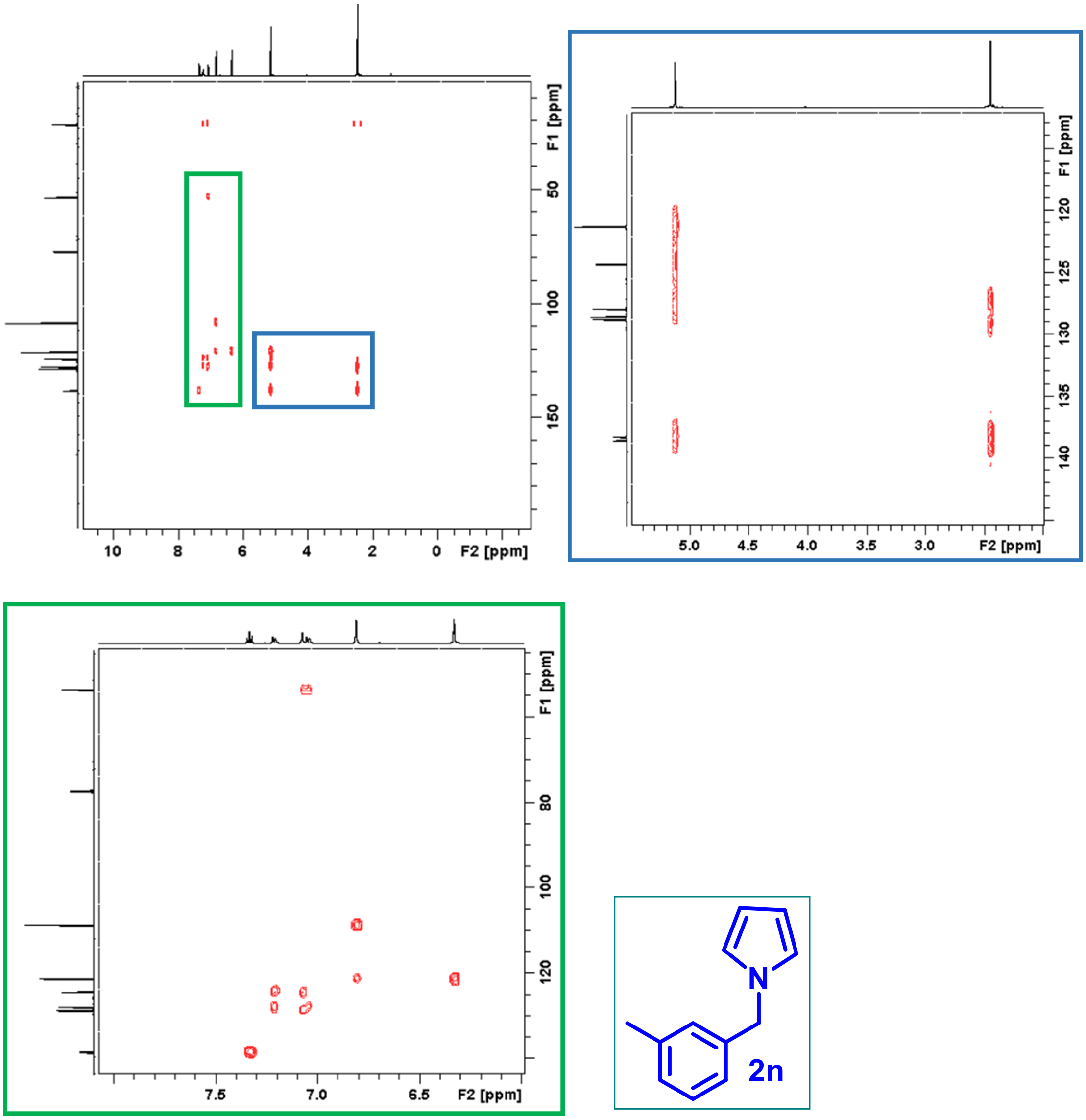

Figure S17. HMBC NMR spectrum (600 MHZ, $CDCl_3$, 298 K) of the derivative **2n**. Expansions of the significant part of the spectrum are visibile in blue and green squares.

XI. ^{1}H and ^{13}C NMR Spectra and 2D NMR Spectra of amides 4ac-ge.

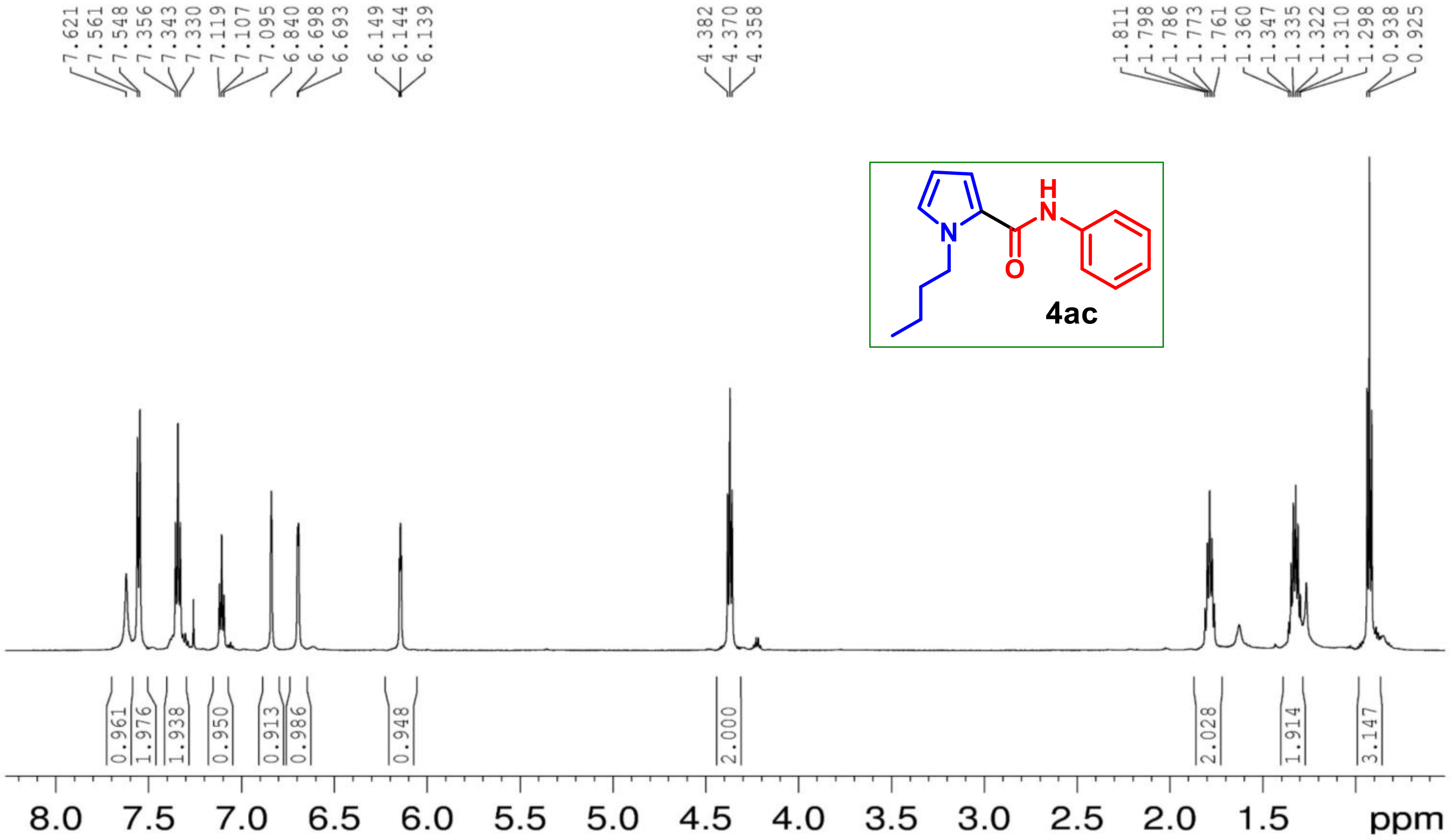

Figure S18. ^{1}H NMR spectrum (600 MHz, $CDCl_3$, 298 K) of the derivative **4ac**.

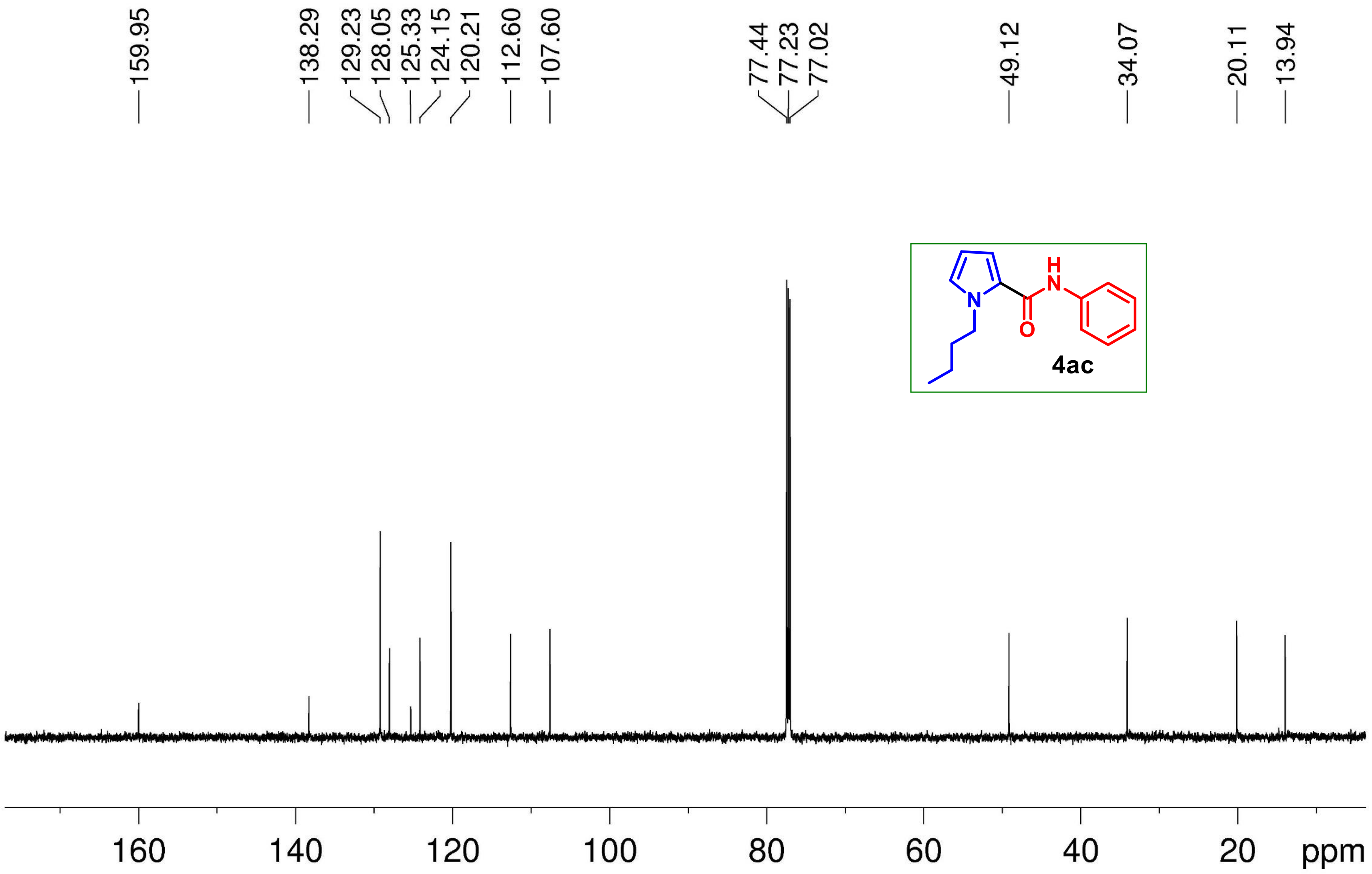

Figure S19. ^{13}C NMR spectrum (150 MHz, $CDCl_3$, 298 K) of the derivative **4ac**.

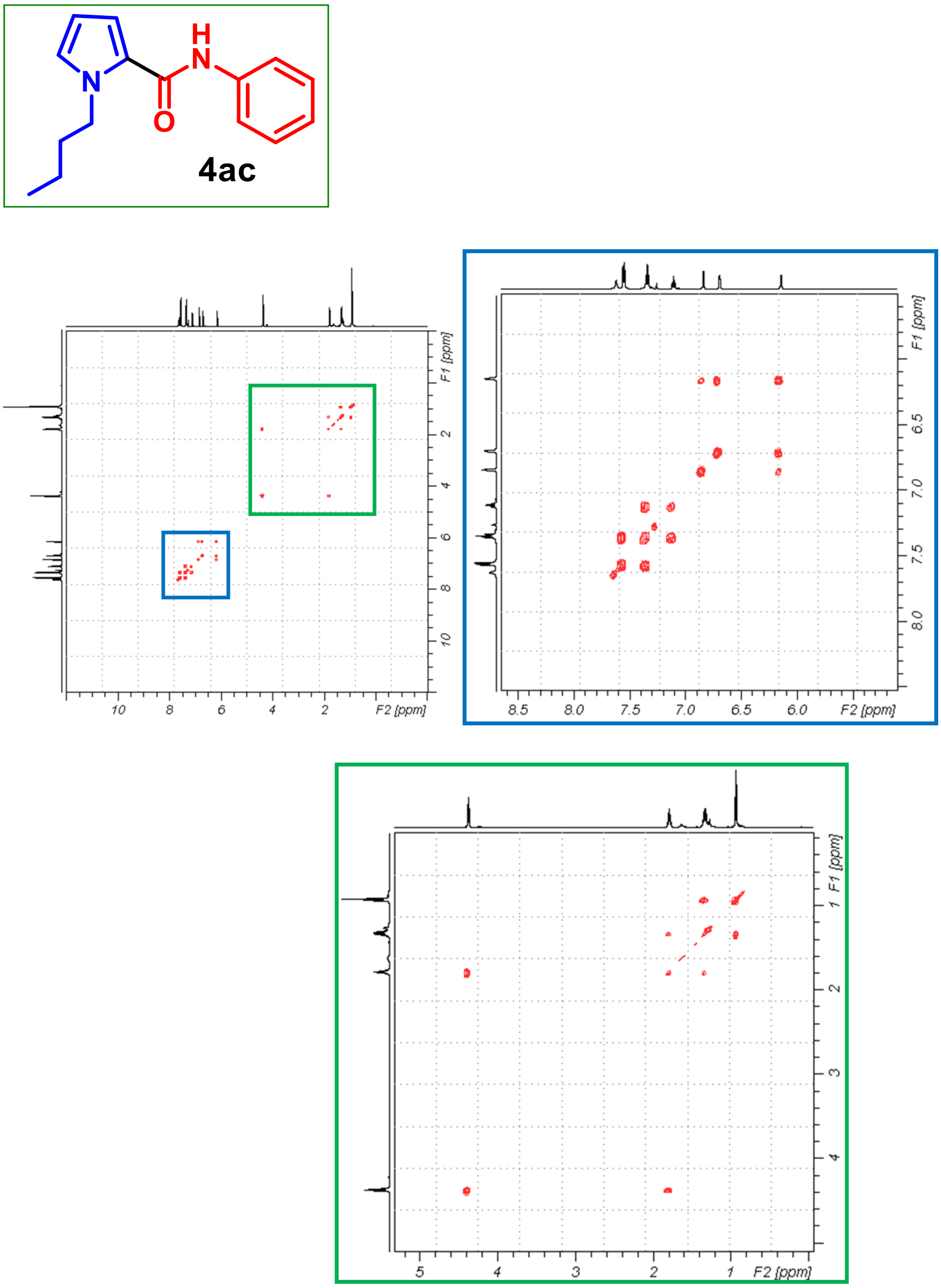

Figure S20. COSY NMR spectrum (600 MHz, $CDCl_3$, 298 K) of the derivative **4ac**, with expansions of the significant portions of the spectrum in blue and green squares.

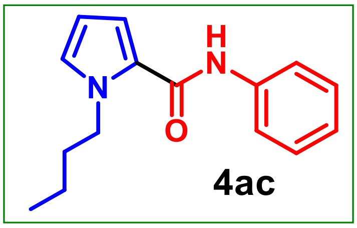

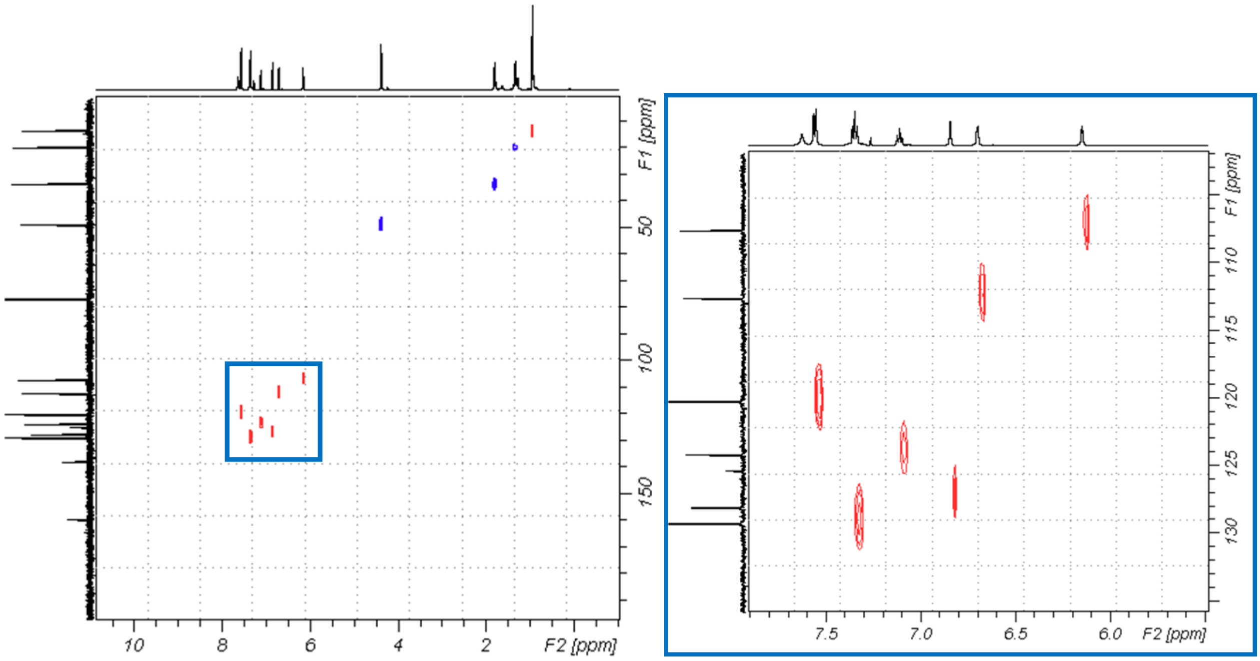

Figure S21. HSQC NMR spectrum (600 MHz, $CDCl_3$, 298K) of the derivative **4ac** with expansion of significant portion of the spectrum in blue square.

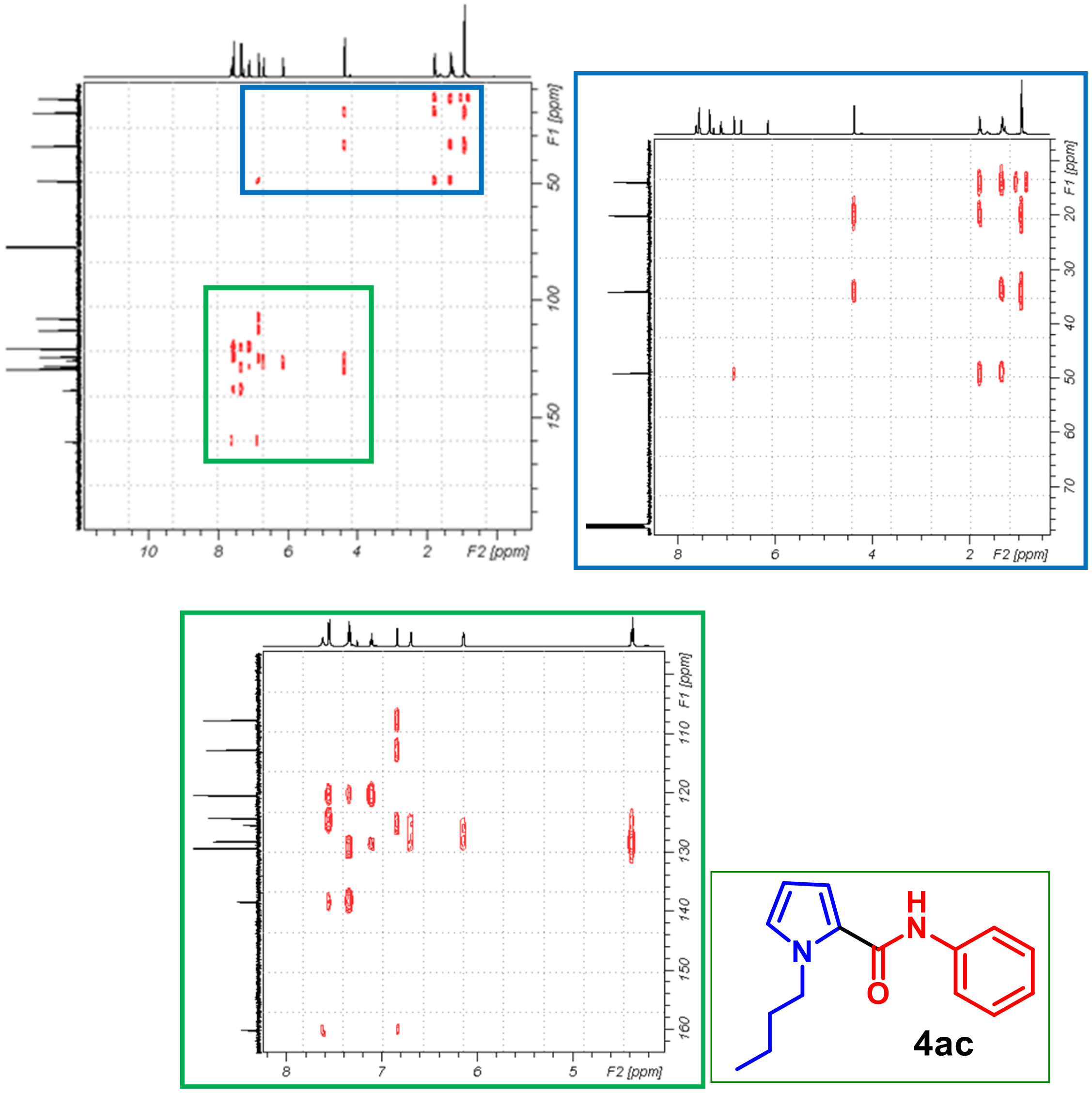

Figure S22. HMBC NMR spectrum (600 MHz, $CDCl_3$, 298K) of the derivative **4ac** with expansions of significant portions of the spectrum in blue and green squares.

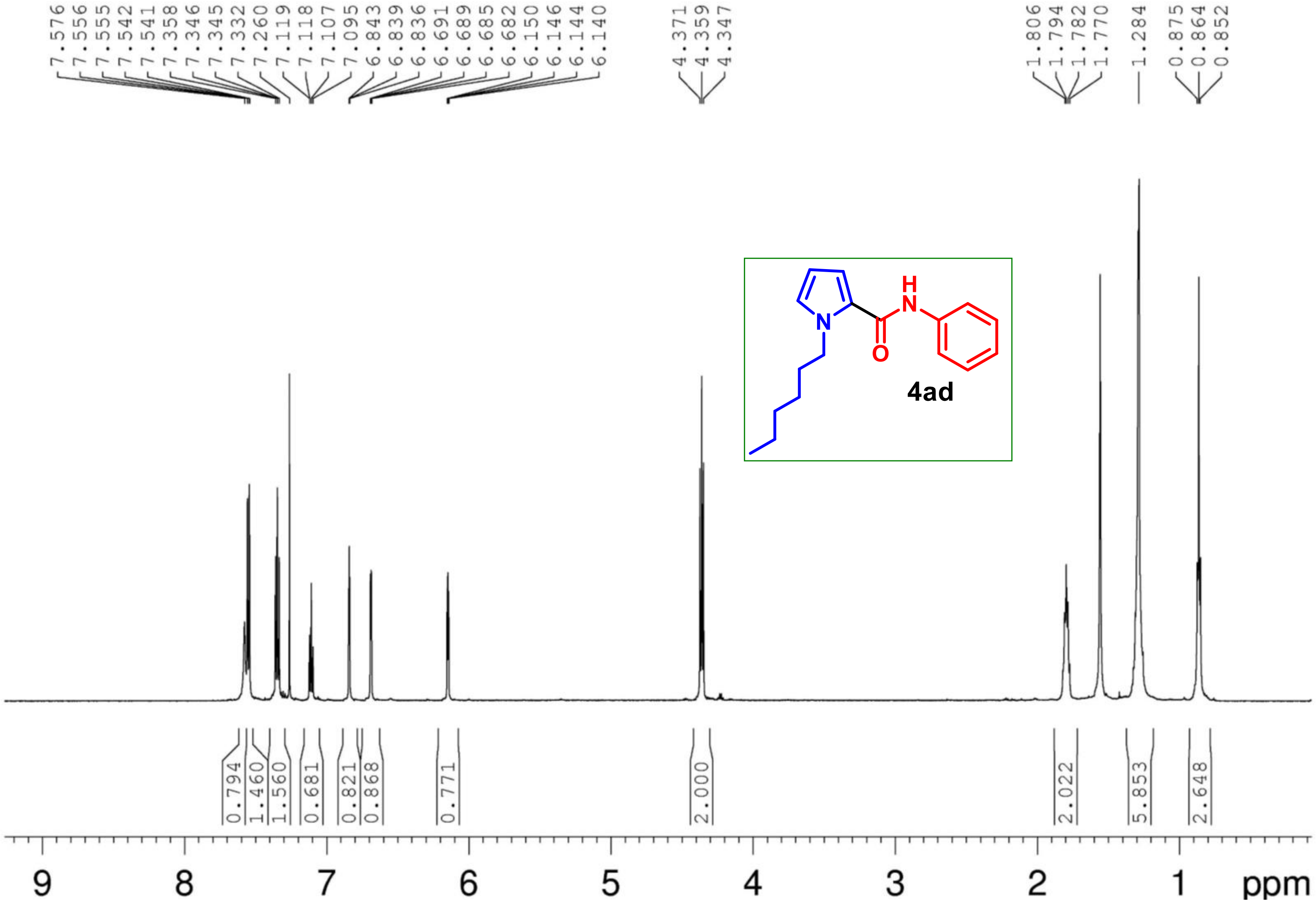

Figure S23. ^{1}H NMR spectrum (600 MHz, $CDCl_3$, 298K) of the derivative **4ad**.

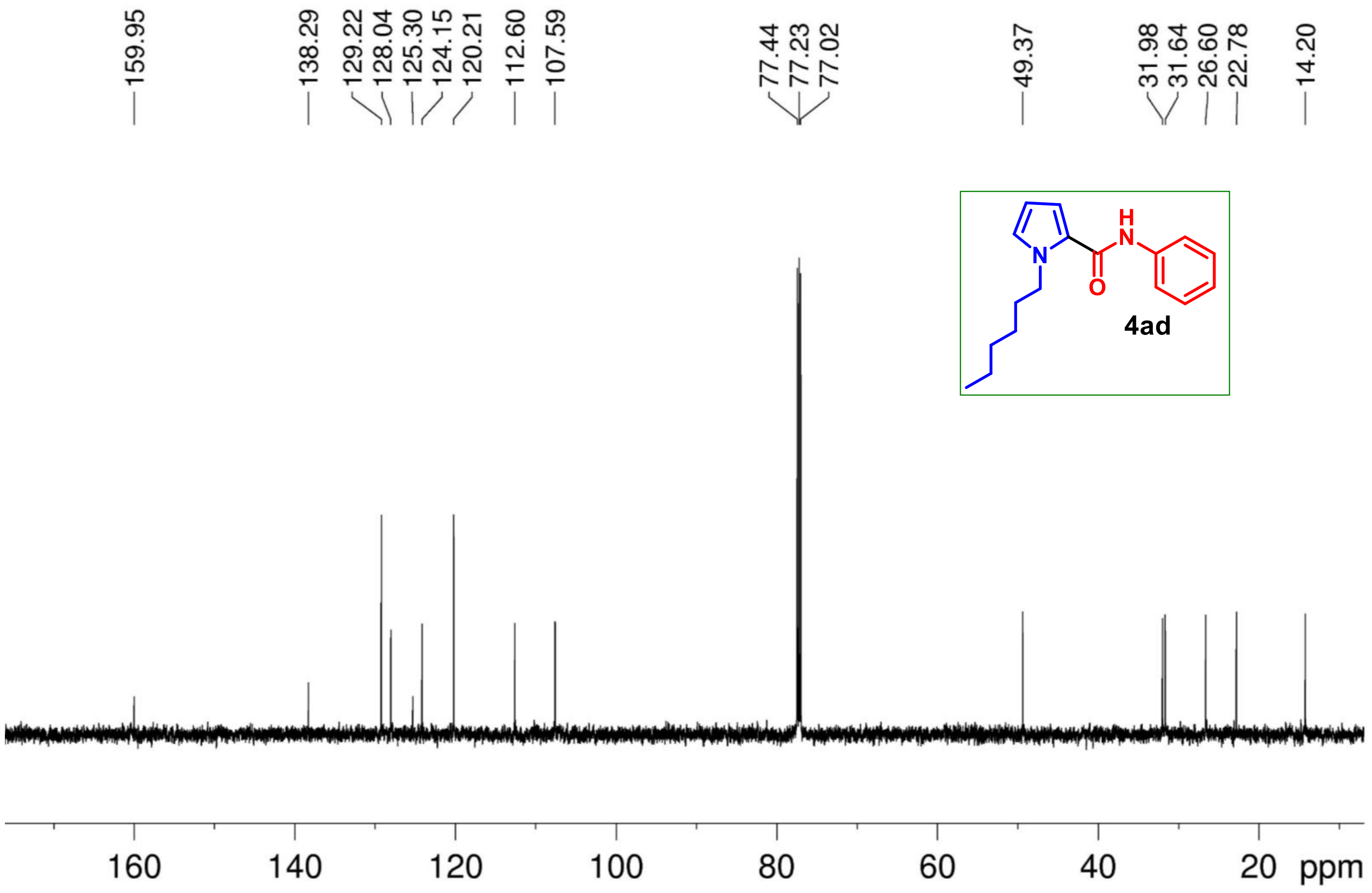

Figure S24. ^{13}C NMR spectrum (150 MHz, $CDCl_3$, 298K) of the derivative **4ad**.

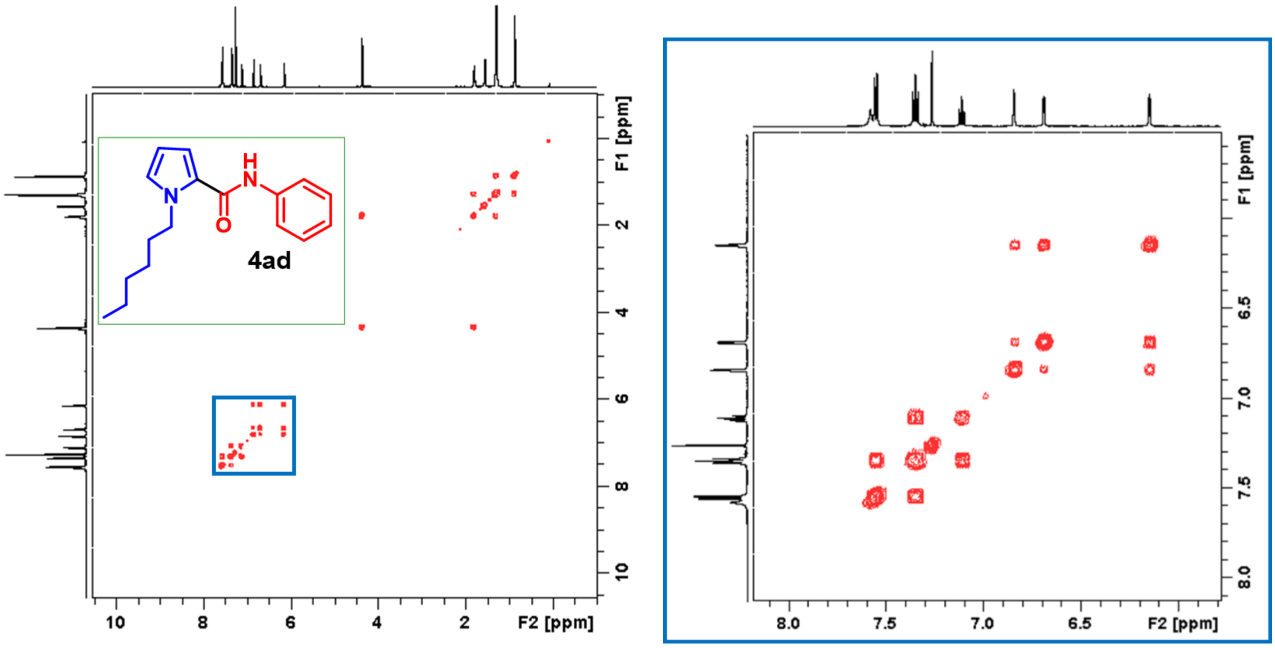

Figure S25. COSY NMR spectrum (600 MHz, $CDCl_3$, 298 K) of the derivative **4ad**, with expansion of significant portion of the spectrum in blue square.

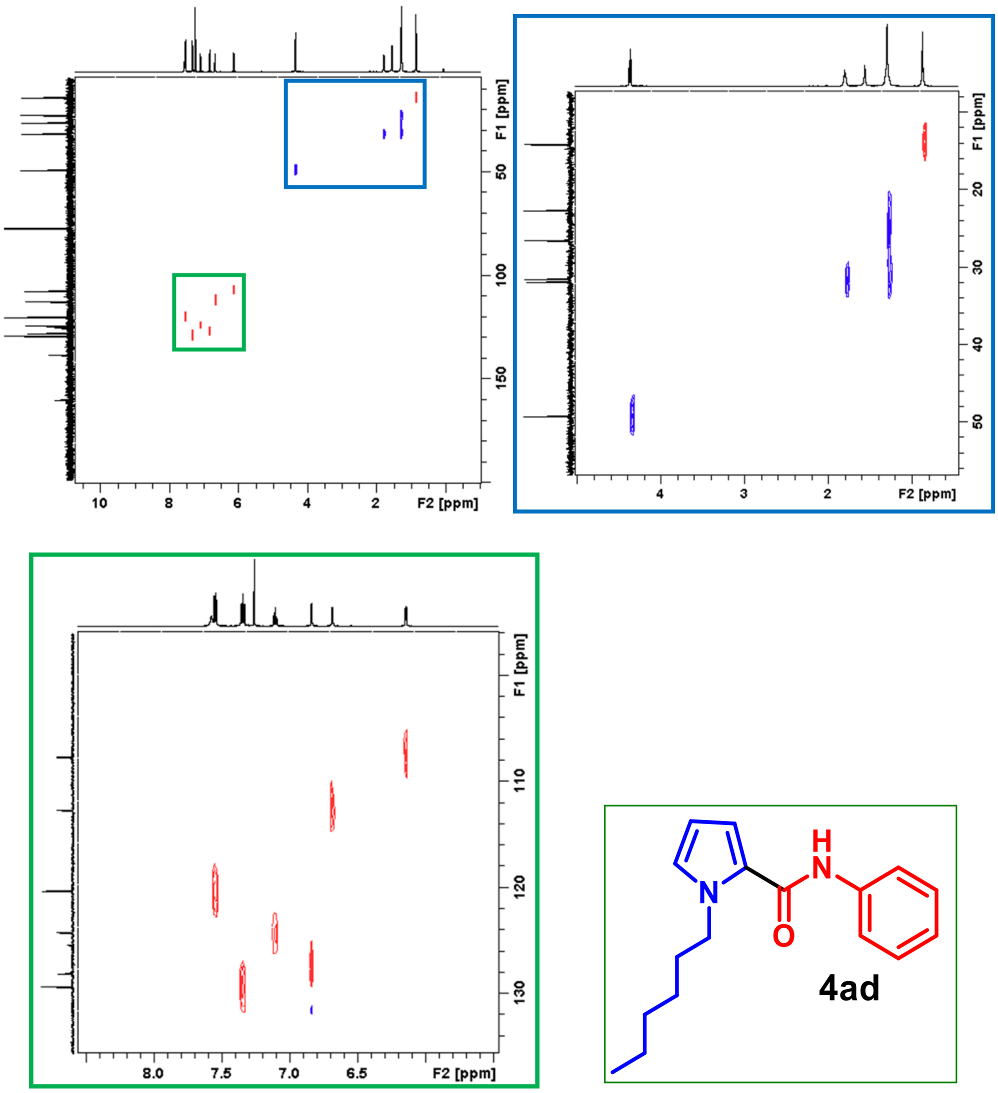

Figure S26. HSQC NMR spectrum (600 MHz, $CDCl_3$, 298 K) of the derivative **4ad**, with expansions of significant portions of the spectrum in blue and green squares.

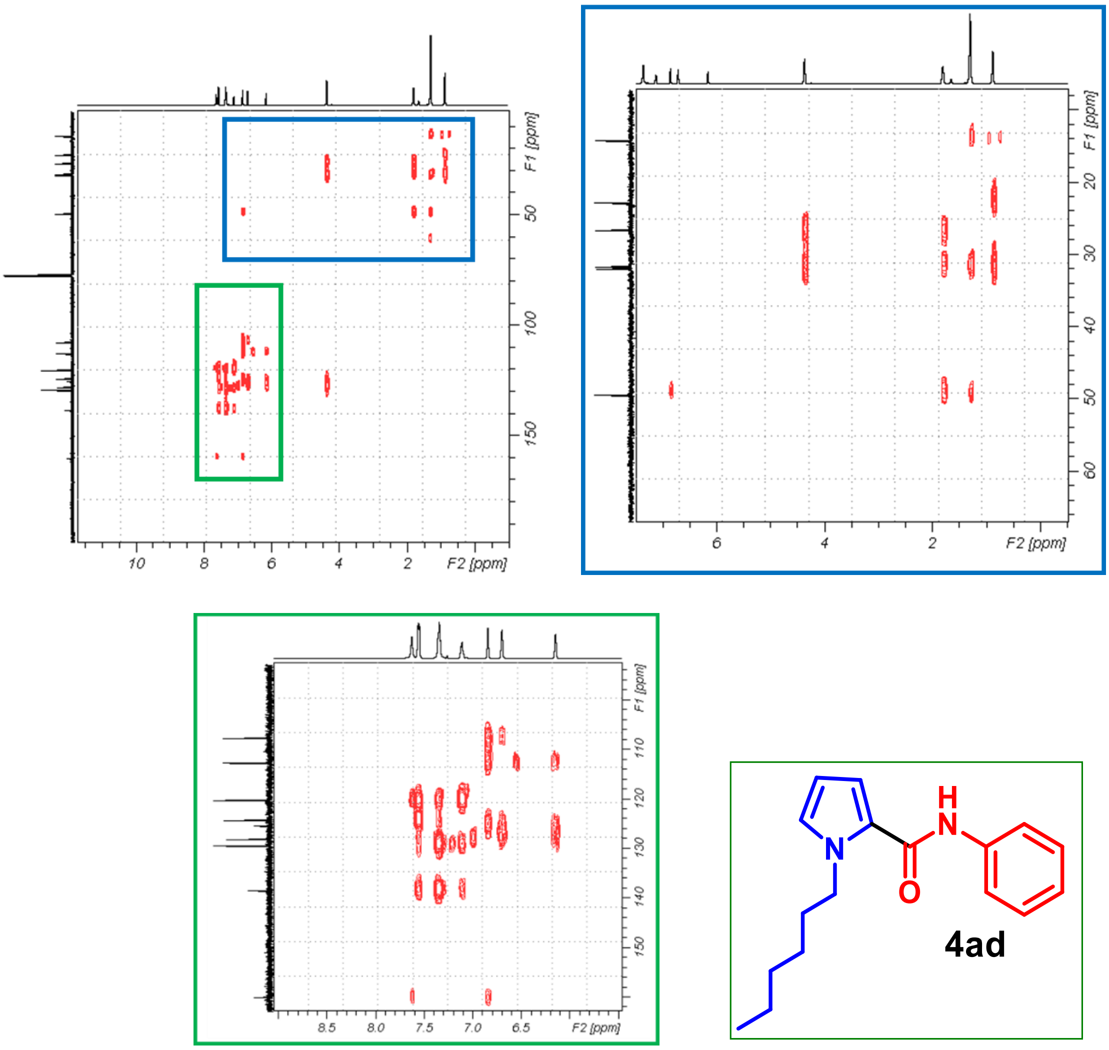

Figure S27. HMBC NMR spectrum (600 MHz, $CDCl_3$, 298 K) of the derivative **4ad**, with expansions of significant portions of the spectrum in blue and green squares.

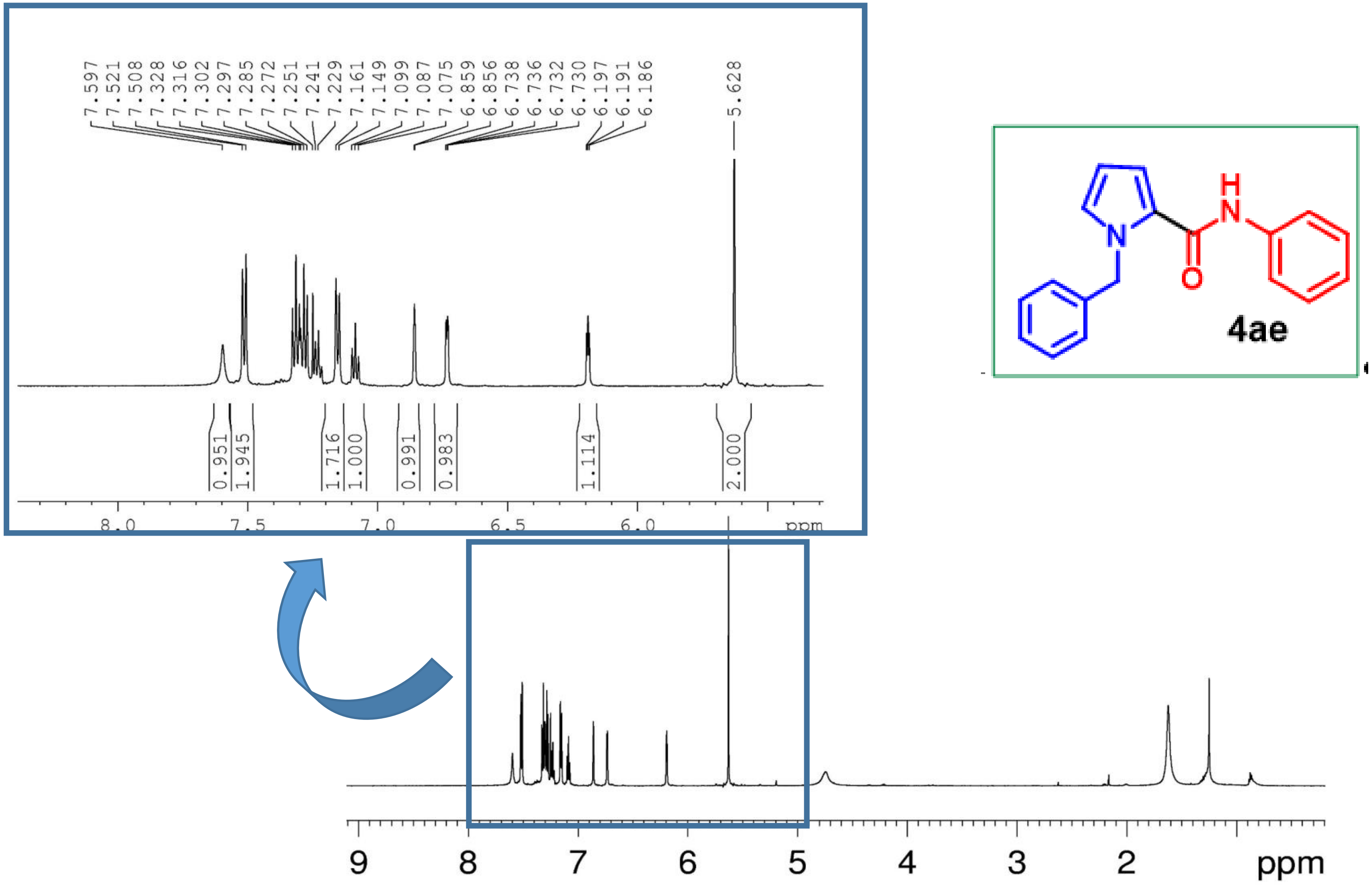

Figure S28. ^{1}H NMR spectrum (600 MHz, $CDCl_3$, 298 K) of the derivative **4ae**, with expansion of the aromatic portion of the spectrum in blue square.

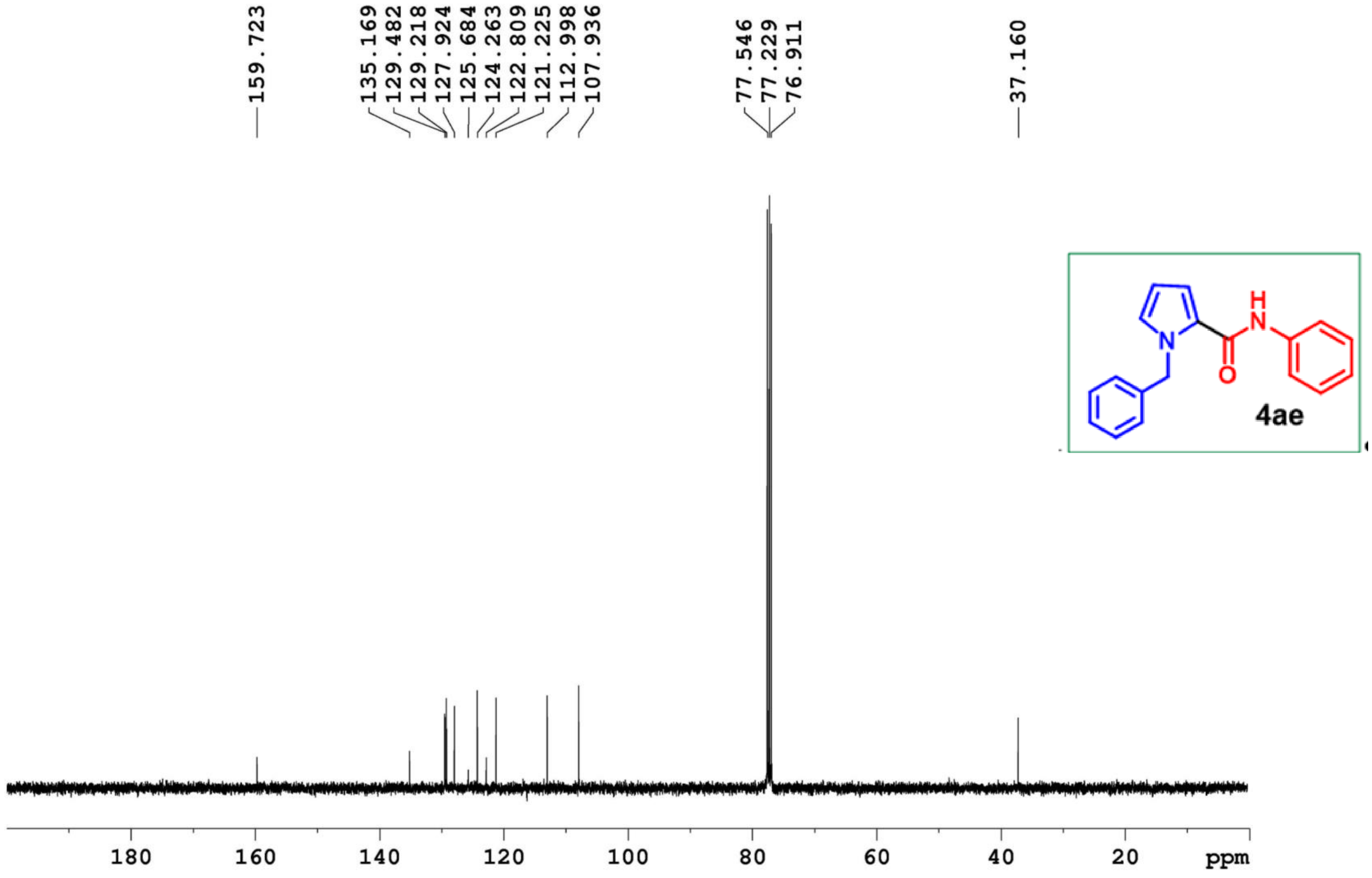

Figure S29. ^{13}C NMR spectrum (150 MHz, $CDCl_3$, 298 K) of the derivative **4ae**.

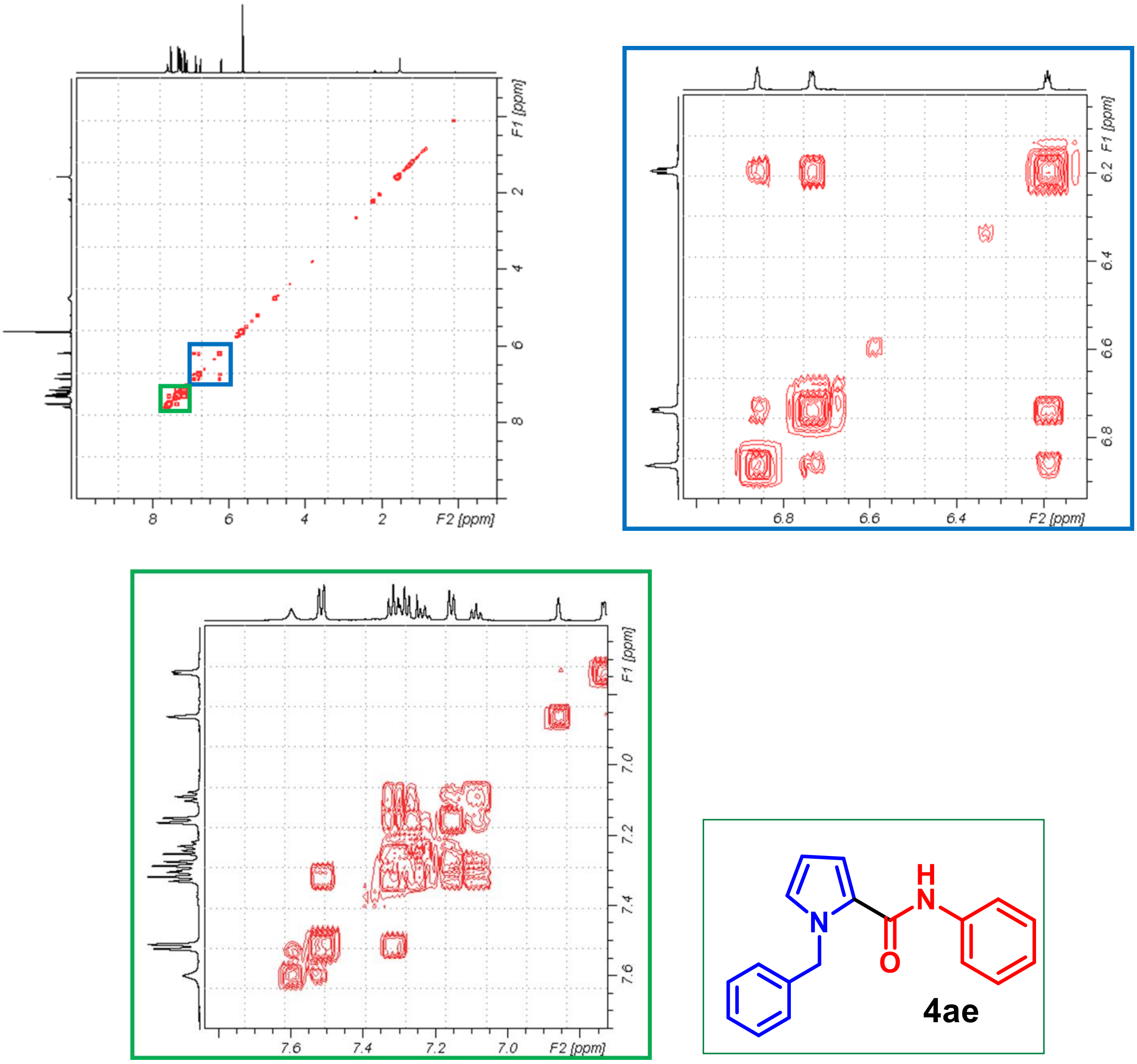

Figure S30. COSY NMR spectrum (600 MHz, $CDCl_3$, 298 K) of the derivative **4ae**, with expansions of significant portions of the spectrum in blue and green squares.

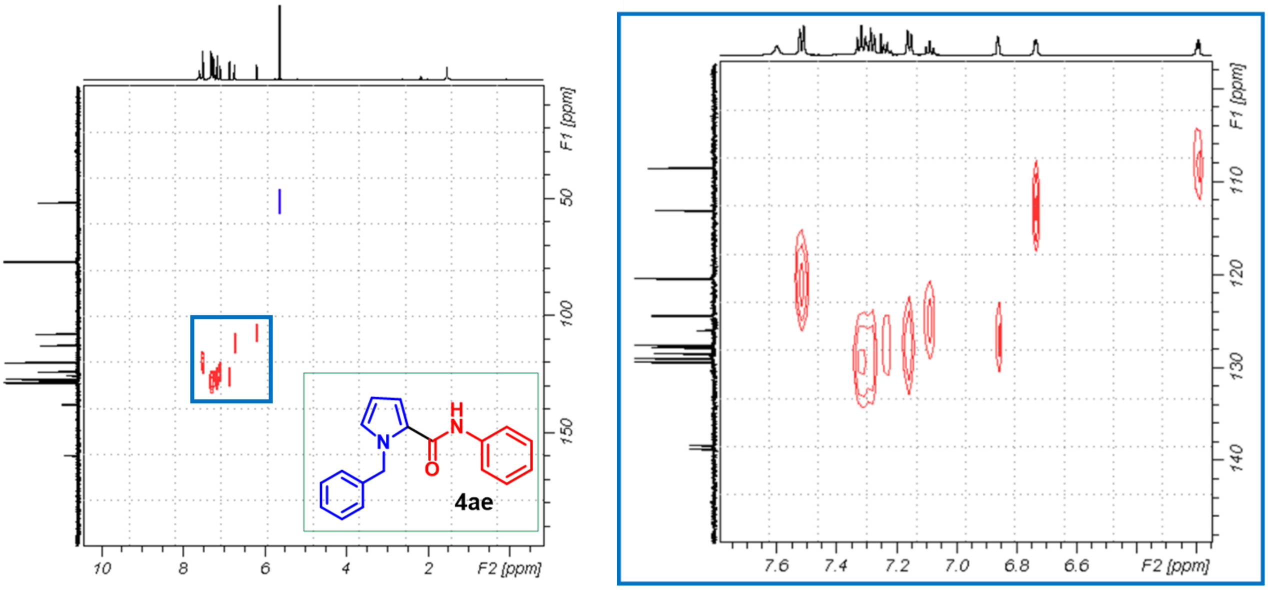

Figure S31. HSQC NMR spectrum (600 MHz, $CDCl_3$, 298 K) of the derivative **4ae**, with expansion of significant portion of the spectrum in blue square.

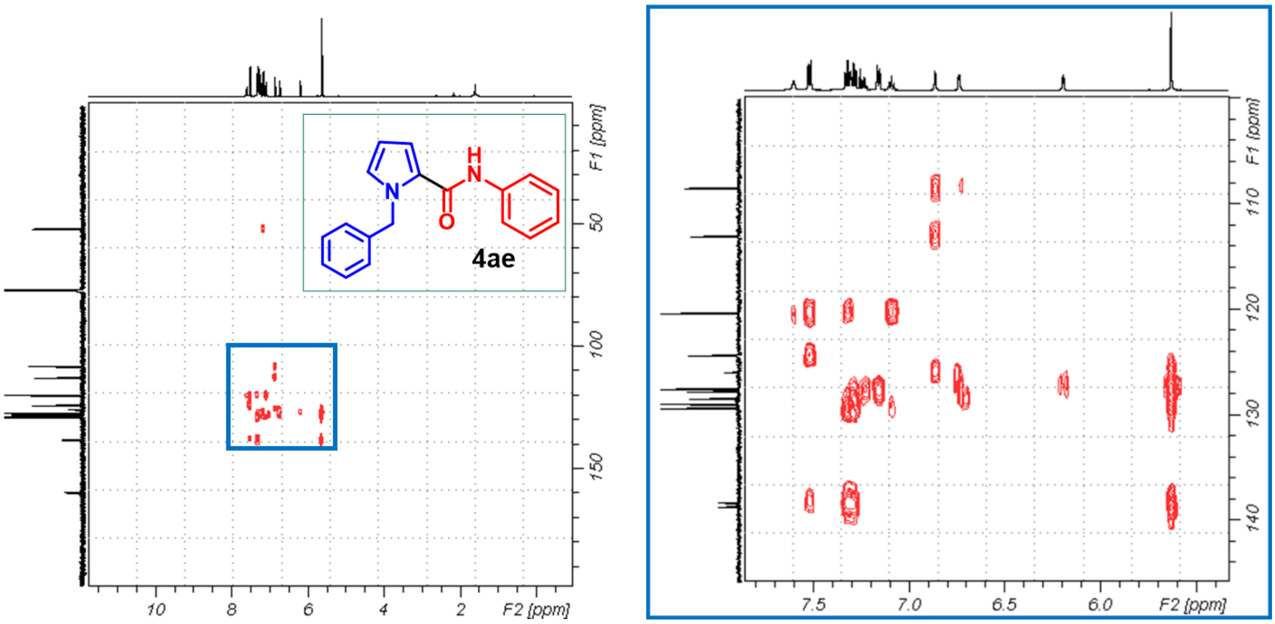

Figure S32. HMBC NMR spectrum (600 MHz, $CDCl_3$, 298 K) of the derivative **4ae**, with expansion of significant portion of the spectrum in blue square.

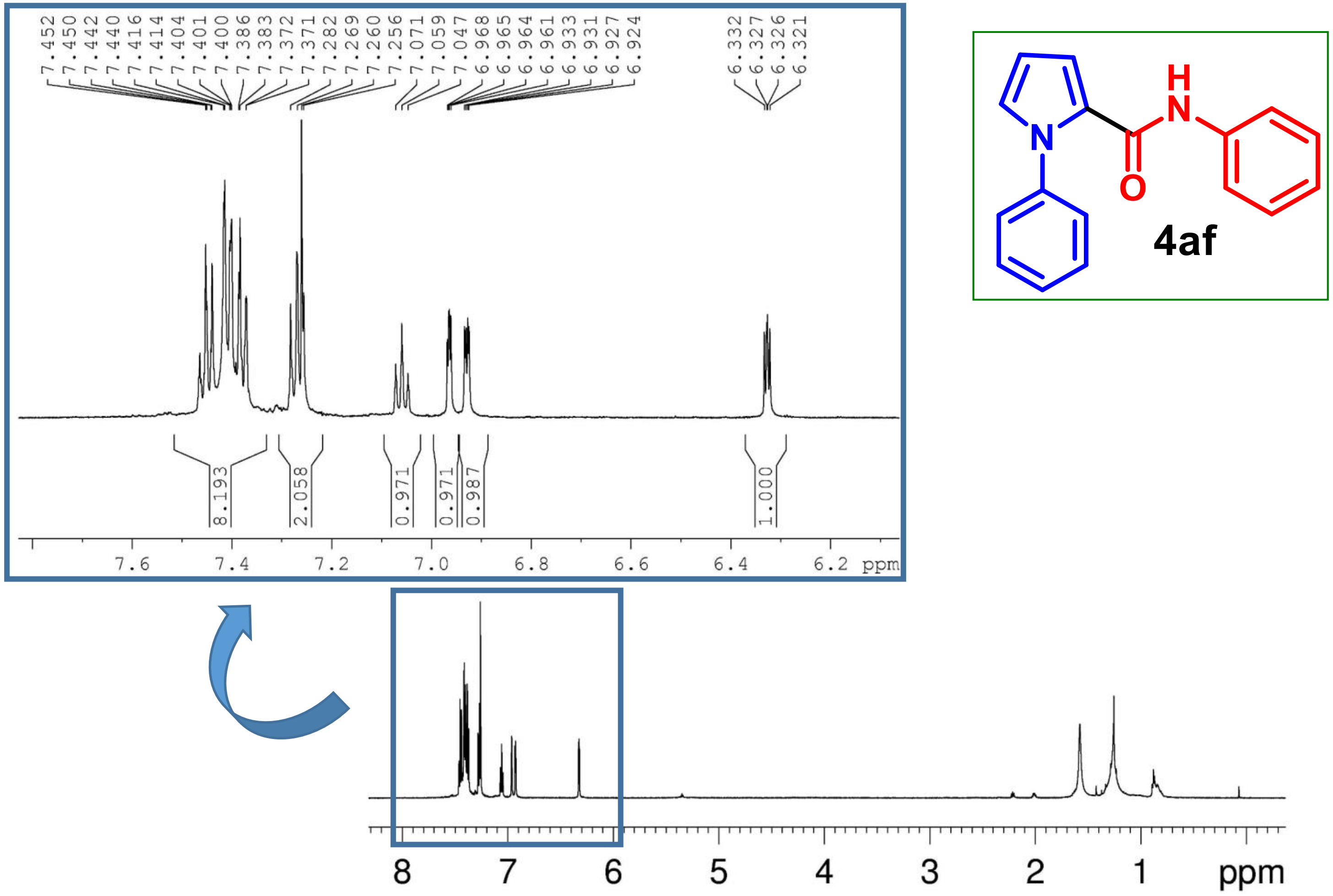

Figure S33. ^{1}H NMR spectrum (600 MHz, $CDCl_3$, 298 K) of the derivative **4af**, with expansion of significant portion of the spectrum in blue square.

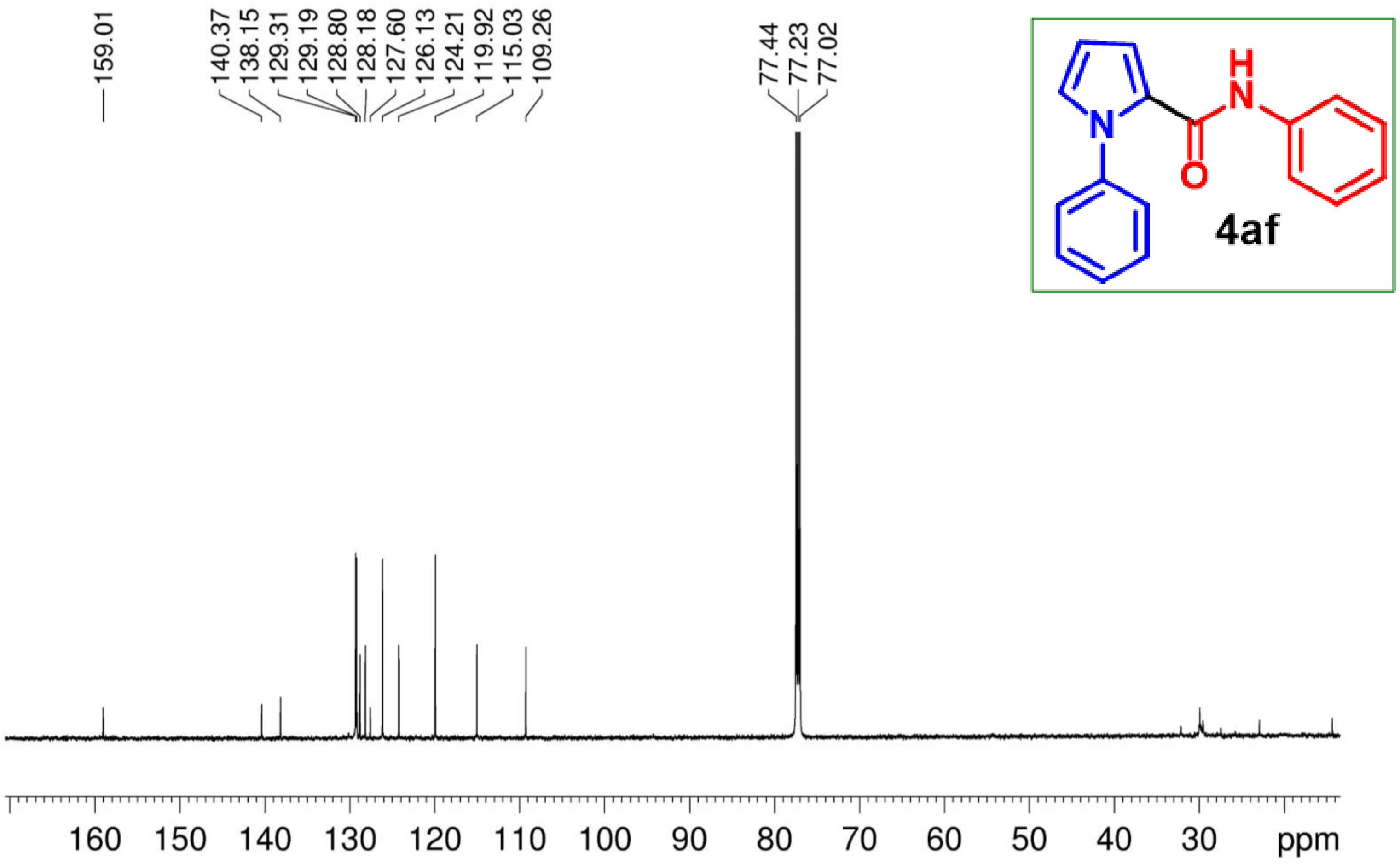

Figure S34. ^{13}C NMR spectrum (150 MHz, $CDCl_3$, 298 K) of the derivative **4af**.

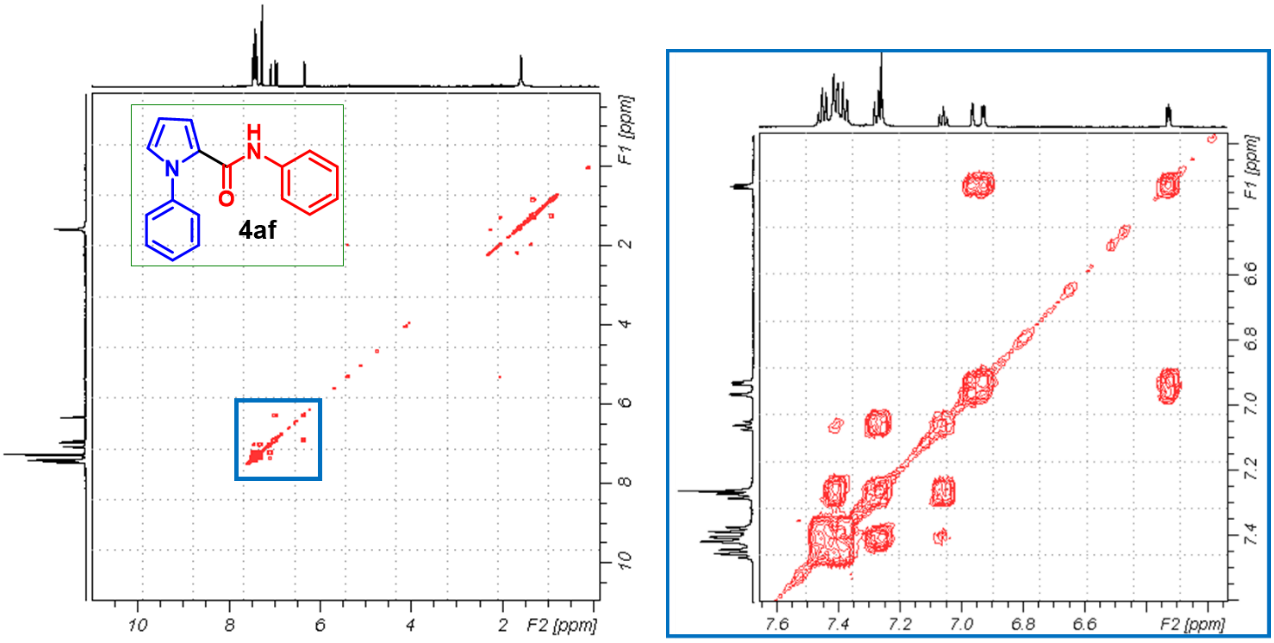

Figure S35. COSY NMR spectrum (600 MHz, $CDCl_3$, 298 K) of the derivative **4af**, with expansion of significant portion of the spectrum in blue square.

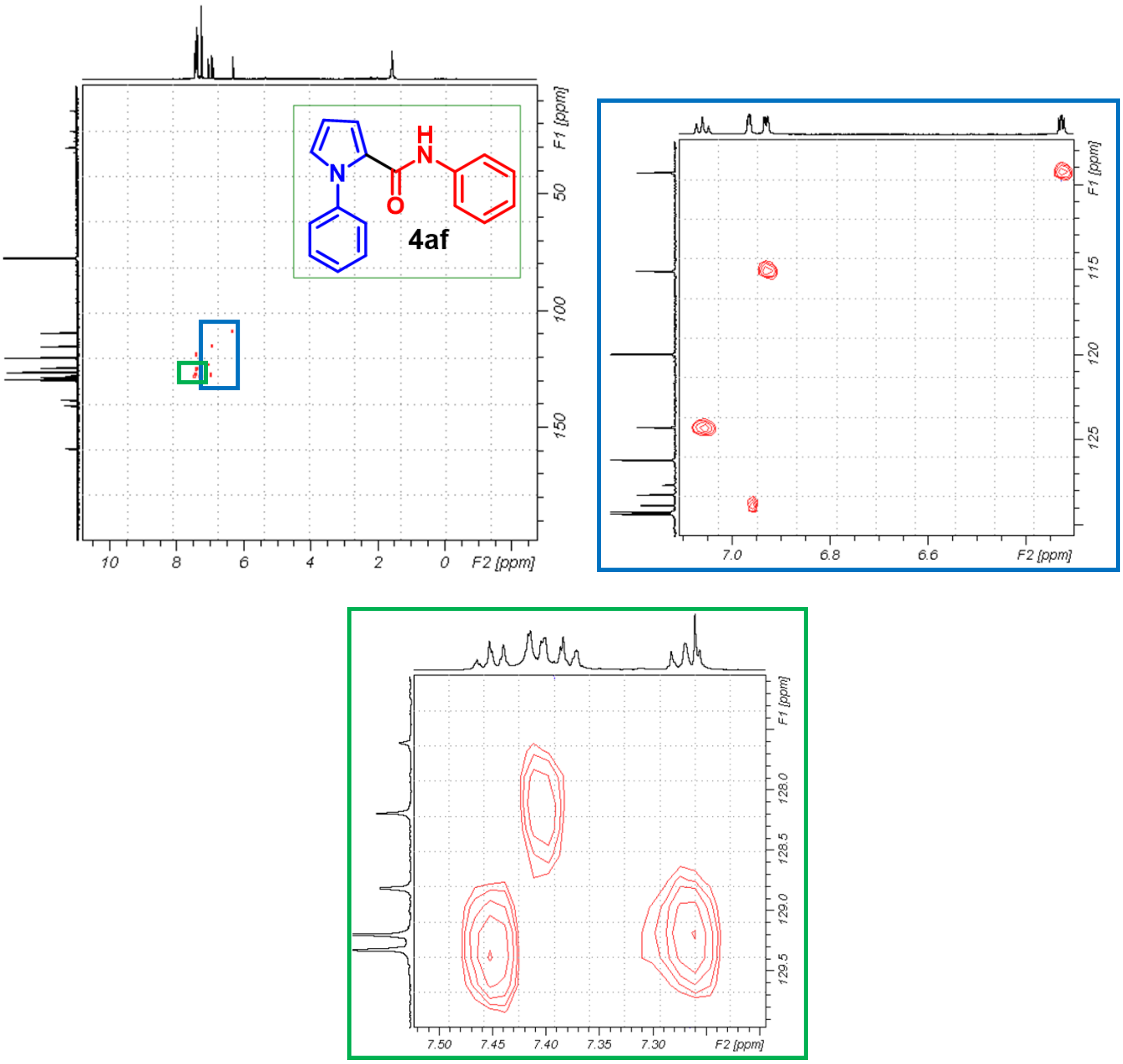

Figure S36. HSQC NMR spectrum (600 MHz, $CDCl_3$, 298 K) of the derivative **4af**, with expansions of significant portions of the spectrum in blue and green squares.

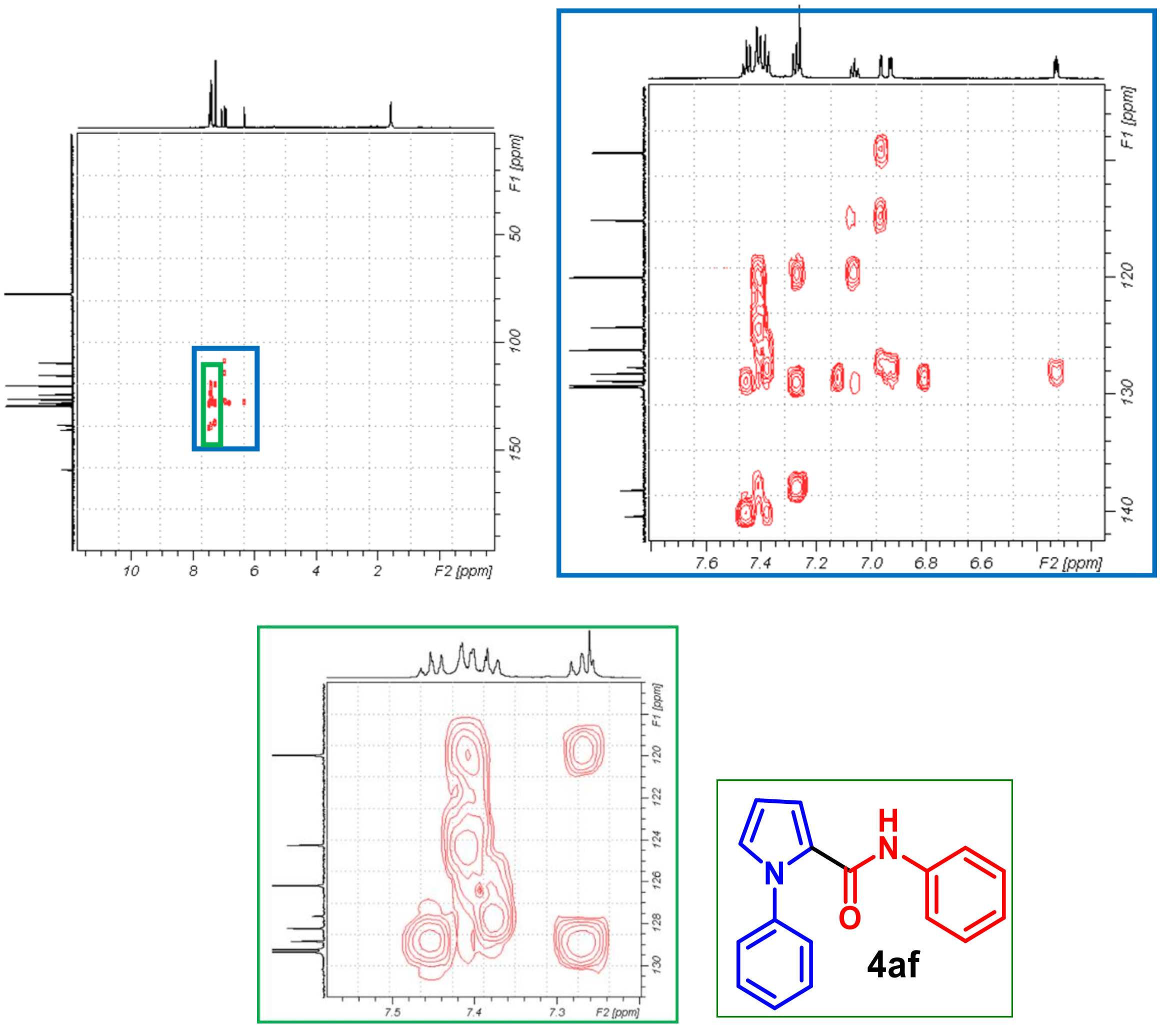

Figure S37. HMBC NMR spectrum (600 MHz, $CDCl_3$, 298 K) of the derivative **4af**, with expansions of significant portions of the spectrum in blue and green squares.

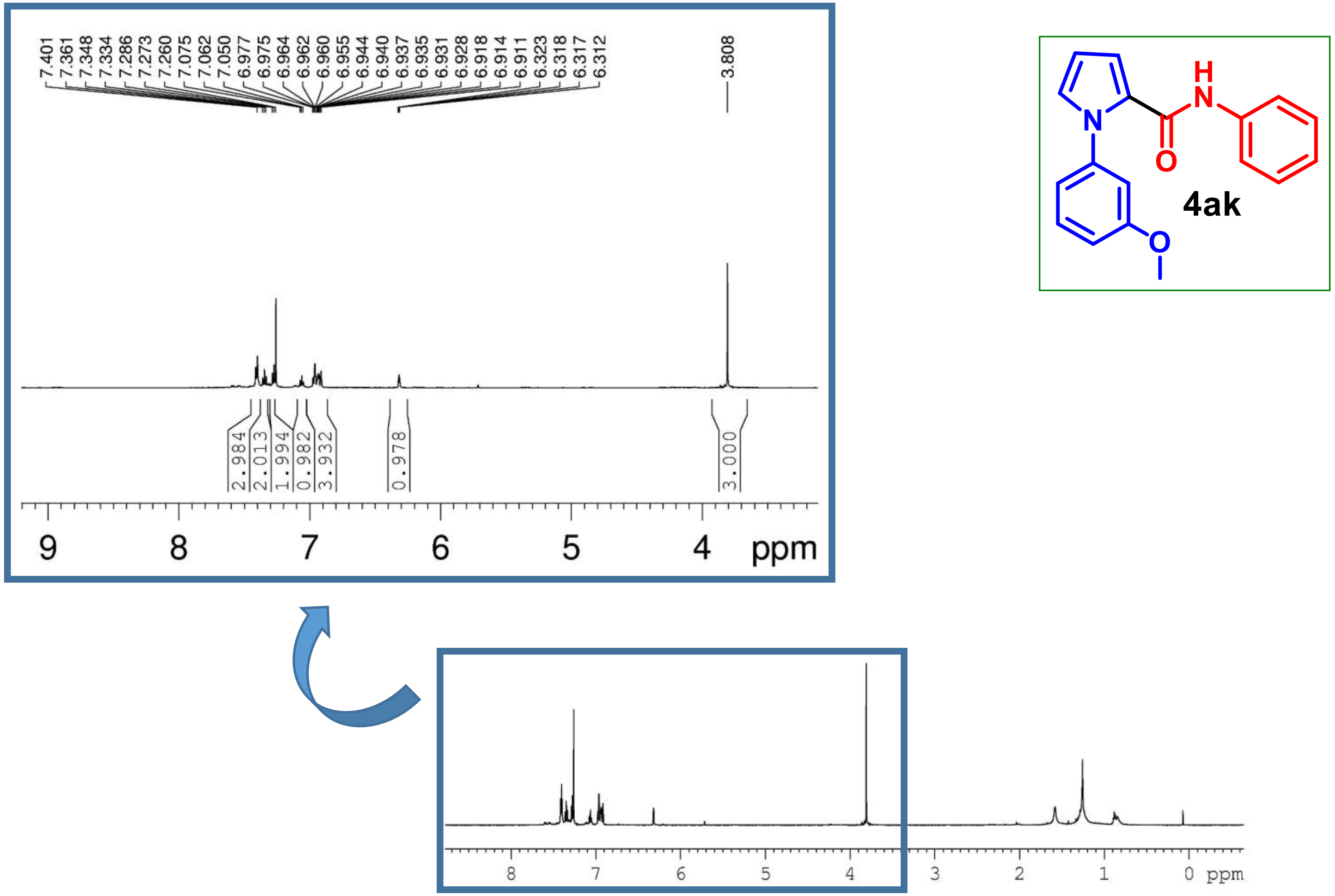

Figure S38. ^{1}H NMR spectrum (600 MHz, $CDCl_3$, 298 K) of the derivative **4ak**, with expansion of significant portion of the spectrum in blue square.

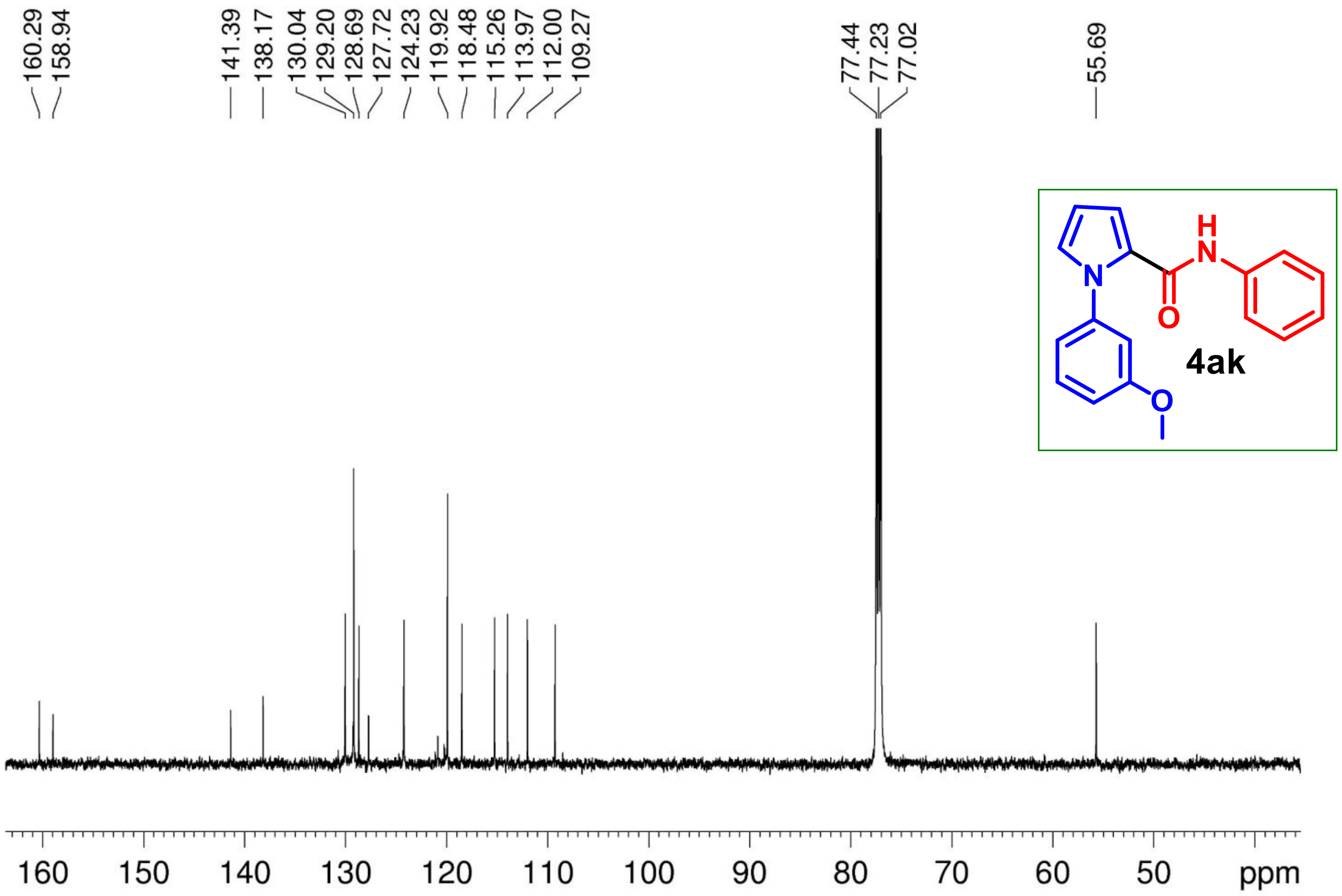

Figure S39. ^{13}C NMR spectrum (150 MHz, $CDCl_3$, 298 K) of the derivative **4ak**.

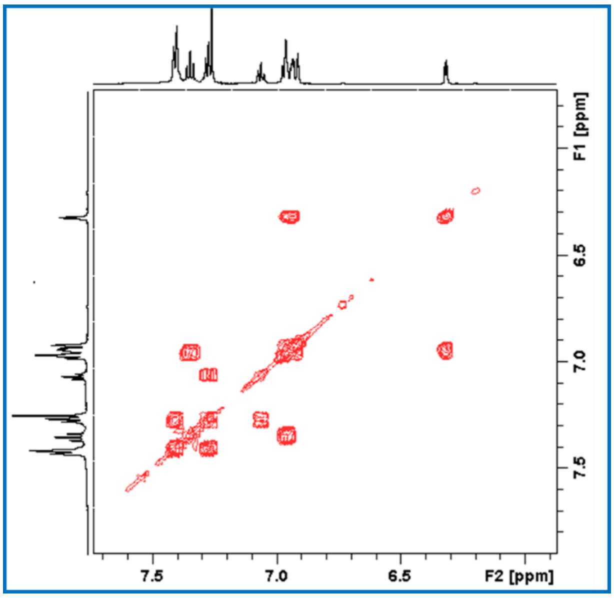

Figure S40. COSY NMR spectrum (600 MHz, $CDCl_3$, 298 K) of the derivative **4ak**, with expansion of significant portion of the spectrum in blue square.

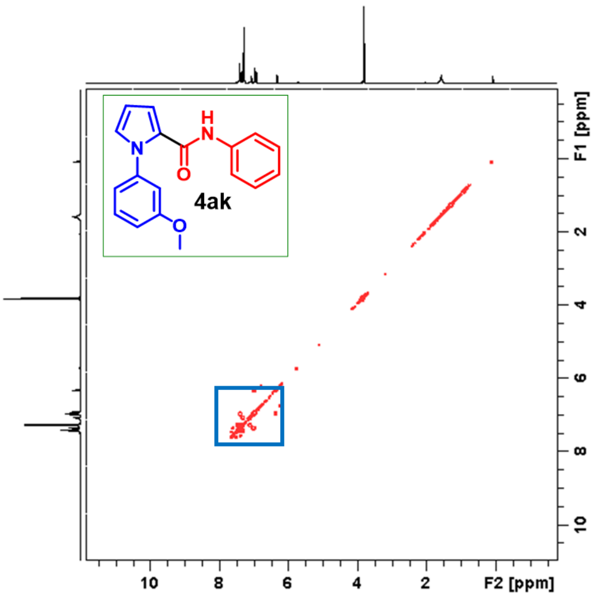

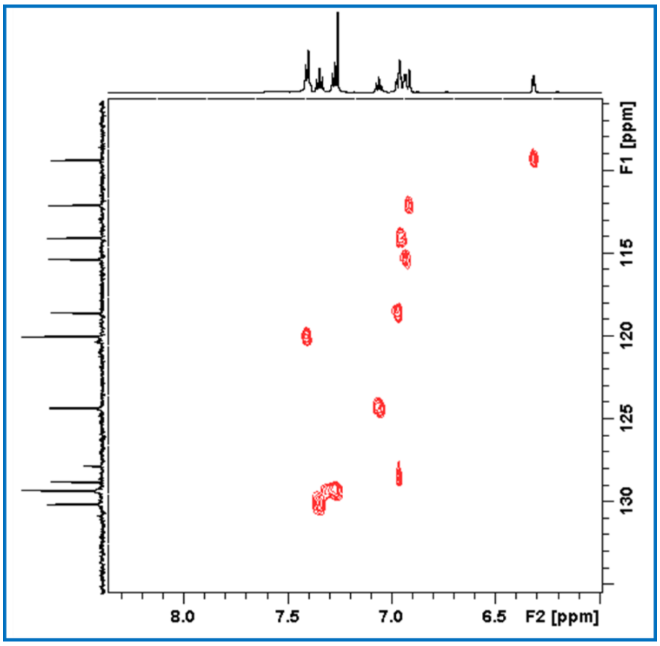

Figure S41. HSQC NMR spectrum (600 MHz, $CDCl_3$, 298 K) of the derivative **4ak**, with expansion of significant portion of the spectrum in blue square.

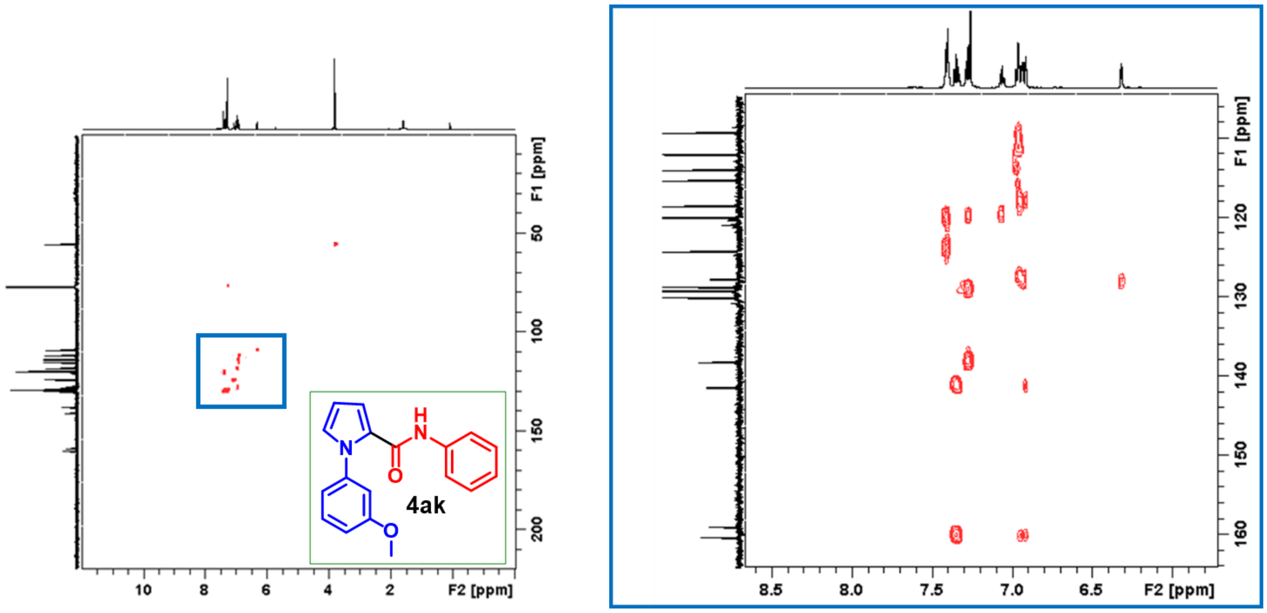

Figure S42. HMBC NMR spectrum (600 MHz, $CDCl_3$, 298 K) of the derivative **4ak**, with expansion of significant portion of the spectrum in blue square.

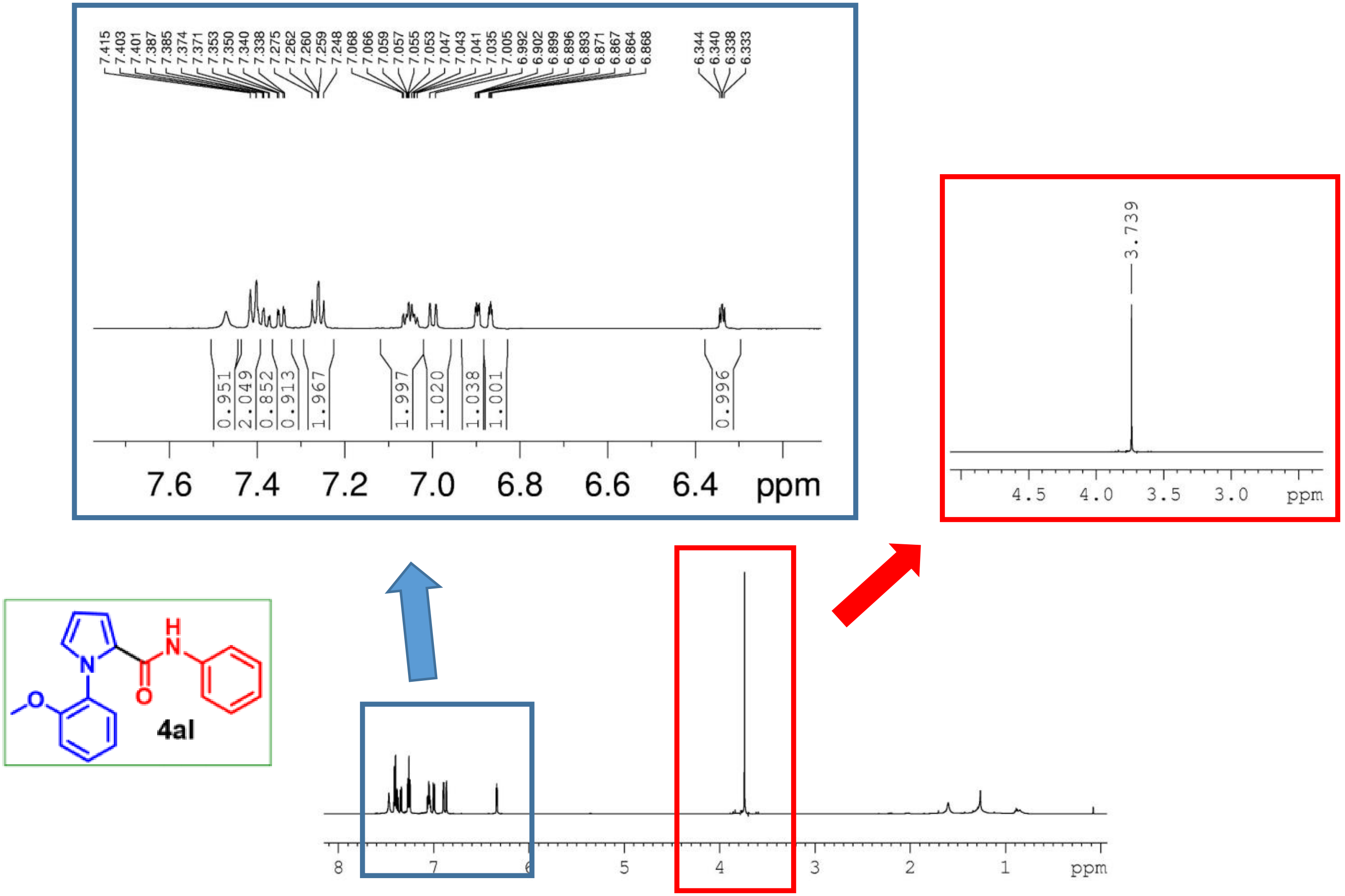

Figure S43. ^{1}H NMR spectrum (600 MHz, $CDCl_3$, 298 K) of the derivative **4al**, with expansions of significant portions of the spectrum in blue, red squares.

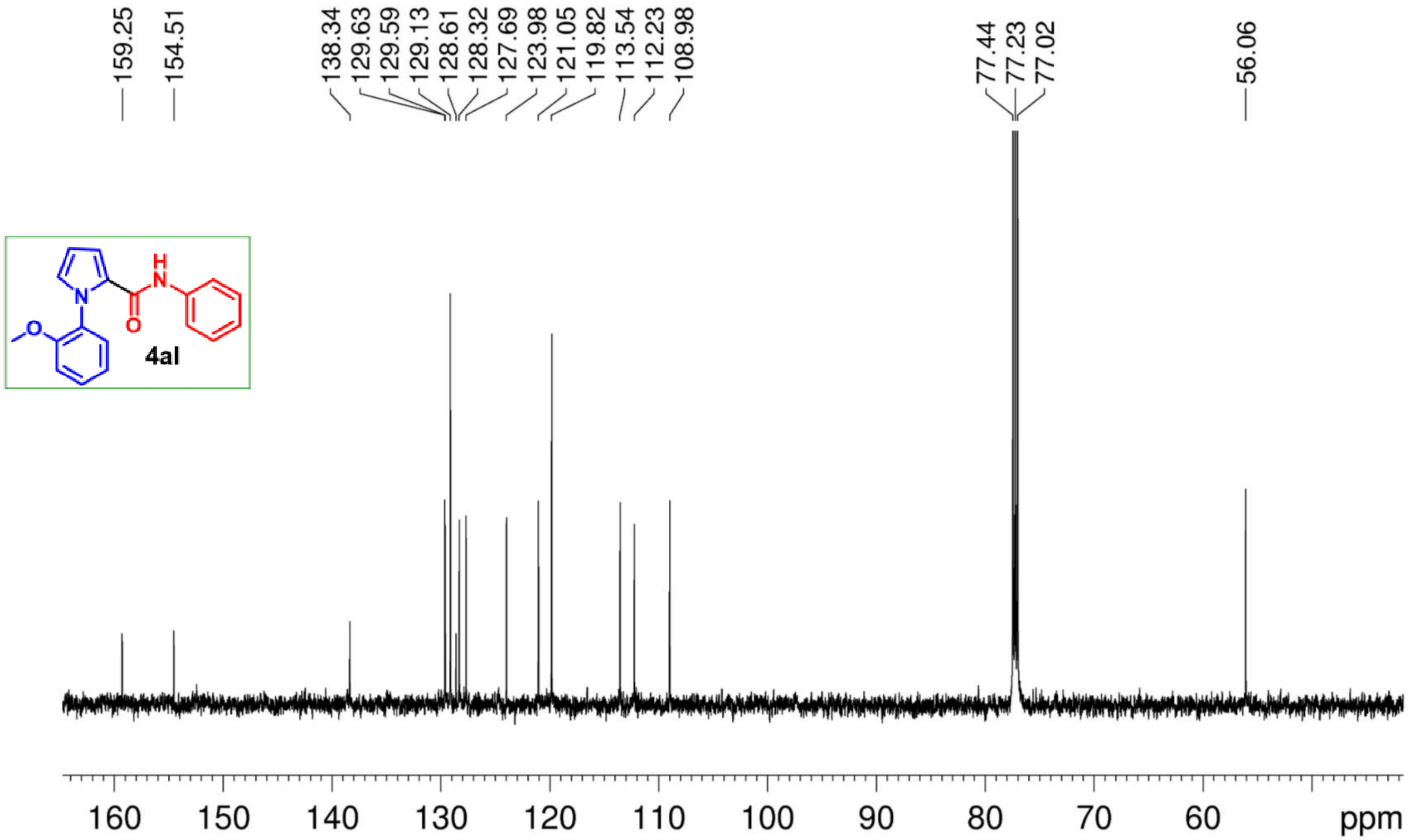

Figure S44. ^{13}C NMR spectrum (150 MHz, $CDCl_3$, 298 K) of the derivative **4al**.

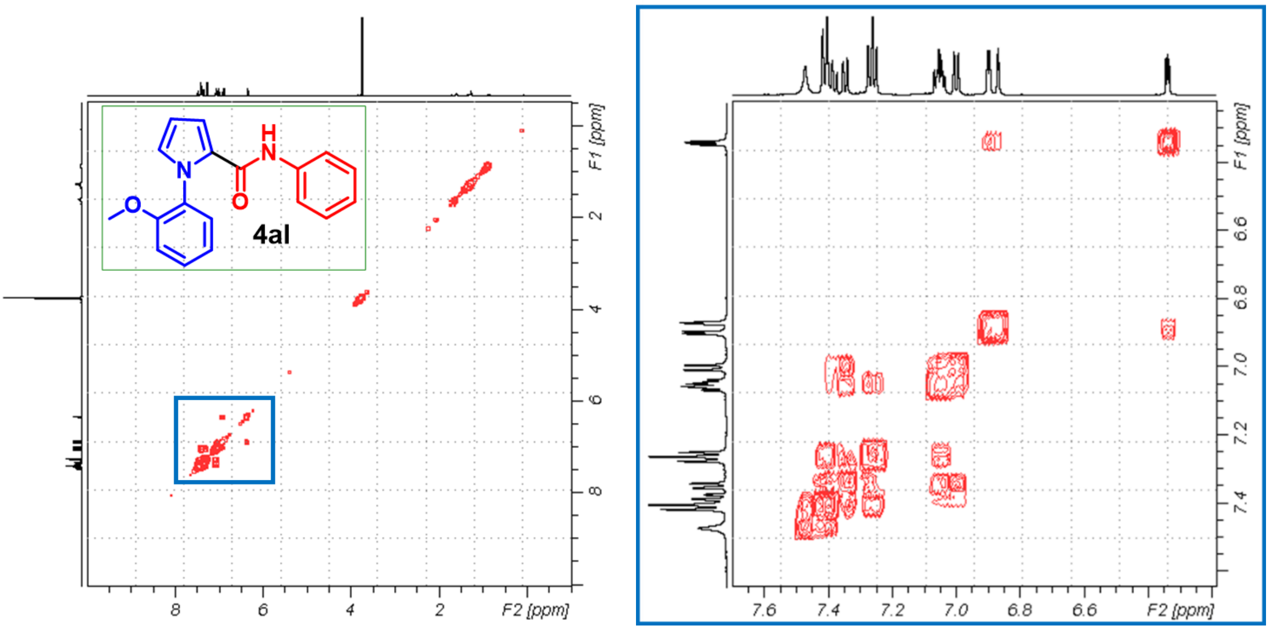

Figure S45. COSY NMR spectrum (600 MHz, $CDCl_3$, 298 K) of the derivative **4al**, with expansion of significant portion of the spectrum in blue square.

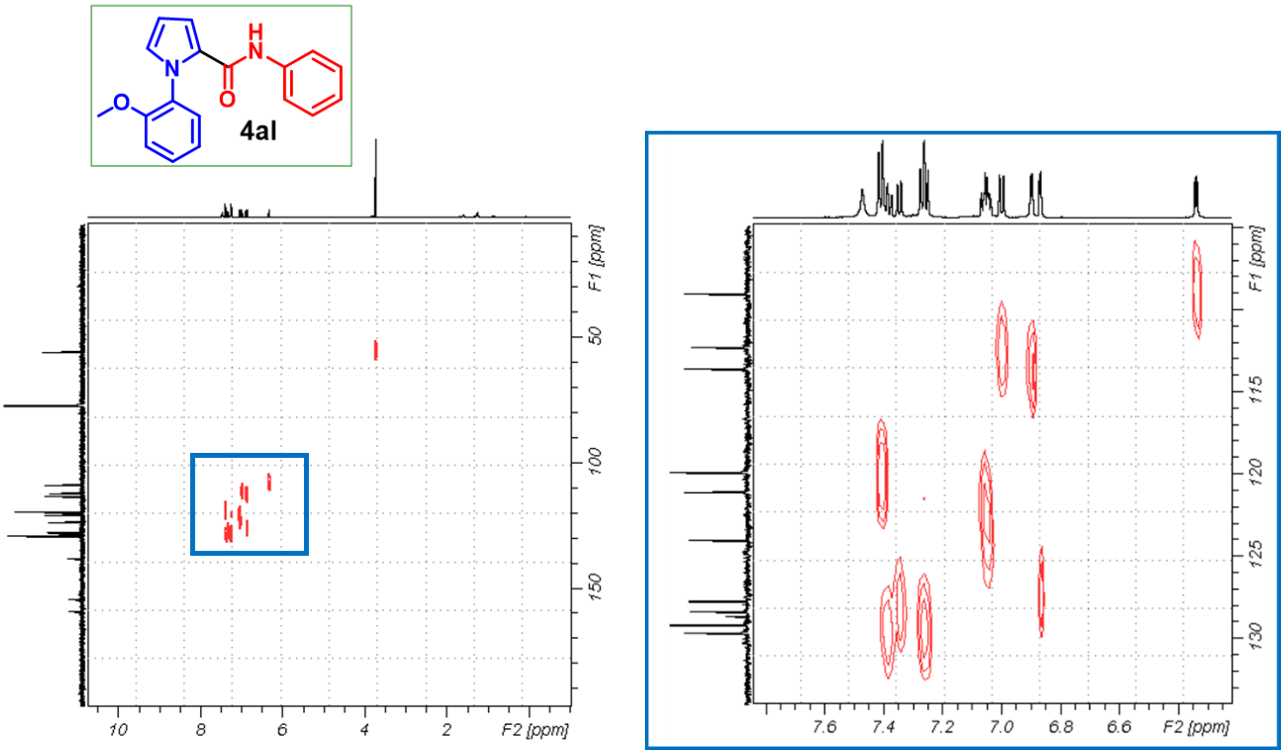

Figure S46. HSQC NMR spectrum (600 MHz, $CDCl_3$, 298 K) of the derivative **4al**, with expansion of significant portion of the spectrum in blue square.

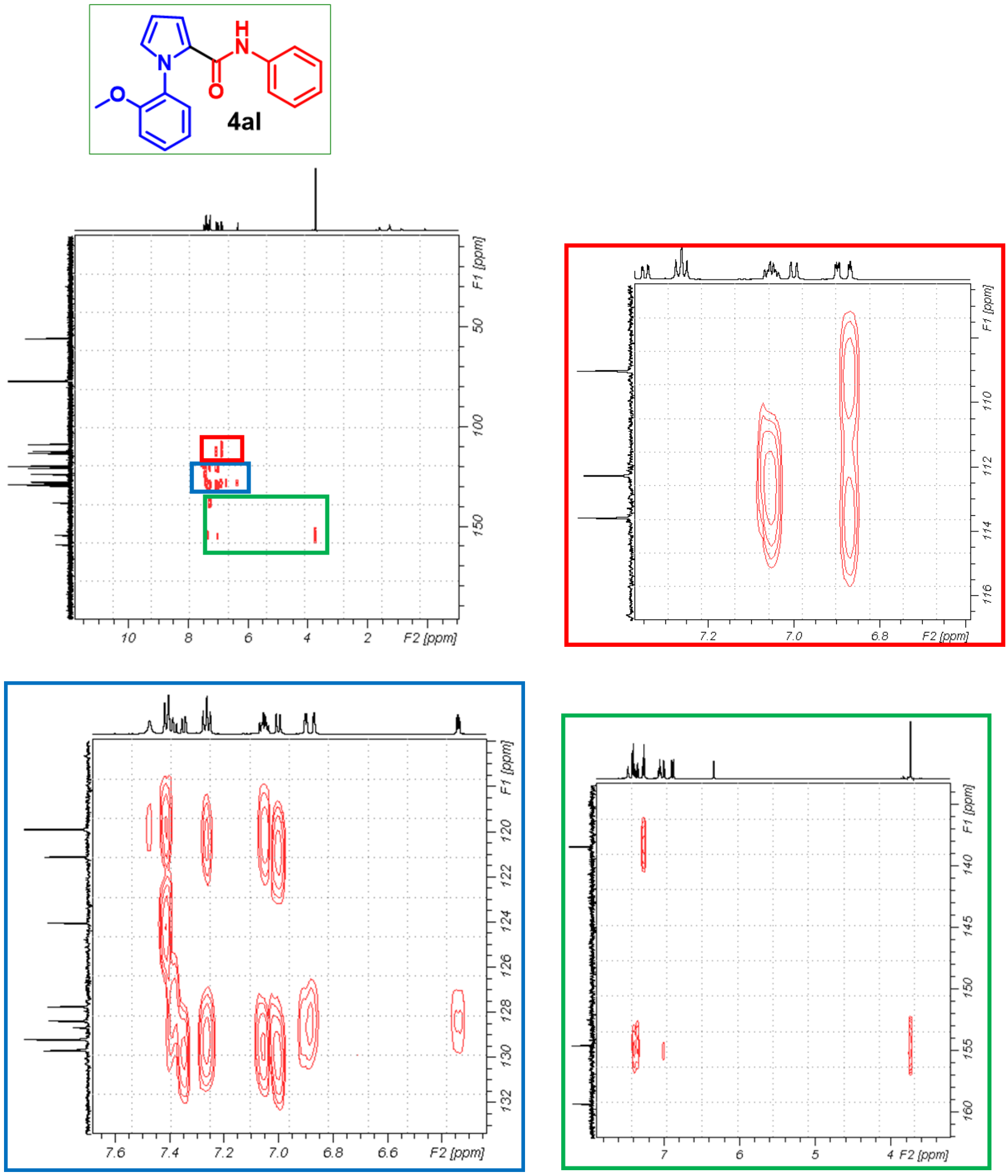

Figure S47. HMBC NMR spectrum (600 MHz, $CDCl_3$, 298 K) of the derivative **4al**, with expansions of significant portions of the spectrum in blue, red and green squares.

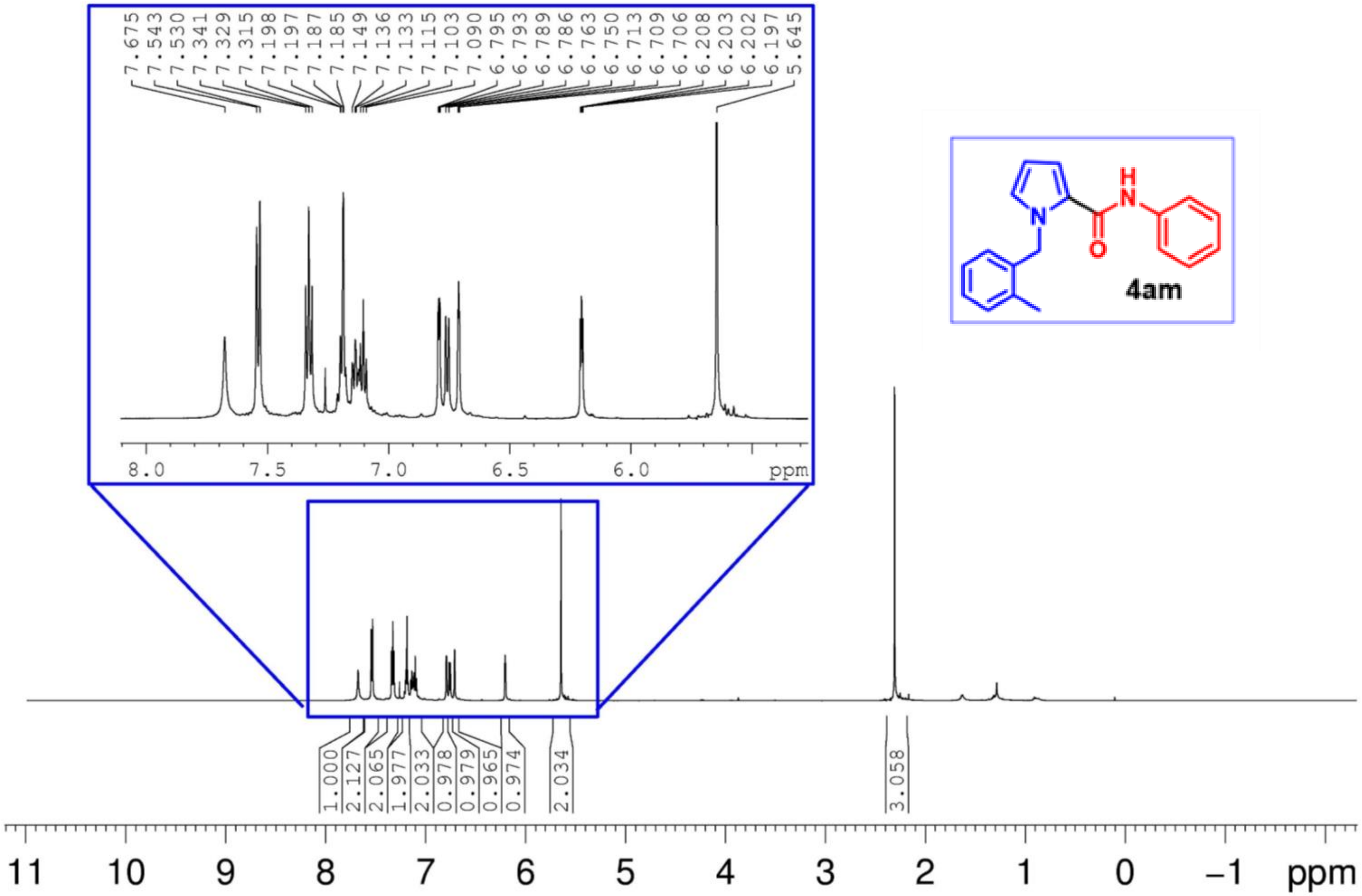

Figure S48. ^{1}H NMR spectrum (600 MHz, $CDCl_3$, 298 K) of the derivative **4am**, with expansion of significant portion of the spectrum in blue square.

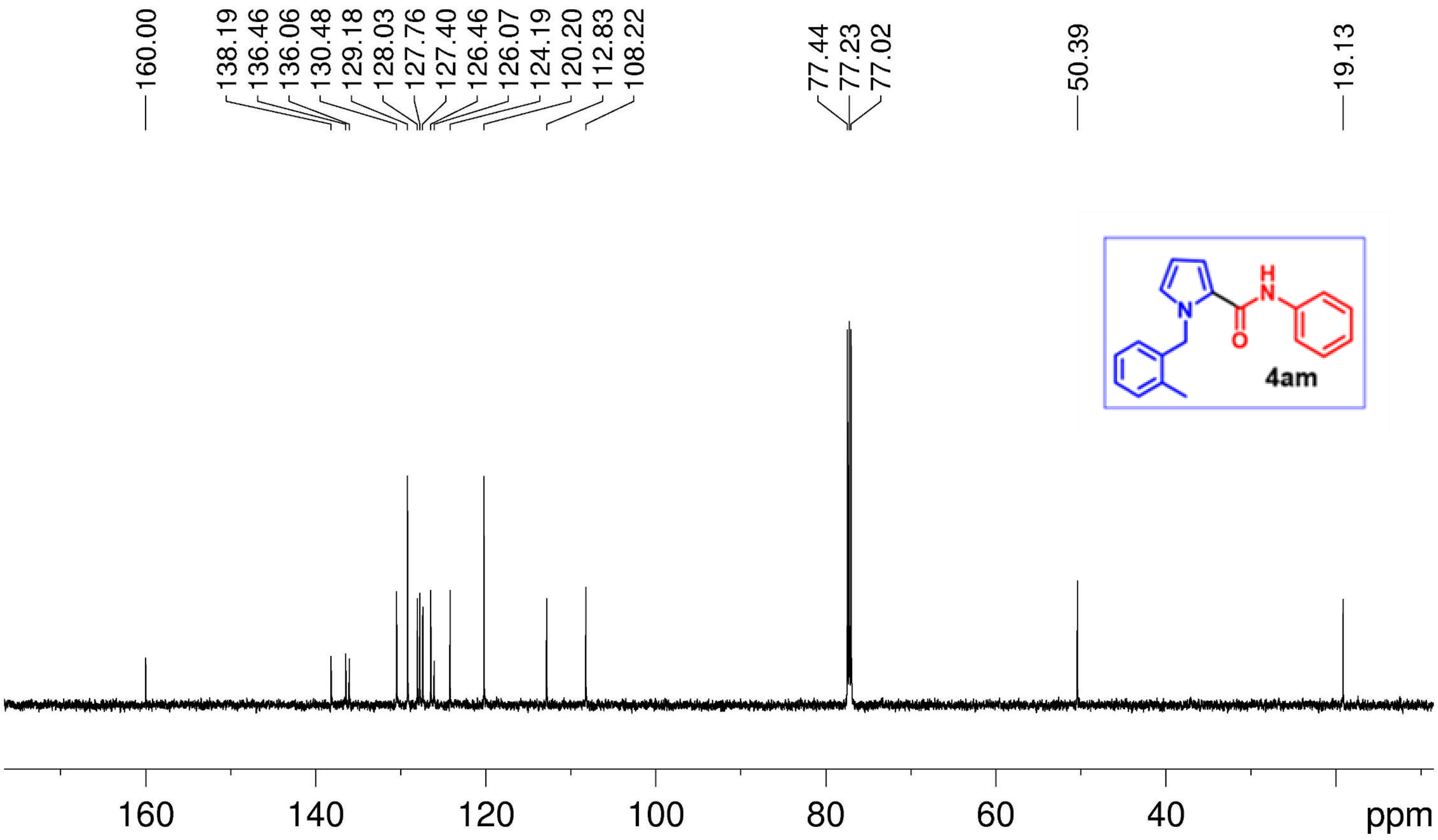

Figure S49. ^{13}C NMR spectrum (150 MHz, $CDCl_3$, 298 K) of the derivative **4am**.

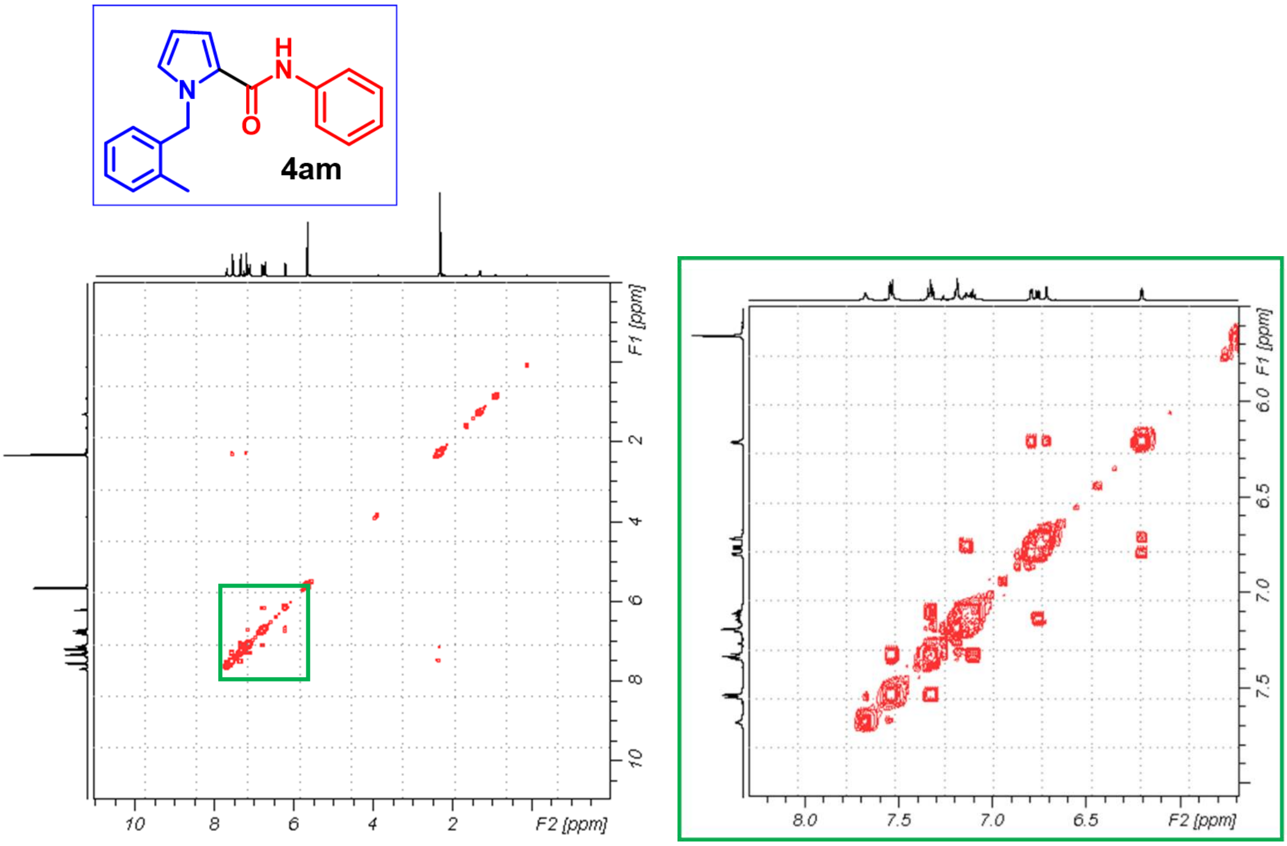

Figure S50. COSY NMR spectrum (600 MHz, $CDCl_3$, 298 K) of the derivative **4am**, with expansion of significant portion of the spectrum in green square.

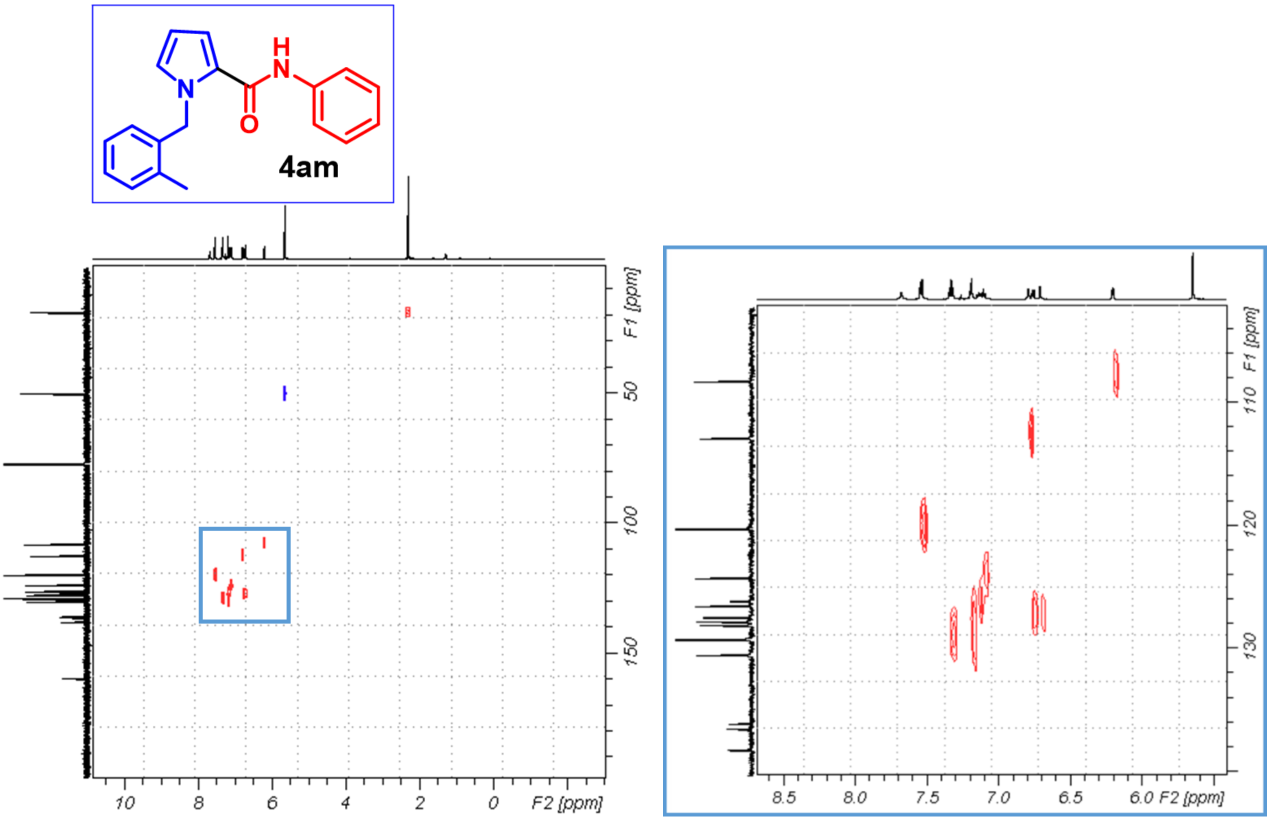

Figure S51. HSQC NMR spectrum (600 MHz, $CDCl_3$, 298 K) of the derivative **4am**, with expansion of significant portion of the spectrum in blue square.

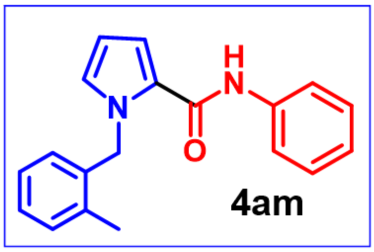

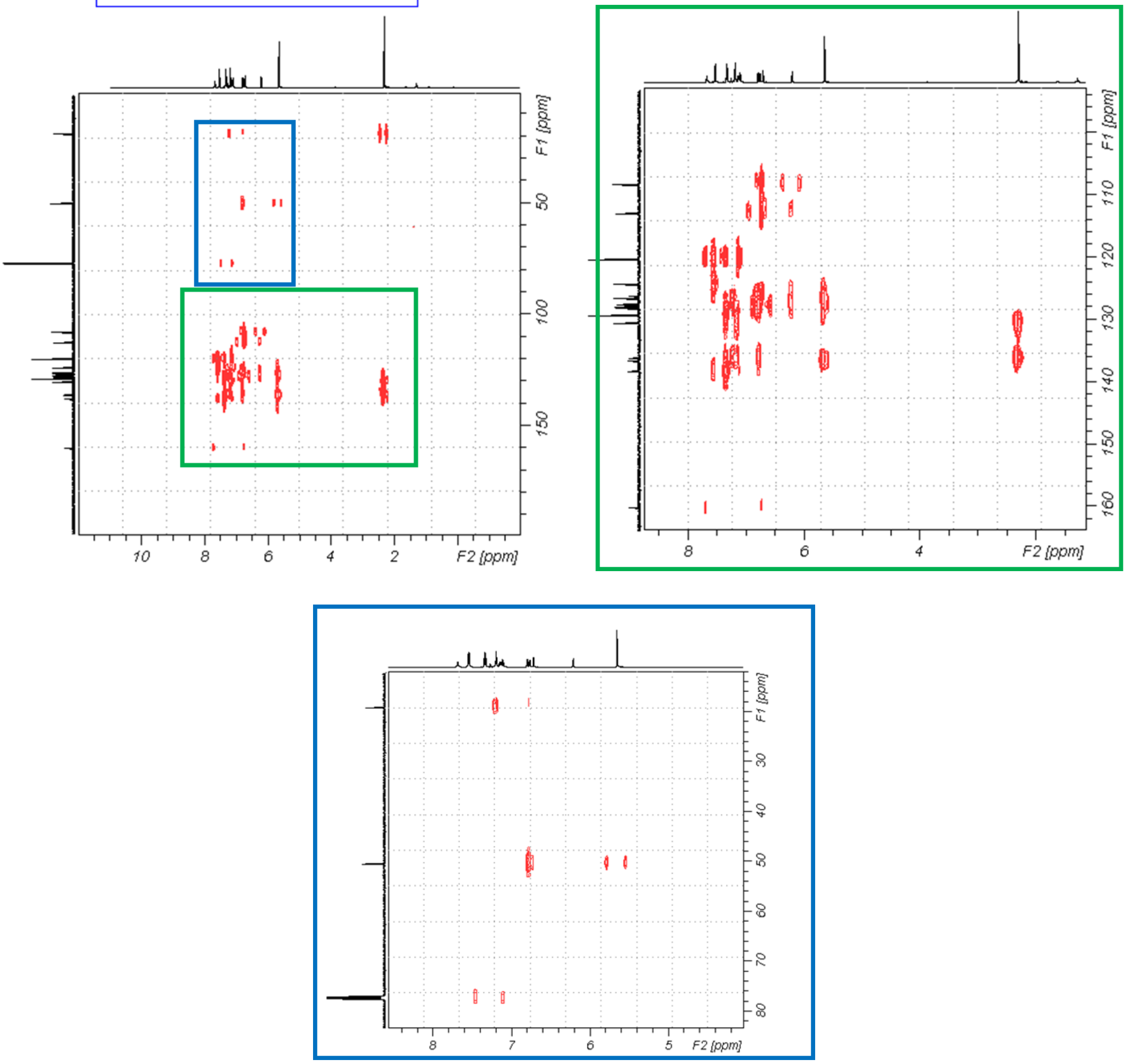

Figure S52. HMBC NMR spectrum (600 MHz, $CDCl_3$, 298 K) of the derivative **4am**, with expansions of significant portions of the spectrum in blue and green squares.

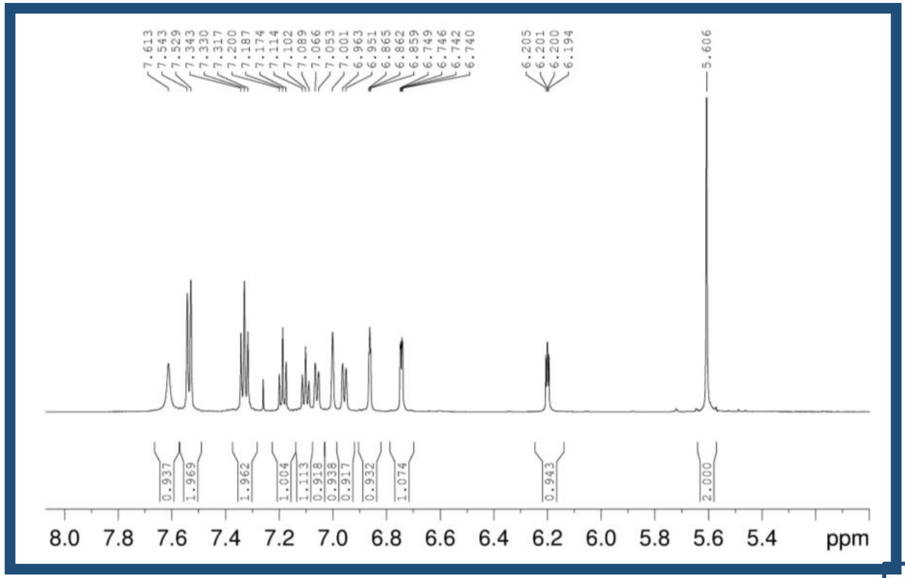

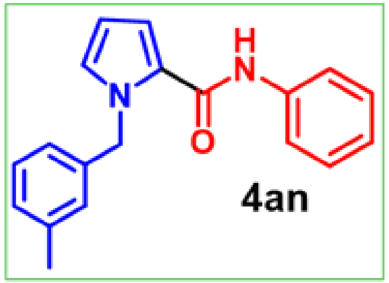

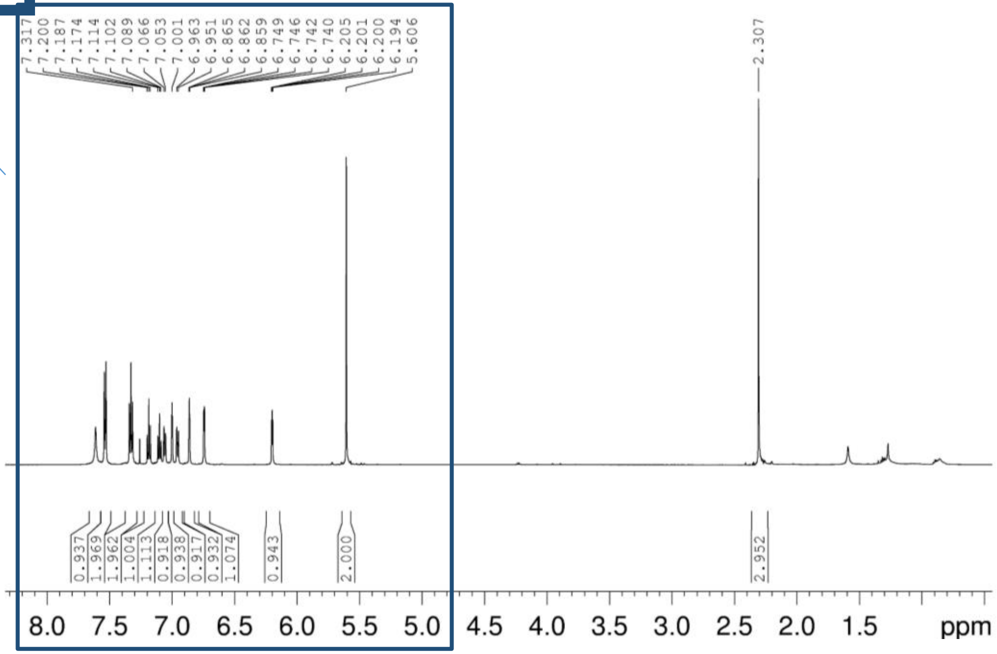

Figure S53. ^{1}H NMR spectrum (600 MHz, $CDCl_3$, 298 K) of the derivative **4an**, with expansion of significant portion of the spectrum in blue square.

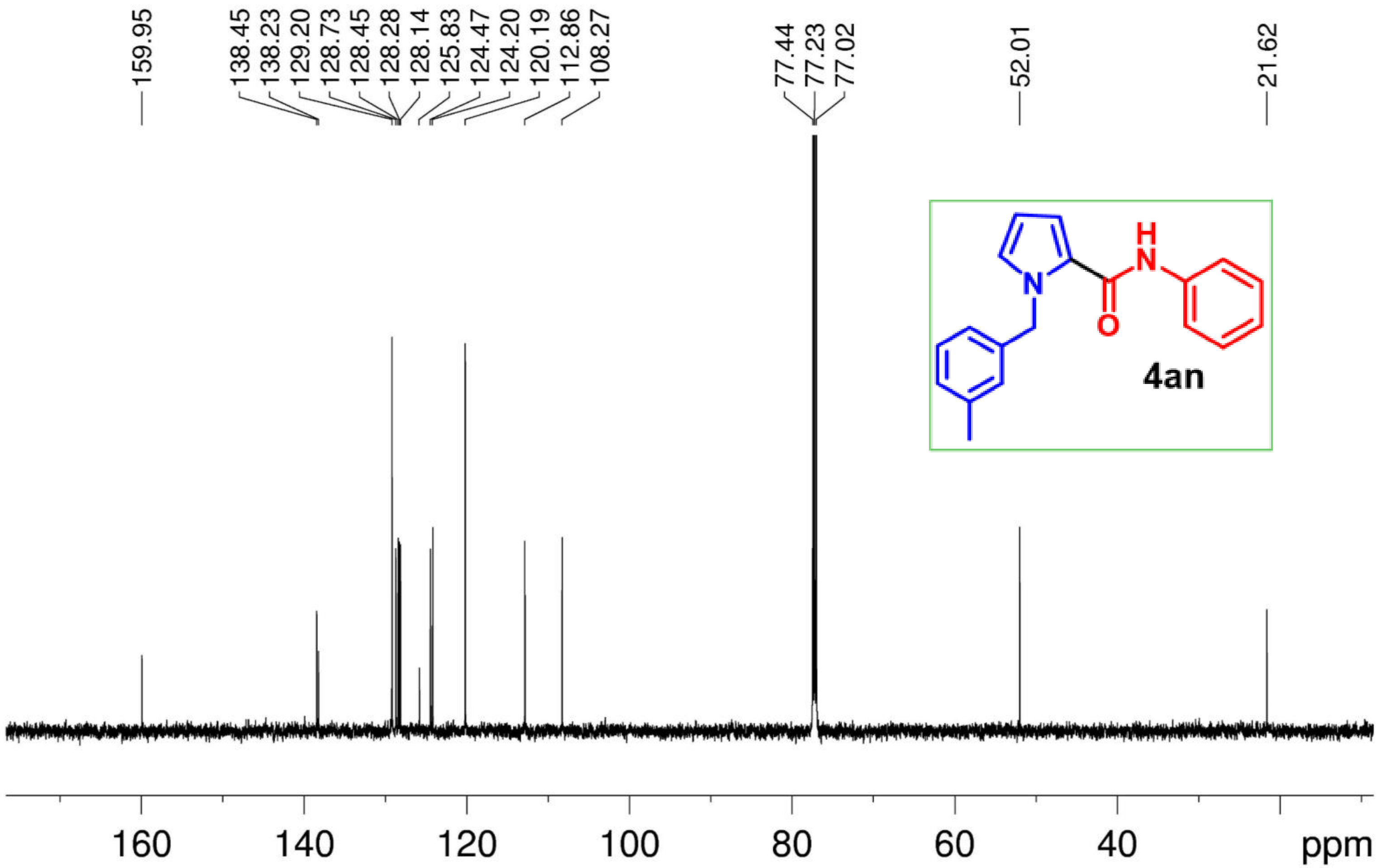

Figure S54. ^{13}C NMR spectrum (150 MHz, $CDCl_3$, 298 K) of the derivative **4an**.

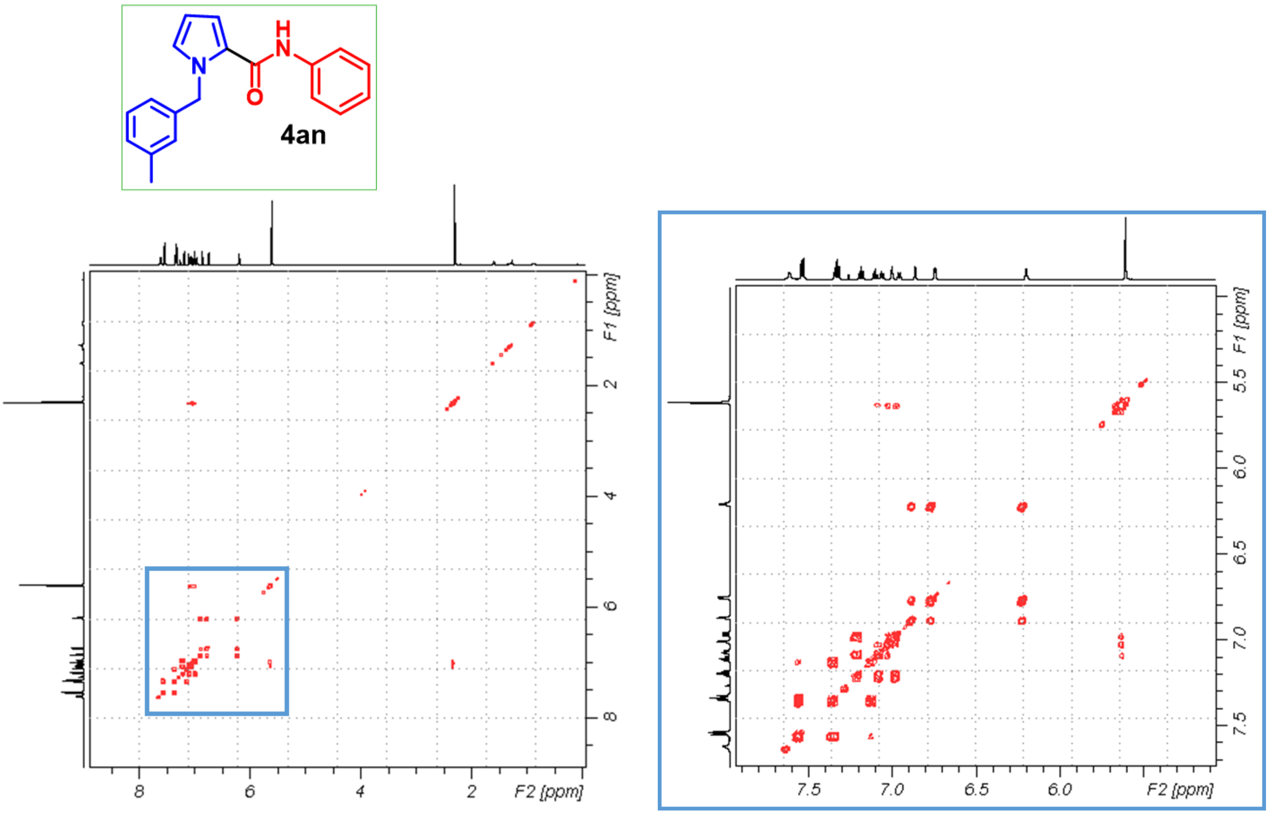

Figure S55. COSY NMR spectrum (600 MHz, $CDCl_3$, 298 K) of the derivative **4an**, with expansion of significant portion of the spectrum in blue square.

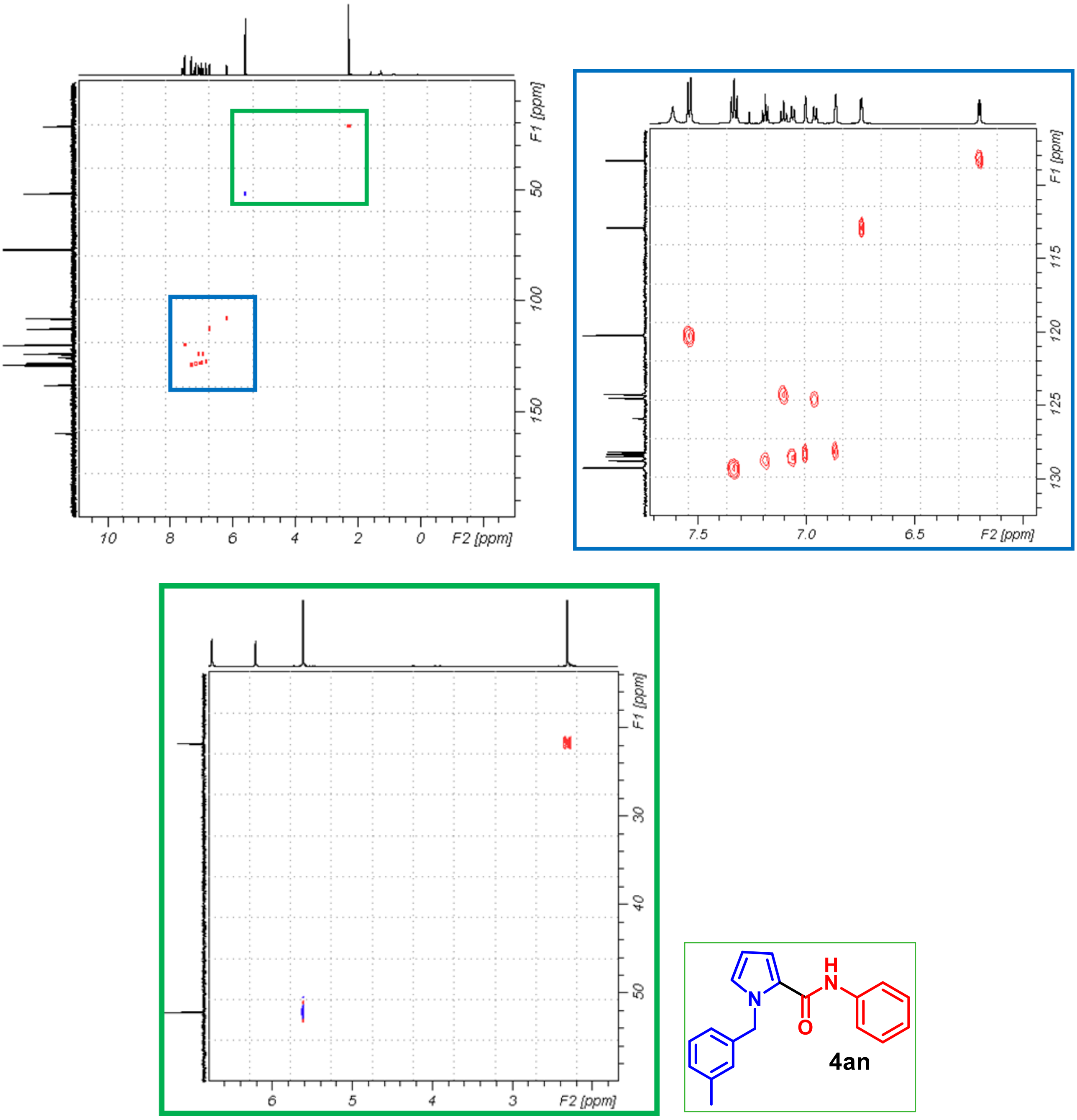

Figure S56. HSQC NMR spectrum (600 MHz, $CDCl_3$, 298 K) of the derivative **4an**, with expansions of significant portions of the spectrum in blue and green squares.

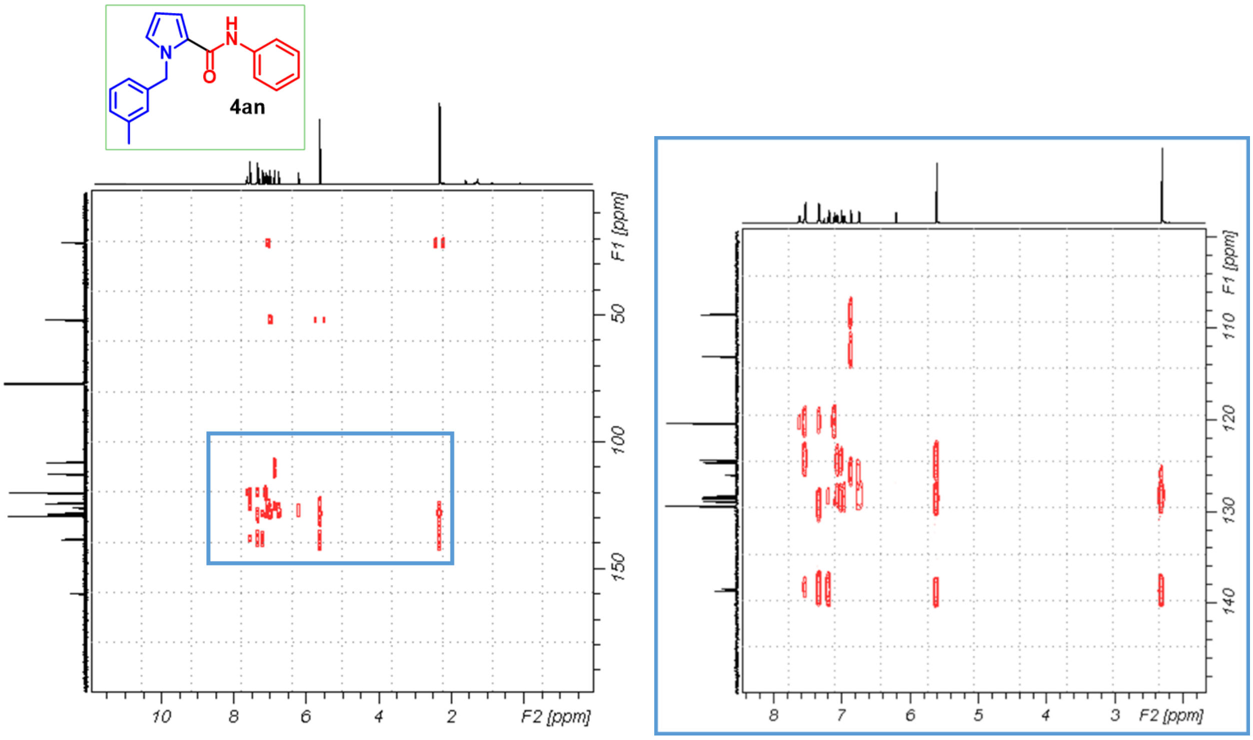

Figure S57. HMBC NMR spectrum (600 MHz, $CDCl_3$, 298 K) of the derivative **4an**, with expansion of significant portion of the spectrum in blue square.

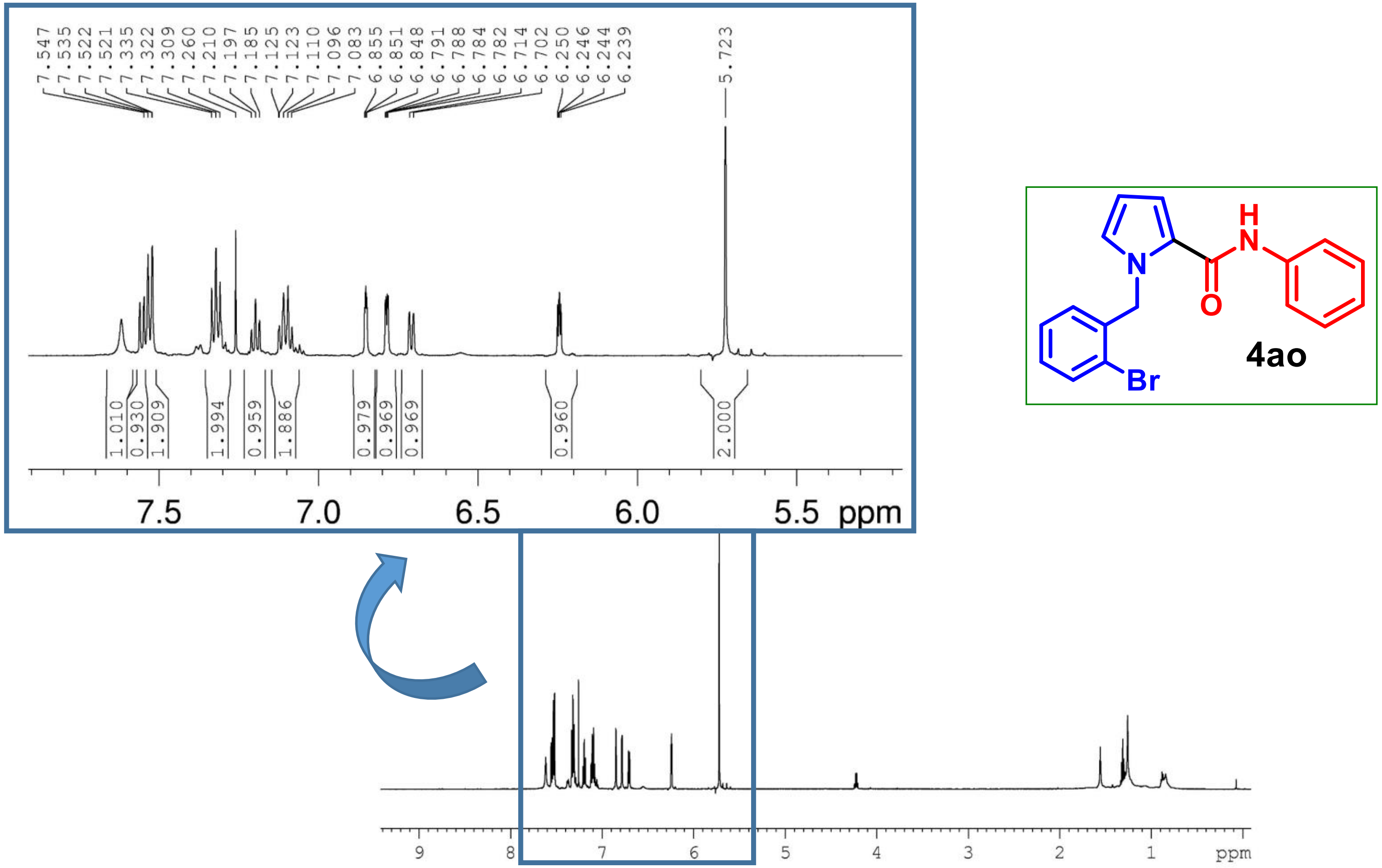

Figure S58. ^{1}H NMR spectrum (600 MHz, $CDCl_3$, 298 K) of the derivative **4ao**, with expansion of significant portion of the spectrum in blue square.

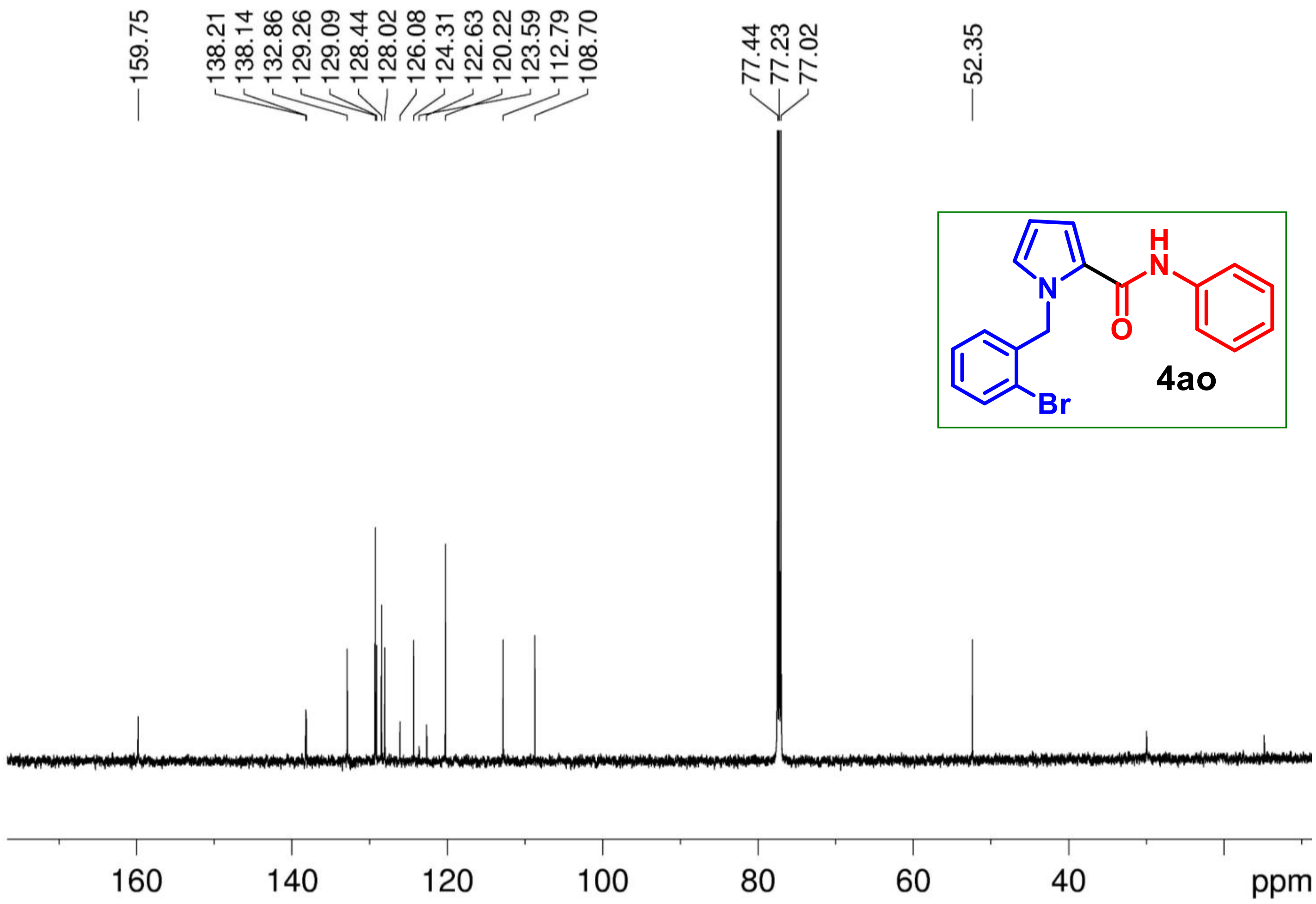

Figure S59. ^{13}C NMR spectrum (150 MHz, $CDCl_3$, 298 K) of the derivative **4ao**.

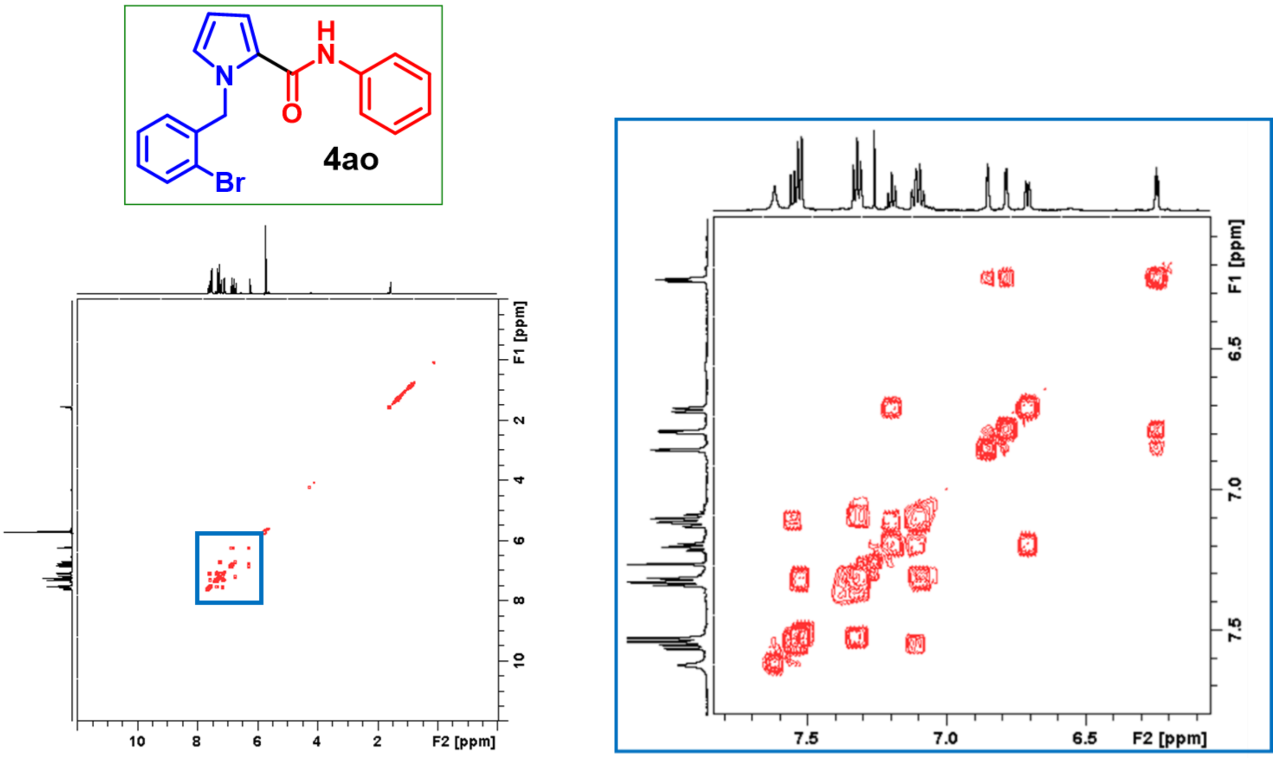

Figure S60. COSY NMR spectrum (600 MHz, $CDCl_3$, 298 K) of the derivative **4ao**, with expansion of significant portion of the spectrum in blue square.

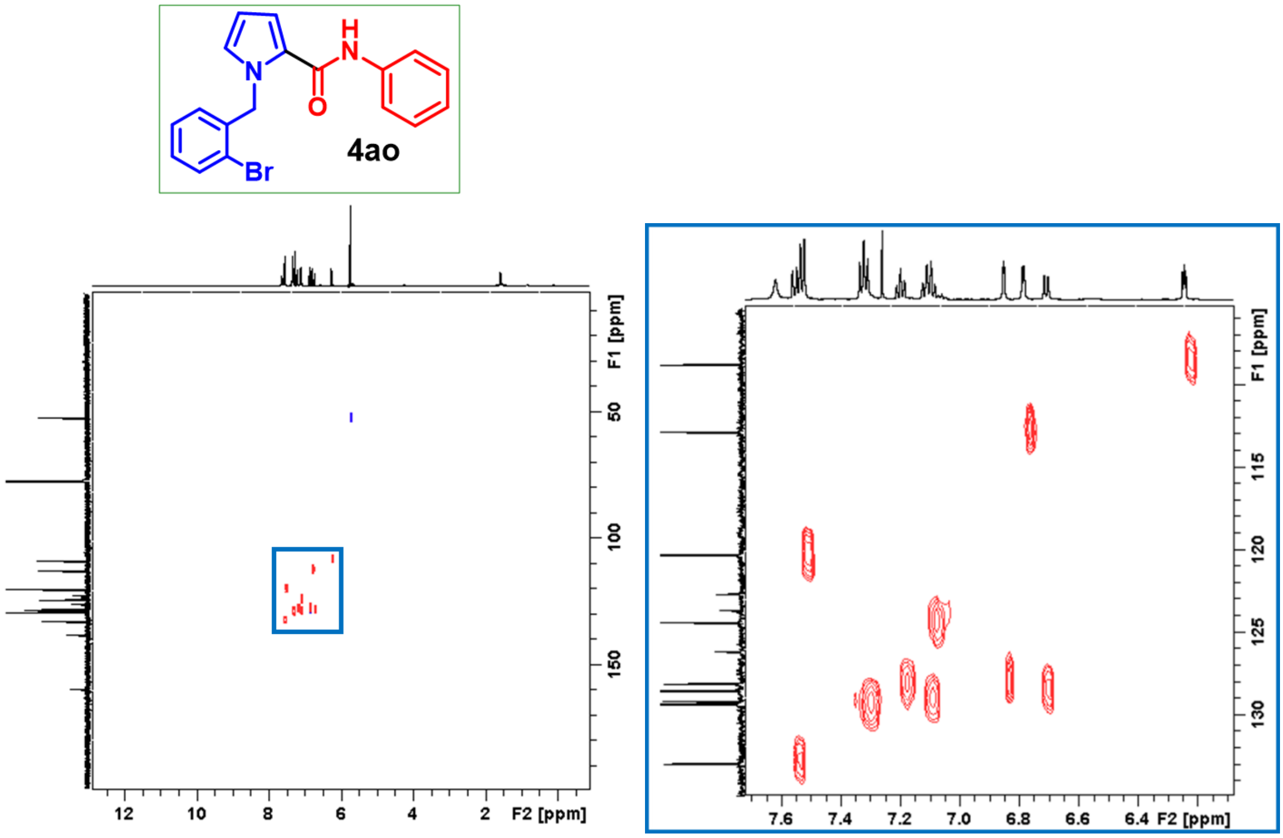

Figure S61. HSQC NMR spectrum (600 MHz, $CDCl_3$, 298 K) of the derivative **4ao**, with expansion of significant portion of the spectrum in blue square.

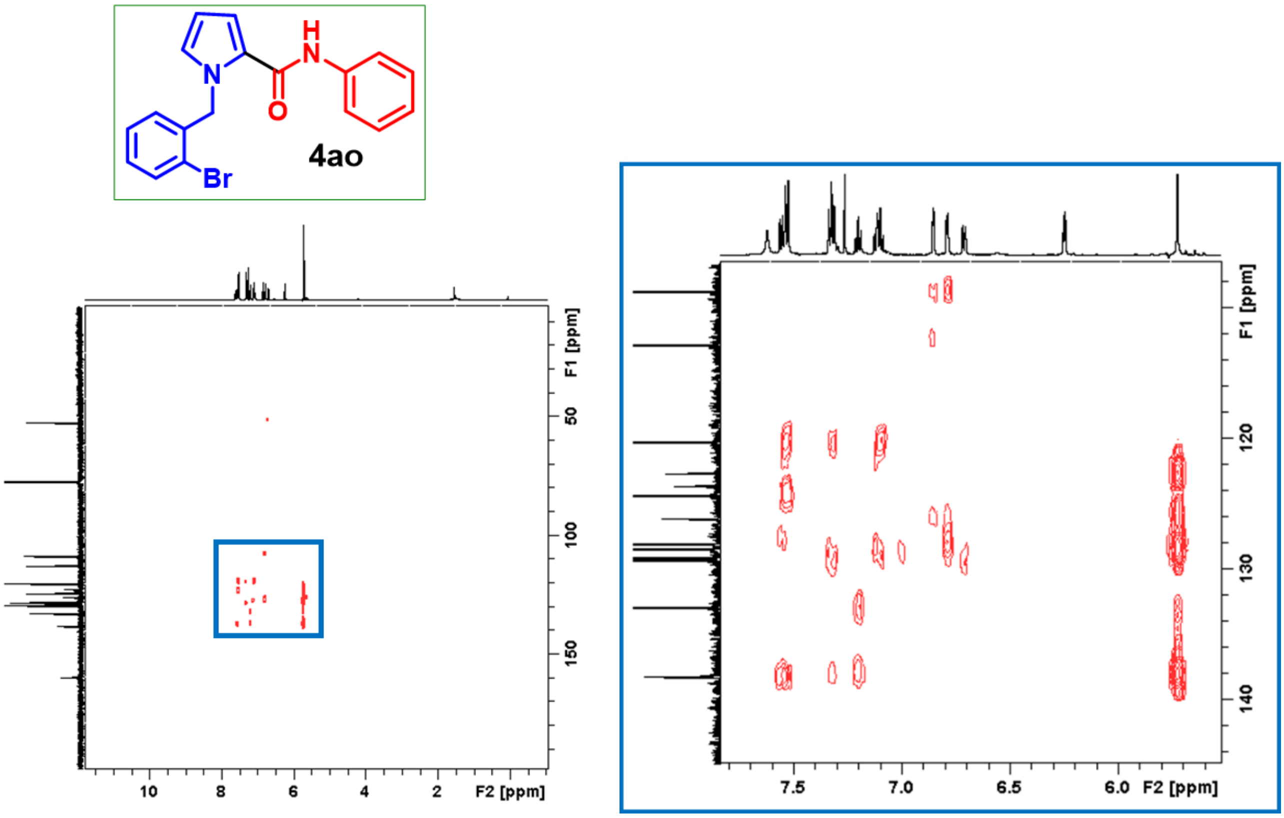

Figure S62. HMBC NMR spectrum (600 MHz, $CDCl_3$, 298 K) of the derivative **4ao**, with expansion of significant portion of the spectrum in blue square.

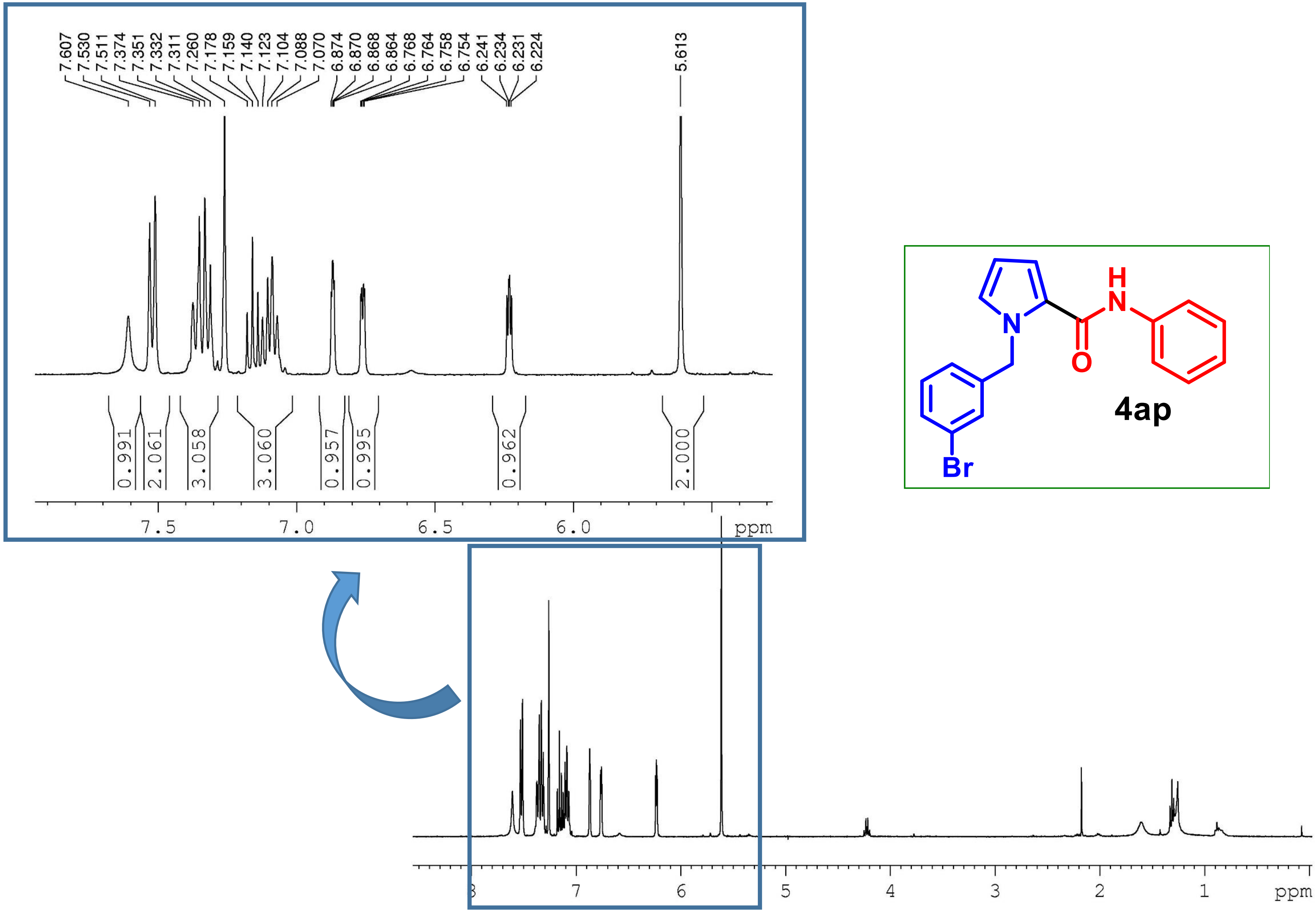

Figure S63. ^{1}H NMR spectrum (400 MHz, $CDCl_3$, 298 K) of the derivative **4ap**, with expansion of significant portion of the spectrum in blue square.

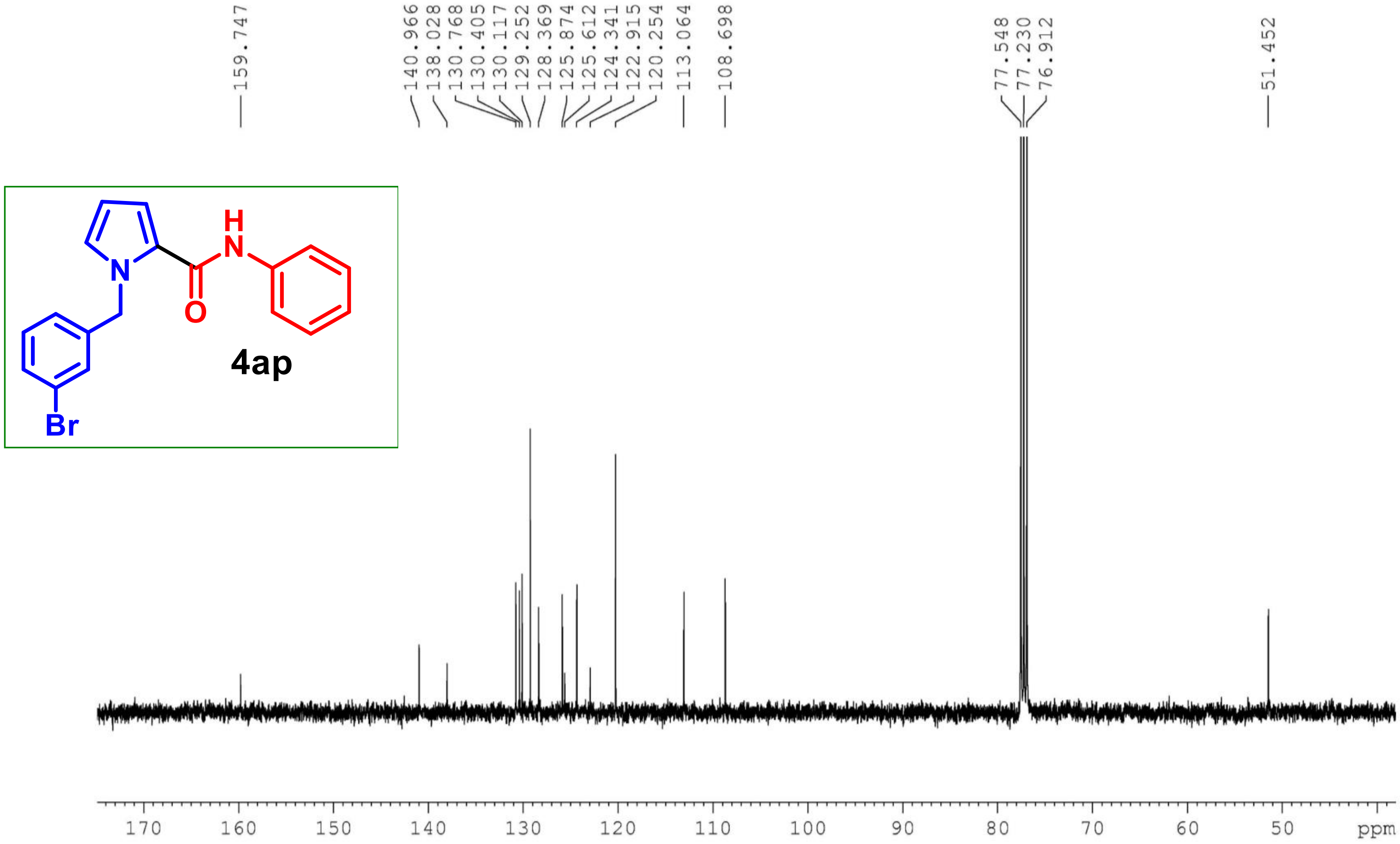

Figure S64. ^{13}C NMR spectrum (100 MHz, $CDCl_3$, 298 K) of the derivative **4ap**.

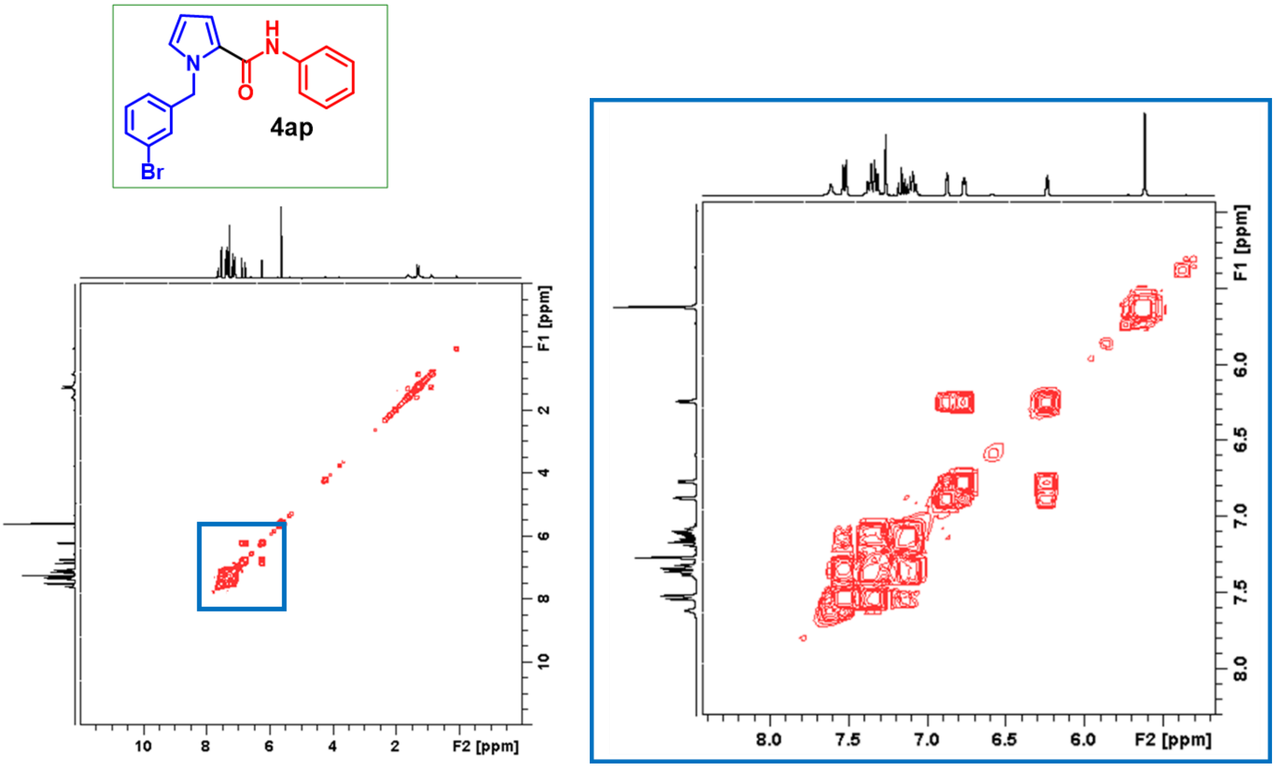

Figure S65. COSY NMR spectrum (300 MHz, $CDCl_3$, 298 K) of the derivative **4ap**, with expansion of significant portion of the spectrum in blue square.

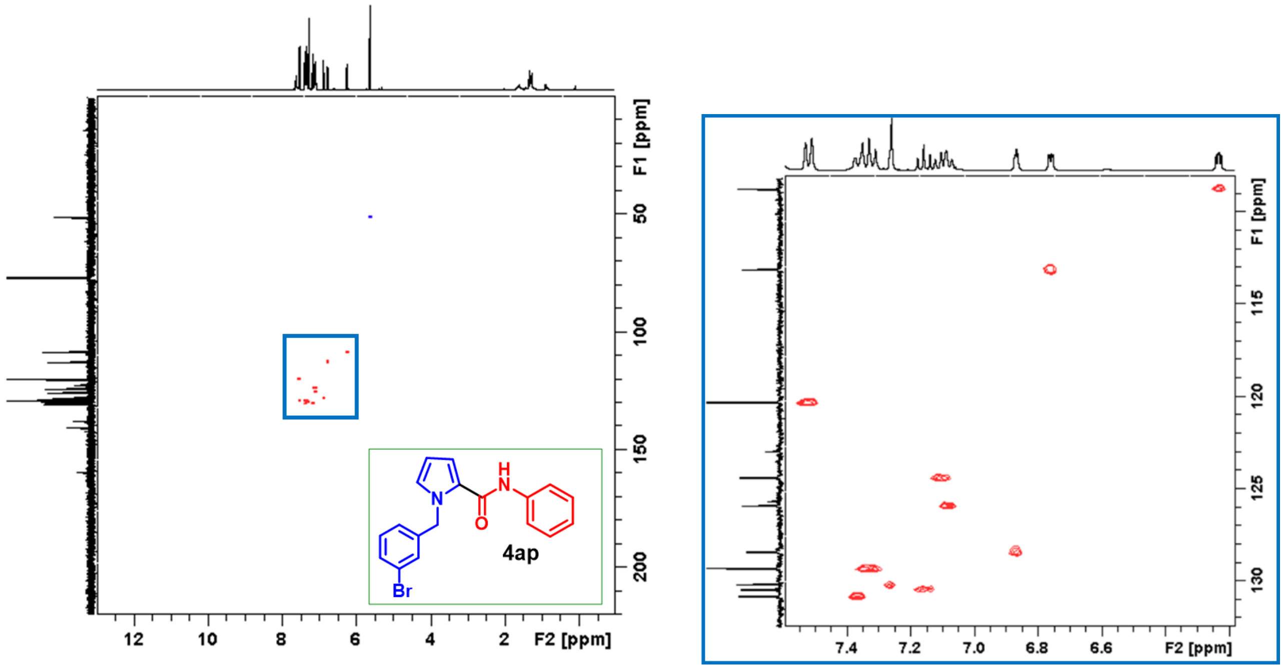

Figure S66. HSQC NMR spectrum (400 MHz, $CDCl_3$, 298 K) of the derivative **4ap**, with expansion of significant portion of the spectrum in blue square.

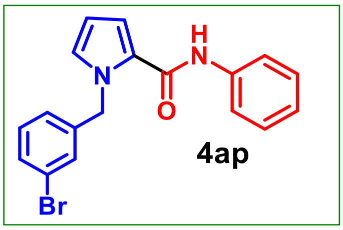

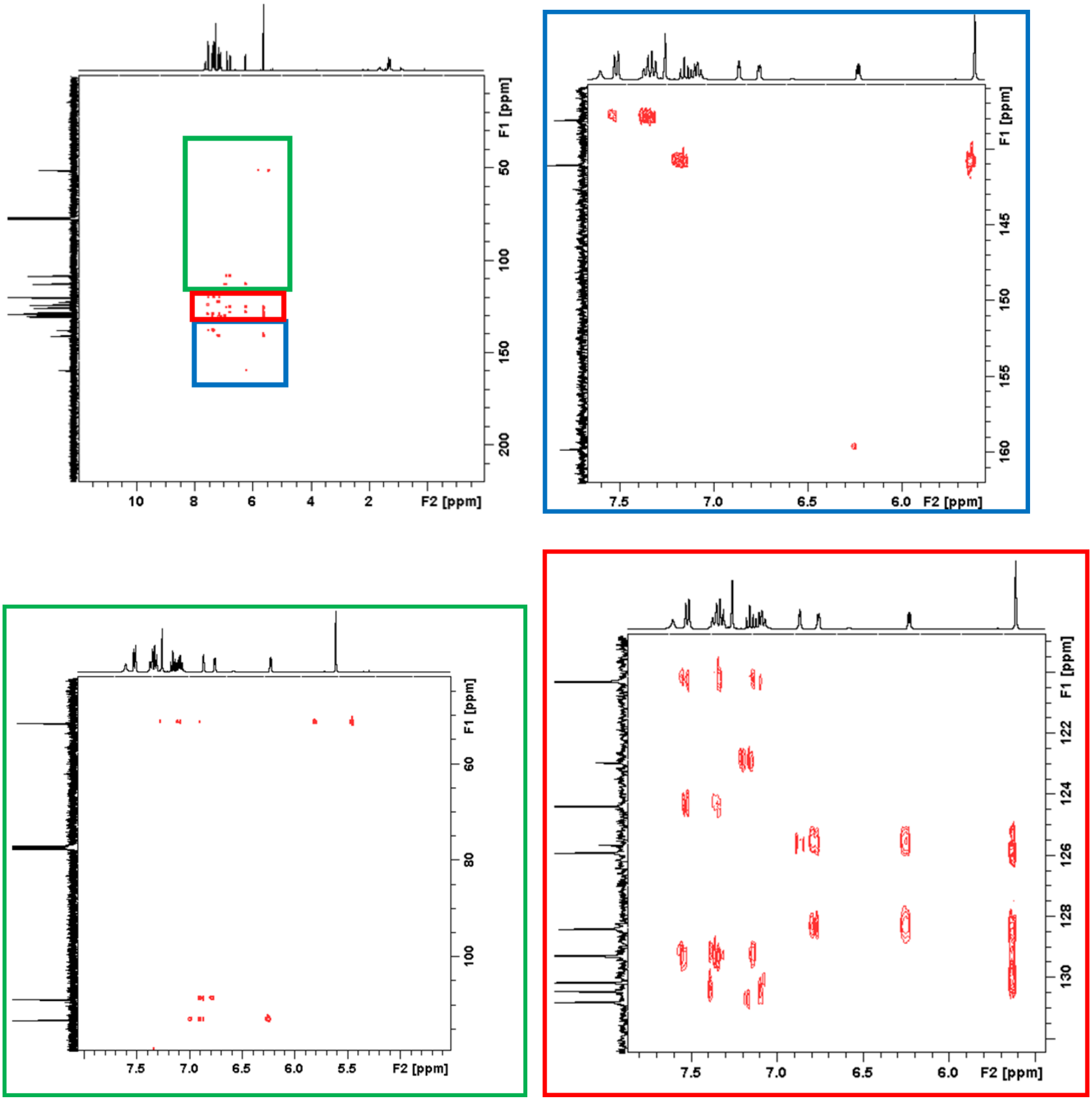

Figure S67. HMBC NMR spectrum (400 MHz, $CDCl_3$, 298 K) of the derivative **4ap**, with expansions of significant portions of the spectrum in blue, green and red squares.

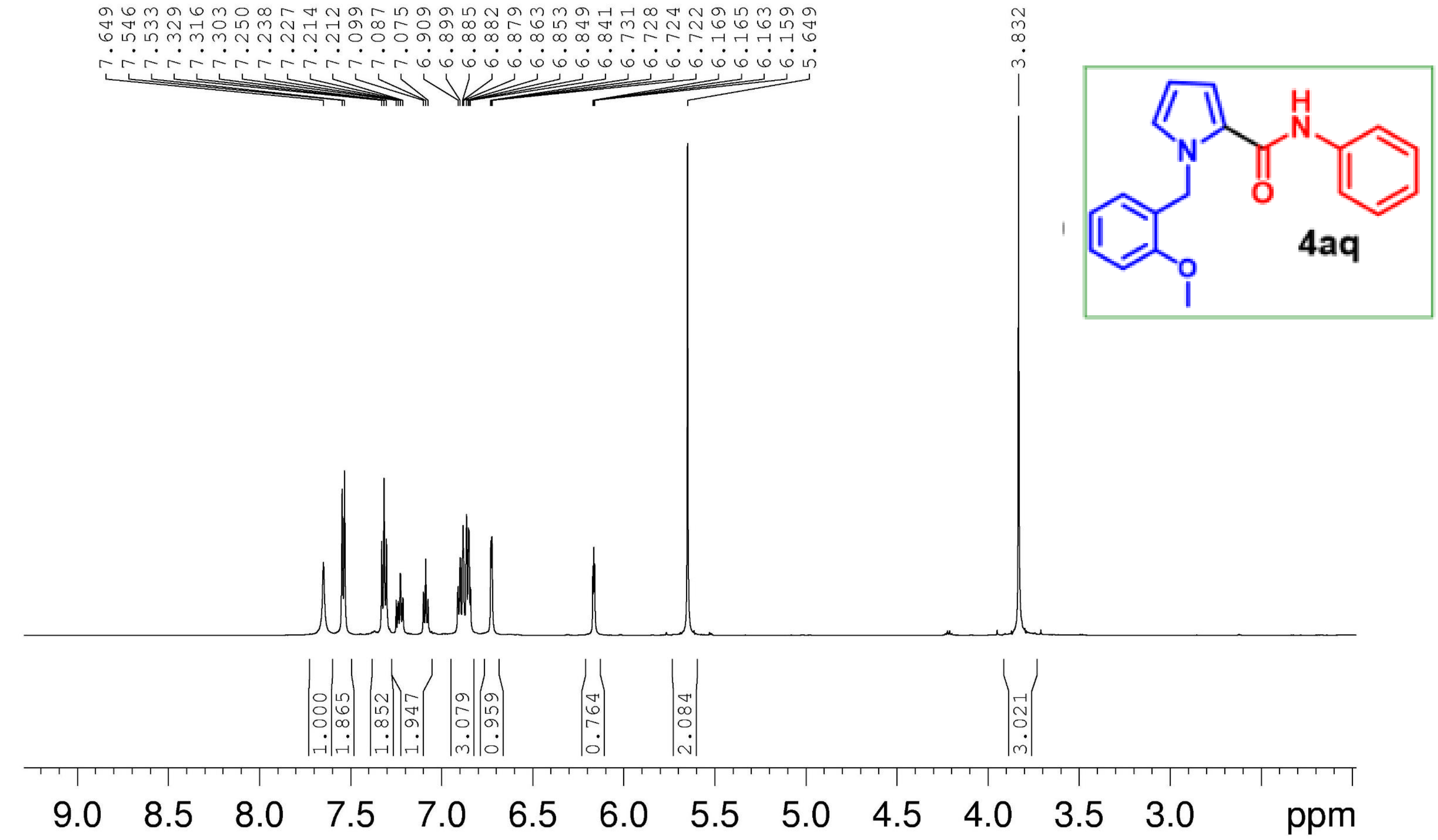

Figure S68. ^{1}H NMR spectrum (600 MHz, $CDCl_3$, 298 K) of the derivative **4aq**.

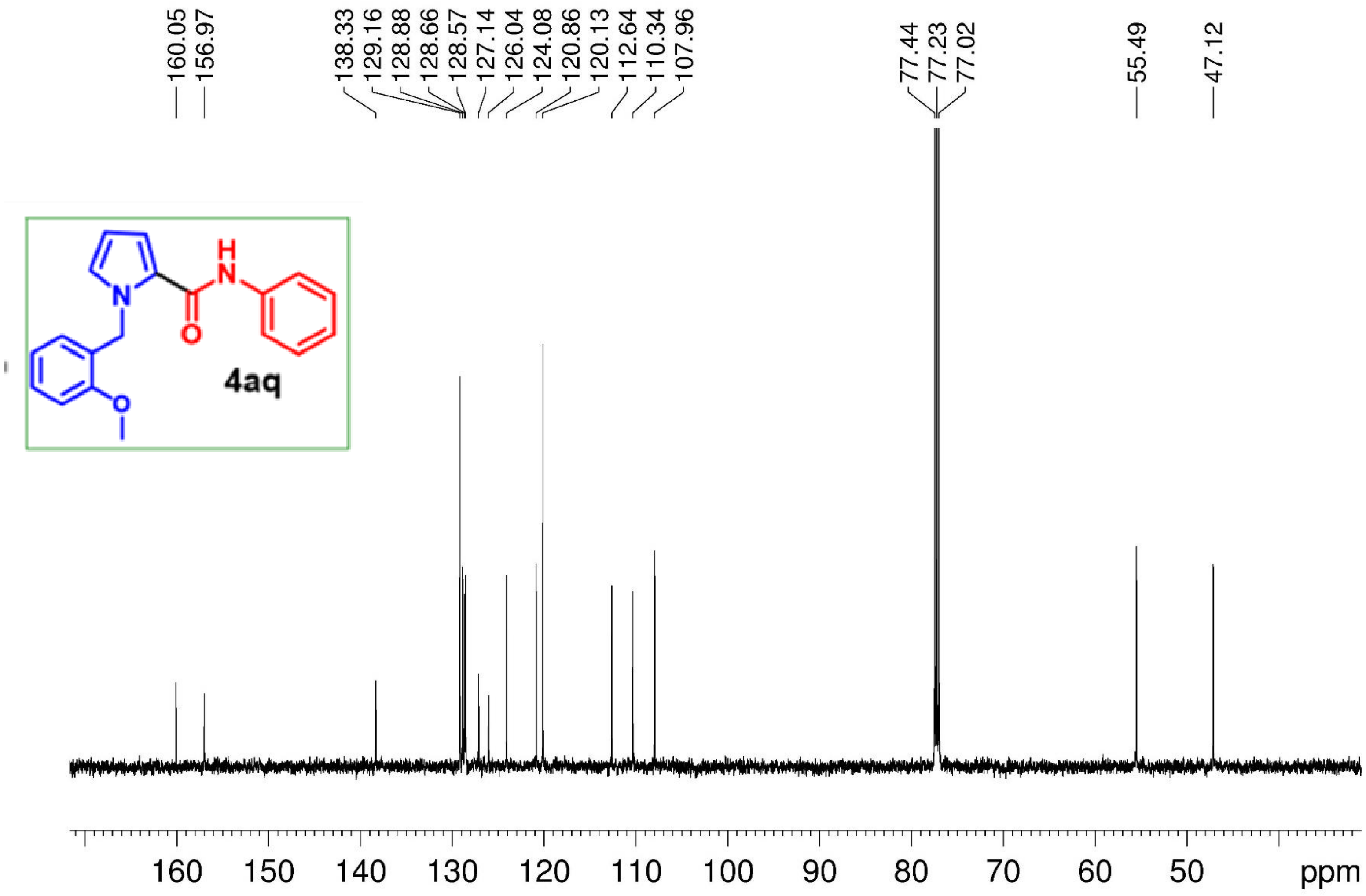

Figure S69. ^{13}C NMR spectrum (150 MHz, $CDCl_3$, 298 K) of the derivative **4aq**.

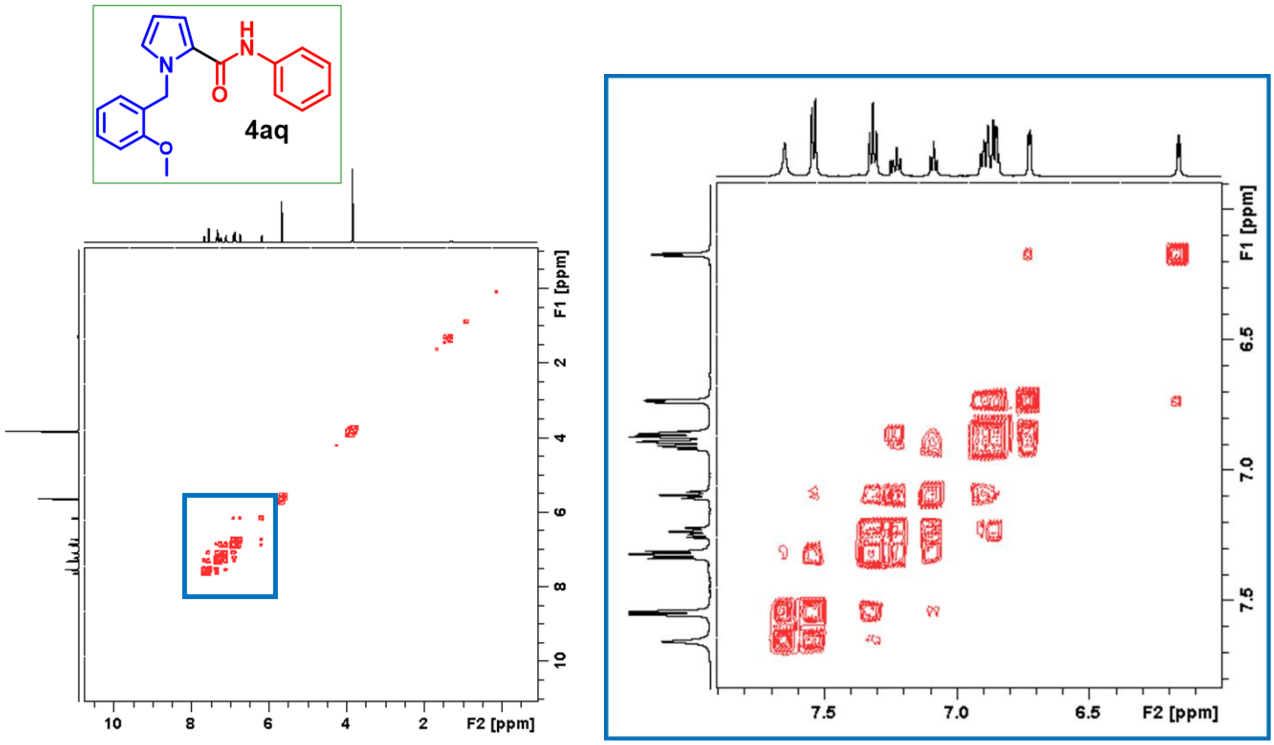

Figure S70. COSY NMR spectrum (600 MHz, $CDCl_3$, 298 K) of the derivative **4aq**, with expansion of significant portion of the spectrum in blue square.

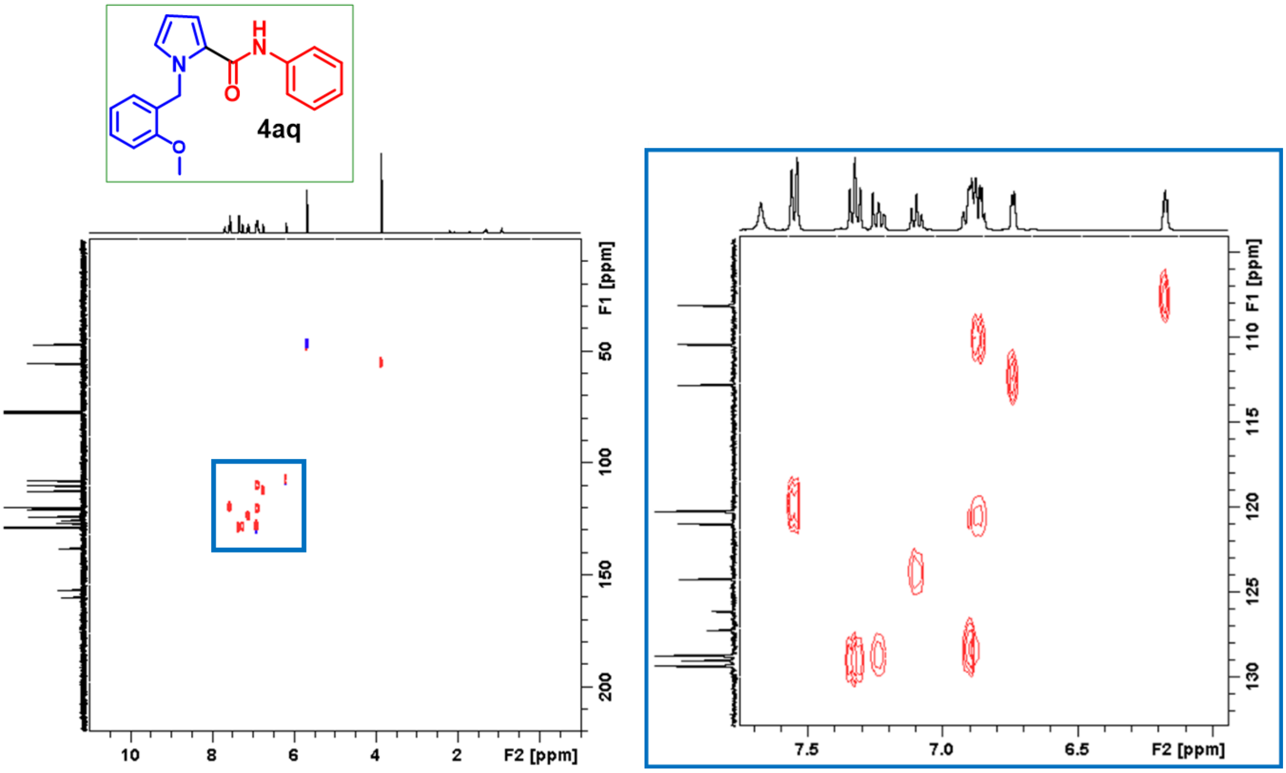

Figure S71. HSQC NMR spectrum (400 MHz, $CDCl_3$, 298 K) of the derivative **4aq**, with expansion of significant portion of the spectrum in blue square.

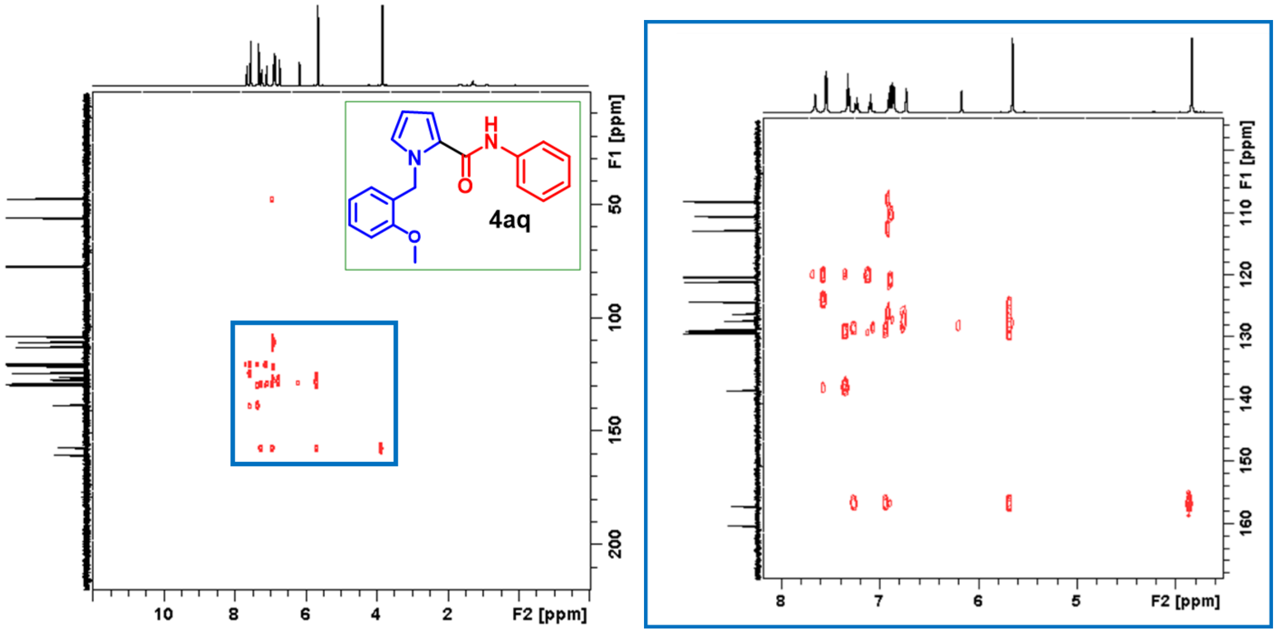

Figure S72. HMBC NMR spectrum (600 MHz, $CDCl_3$, 298 K) of the derivative **4aq**, with expansion of significant portion of the spectrum in blue square.

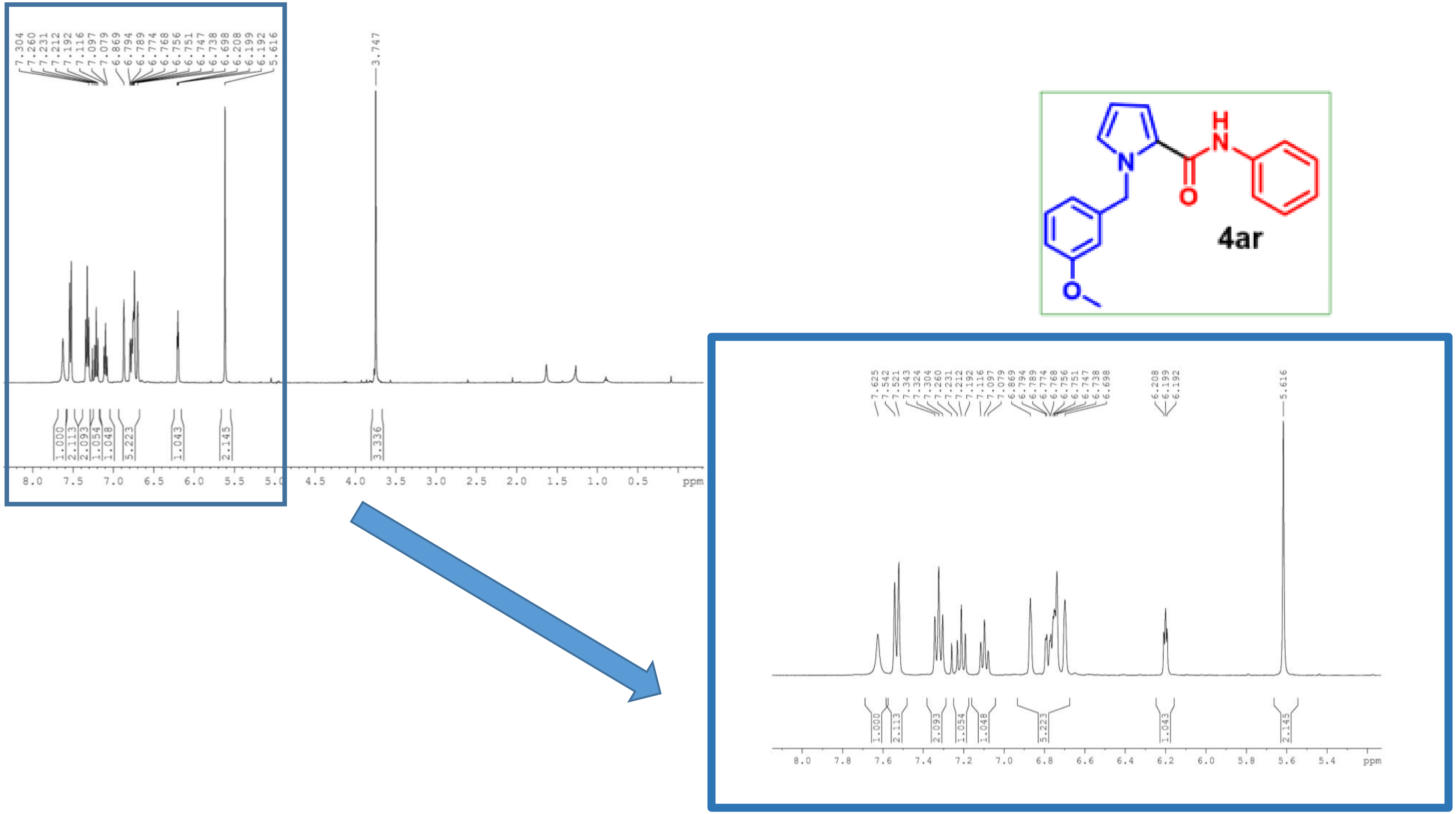

Figure S73. ^{1}H NMR spectrum (400 MHz, $CDCl_3$, 298 K) of the derivative **4ar**, with expansion of significant portion of the spectrum in blue square.

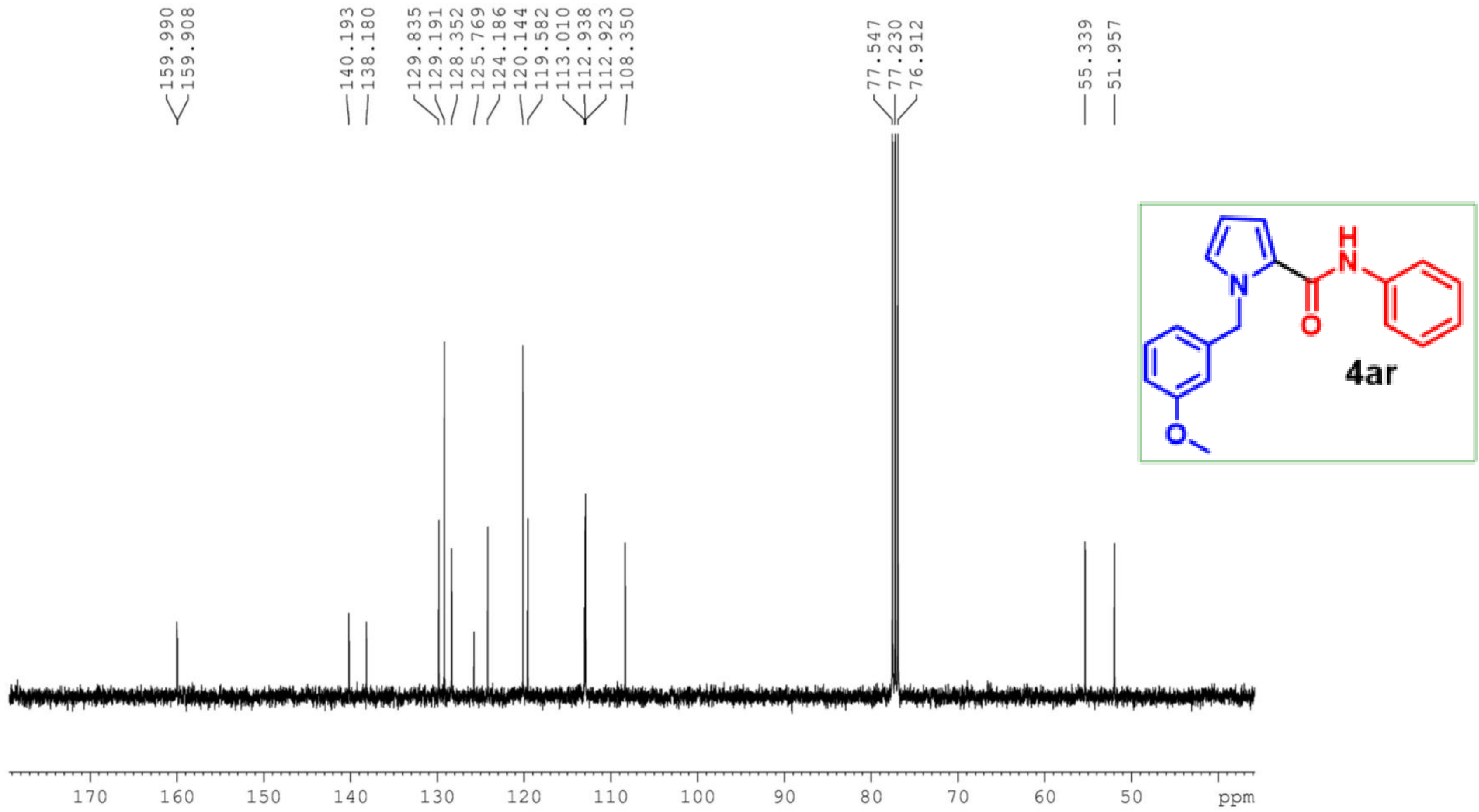

Figure S74. ^{13}C NMR spectrum (100 MHz, $CDCl_3$, 298 K) of the derivative **4ar**.

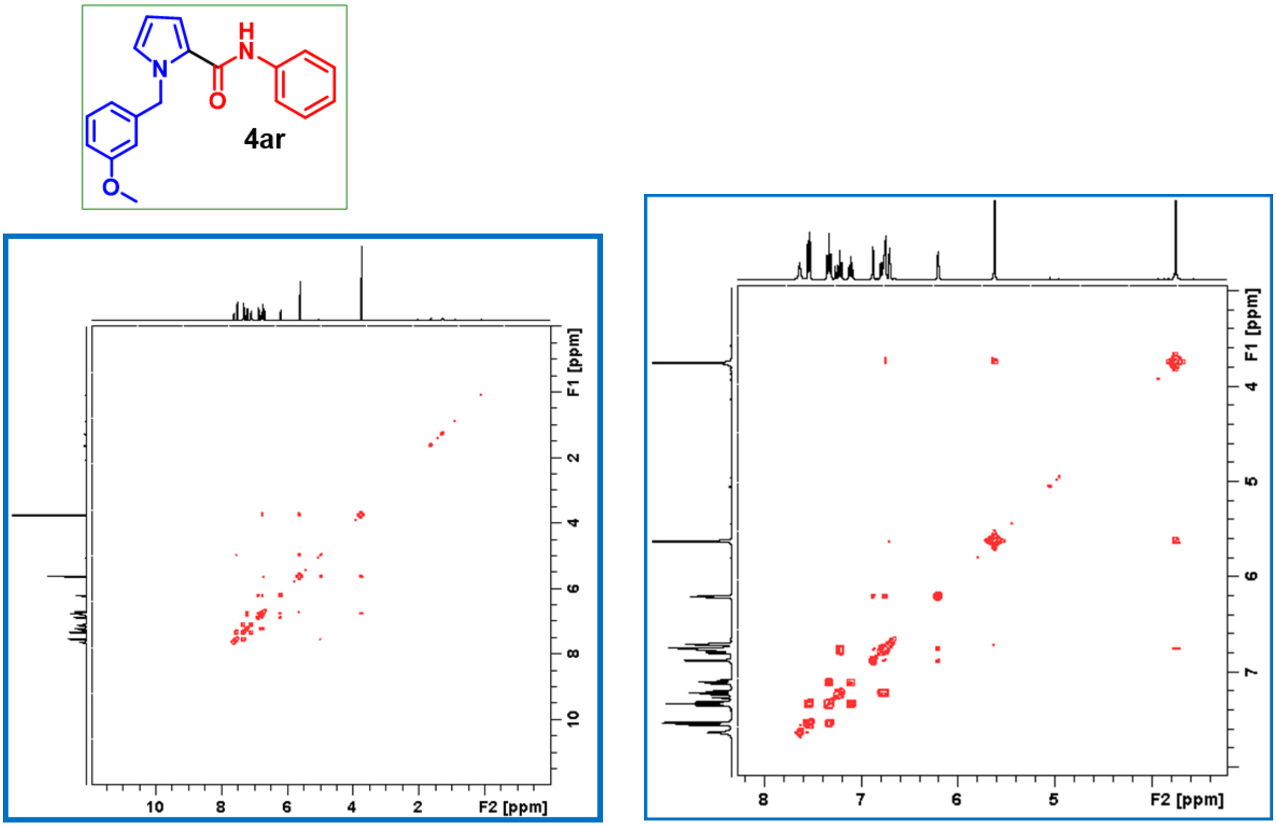

Figure S75. COSY NMR spectrum (400 MHz, $CDCl_3$, 298 K) of the derivative **4ar**, with expansion of significant portion of the spectrum in blue square.

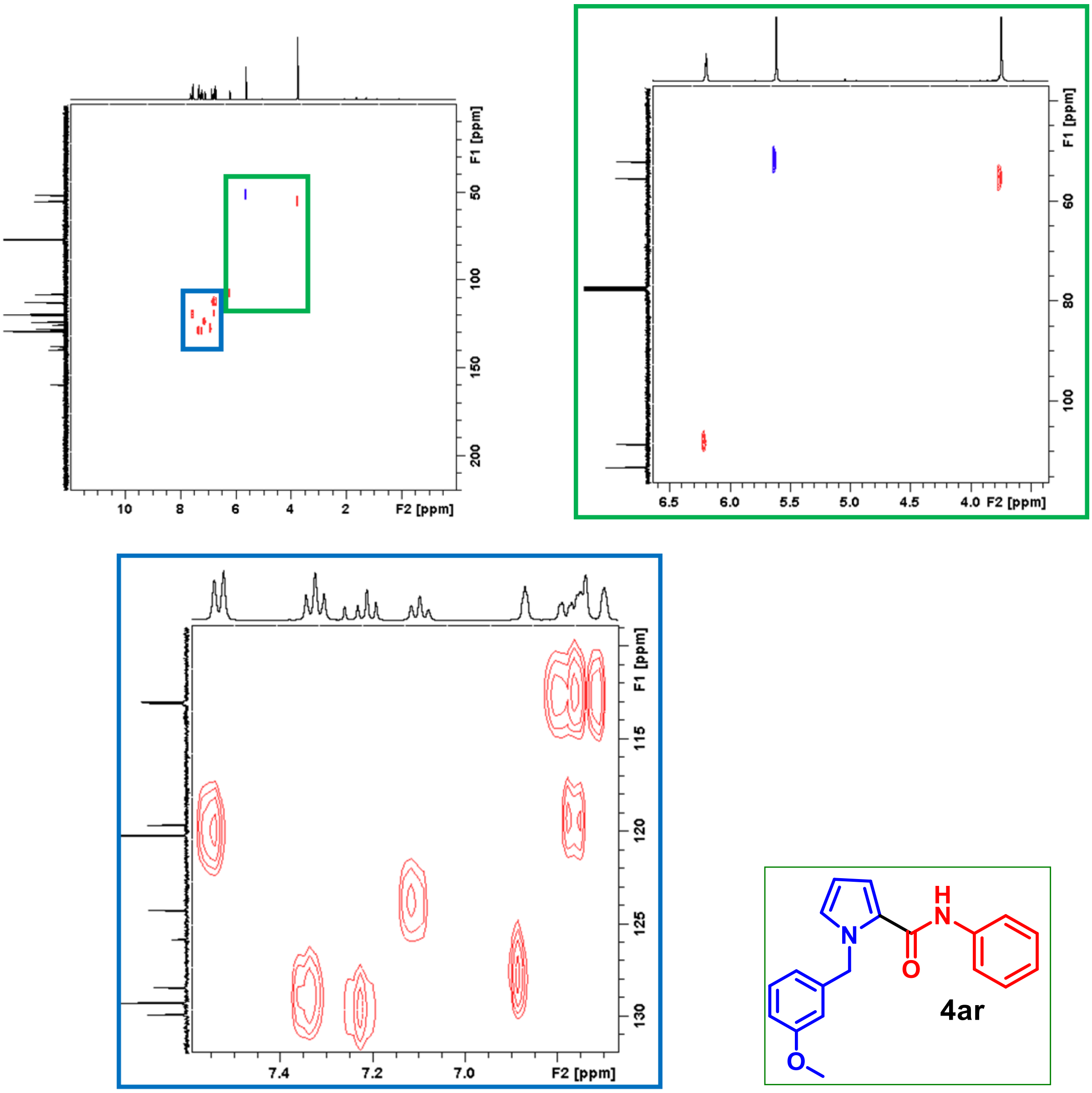

Figure S76. HSQC NMR spectrum (400 MHz, $CDCl_3$, 298 K) of the derivative **4ar**, with expansion of significant portion of the spectrum in blue square.

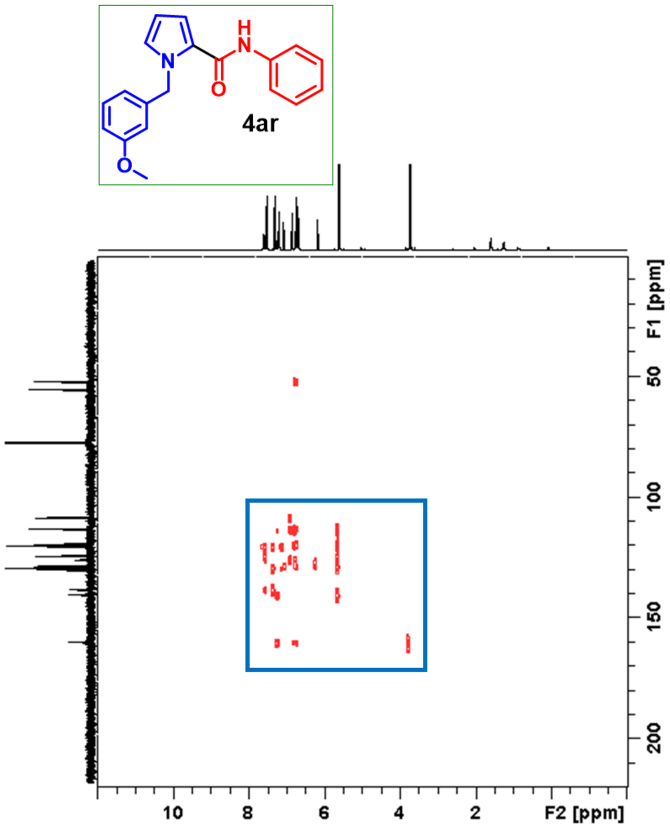

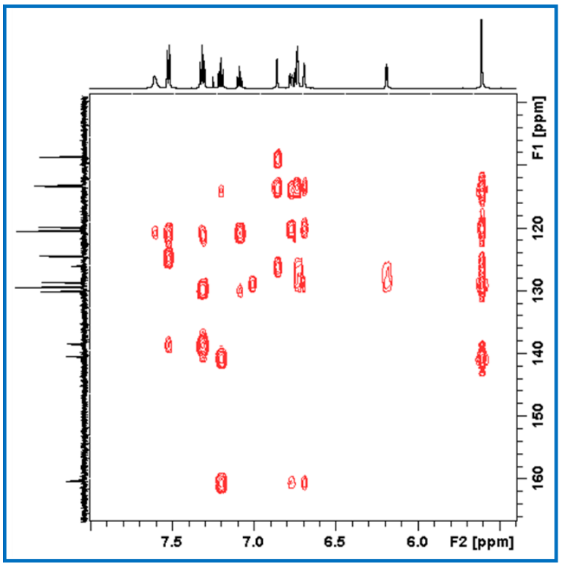

Figure S77. HMBC NMR spectrum (600 MHz, $CDCl_3$, 298 K) of the derivative **4ar**, with expansion of significant portion of the spectrum in blue square.

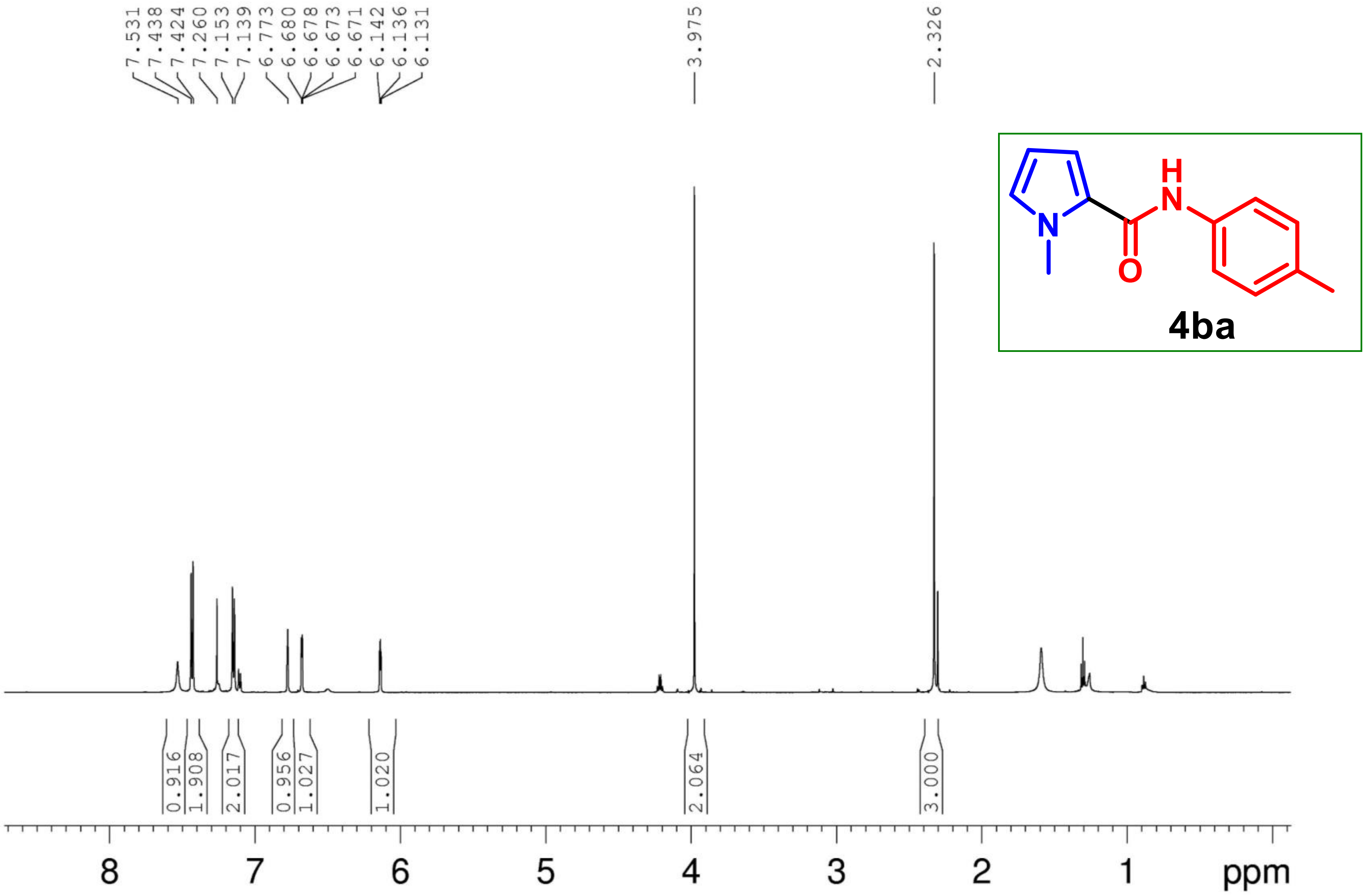

Figure S78. ¹H NMR spectrum (600 MHz, $CDCl_3$, 298 K) of the derivative **4ba**.

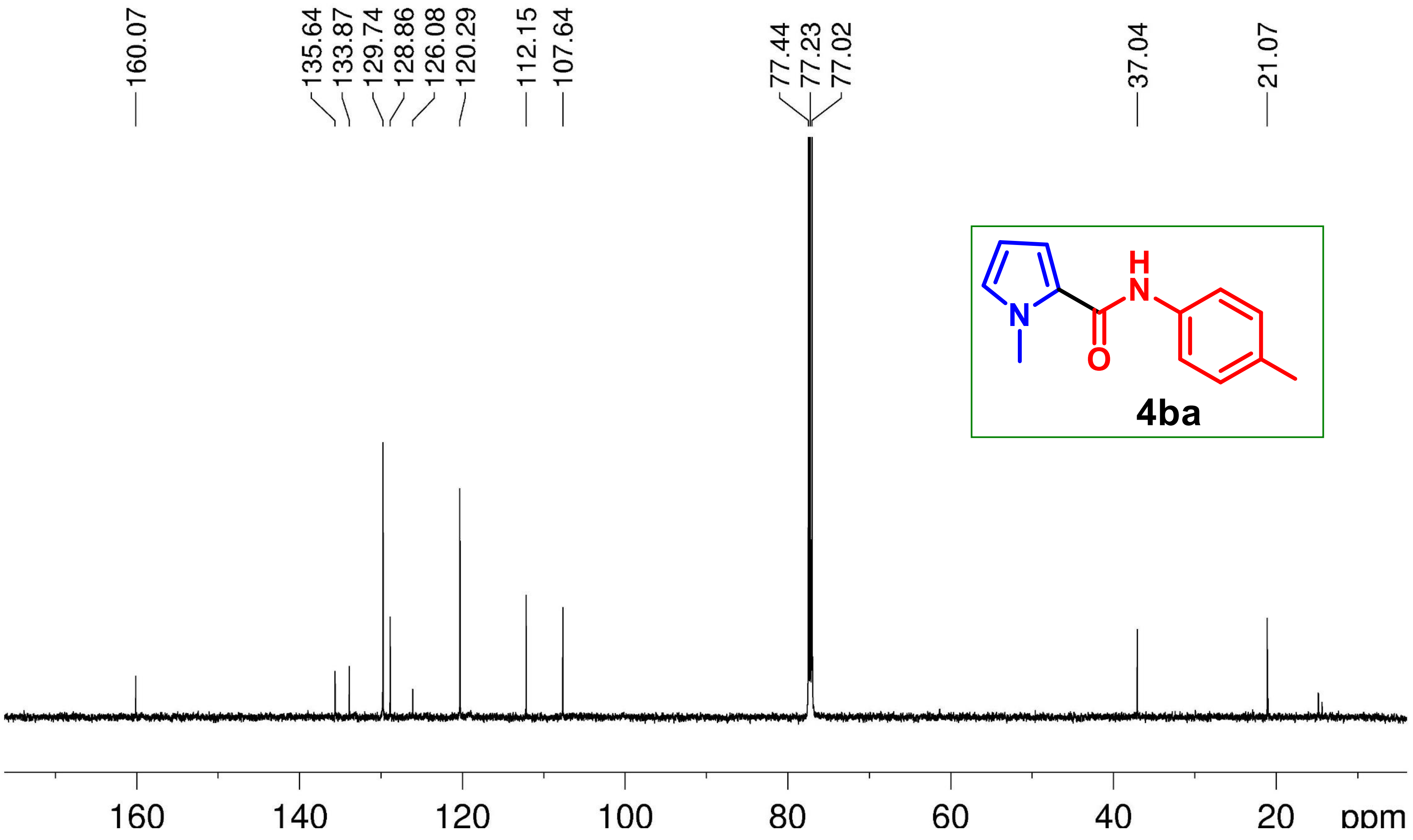

Figure S79. ^{13}C NMR spectrum (150 MHz, $CDCl_3$, 298 K) of the derivative **4ba**.

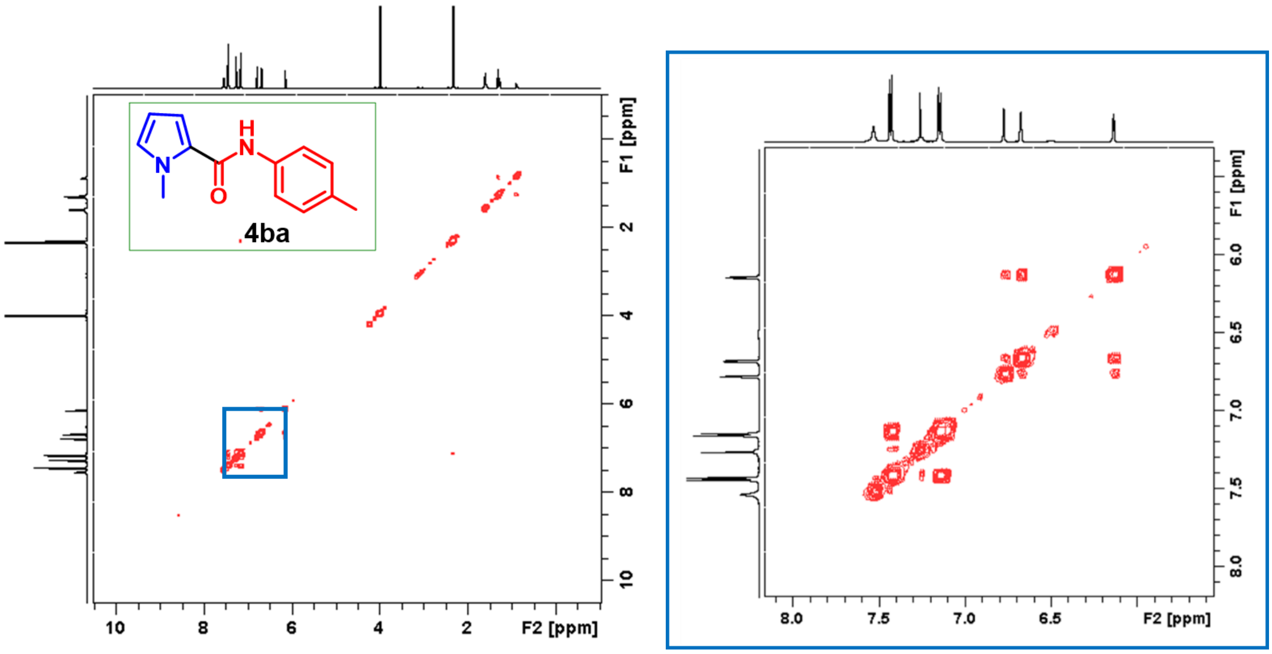

Figure S80. COSY NMR spectrum (600 MHz, $CDCl_3$, 298 K) of the derivative **4ba**, with expansion of significant portion of the spectrum in blue square.

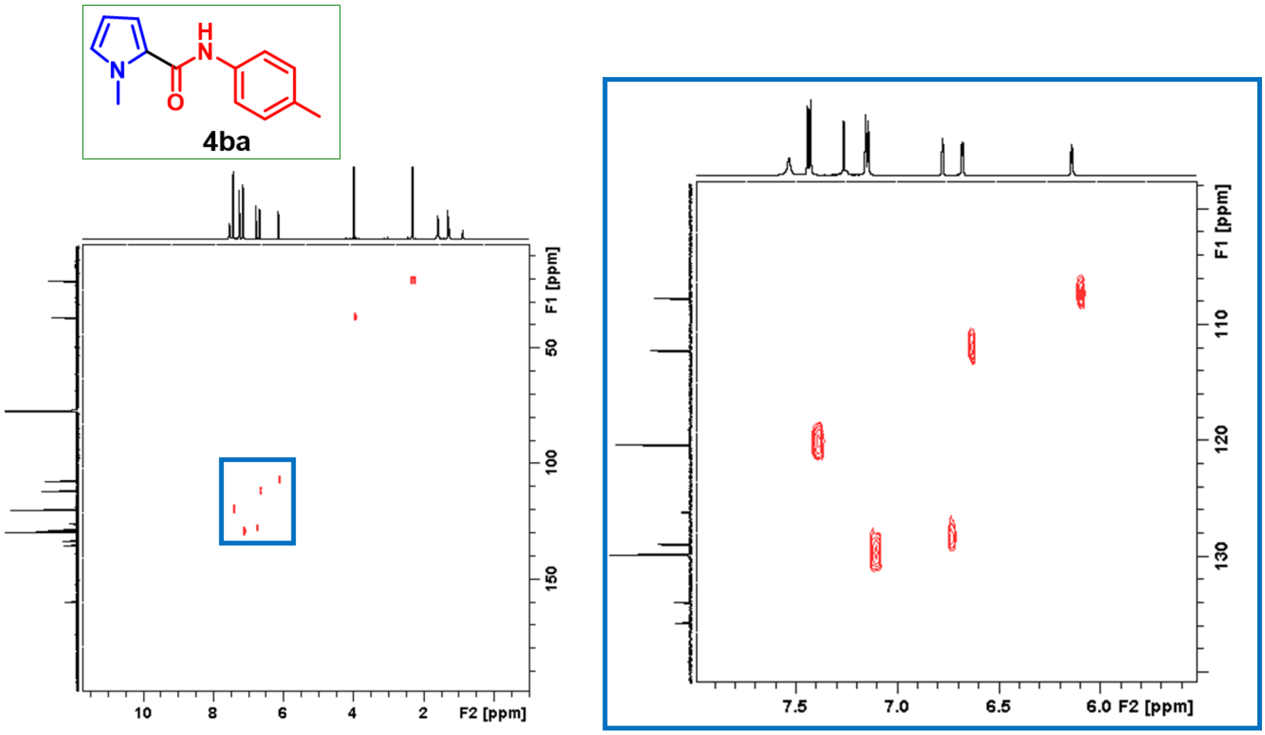

Figure S81. HSQC NMR spectrum (600 MHz, $CDCl_3$, 298 K) of the derivative **4ba**, with expansion of significant portion of the spectrum in blue square.

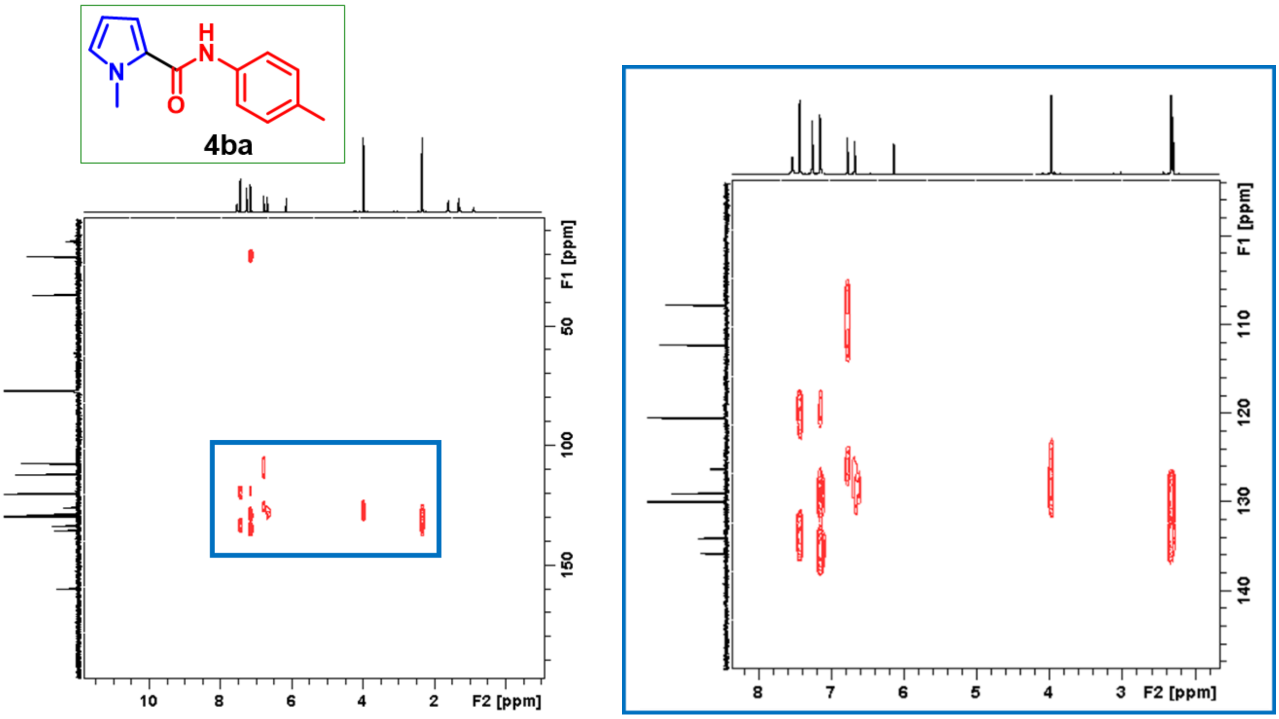

Figure S82. HMBC NMR spectrum (600 MHz, $CDCl_3$, 298 K) of the derivative **4ba**, with expansion of significant portion of the spectrum in blue square.

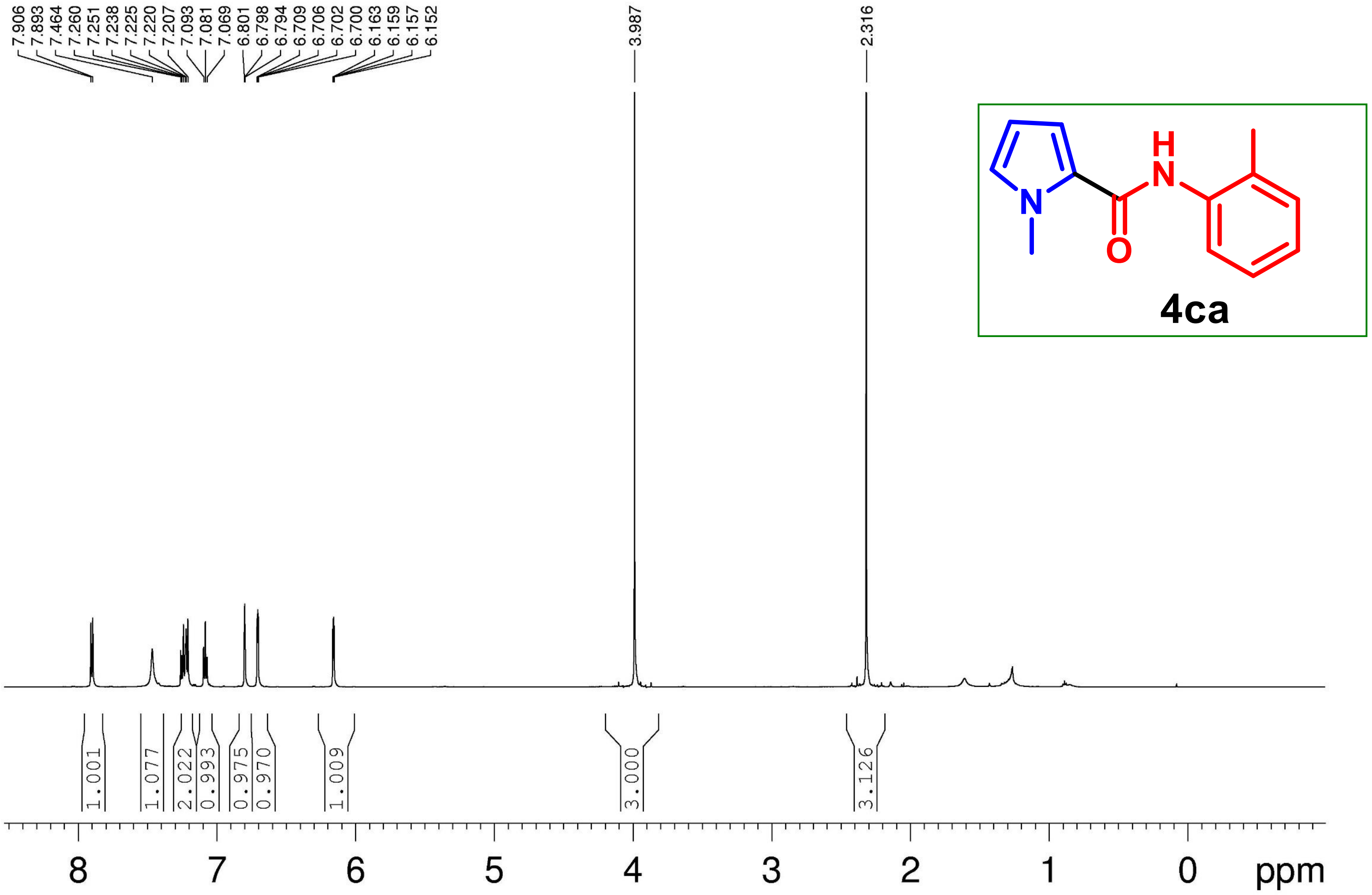

Figure S83. ^{1}H NMR spectrum (600 MHz, $CDCl_3$, 298 K) of the derivative **4ca**.

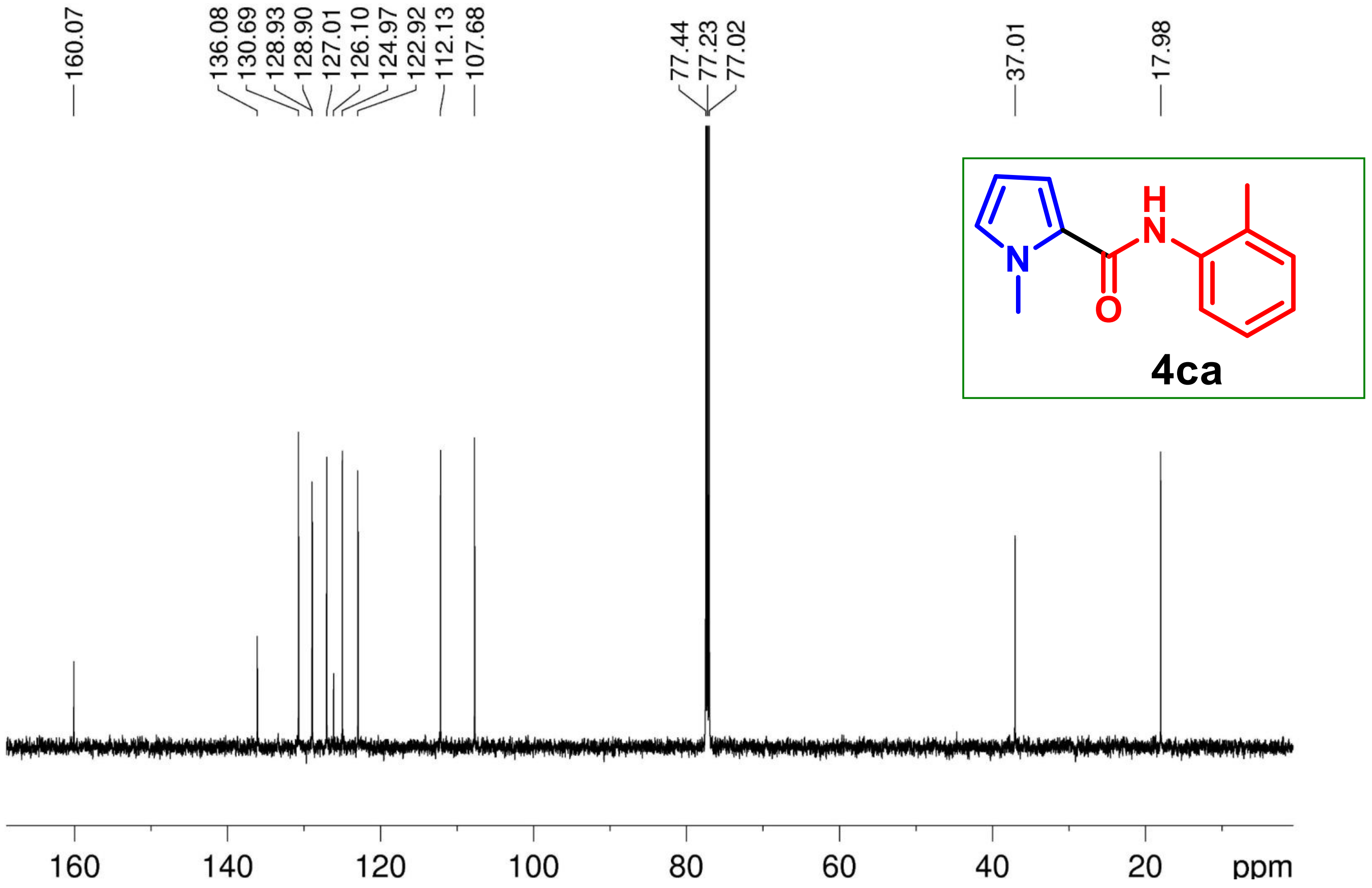

Figure S84. ^{13}C NMR spectrum (150 MHz, $CDCl_3$, 298 K) of the derivative **4ca**.

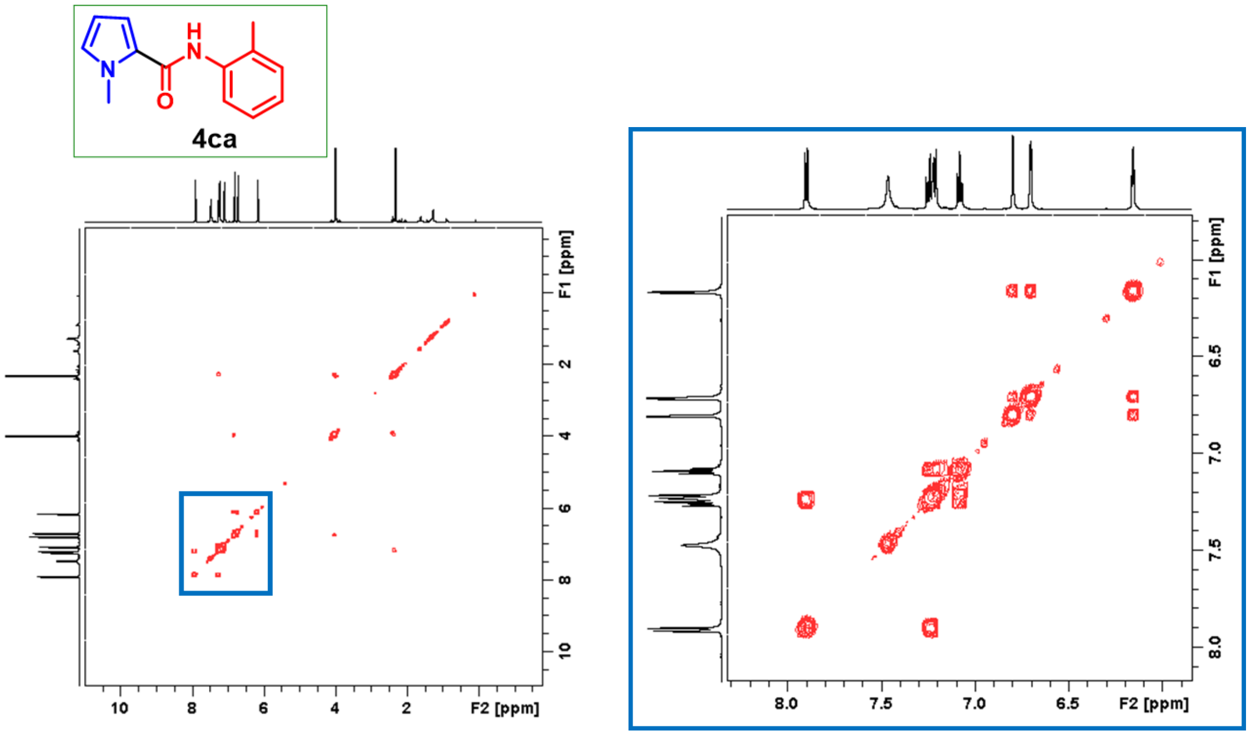

Figure S85. COSY NMR spectrum (600 MHz, $CDCl_3$, 298 K) of the derivative **4ca**, with expansion of significant portion of the spectrum in blue square.

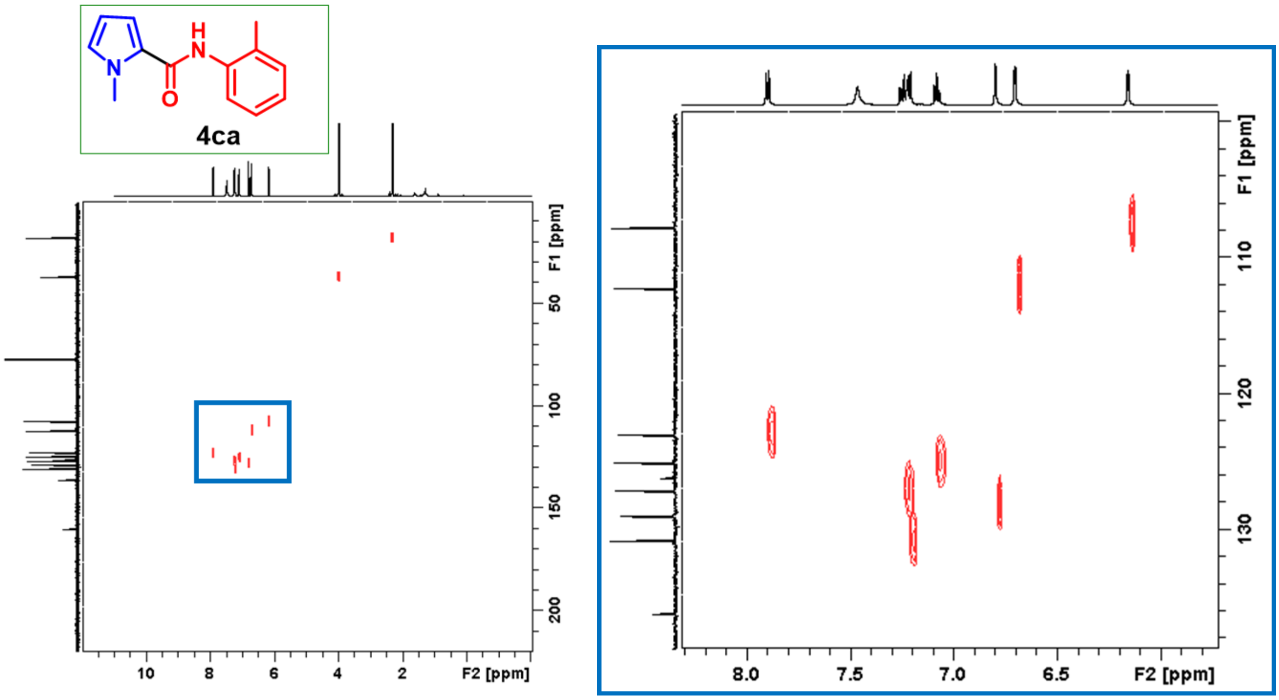

Figure S86. HSQC NMR spectrum (600 MHz, $CDCl_3$, 298 K) of the derivative **4ca**, with expansion of significant portion of the spectrum in blue square.

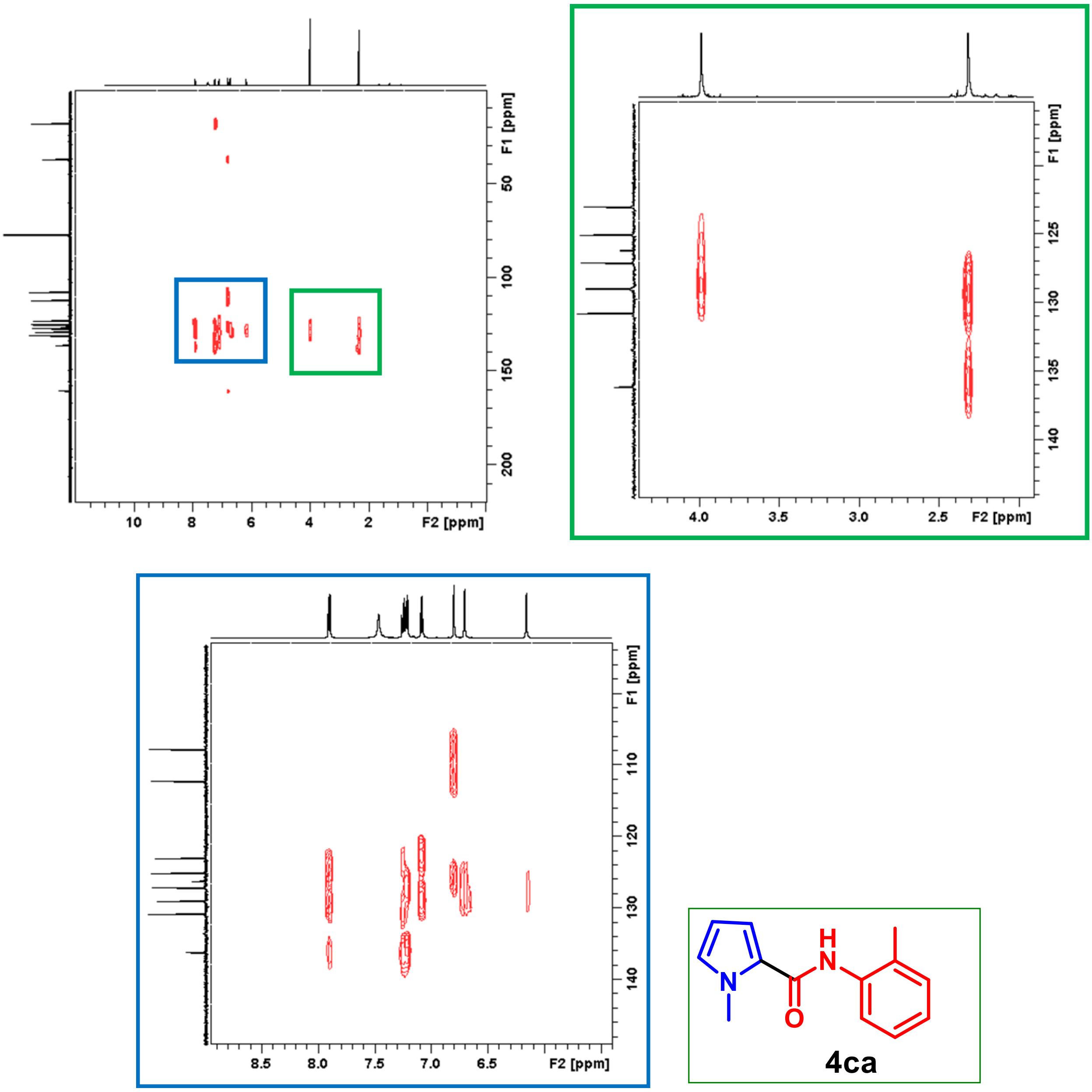

Figure S87. HMBC NMR spectrum (600 MHz, $CDCl_3$, 298 K) of the derivative **4ca**, with expansions of significant portions of the spectrum in blue and green squares.

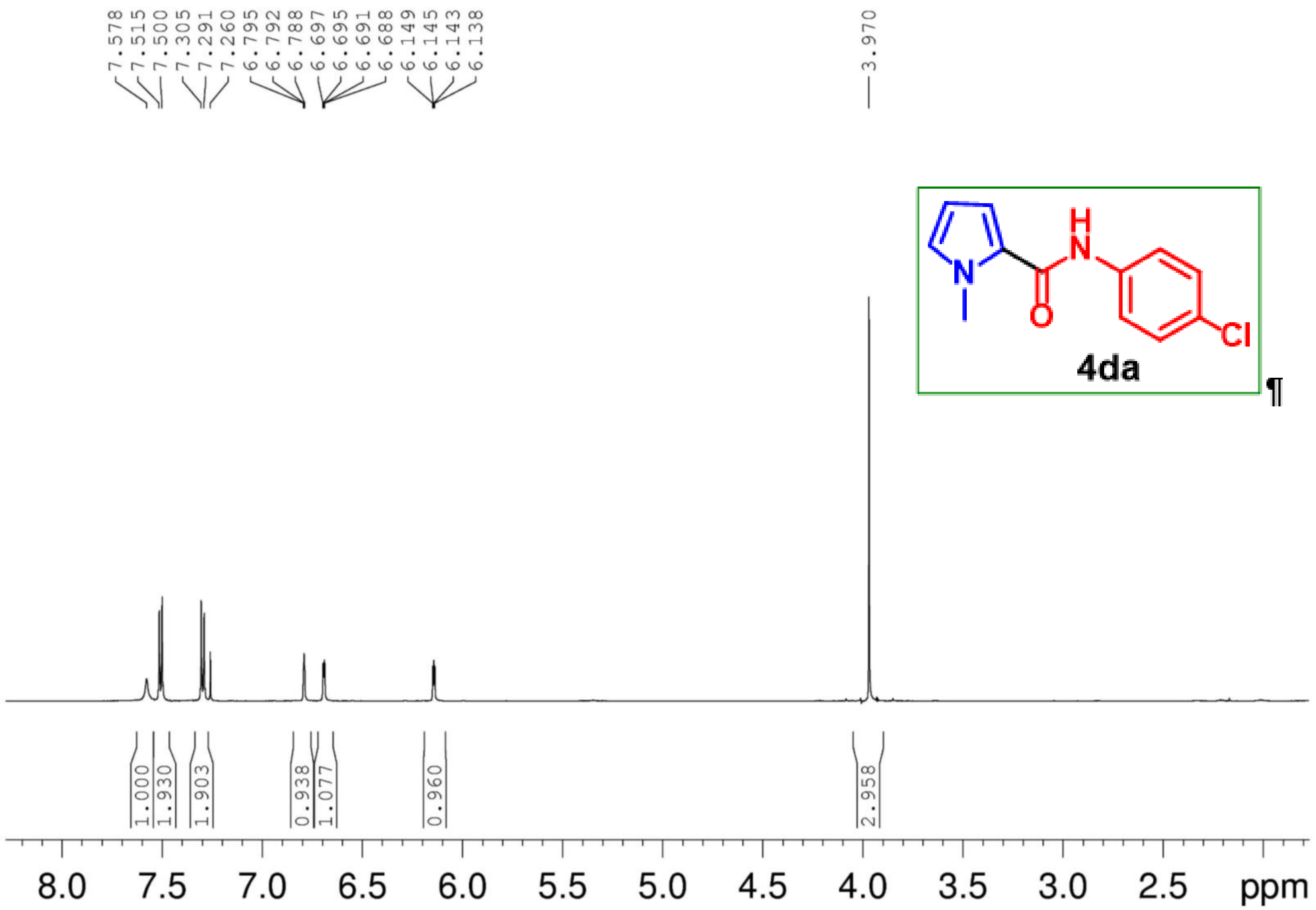

Figure S88. ^{1}H NMR spectrum (600 MHz, $CDCl_3$, 298 K) of the derivative **4da**.

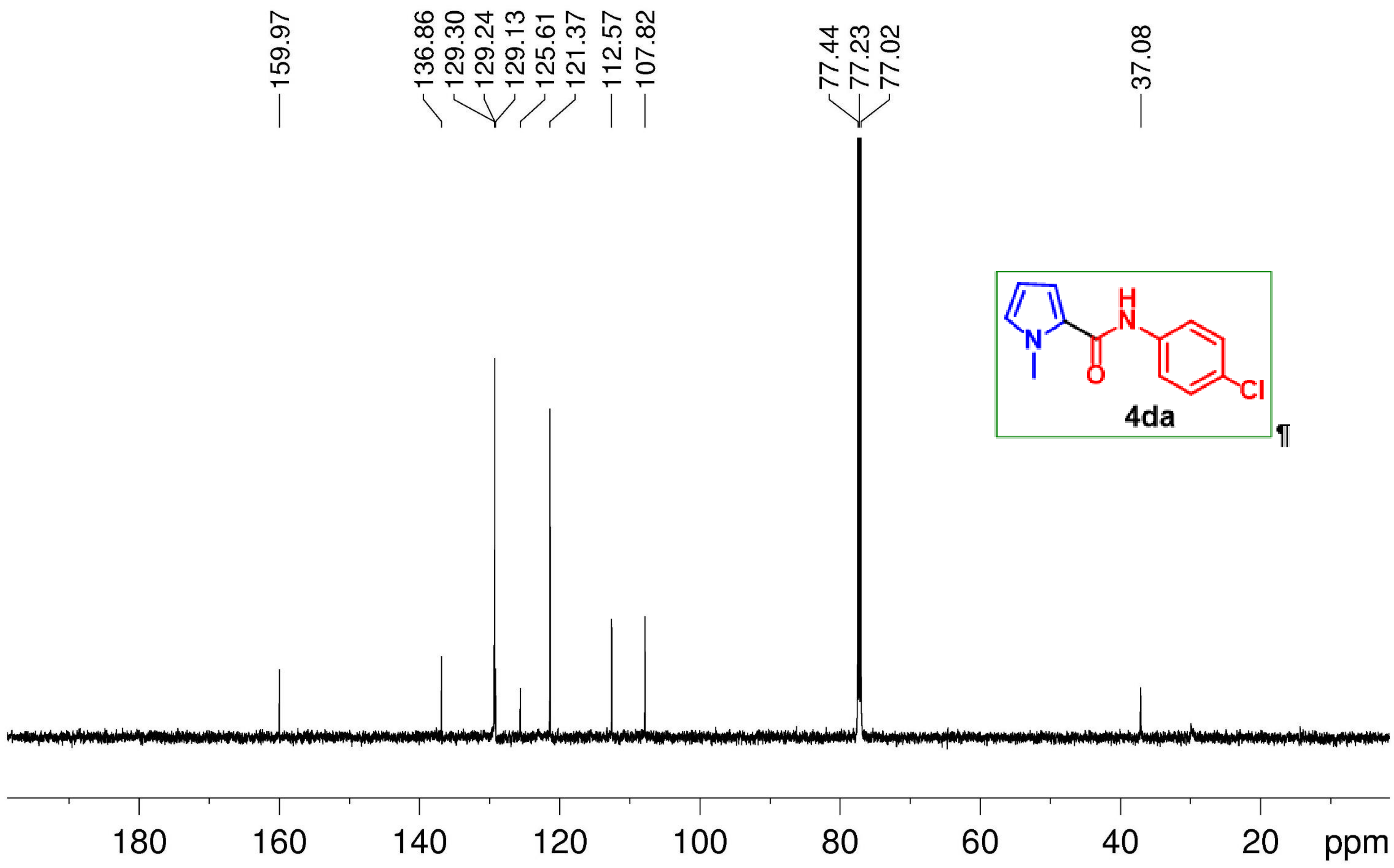

Figure S89. ^{13}C NMR spectrum (150 MHz, $CDCl_3$, 298 K) of the derivative **4da**.

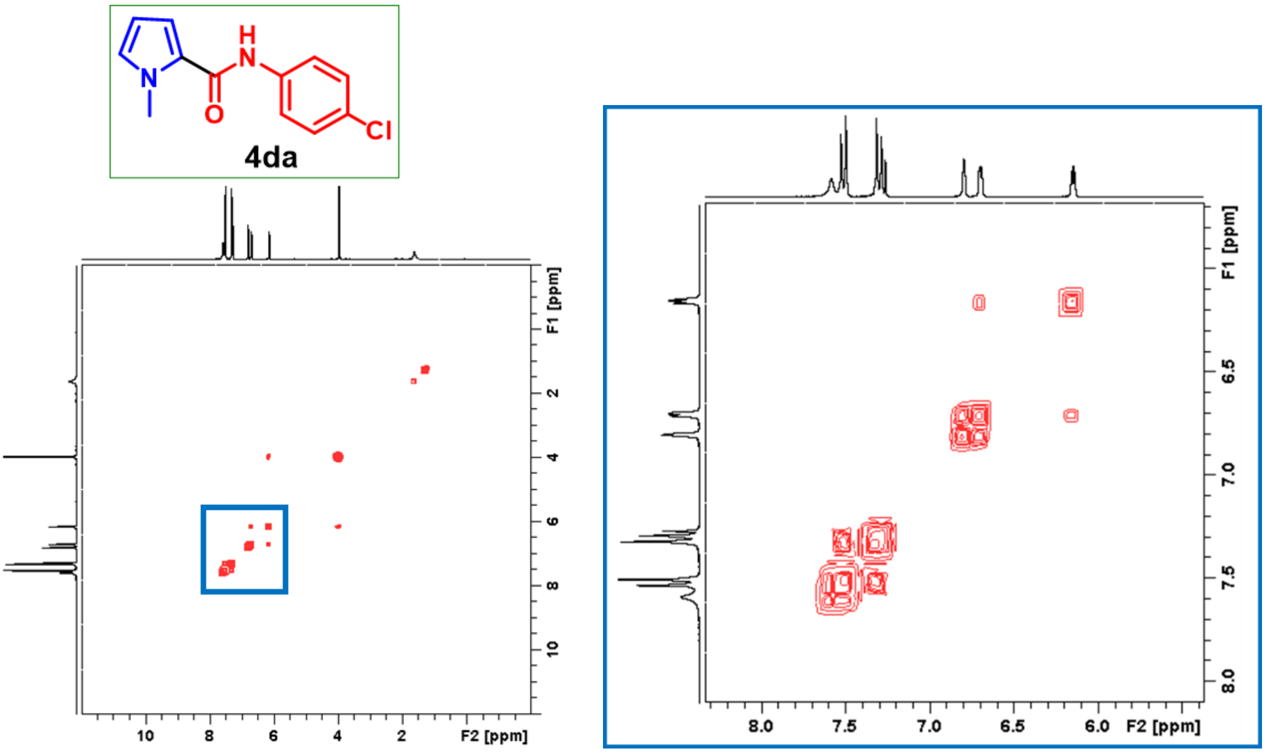

Figure S90. COSY NMR spectrum (300 MHz, $CDCl_3$, 298 K) of the derivative **4da**, with expansion of significant portion of the spectrum in blue square.

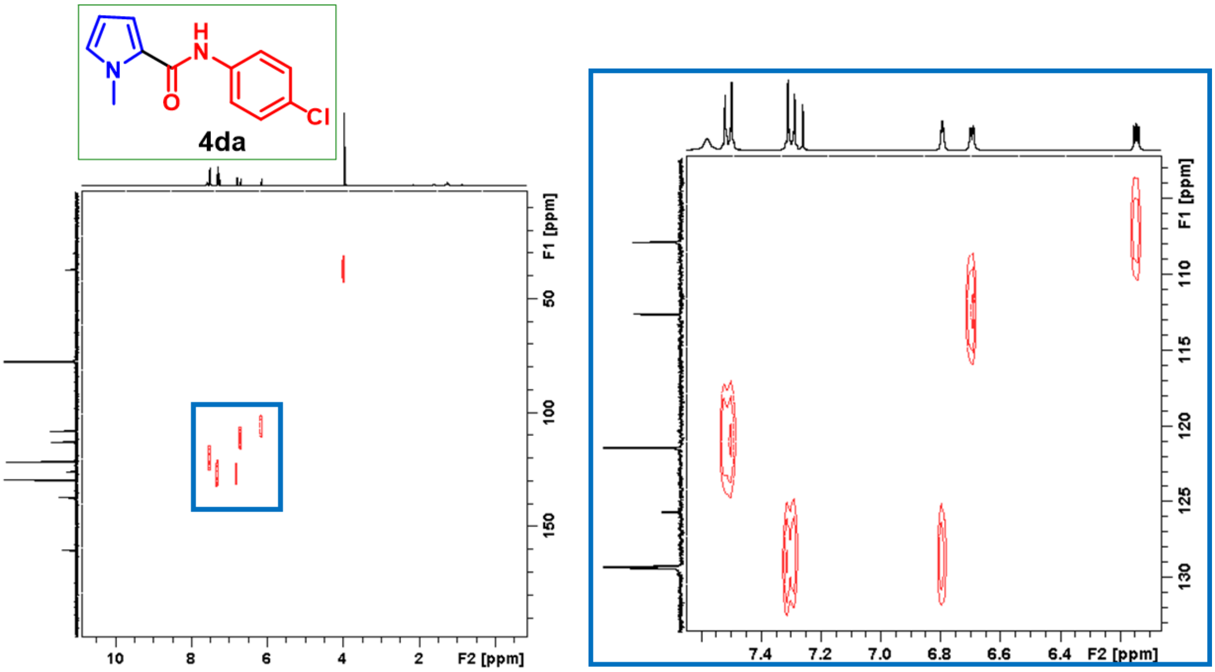

Figure S91. HSQC NMR spectrum (400 MHz, $CDCl_3$, 298 K) of the derivative **4da**, with expansion of significant portion of the spectrum in blue square.

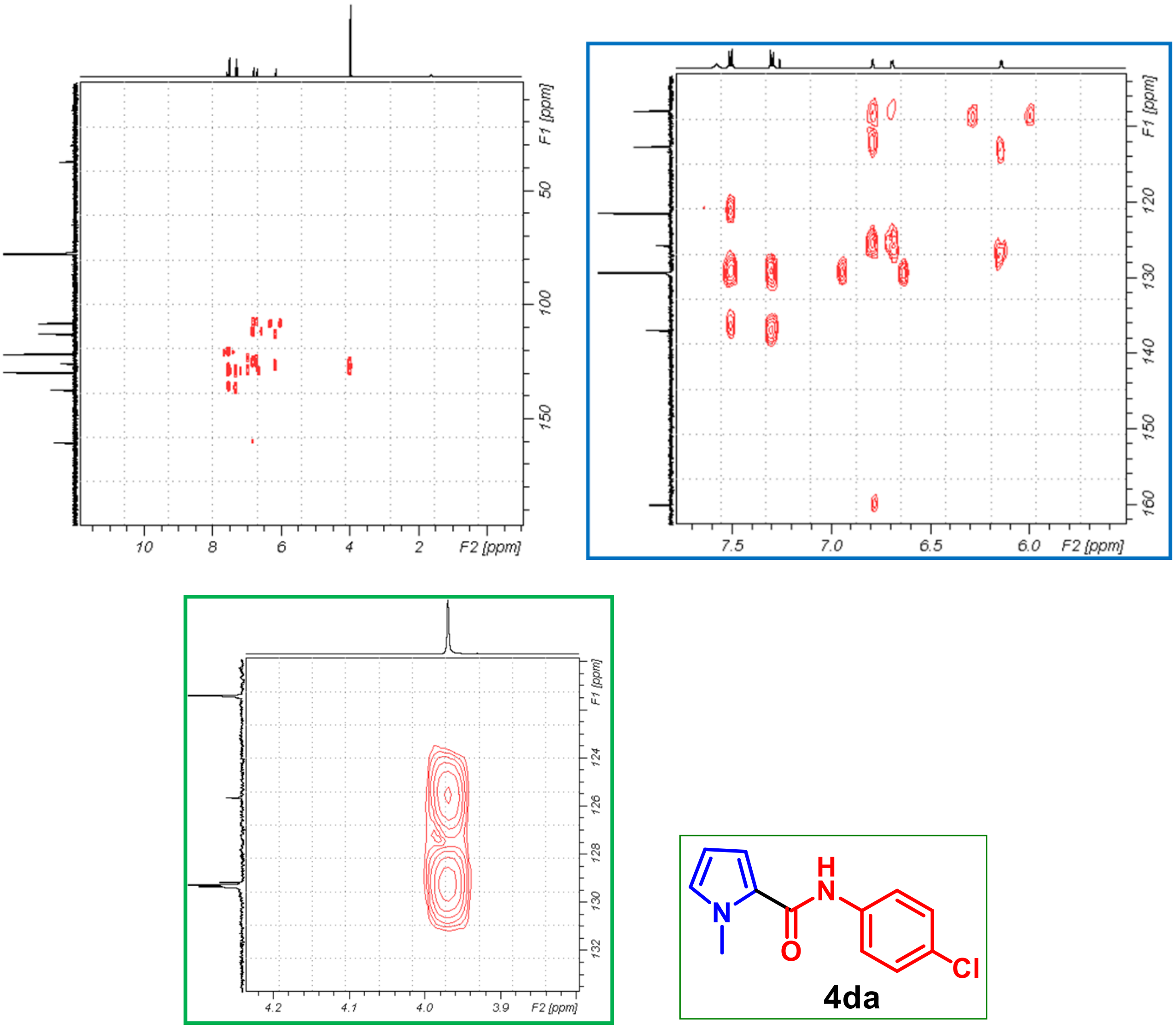

Figure S92. HMBC NMR spectrum (600 MHz, $CDCl_3$, 298 K) of the derivative **4da**, with expansions of significant portions of the spectrum in blue and green squares.

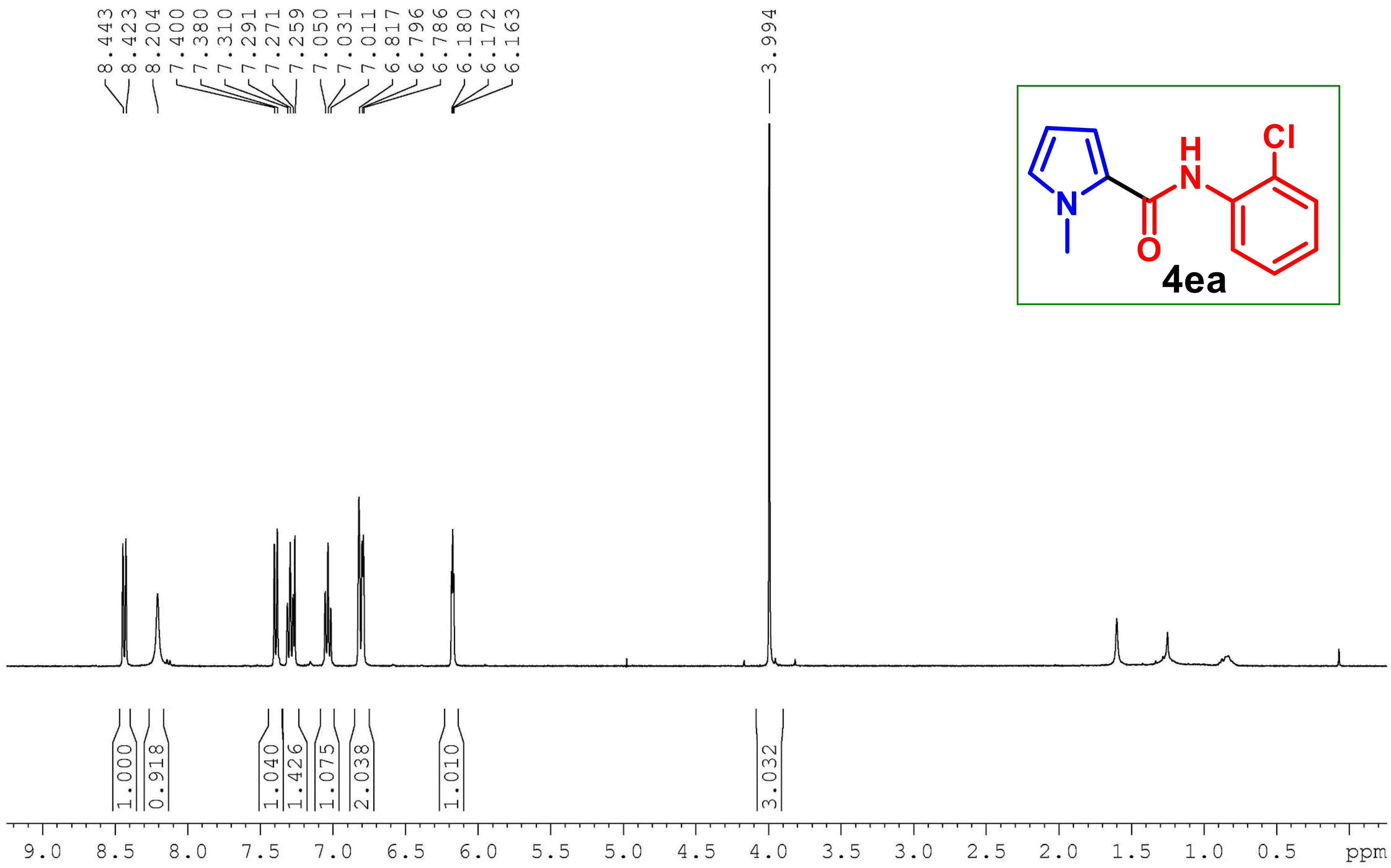

Figure S93. ^{1}H NMR spectrum (400 MHz, $CDCl_3$, 298 K) of the derivative **4ea**.

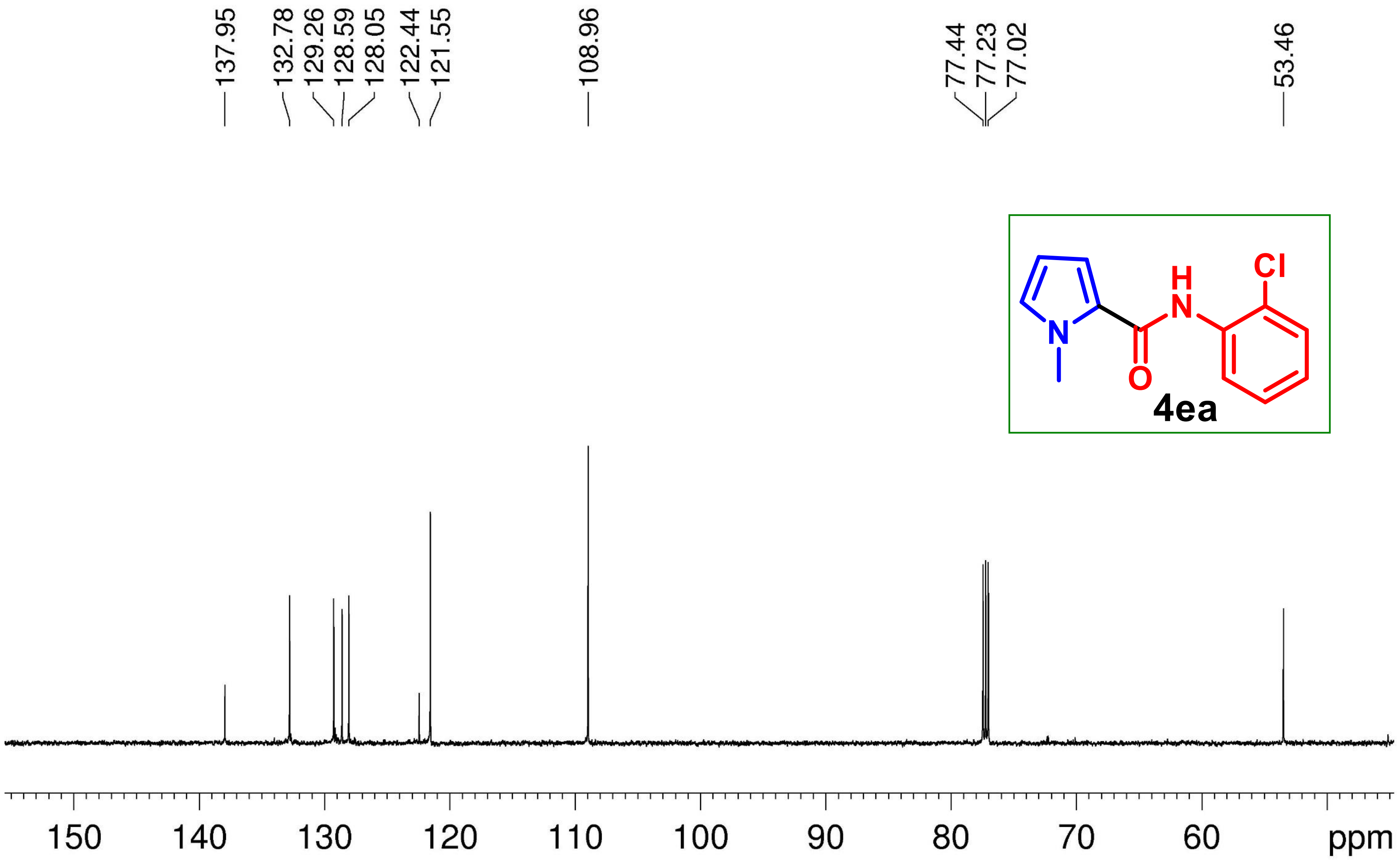

Figure S94. ^{13}C NMR spectrum (150 MHz, $CDCl_3$, 298 K) of the derivative **4ea**.

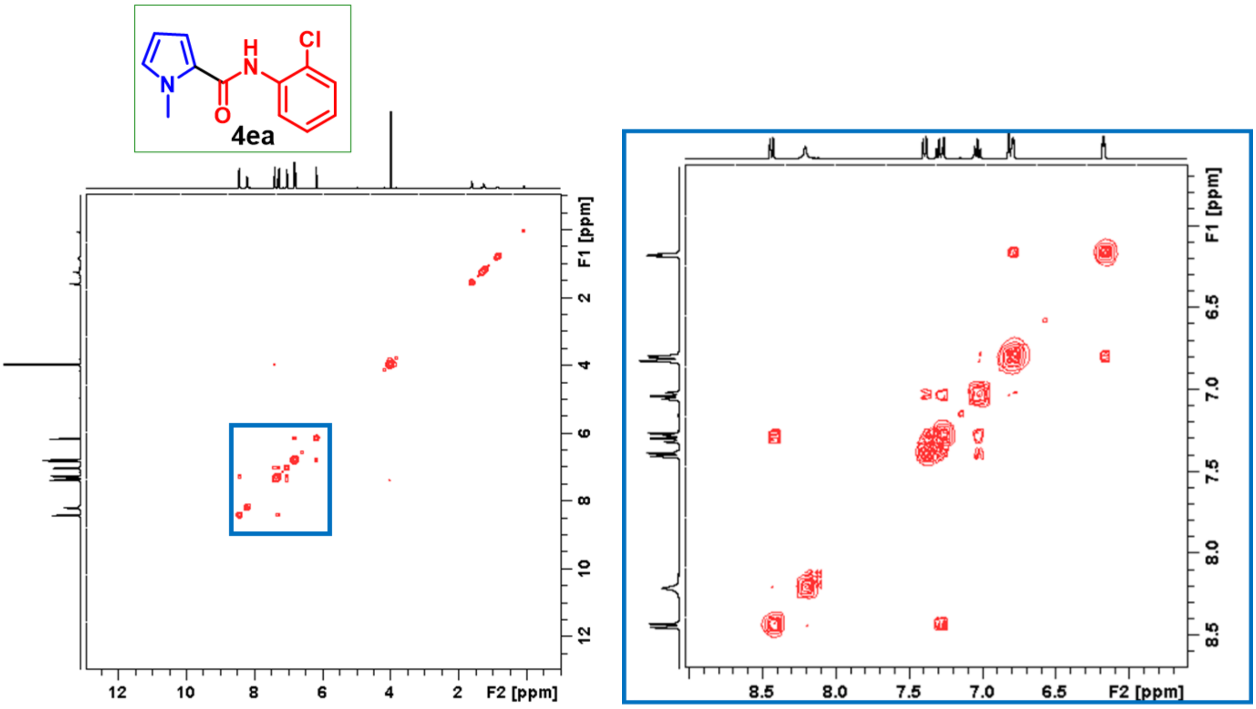

Figure S95. COSY NMR spectrum (600 MHz, $CDCl_3$, 298 K) of the derivative **4ea**, with expansion of significant portion of the spectrum in blue square.

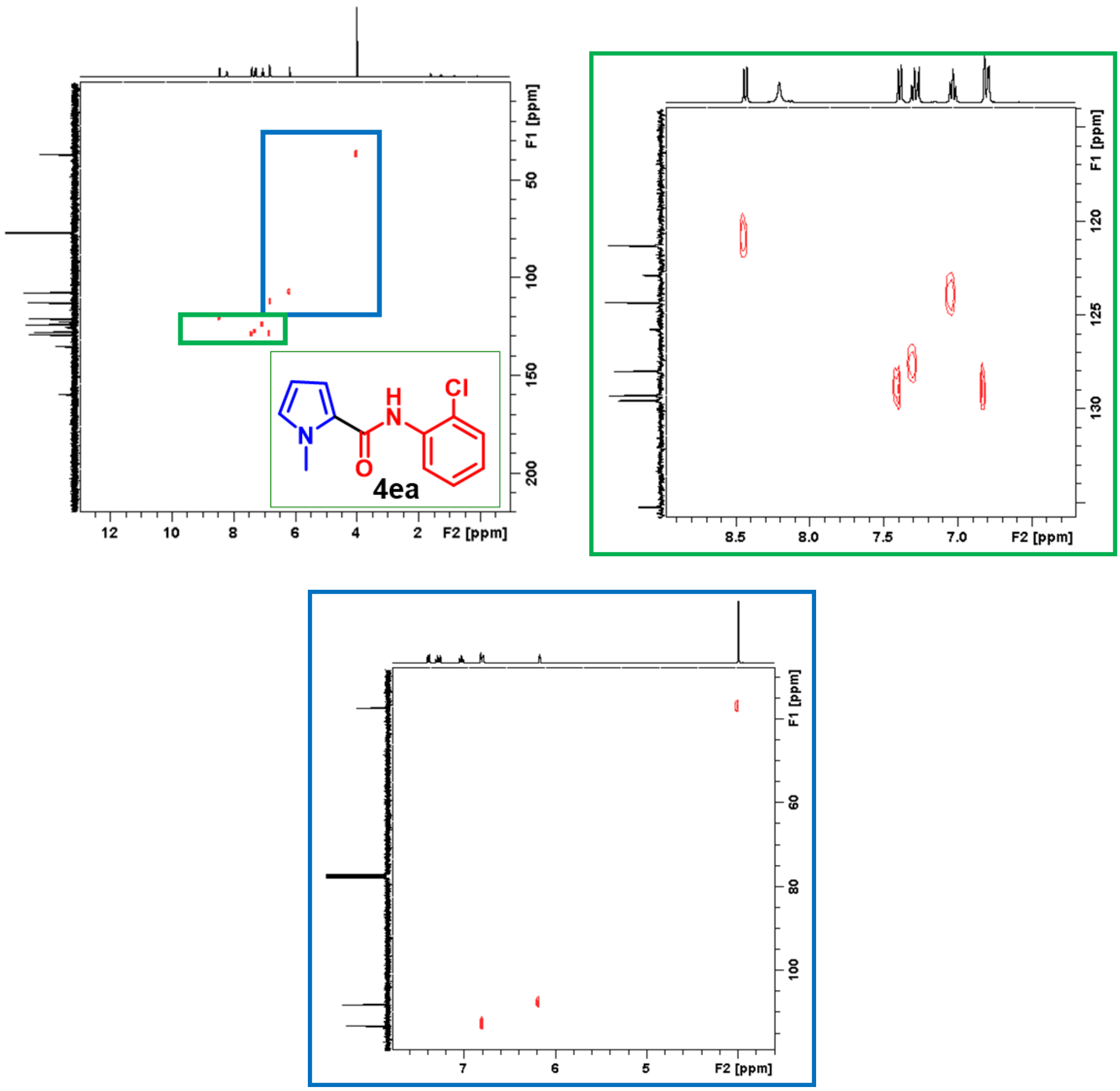

Figure S96. HSQC NMR spectrum (600 MHz, $CDCl_3$, 298 K) of the derivative **4ea**, with expansions of significant portions of the spectrum in blue and green squares.

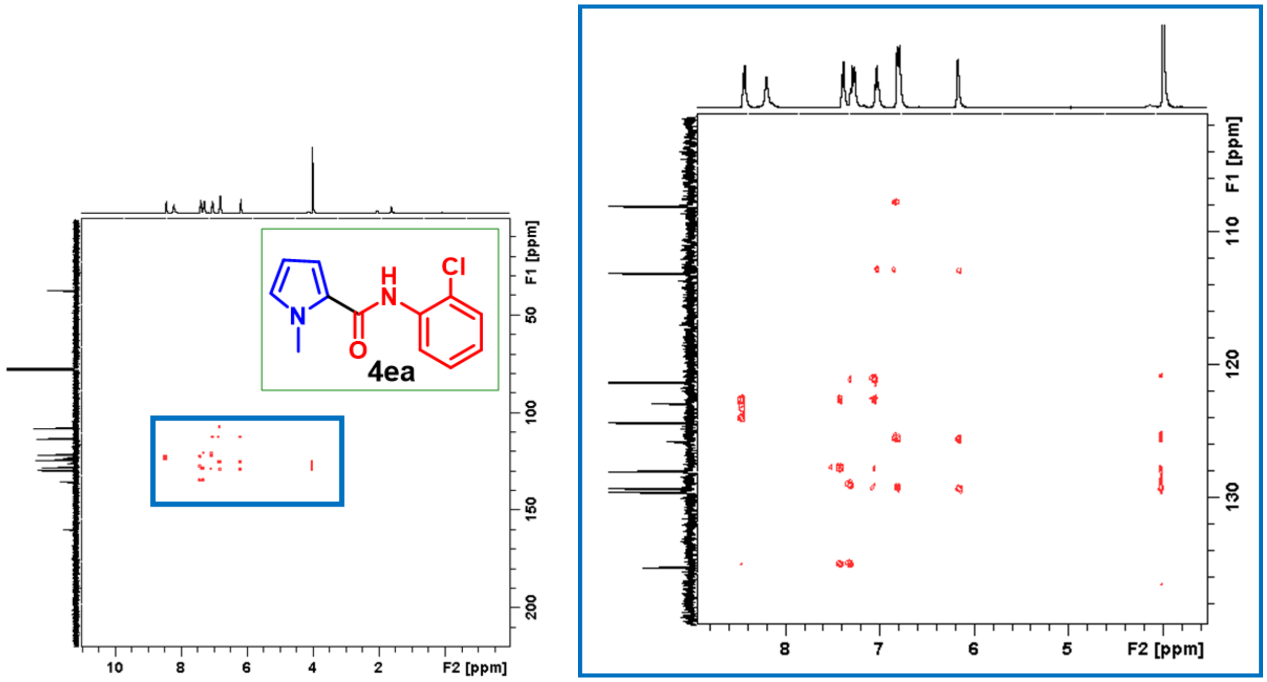

Figure S97. HMBC NMR spectrum (600 MHz, $CDCl_3$, 298 K) of the derivative **4ea**, with expansion of significant portion of the spectrum in blue square.

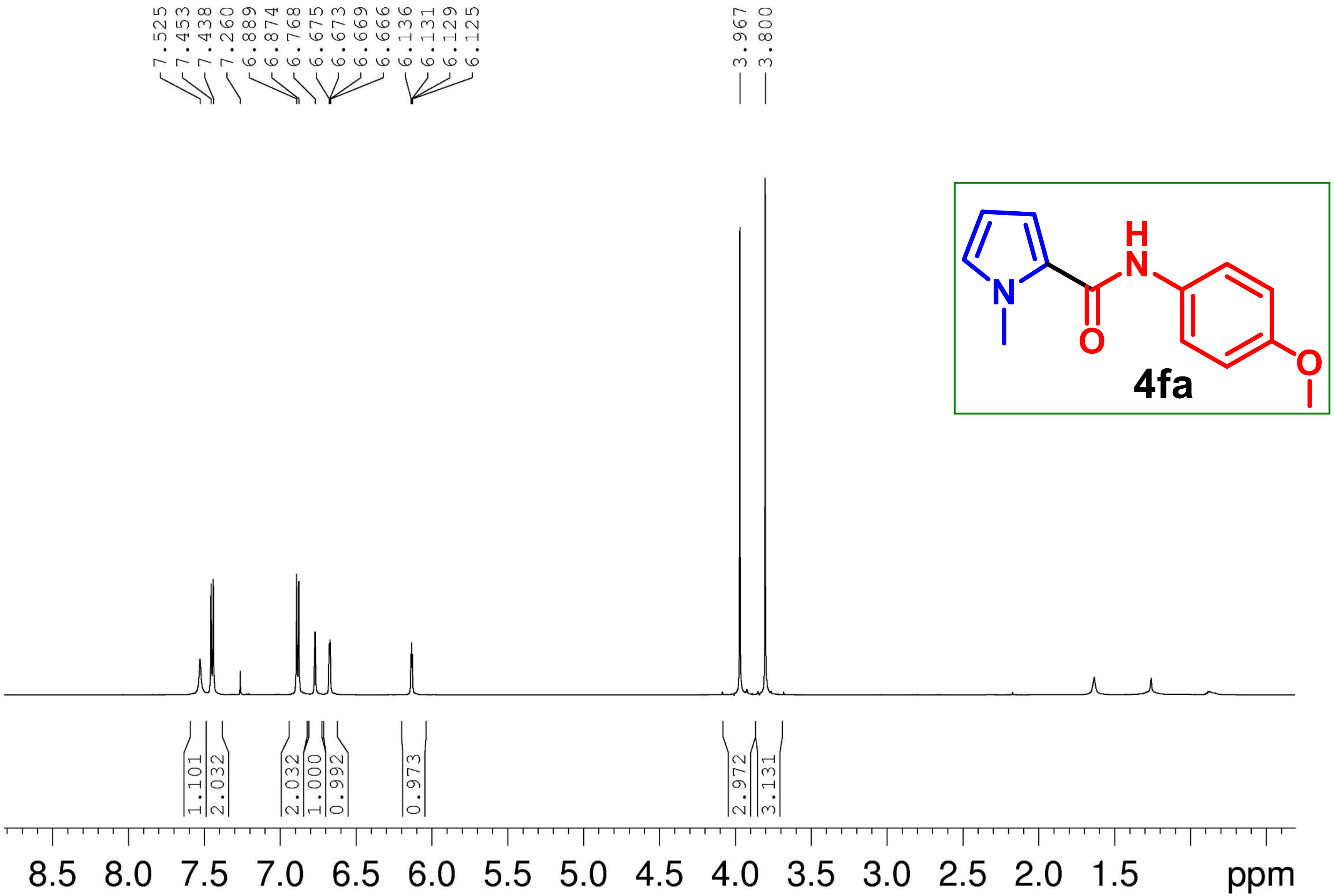

Figure S98. ^{1}H NMR spectrum (600 MHz, $CDCl_3$, 298 K) of the derivative **4fa**.

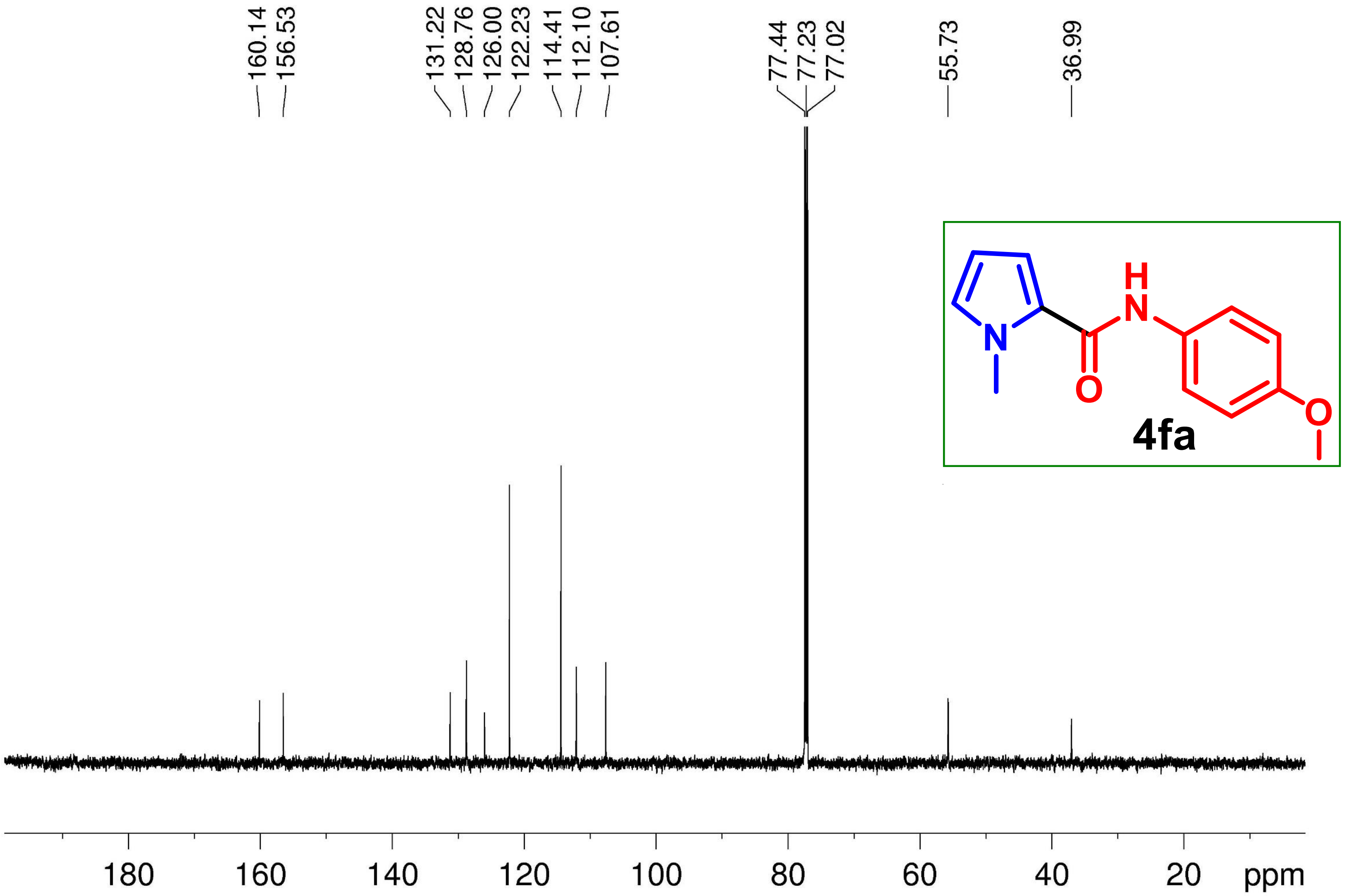

Figure S99. ^{13}C NMR spectrum (150 MHz, $CDCl_3$, 298 K) of the derivative **4fa**.

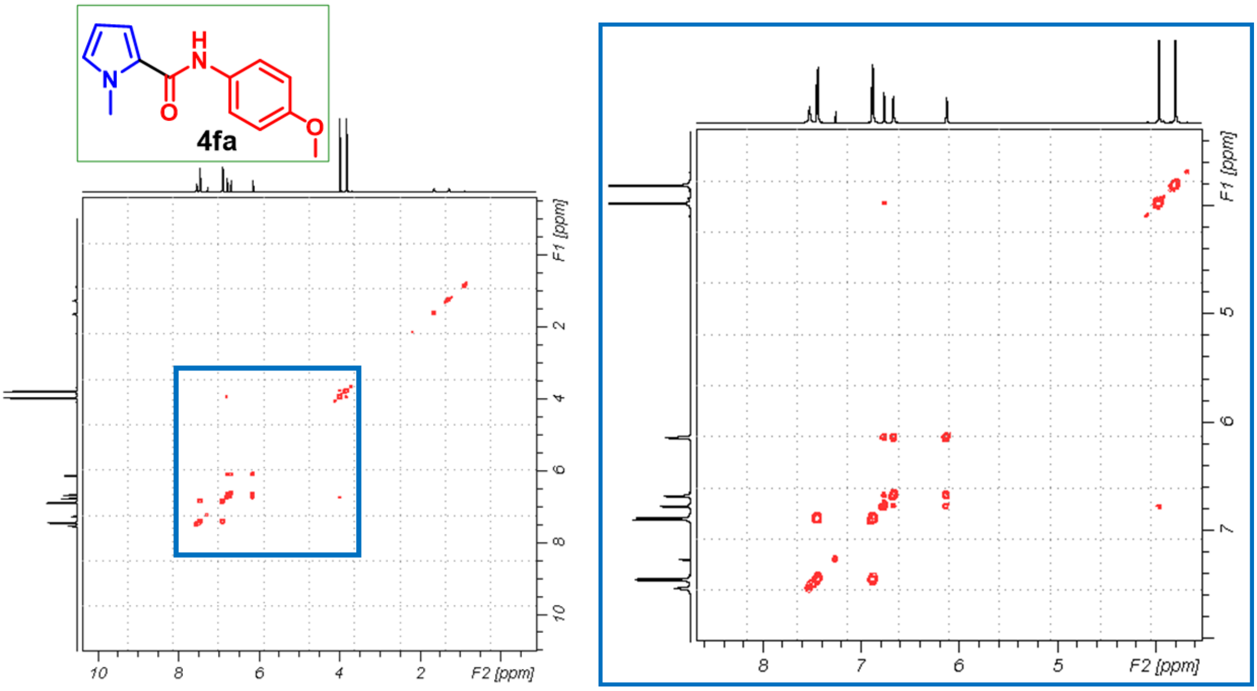

Figure S100. COSY NMR spectrum (600 MHz, $CDCl_3$, 298 K) of the derivative **4fa**, with expansion of significant portion of the spectrum in blue square.

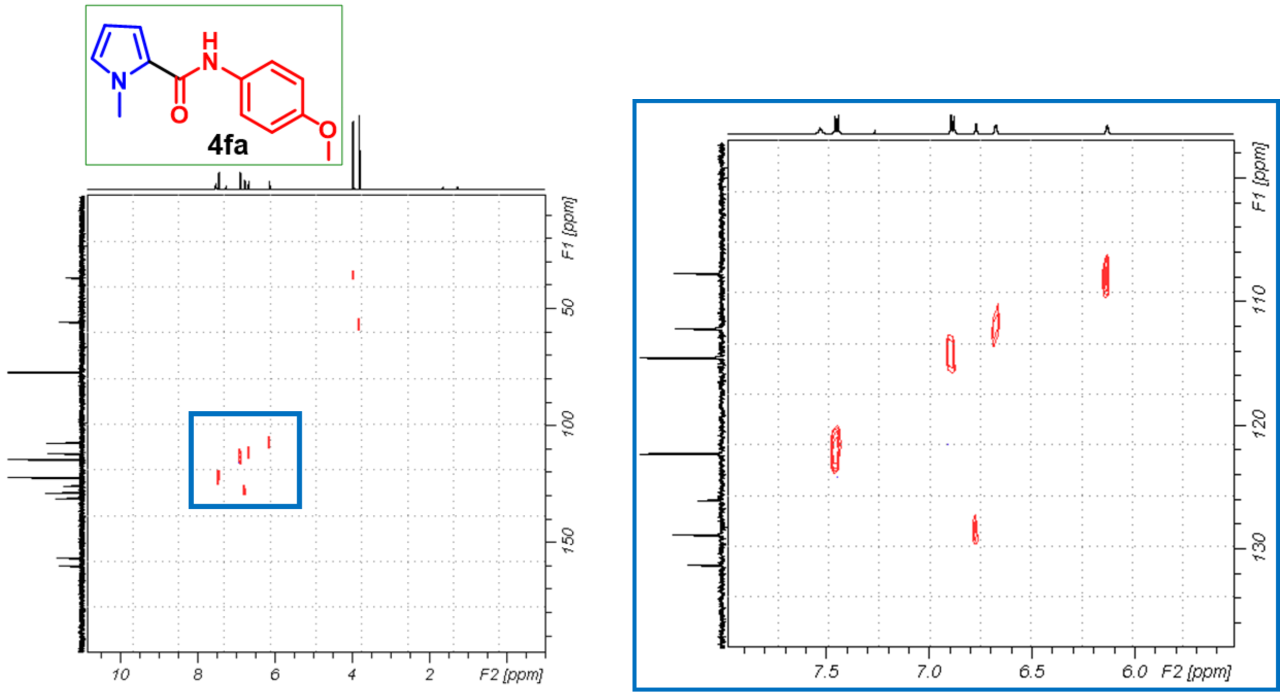

Figure S101. HSQC NMR spectrum (600 MHz, $CDCl_3$, 298 K) of the derivative **4fa**, with expansion of significant portion of the spectrum in blue square.

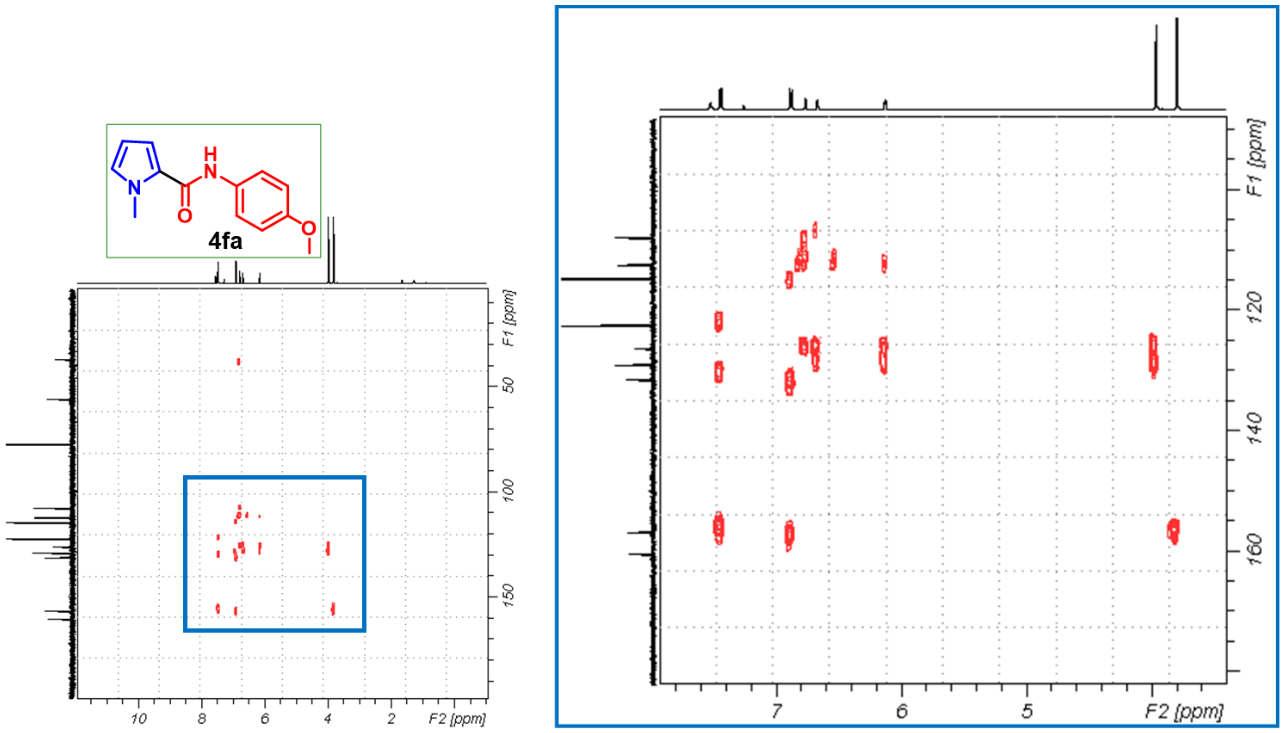

Figure S102. HMBC NMR spectrum (600 MHz, $CDCl_3$, 298 K) of the derivative **4fa**, with expansion of significant portion of the spectrum in blue square.

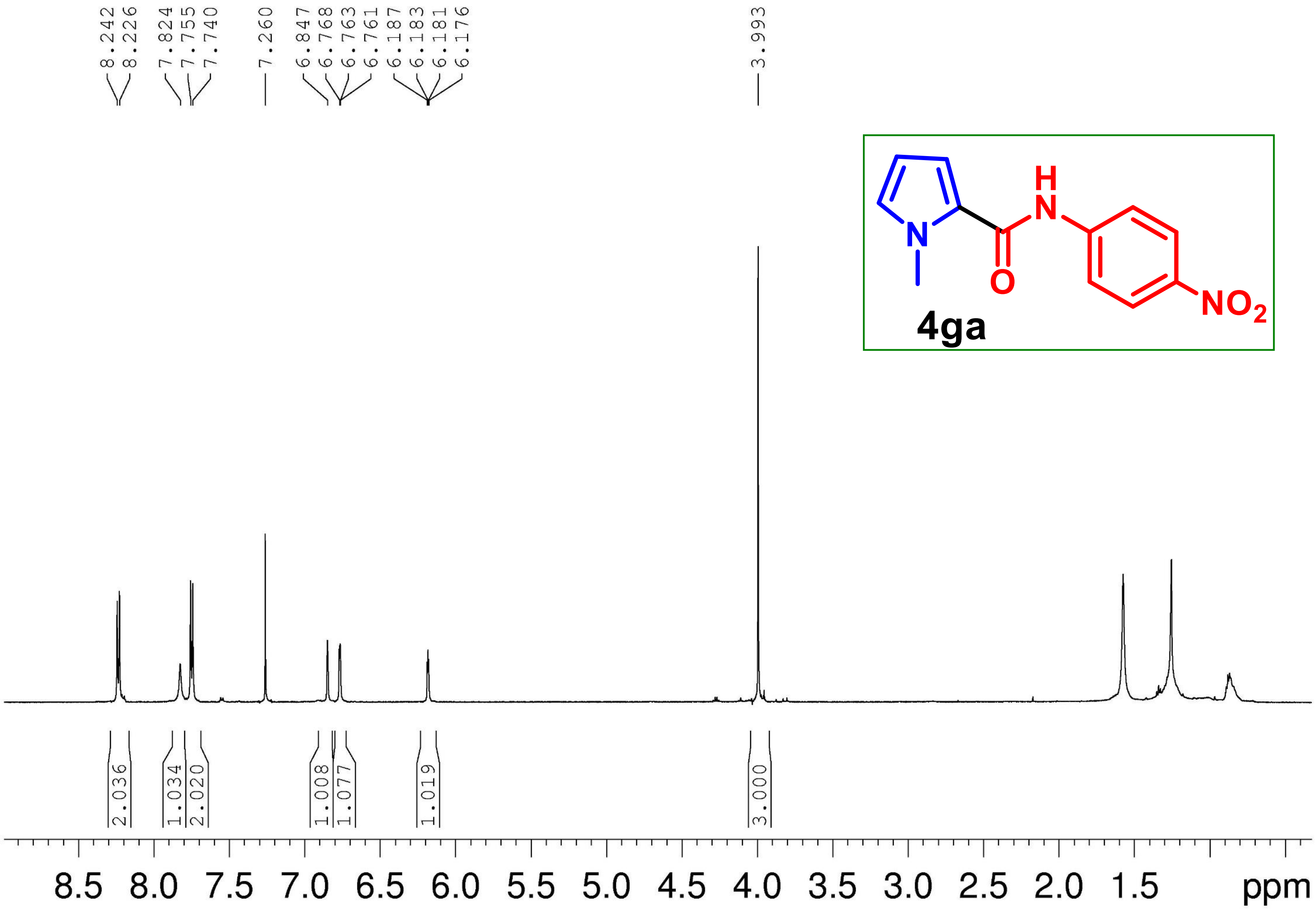

Figure S103. ^{1}H NMR spectrum (600 MHz, $CDCl_3$, 298 K) of the derivative **4ga**.

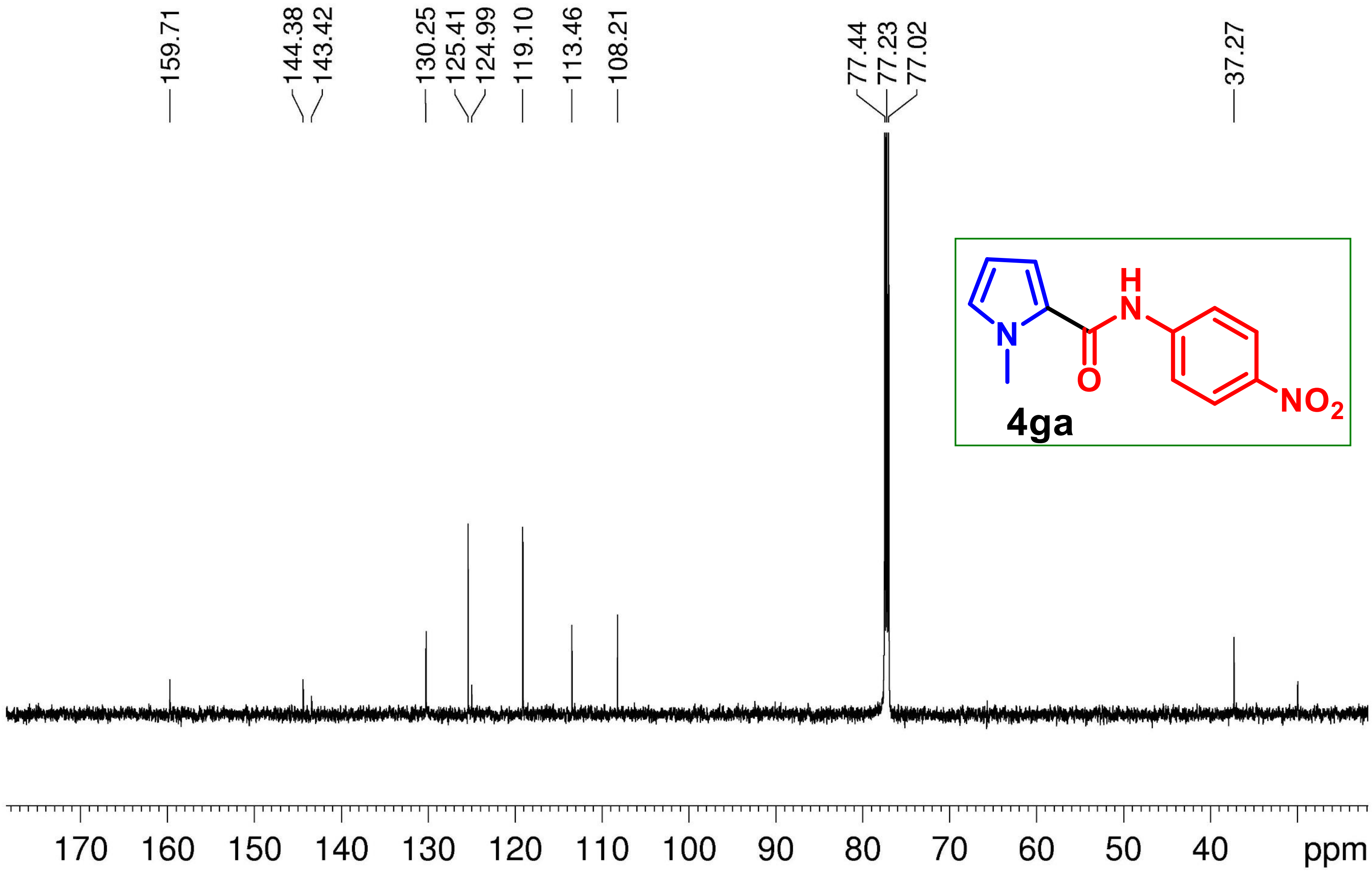

Figure S104. ^{13}C NMR spectrum (150 MHz, $CDCl_3$, 298 K) of the derivative **4ga**.

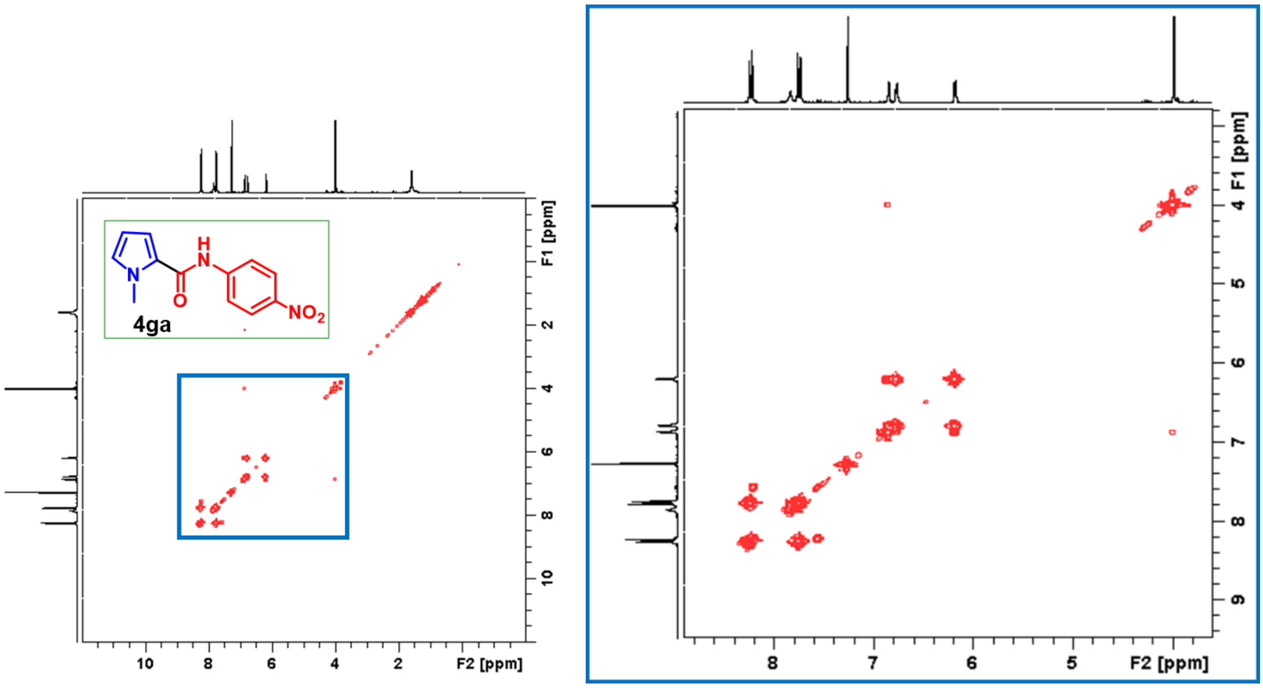

Figure S105. COSY NMR spectrum (600 MHz, $CDCl_3$, 298 K) of the derivative **4ga**, with expansion of significant portion of the spectrum in blue square.

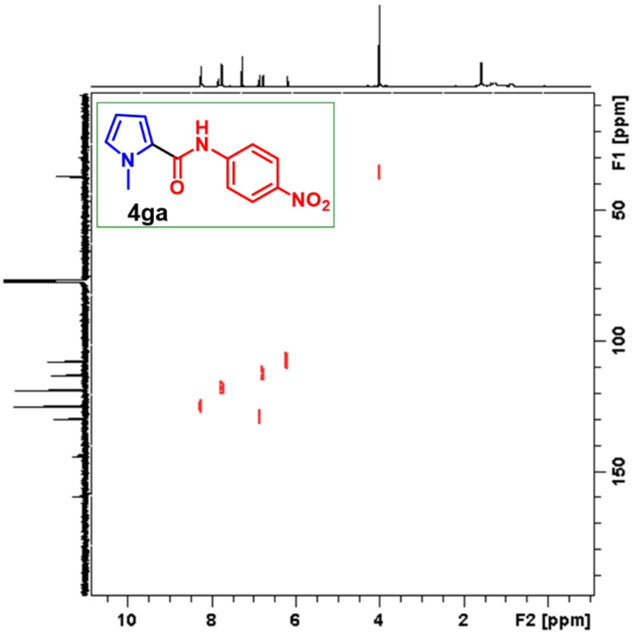

Figure S106. HSQC NMR spectrum (600 MHz, $CDCl_3$, 298 K) of the derivative **4ga**.

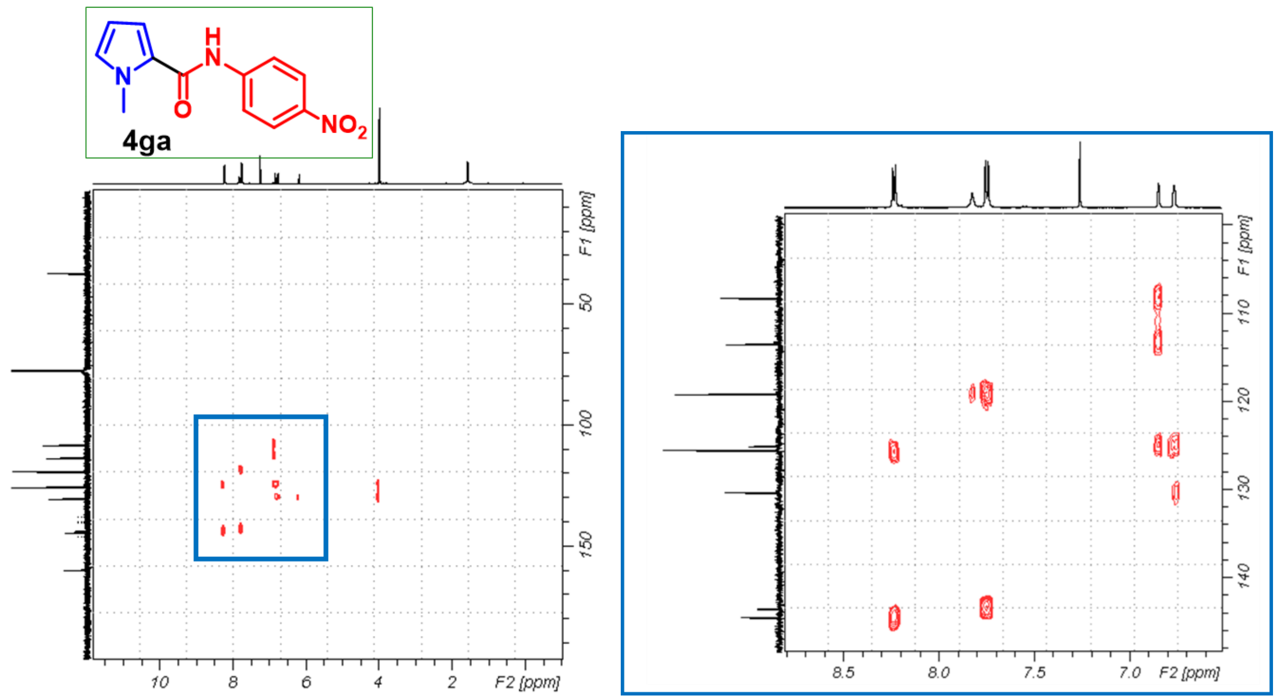

Figure S107. HMBC NMR spectrum (600 MHz, $CDCl_3$, 298 K) of the derivative **4ga**, with expansion of significant portion of the spectrum in blue square.

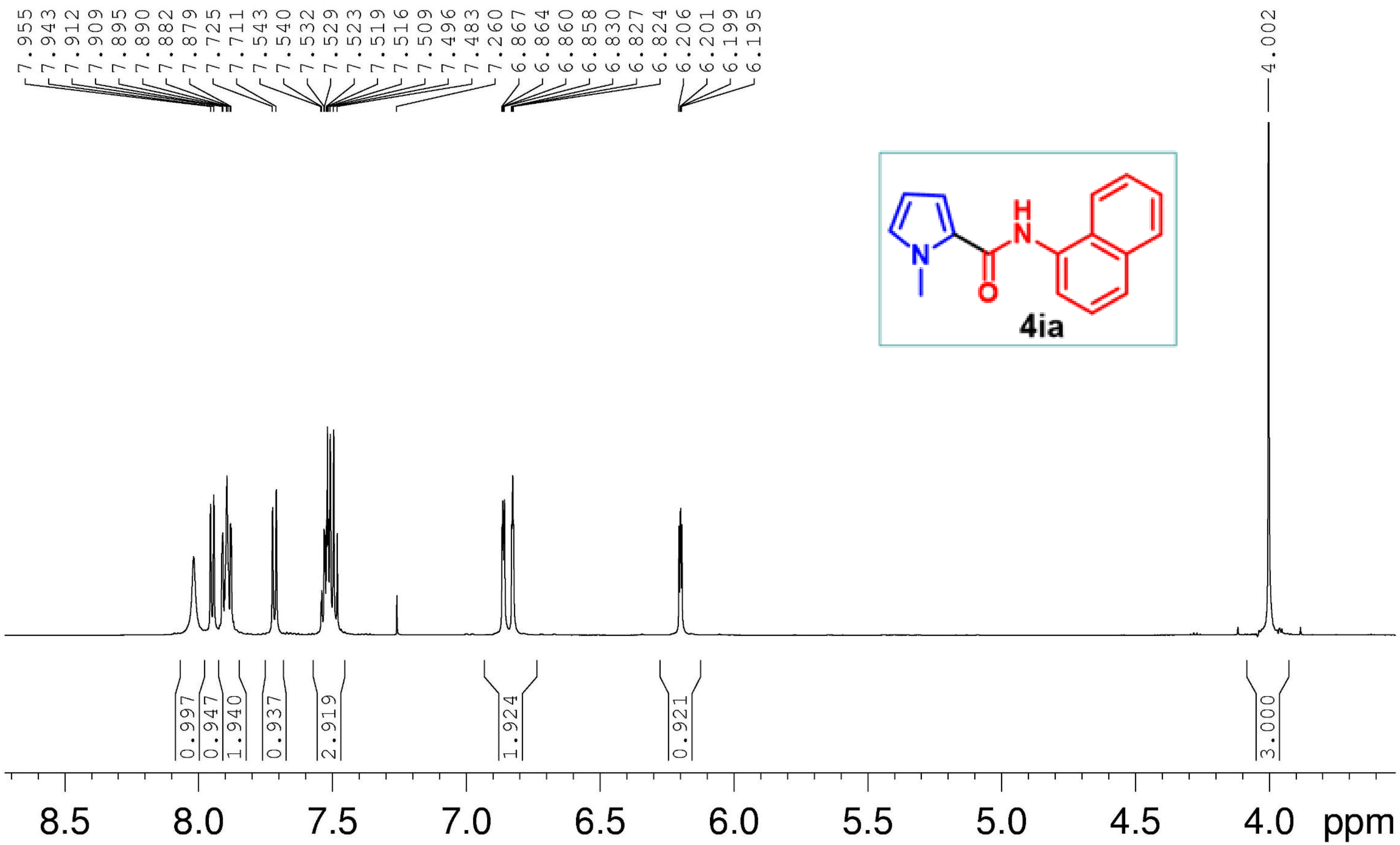

Figure S108. ^{1}H NMR spectrum (600 MHz, $CDCl_3$, 298 K) of the derivative **4ia**

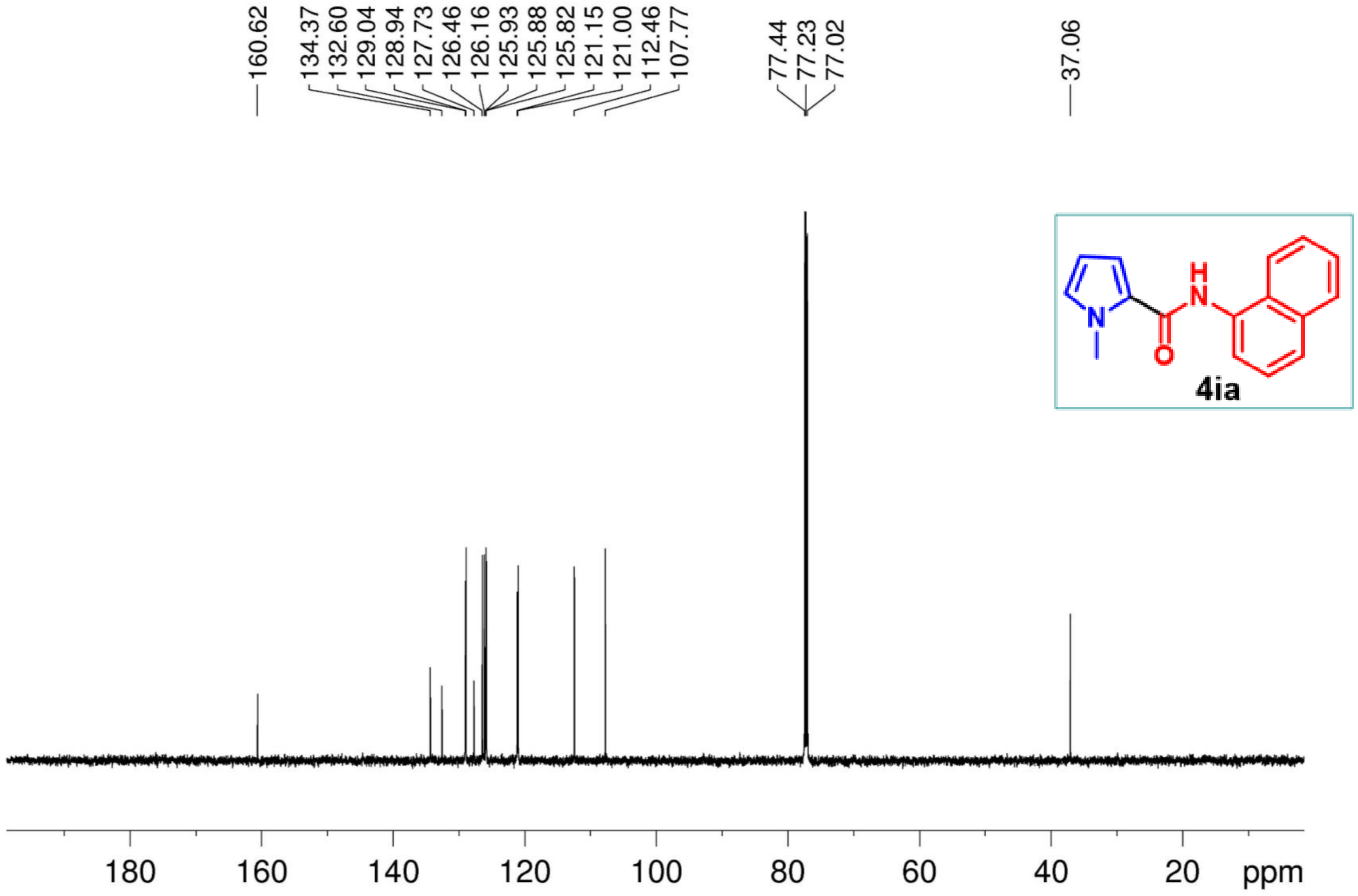

Figure S109. ^{13}C NMR spectrum (150 MHz, $CDCl_3$, 298 K) of the derivative **4ia**.

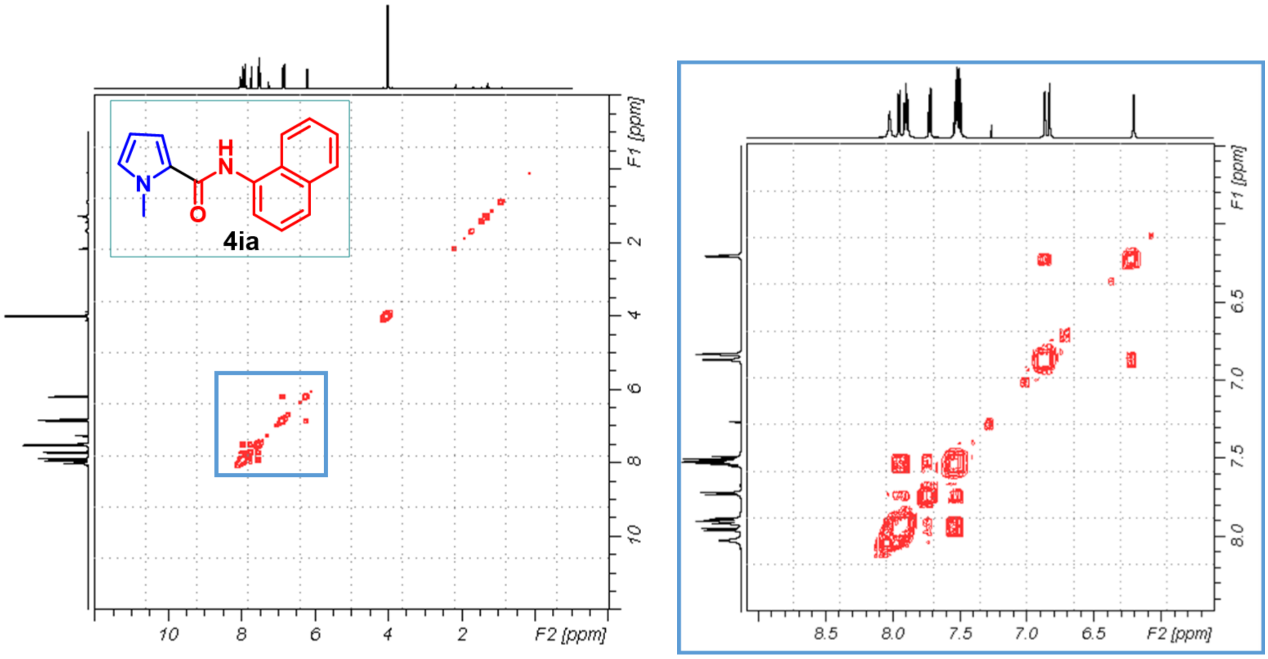

Figure S110. COSY NMR spectrum (600 MHz, $CDCl_3$, 298 K) of the derivative **4ia**, with expansion of significant portion of the spectrum in blue square.

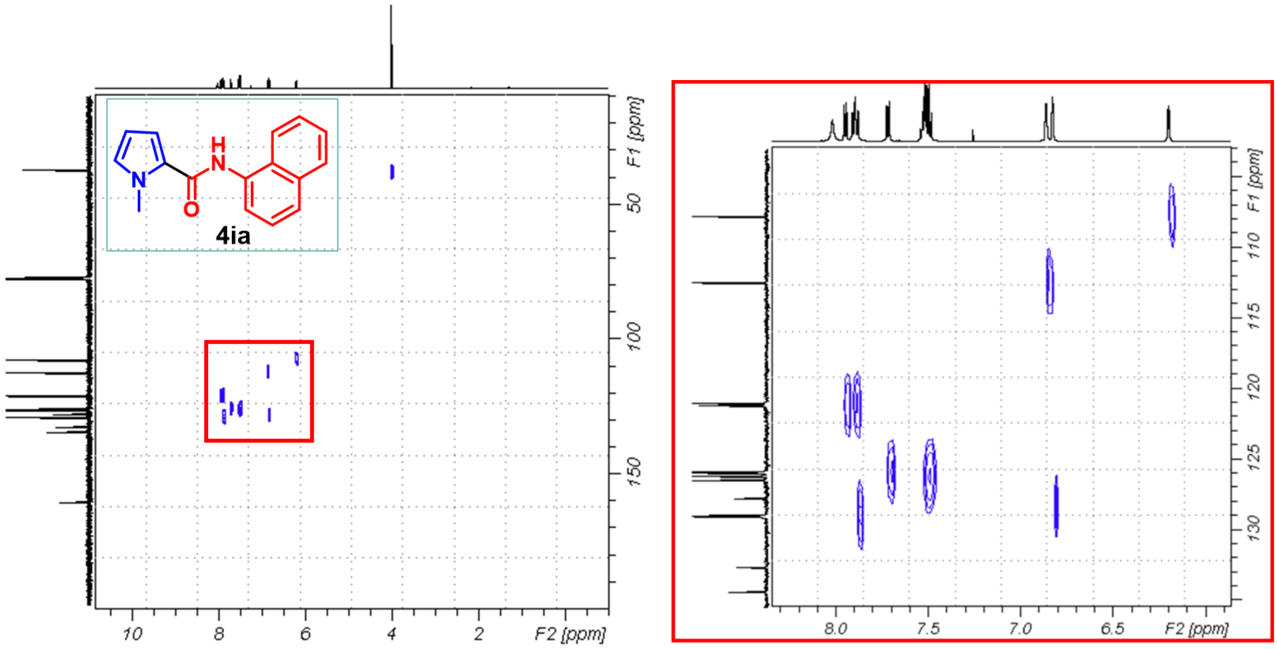

Figure S111. HSQC NMR spectrum (600 MHz, $CDCl_3$, 298 K) of the derivative **4ia**, with expansion of significant portion of the spectrum in red square.

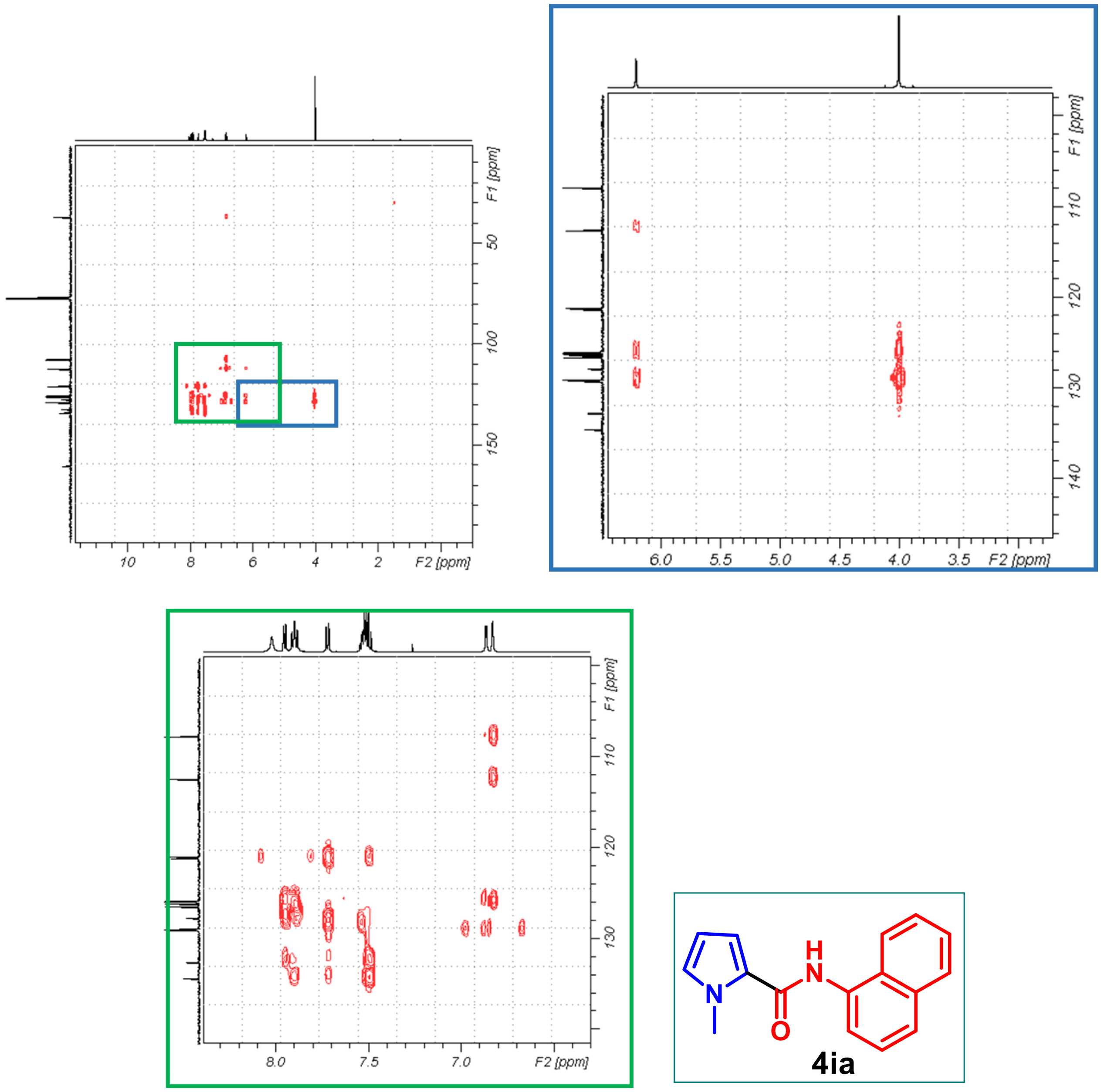

Figure S112. HMBC NMR spectrum (600 MHz, $CDCl_3$, 298 K) of the derivative **4ia**, with expansions of significant portions of the spectrum in blue and green squares.

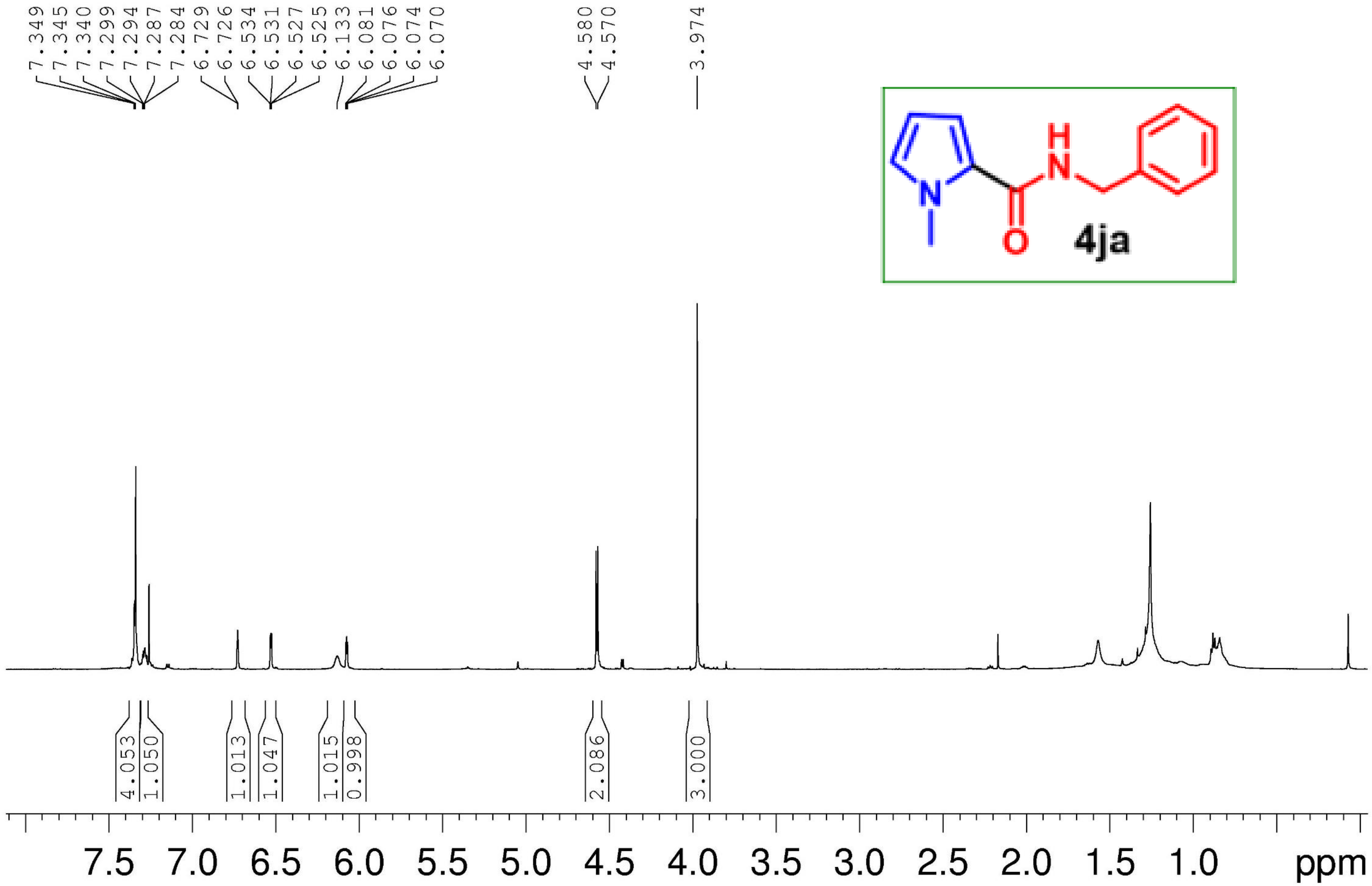

Figure S113. ^{1}H NMR spectrum (600 MHz, $CDCl_3$, 298 K) of the derivative **4ja**.

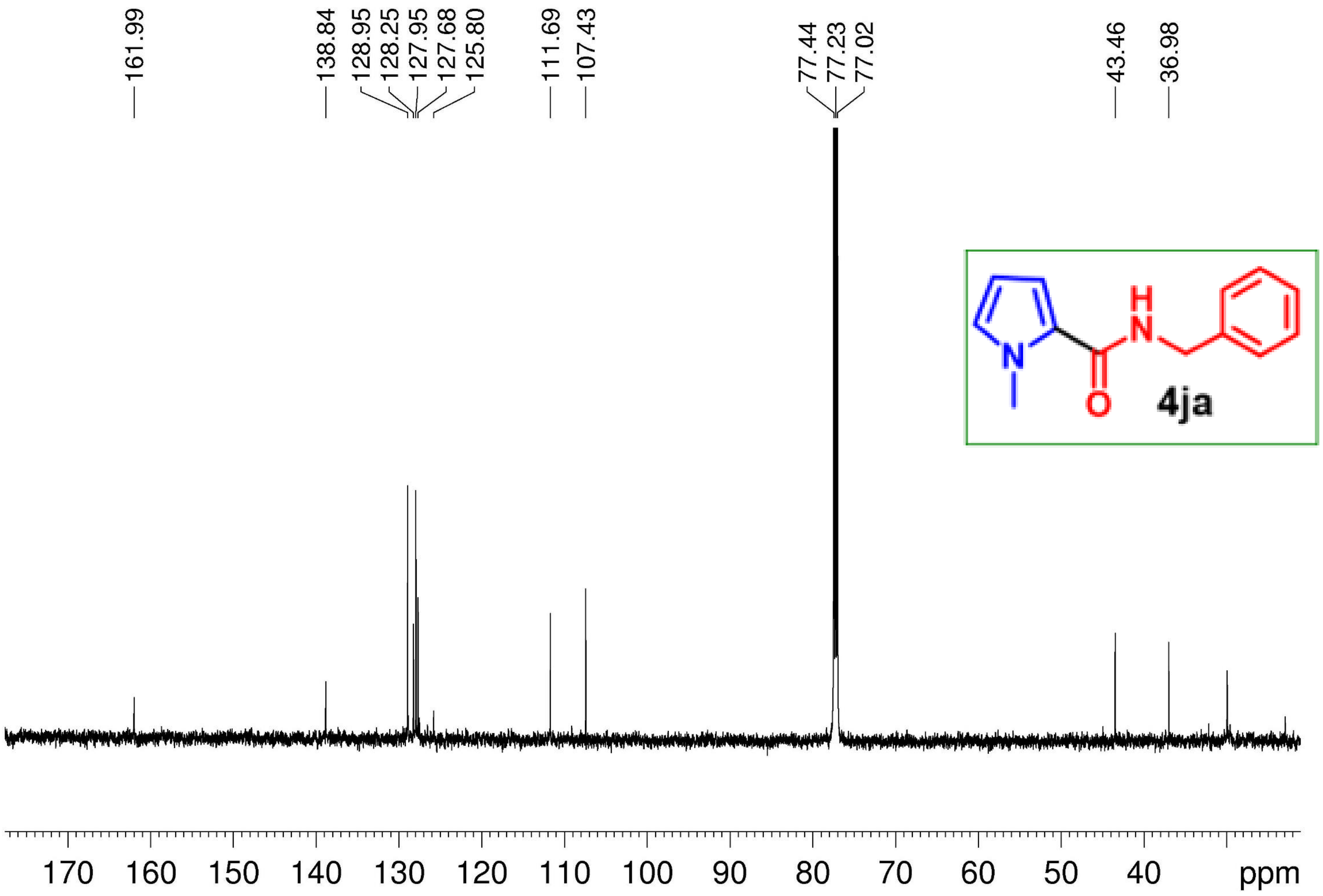

Figure S114. ^{13}C NMR spectrum (150 MHz, $CDCl_3$, 298 K) of the derivative **4ja**.

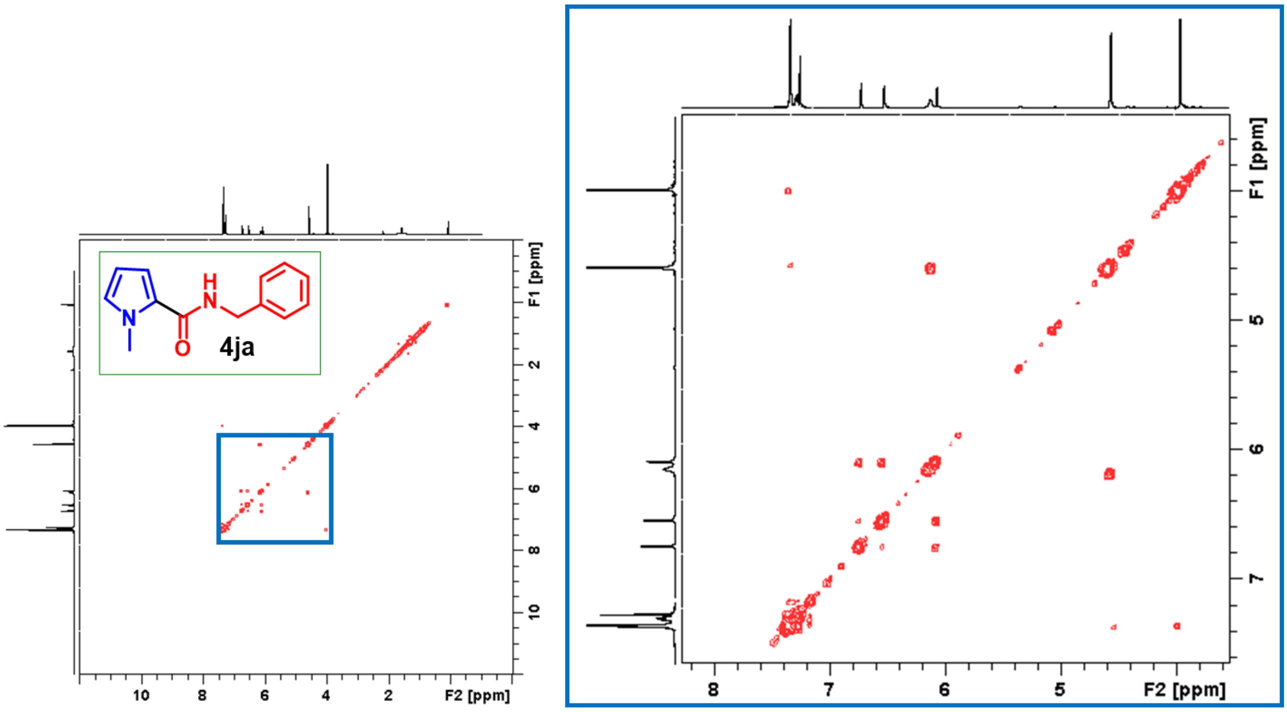

Figure S115. COSY NMR spectrum (600 MHz, $CDCl_3$, 298 K) of the derivative **4ja**, with expansion of significant portion of the spectrum in blue square.

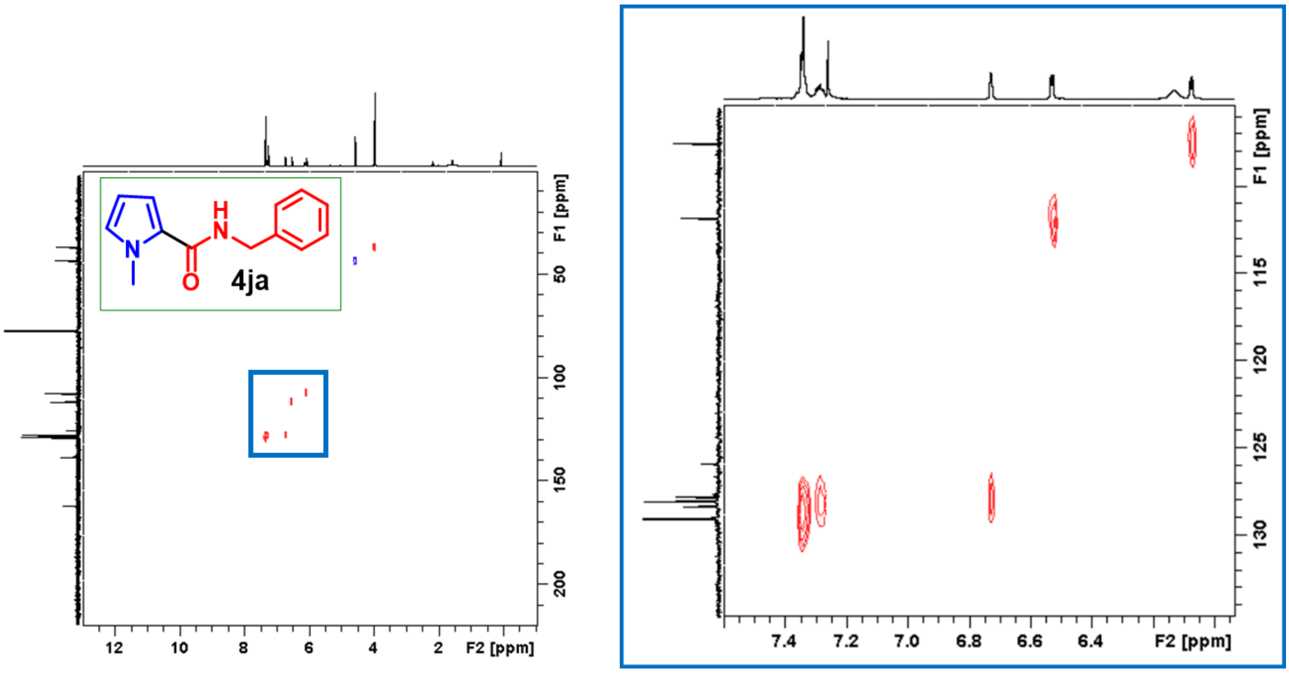

Figure S116. HSQC NMR spectrum (600 MHz, $CDCl_3$, 298 K) of the derivative **4ja**, with expansion of significant portion of the spectrum in blue square.

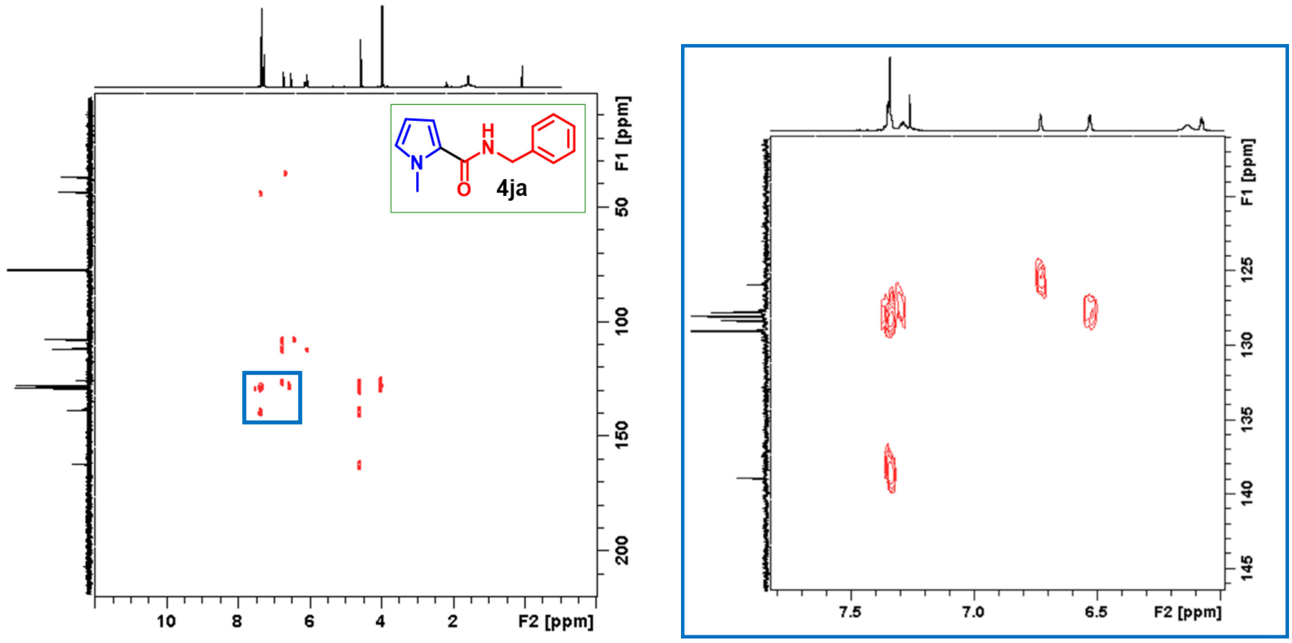

Figure S117. HMBC NMR spectrum (600 MHz, $CDCl_3$, 298 K) of the derivative **4ja**, with expansion of significant portion of the spectrum in blue square.

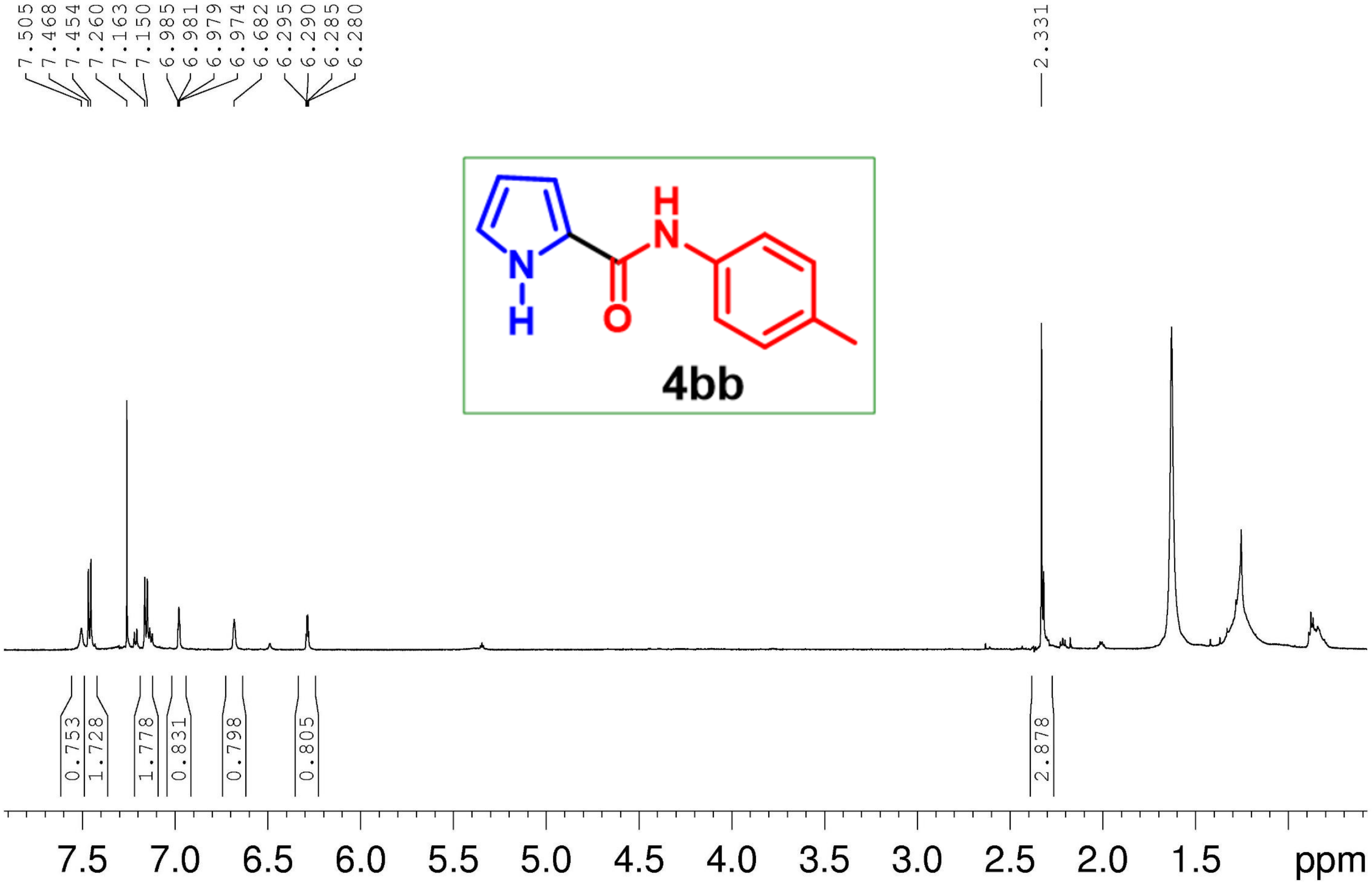

Figure S118. ^{1}H NMR spectrum (600 MHz, $CDCl_3$, 298 K) of the derivative **4bb**.

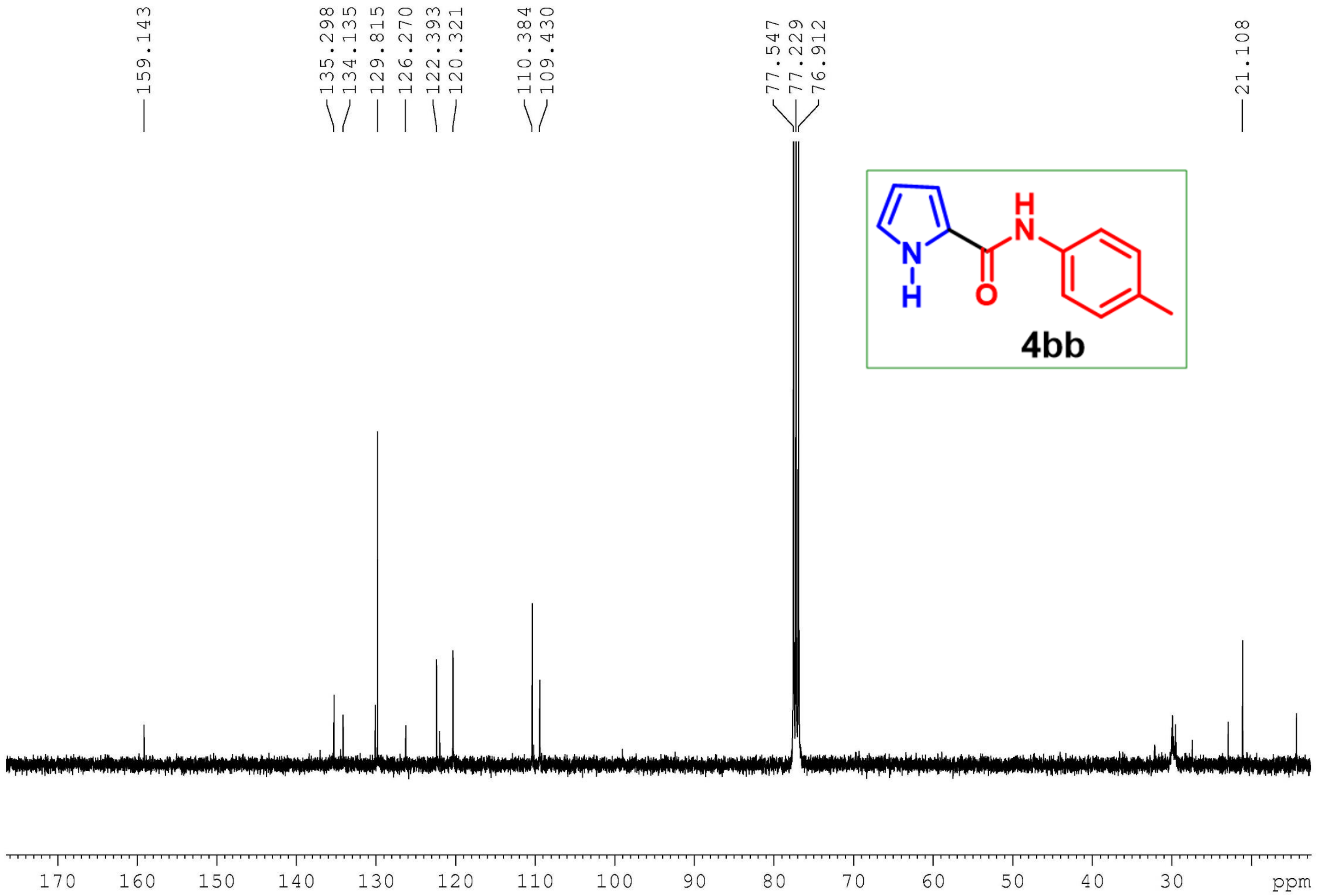

Figure S119. ^{13}C NMR spectrum (100 MHz, $CDCl_3$, 298 K) of the derivative **4bb**

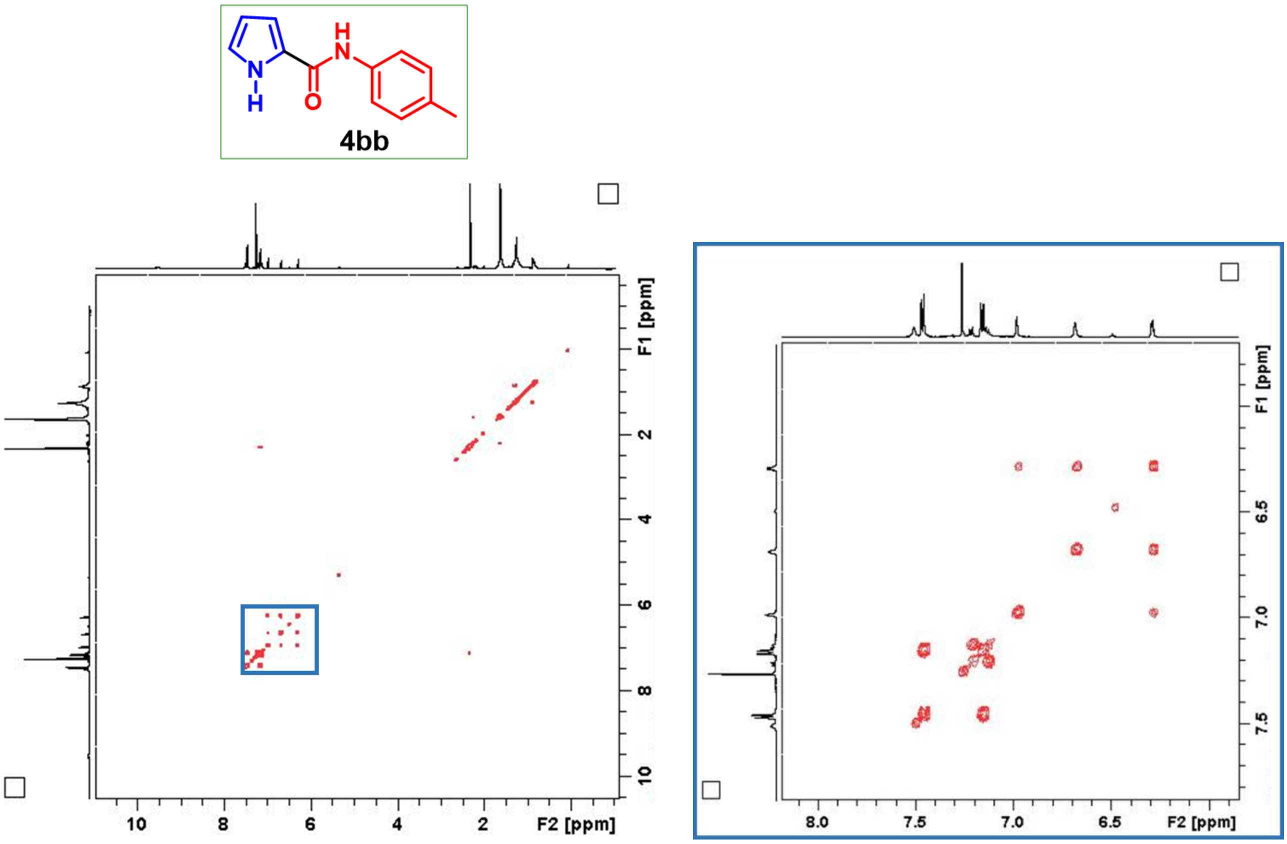

Figure S120. COSY NMR spectrum (600 MHz, $CDCl_3$, 298 K) of the derivative **4bb**, with expansion of significant portion of the spectrum in blue square.

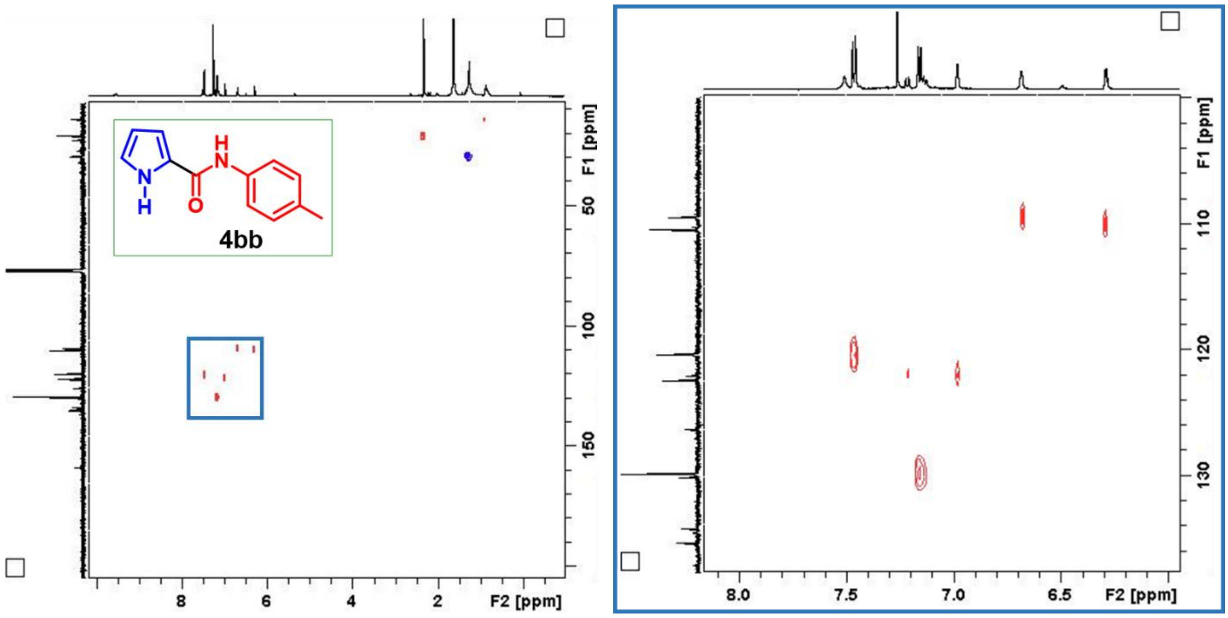

Figure S121. HSQC NMR spectrum (600 MHz, $CDCl_3$, 298 K) of the derivative **4bb**, with expansion of significant portion of the spectrum in blue square.

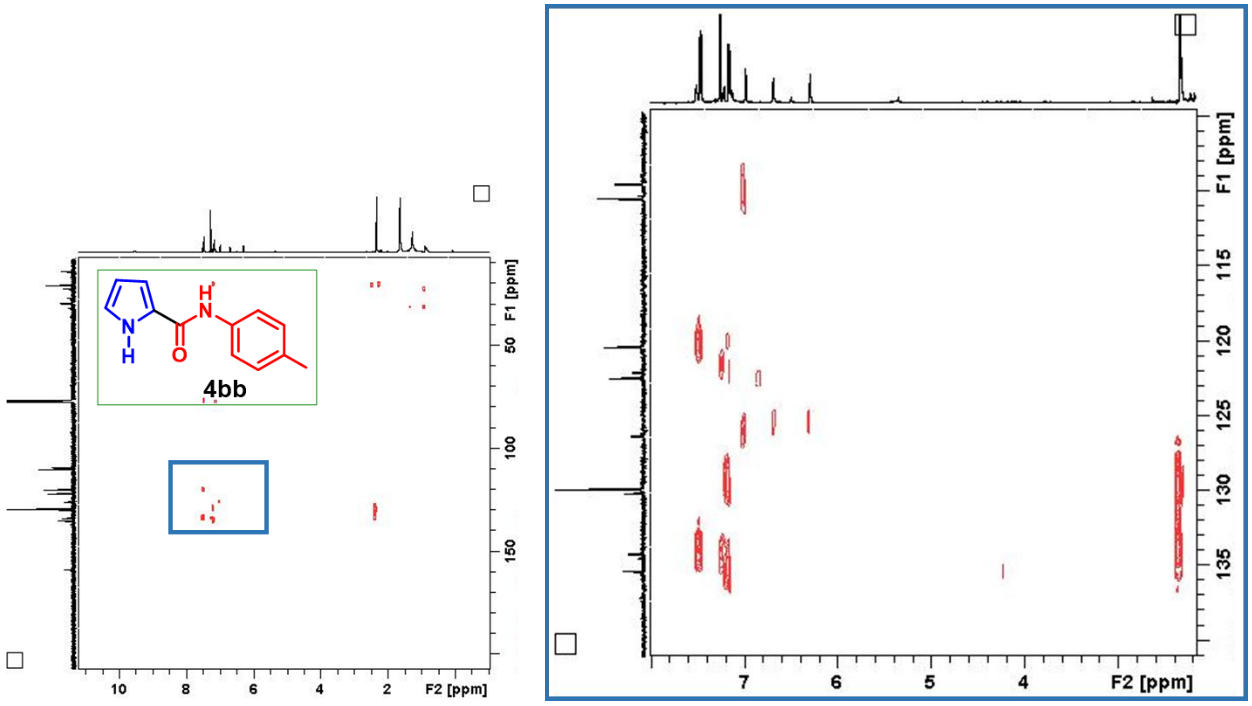

Figure S122. HMBC NMR spectrum (600 MHz, $CDCl_3$, 298 K) of the derivative **4bb**, with expansion of significant portion of the spectrum in blue square.

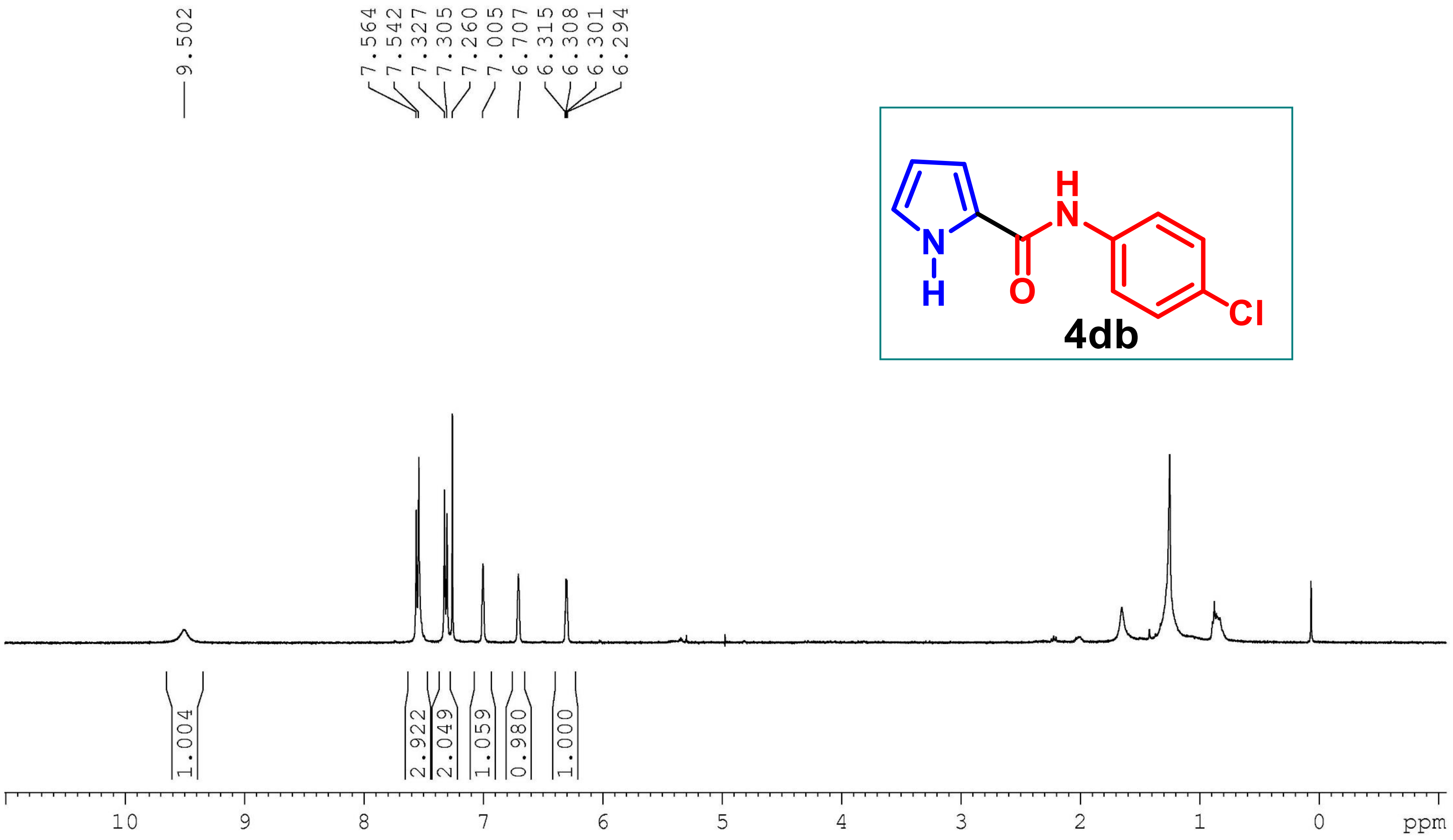

Figure S123. ^{1}H NMR spectrum (400 MHz, $CDCl_3$, 298 K) of the derivative **4db**.

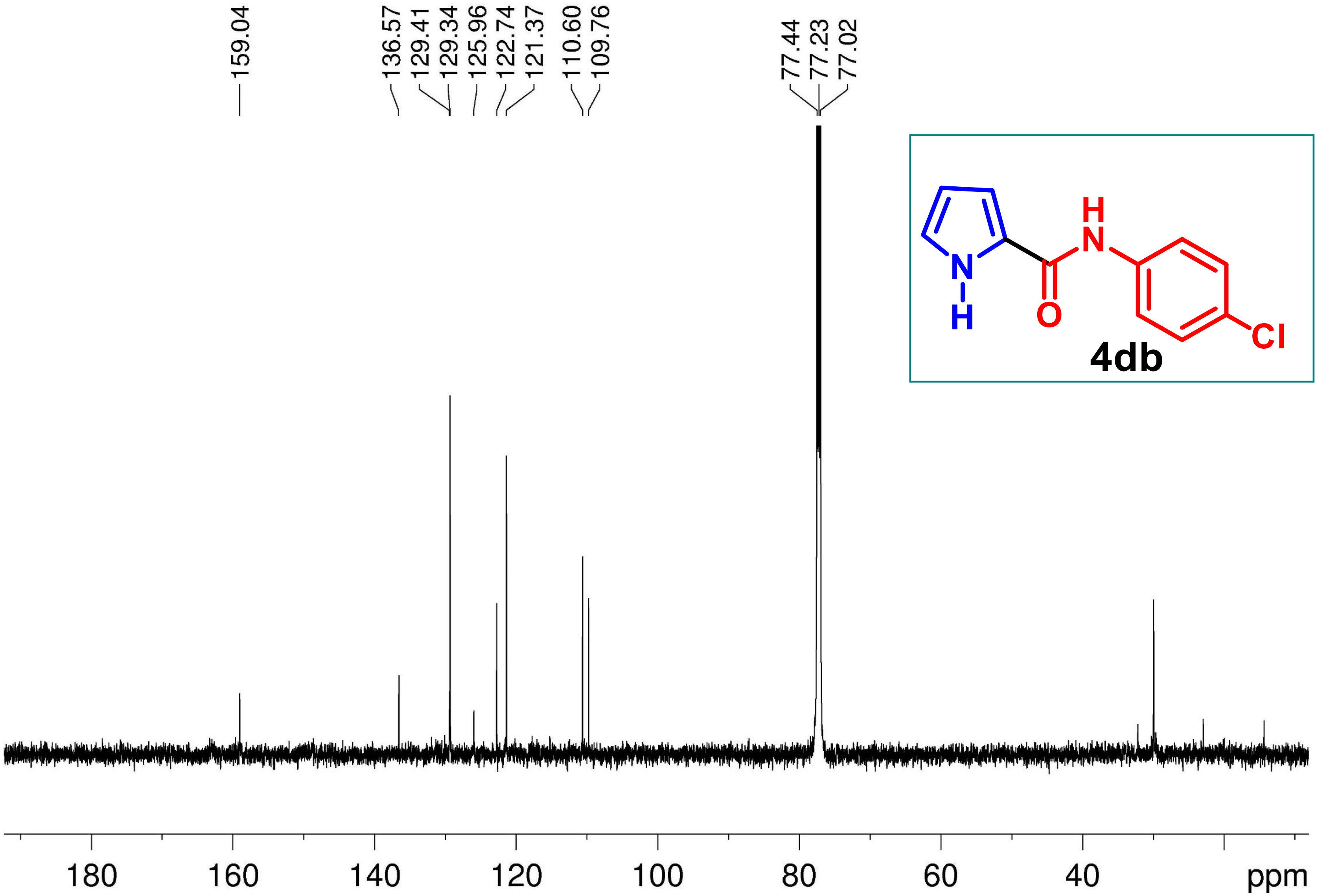

Figure S124. ^{13}C NMR spectrum (150 MHz, $CDCl_3$, 298 K) of the derivative **4db**.

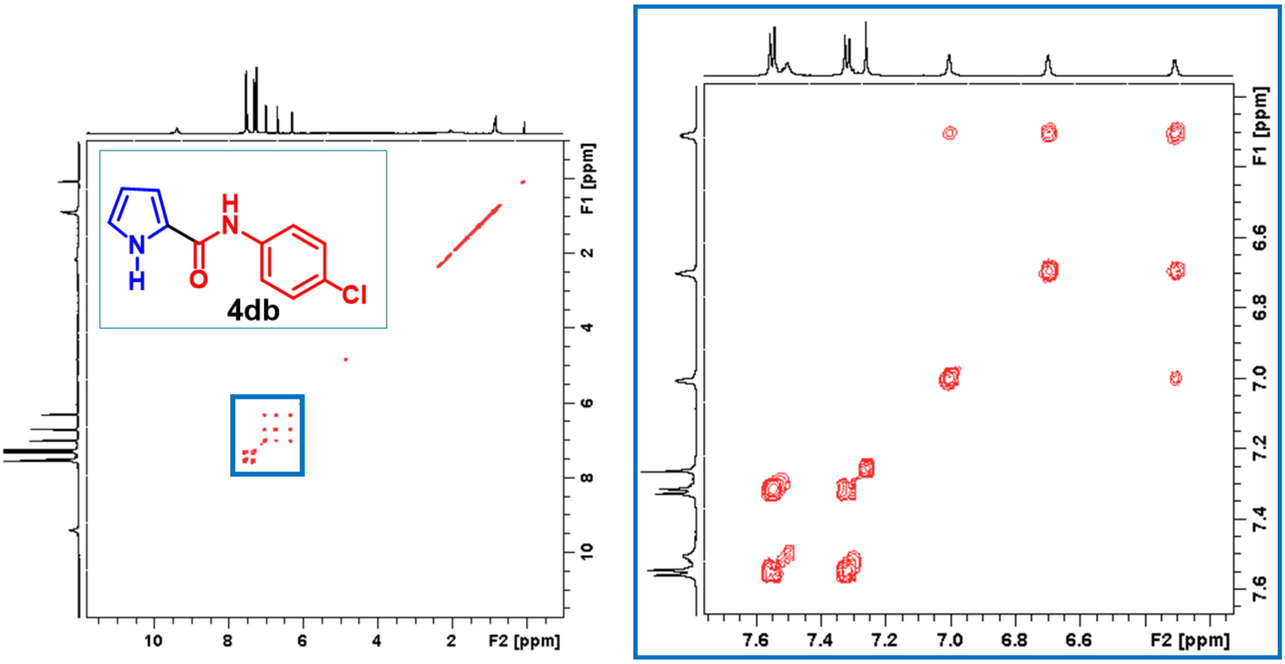

Figure S125. HMBC NMR spectrum (600 MHz, $CDCl_3$, 298 K) of the derivative **4db**, with expansion of significant portion of the spectrum in blue square.

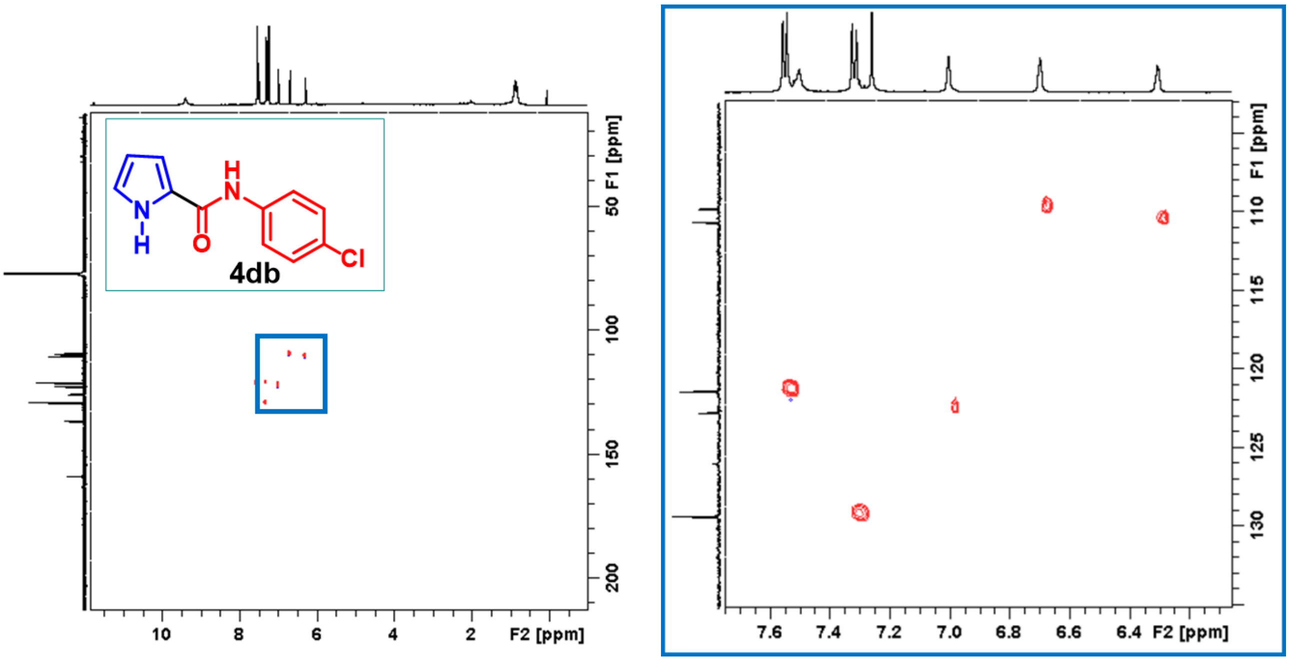

Figure S126. HSQC NMR spectrum (600 MHz, $CDCl_3$, 298 K) of the derivative **4db**, with expansion of significant portion of the spectrum in blue square.

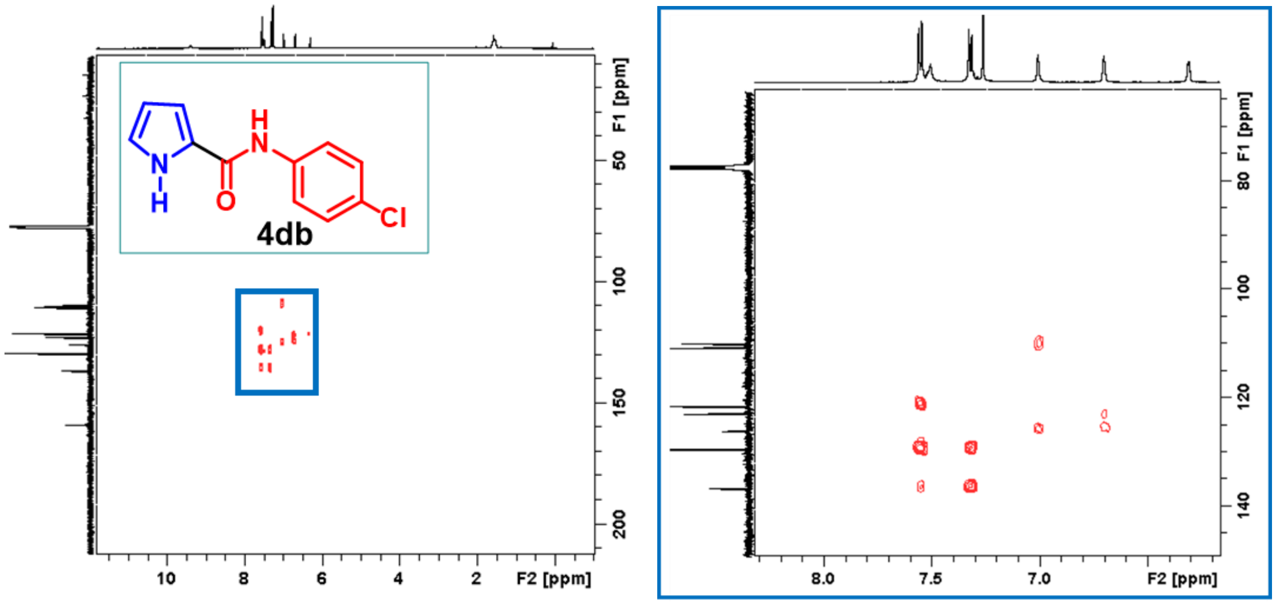

Figure S127. HMBC NMR spectrum (600 MHz, $CDCl_3$, 298 K) of the derivative **4db**, with expansion of significant portion of the spectrum in blue square.

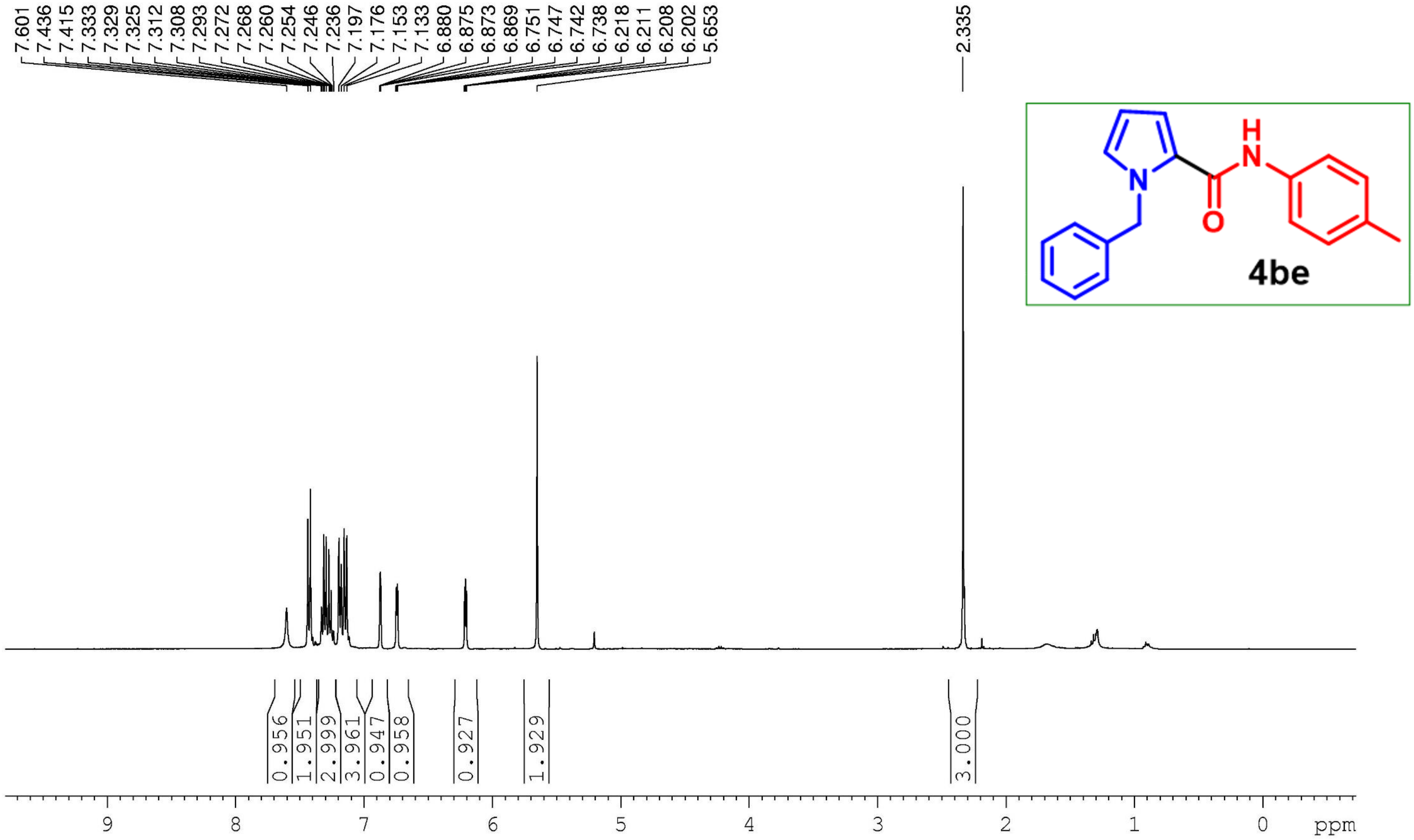

Figure S128. ^{1}H NMR spectrum (400 MHz, $CDCl_3$, 298 K) of the derivative **4be**.

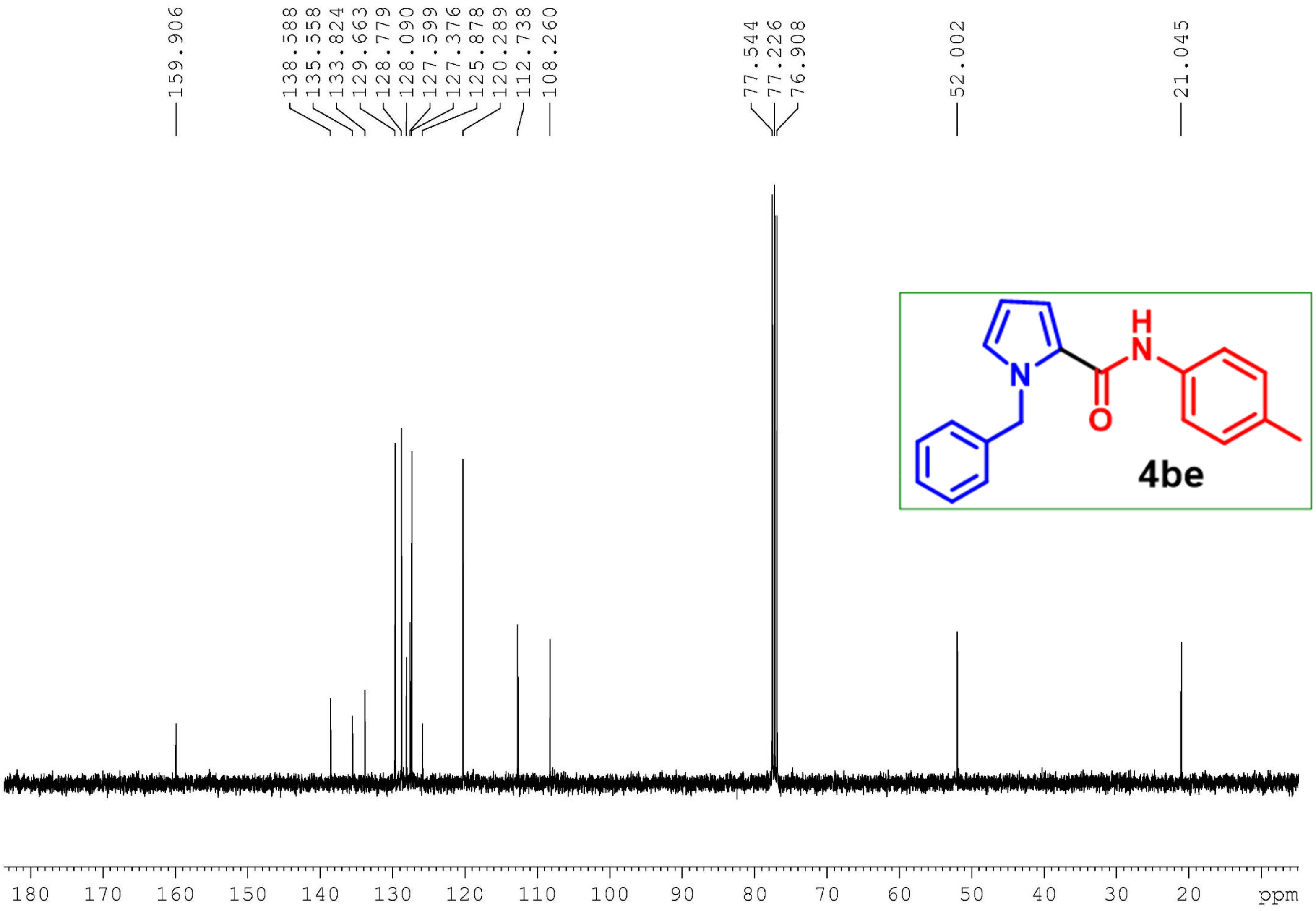

Figure S129. ^{13}C NMR spectrum (100 MHz, $CDCl_3$, 298 K) of the derivative **4be**.

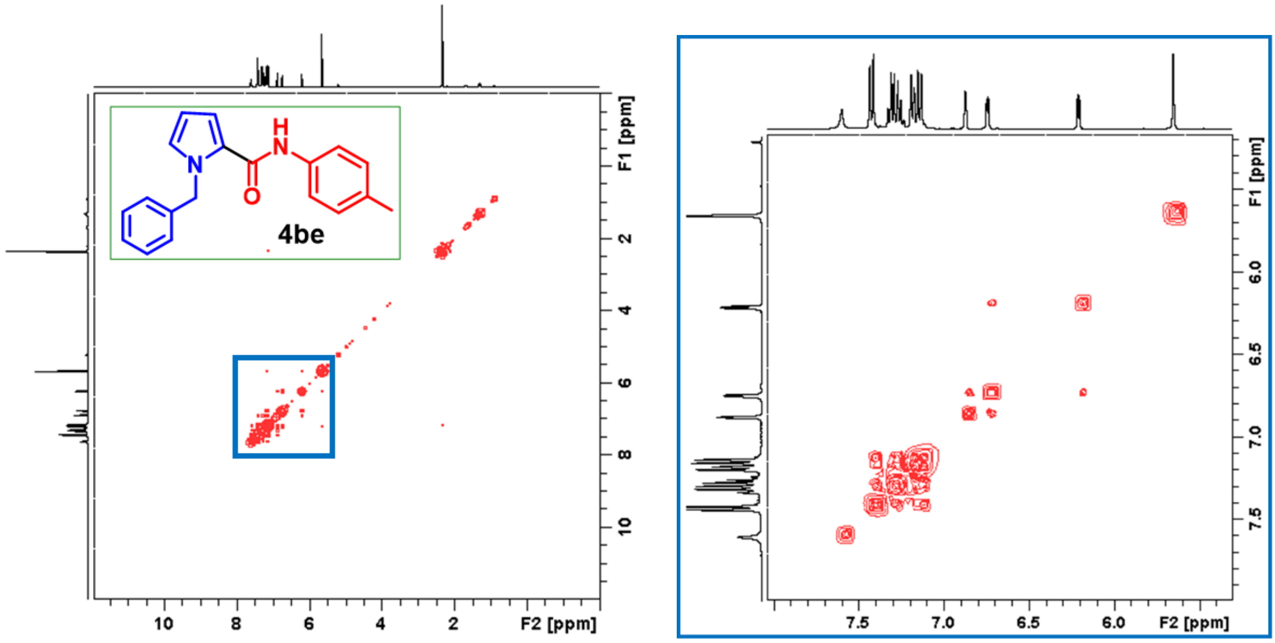

Figure S130. COSY NMR spectrum (400 MHz, $CDCl_3$, 298 K) of the derivative **4be**, with expansion of significant portion of the spectrum in blue square.

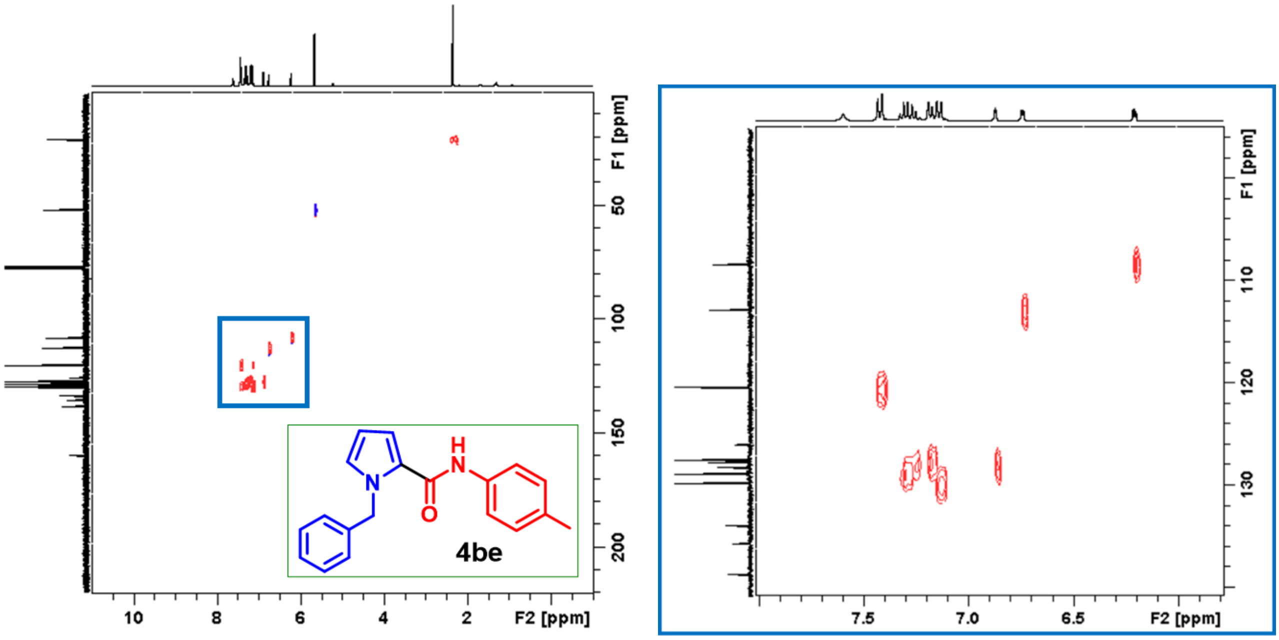

Figure S131. HSQC NMR spectrum (400 MHz, $CDCl_3$, 298 K) of the derivative **4be**, with expansion of significant portion of the spectrum in blue square.

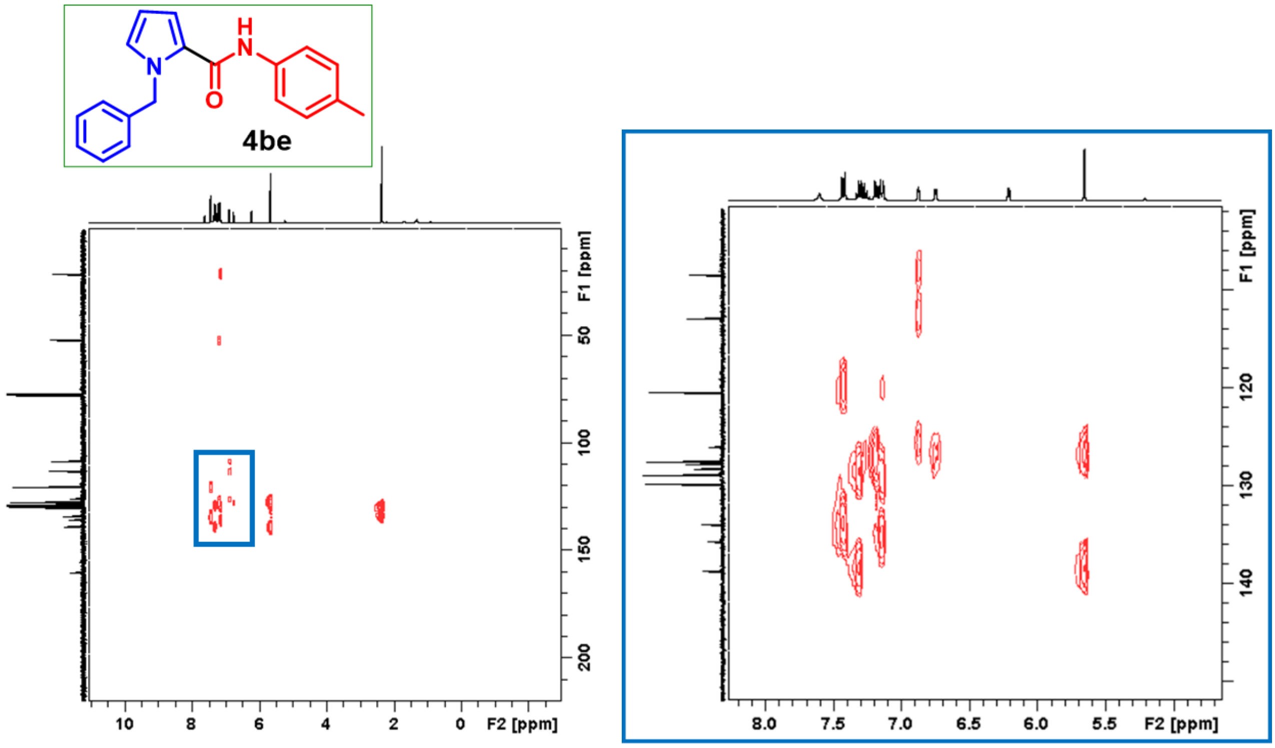

Figure S132. HMBC NMR spectrum (600 MHz, $CDCl_3$, 298 K) of the derivative **4be**, with expansion of significant portion of the spectrum in blue square.

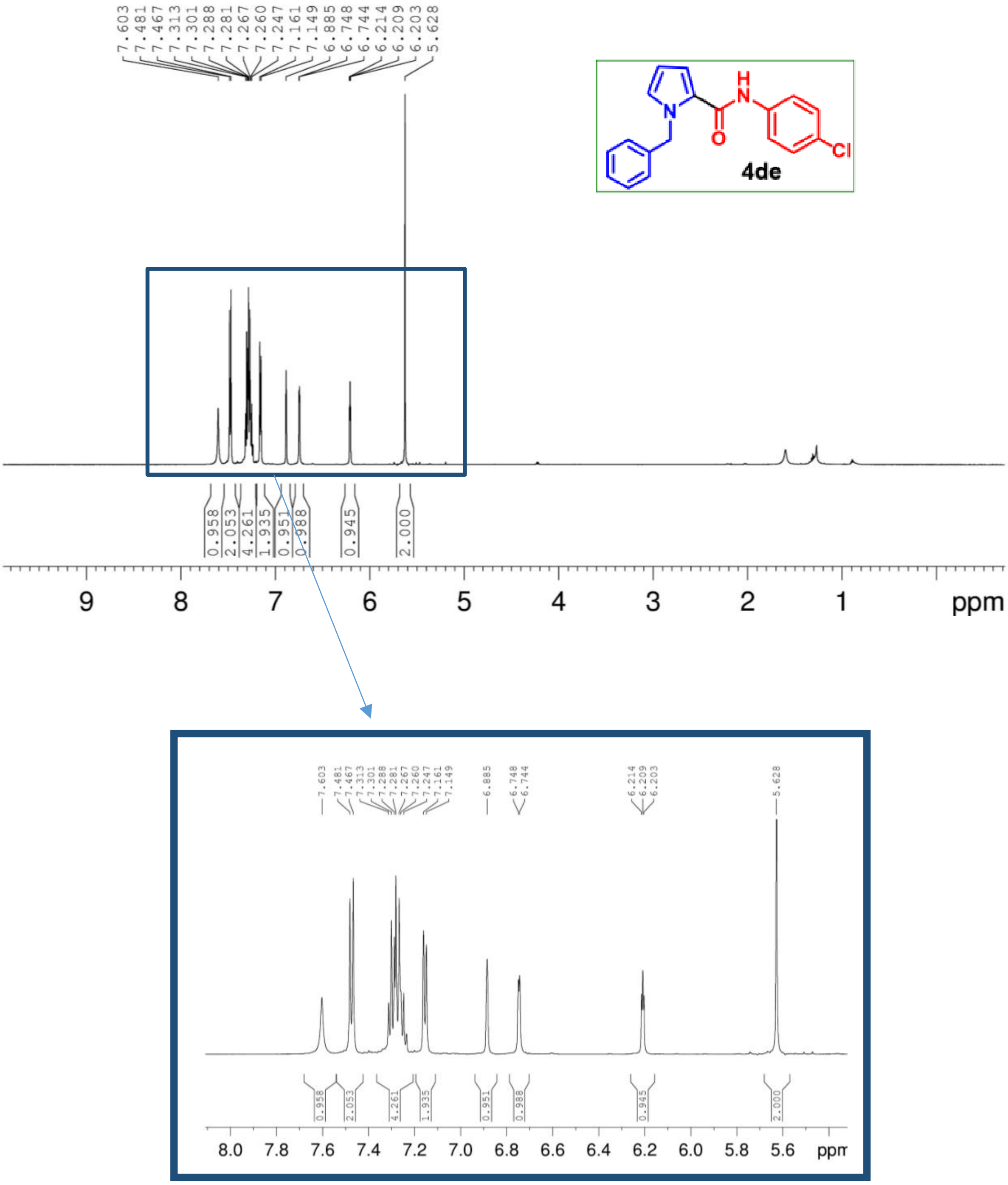

Figure S133. ^{1}H NMR spectrum (600 MHz, $CDCl_3$, 298 K) of the derivative **4de**, with expansion of significant portion of the spectrum in blue square.

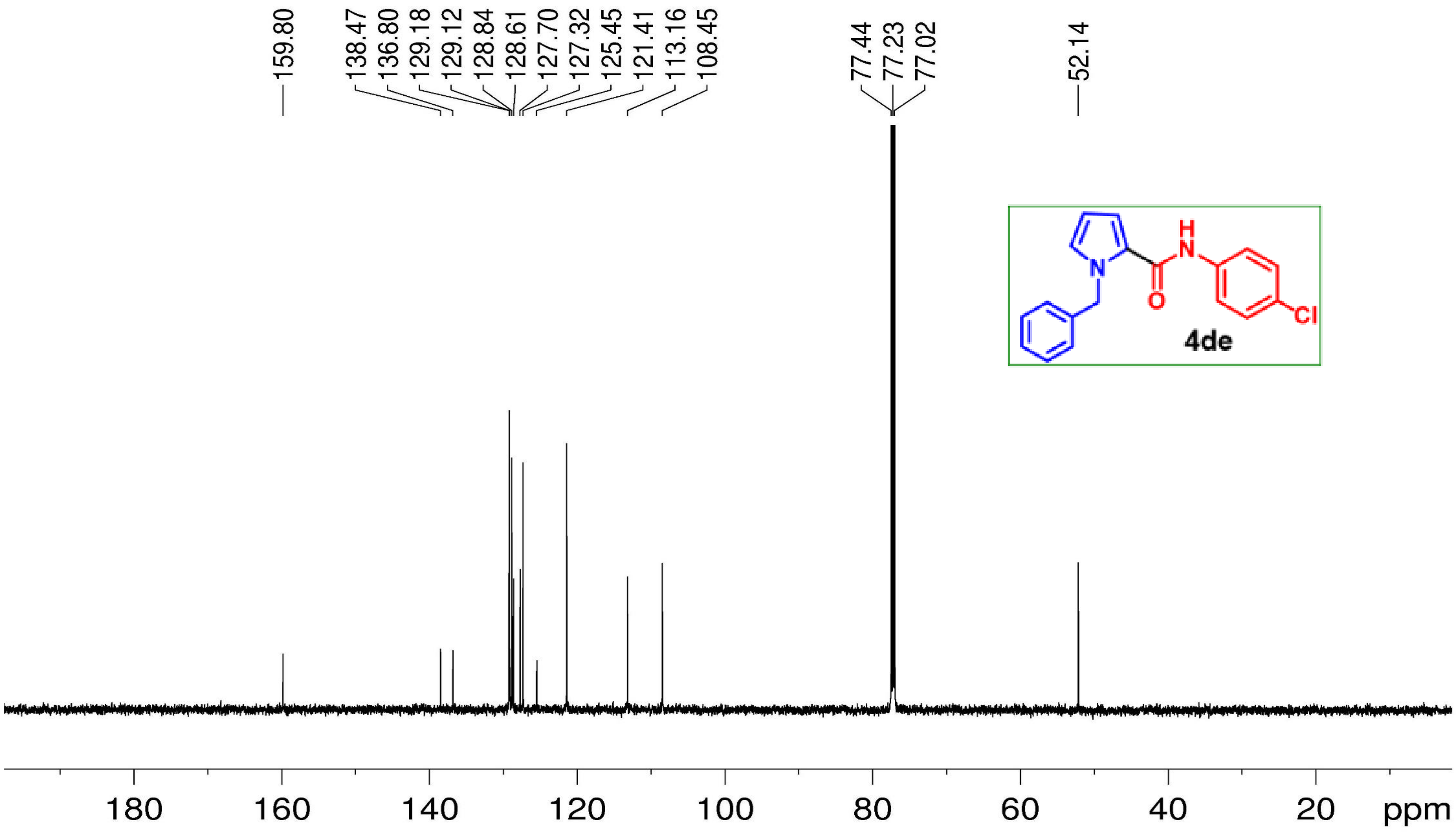

Figure S134. ^{13}C NMR spectrum (150 MHz, $CDCl_3$, 298 K) of the derivative **4de**.

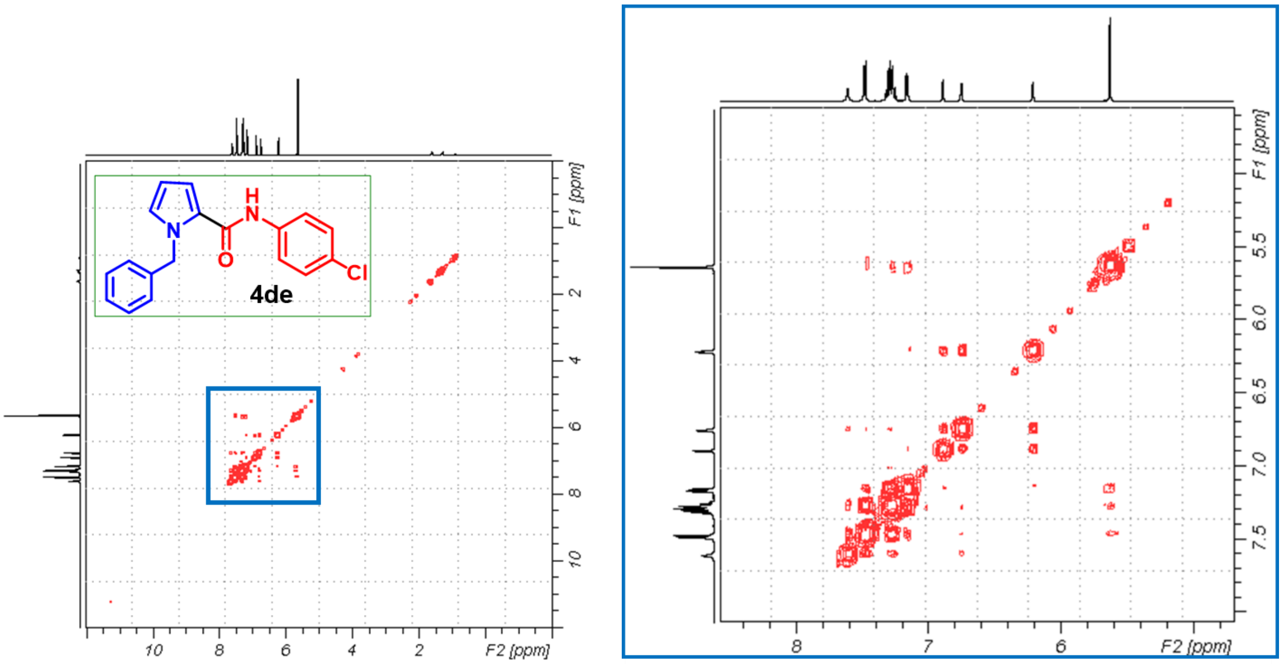

Figure S135. COSY NMR spectrum (600 MHz, $CDCl_3$, 298 K) of the derivative **4de**, with expansion of significant portion of the spectrum in blue square.

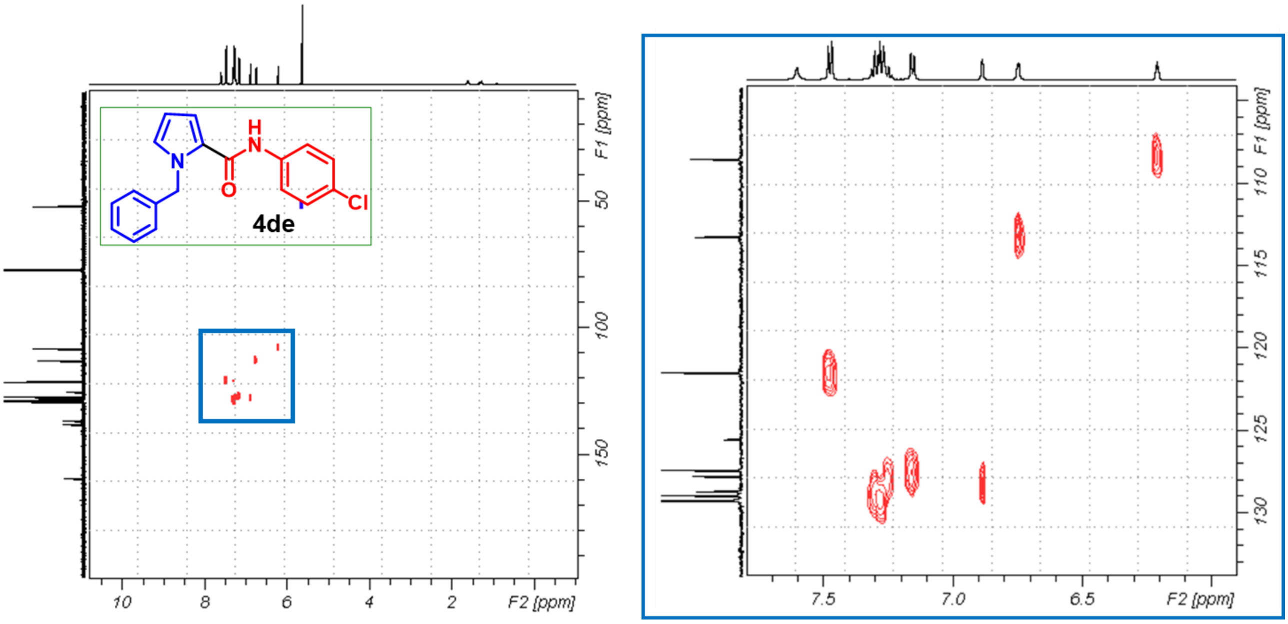

Figure S136. HSQC NMR spectrum (600 MHz, $CDCl_3$, 298 K) of the derivative **4de**, with expansion of significant portion of the spectrum in blue square.

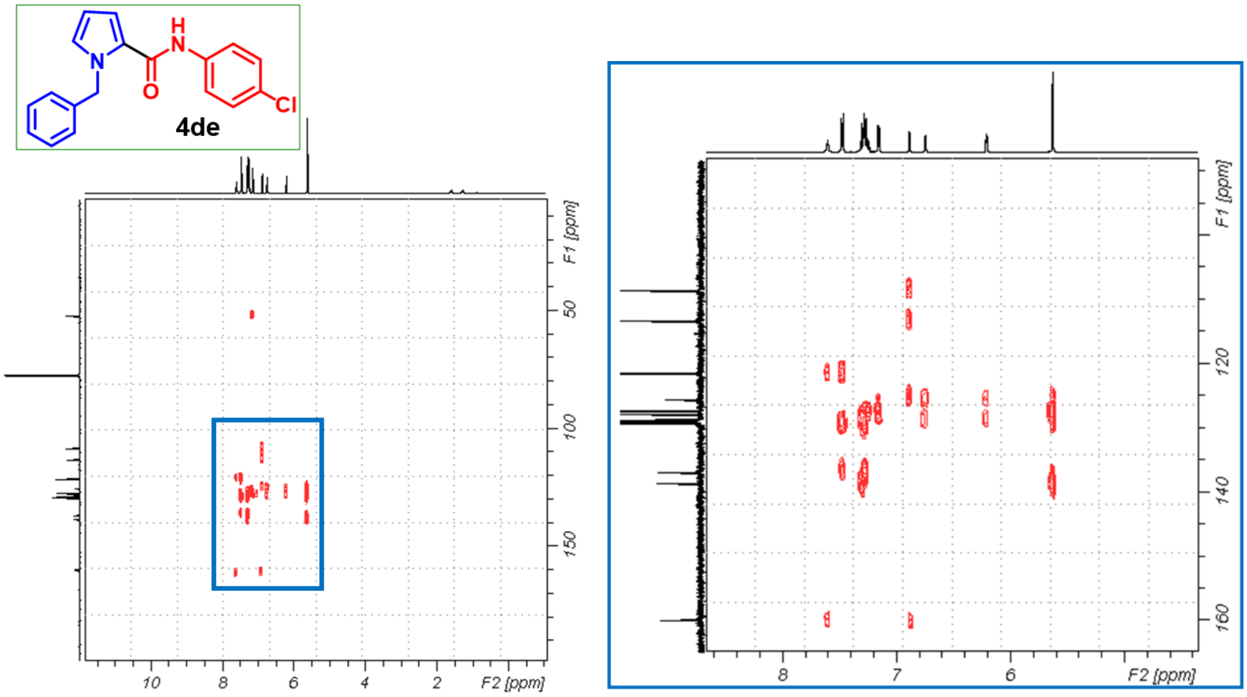

Figure S137. HMBC NMR spectrum (600 MHz, $CDCl_3$, 298 K) of the derivative **4de**, with expansion of significant portion of the spectrum in blue square.

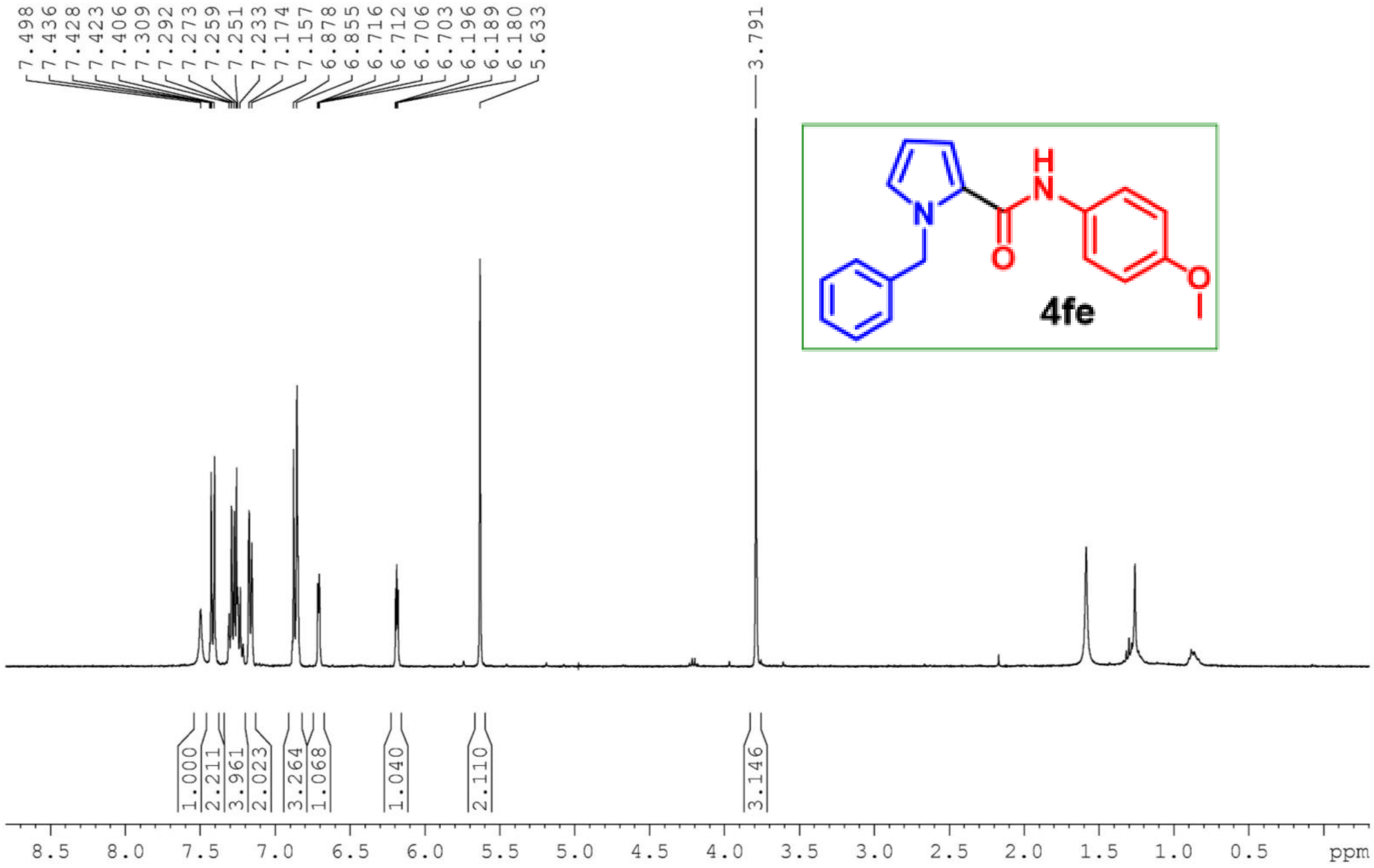

Figure S138. ^{1}H NMR spectrum (400 MHz, $CDCl_3$, 298 K) of the derivative **4fe**.

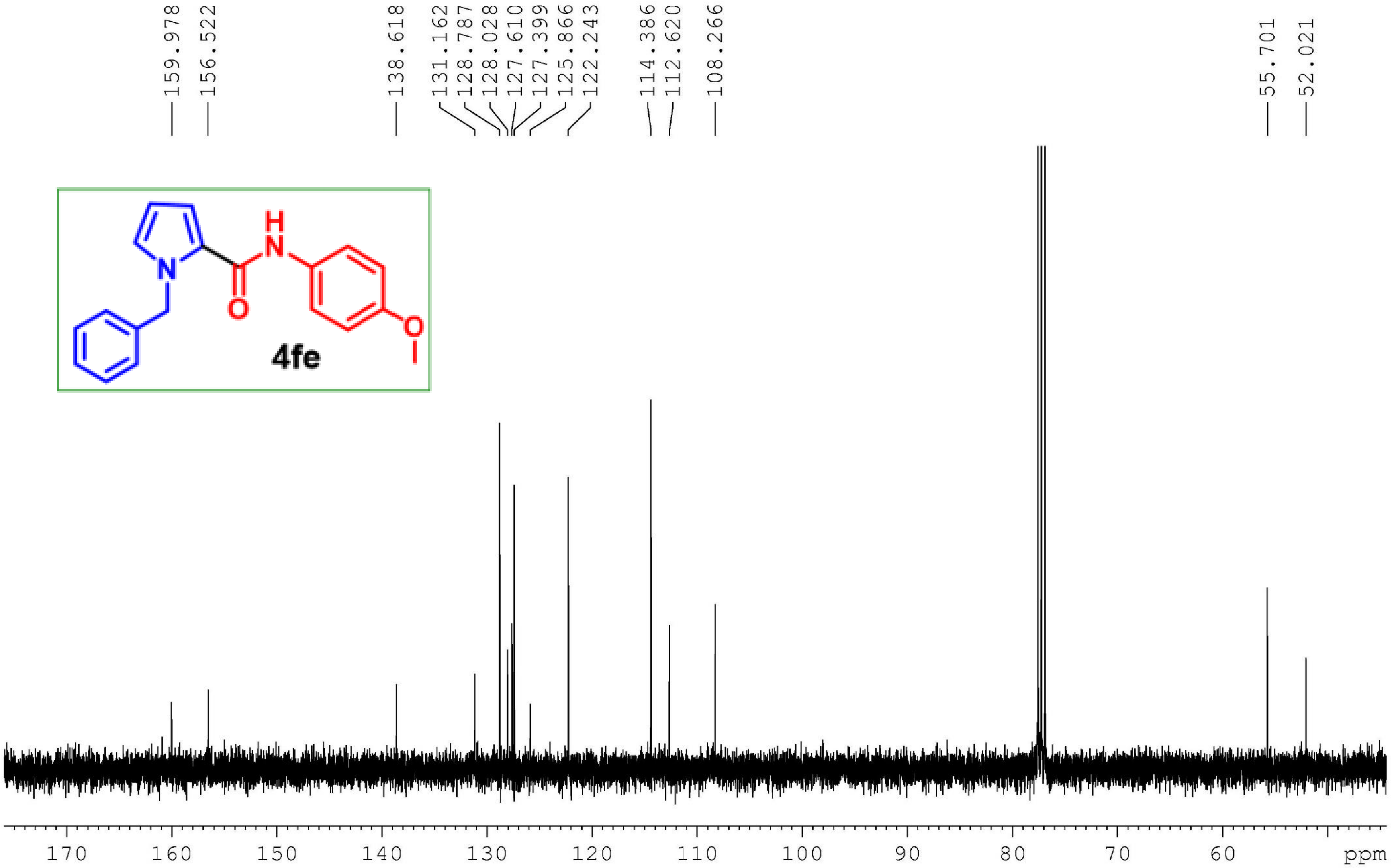

Figure S139. ^{13}C NMR spectrum (100 MHz, $CDCl_3$, 298 K) of the derivative **4fe**.

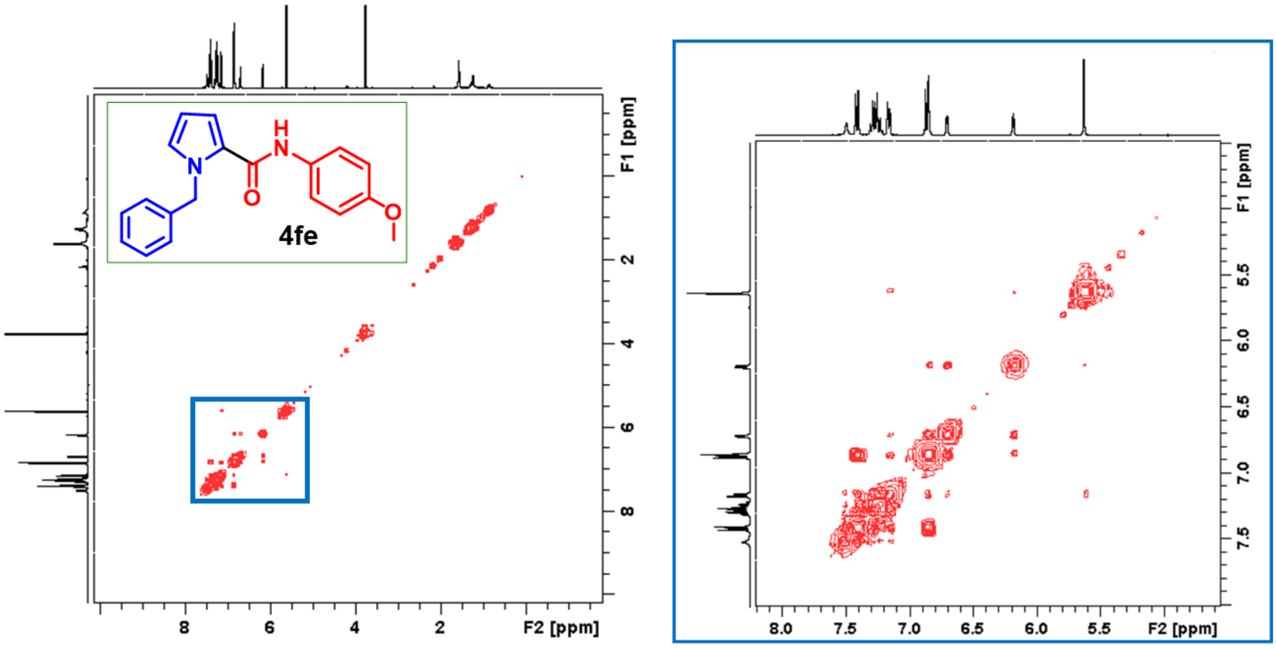

Figure S140. COSY NMR spectrum (400 MHz, $CDCl_3$, 298 K) of the derivative **4fe**, with expansion of significant portion of the spectrum in blue square.

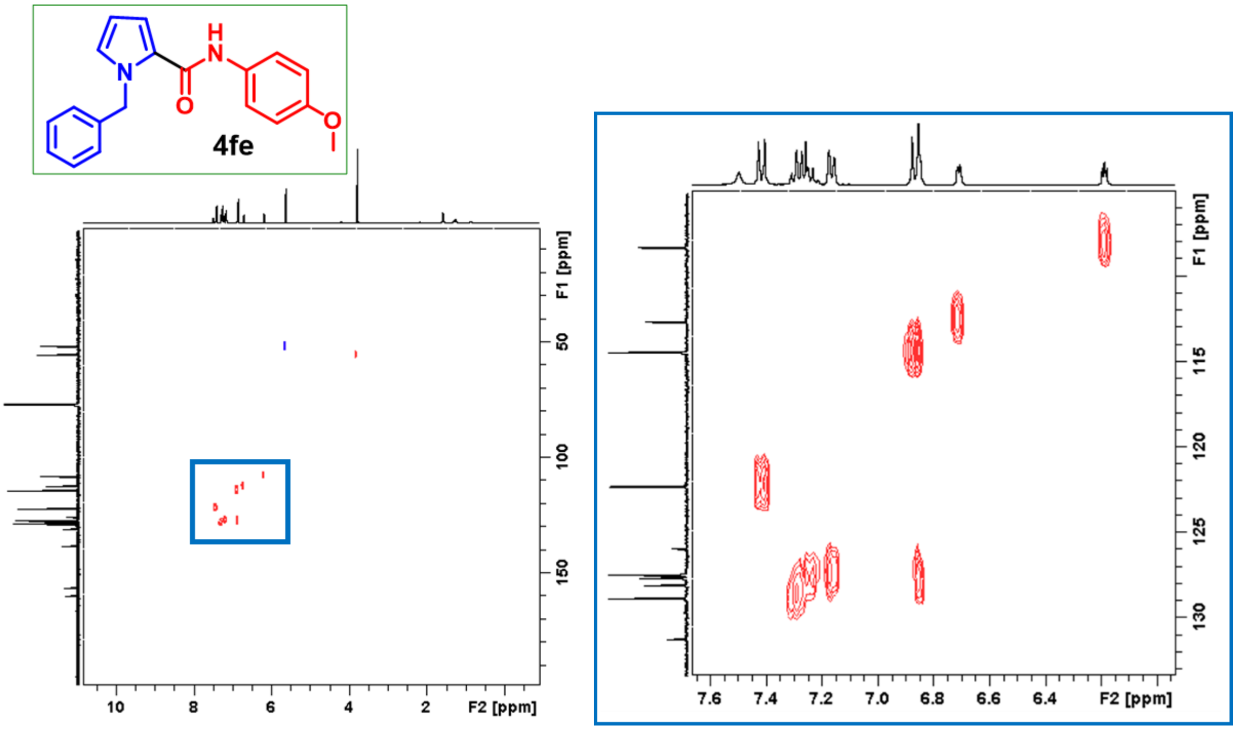

Figure S141. HSQC NMR spectrum (400 MHz, $CDCl_3$, 298 K) of the derivative **4fe**, with expansion of significant portion of the spectrum in blue square.

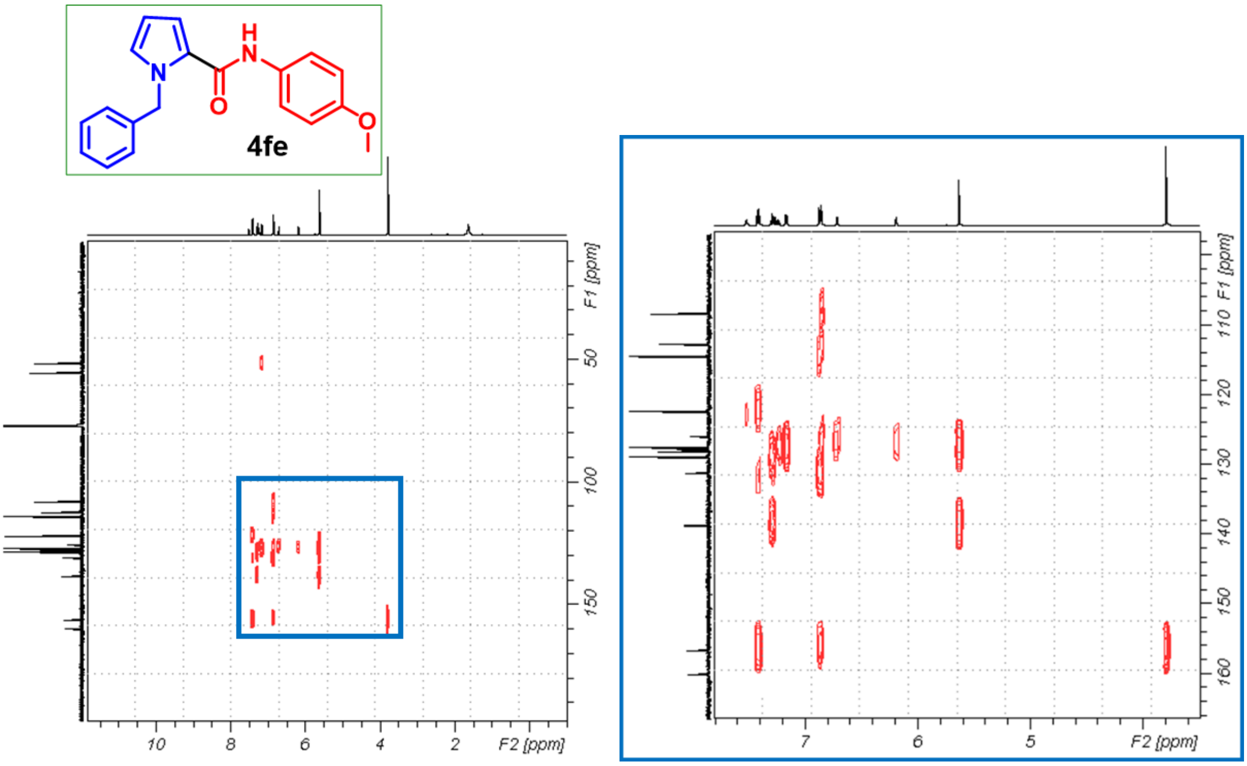

Figure S142. HMBC NMR spectrum (600 MHz, $CDCl_3$, 298 K) of the derivative **4fe**, with expansion of significant portion of the spectrum in blue square.

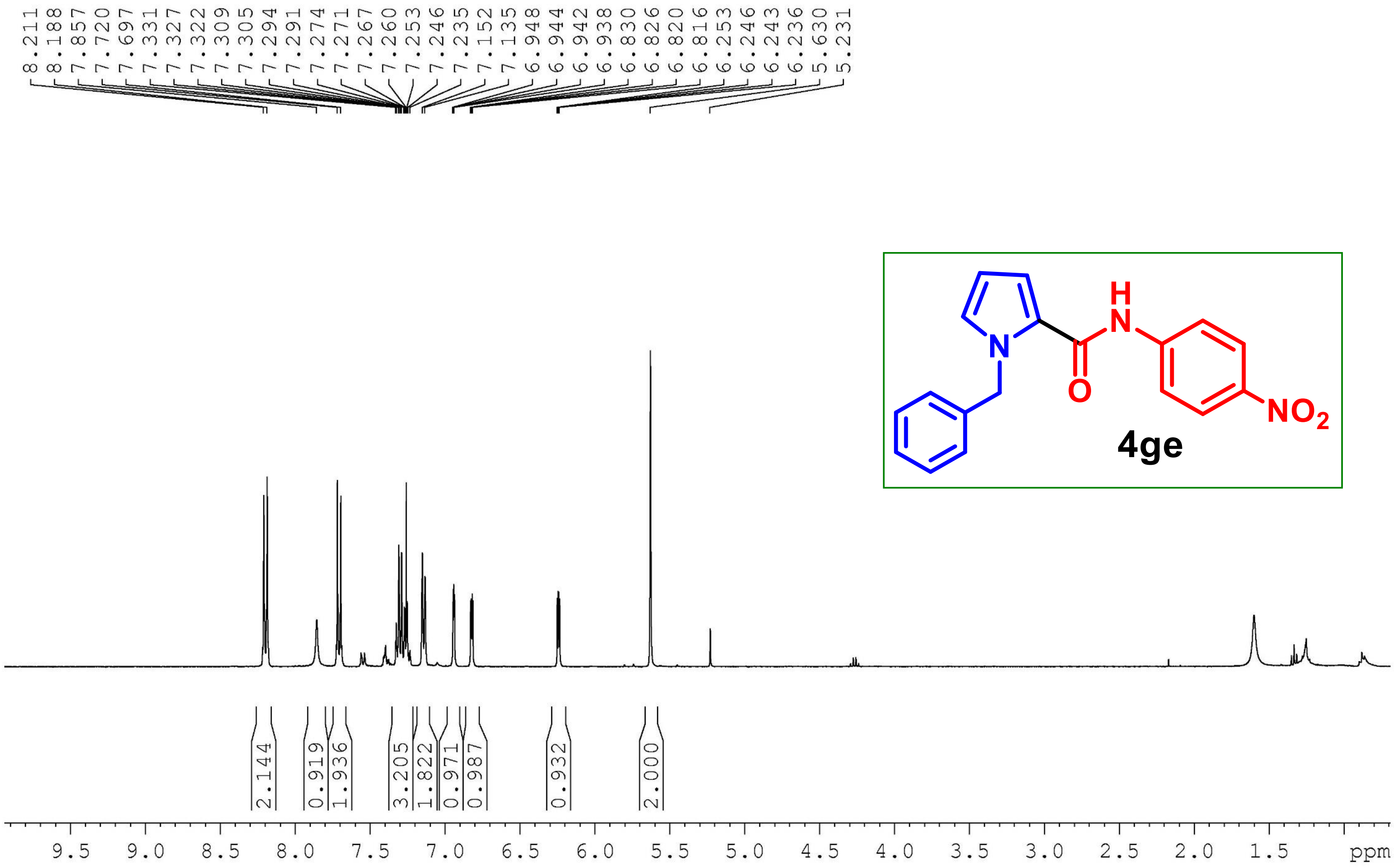

Figure S143. ^{1}H NMR spectrum (400 MHz, $CDCl_3$, 298 K) of the derivative **4ge**.

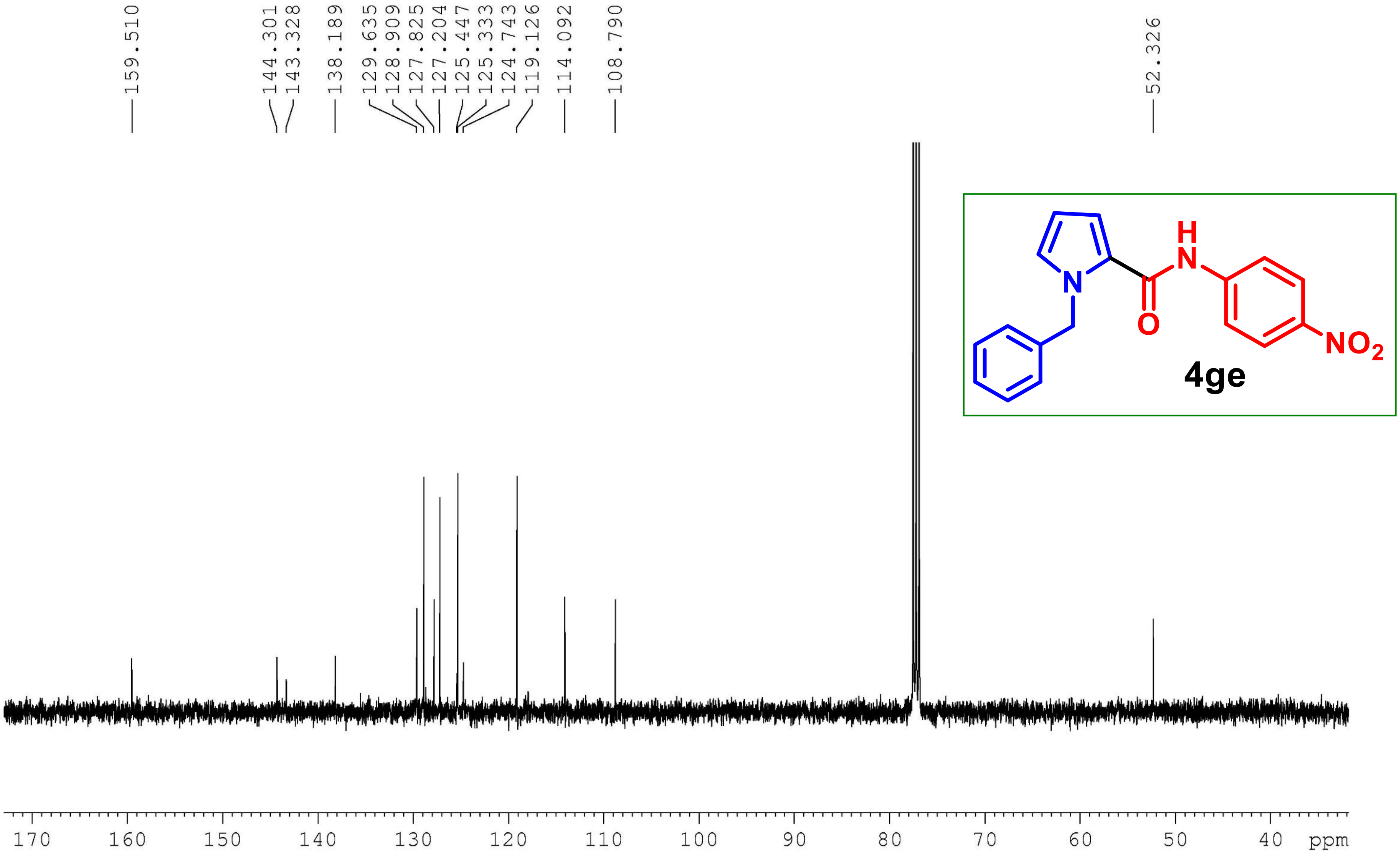

Figure S144. ^{13}C NMR spectrum (100 MHz, $CDCl_3$, 298 K) of the derivative **4ge**.

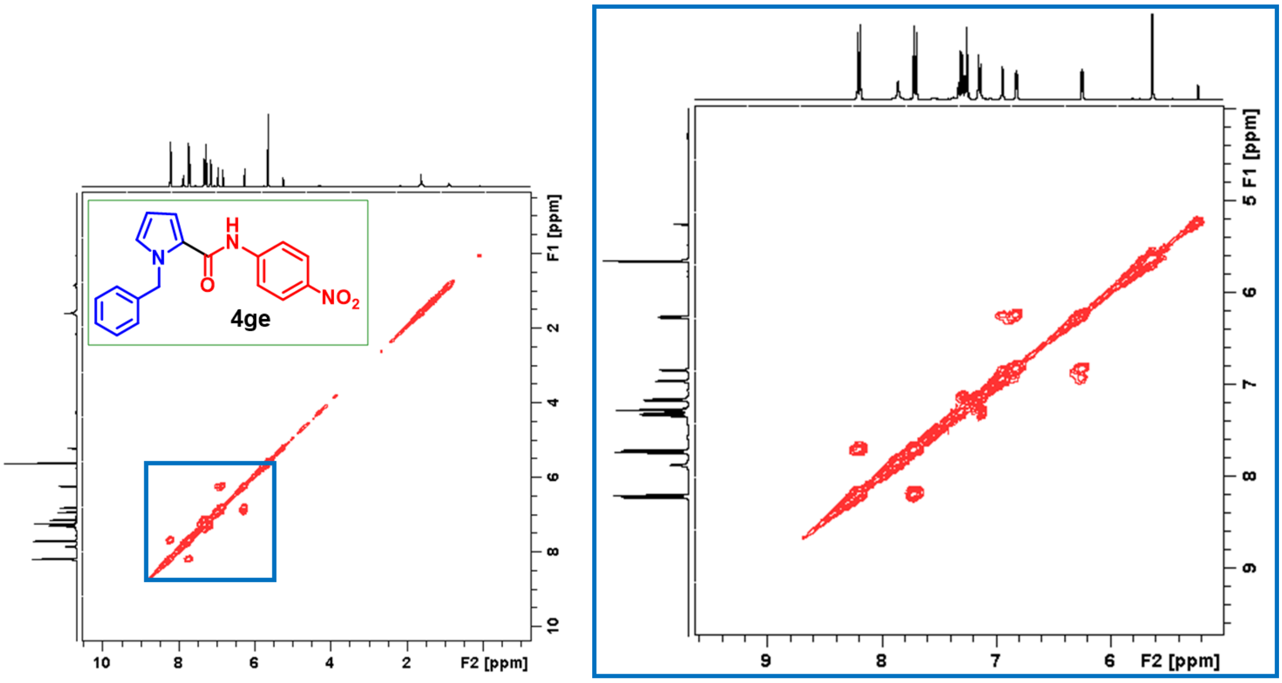

Figure S145. COSY NMR spectrum (600 MHz, $CDCl_3$, 298 K) of the derivative **4ge**, with expansion of significant portion of the spectrum in blue square.

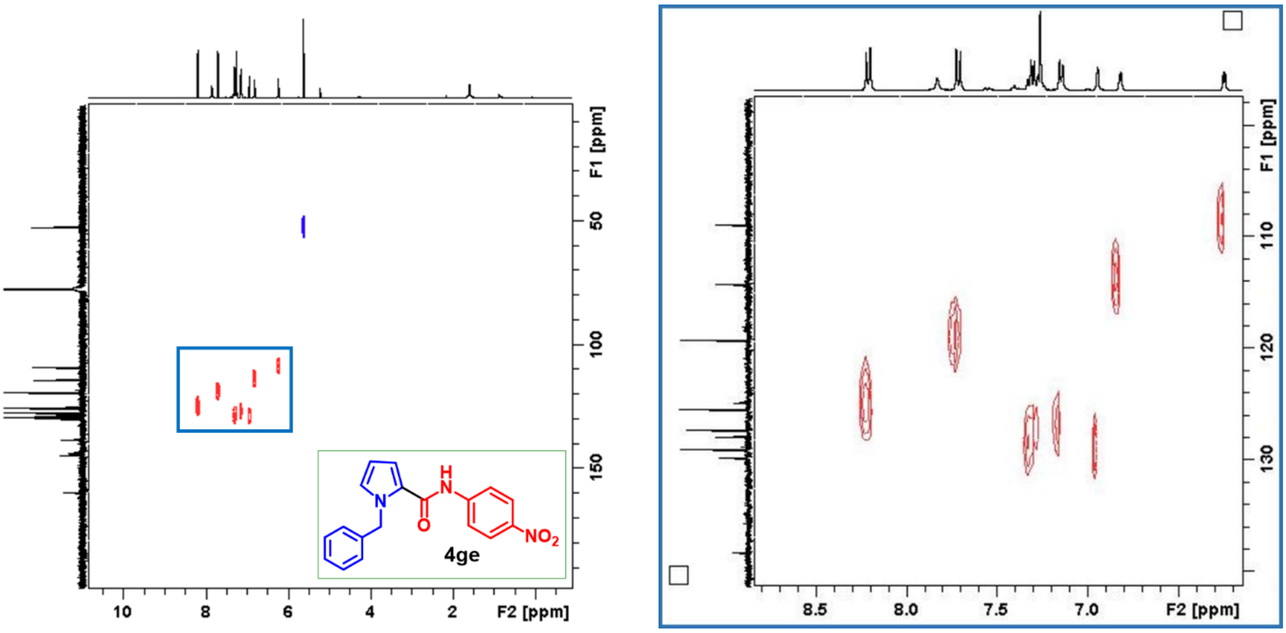

Figure S146. HSQC NMR spectrum (400 MHz, $CDCl_3$, 298 K) of the derivative **4ge**, with expansion of significant portion of the spectrum in blue square.

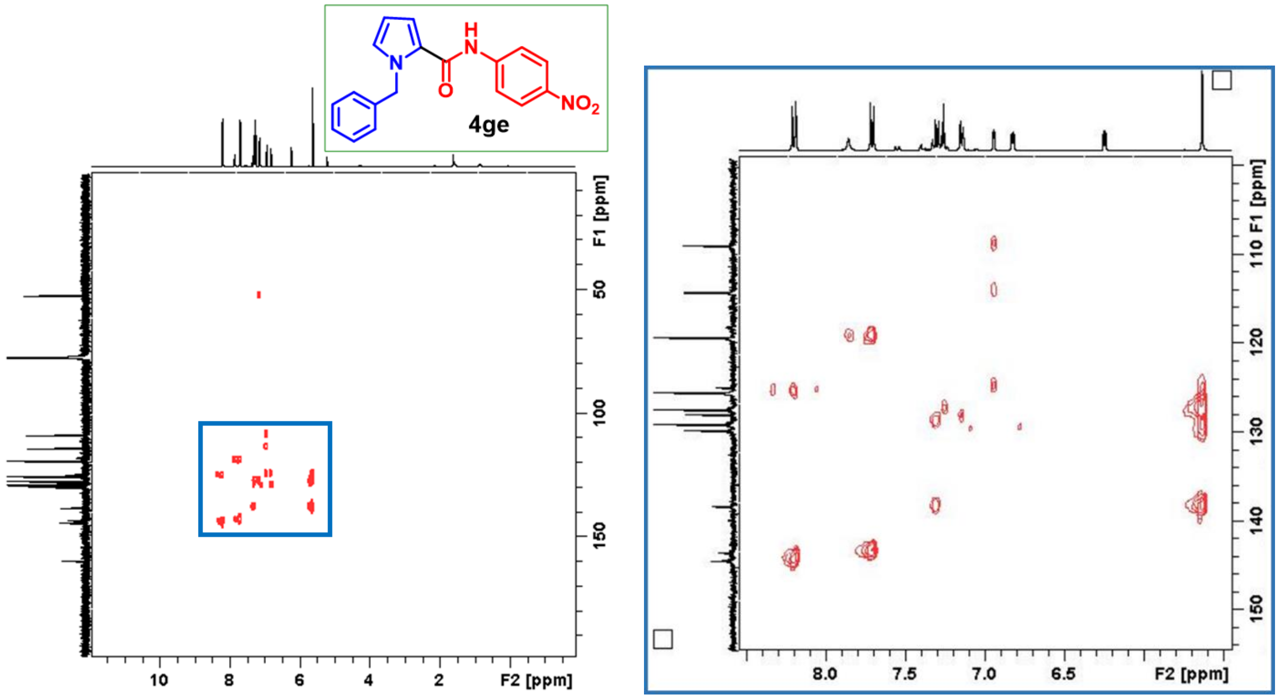

Figure S147. HMBC NMR spectrum (600 MHz, $CDCl_3$, 298 K) of the derivative **4ge**, with expansion of significant portion of the spectrum in blue square.

XII. ^{1}H and ^{13}C NMR Spectra of derivatives 4ha and fb.

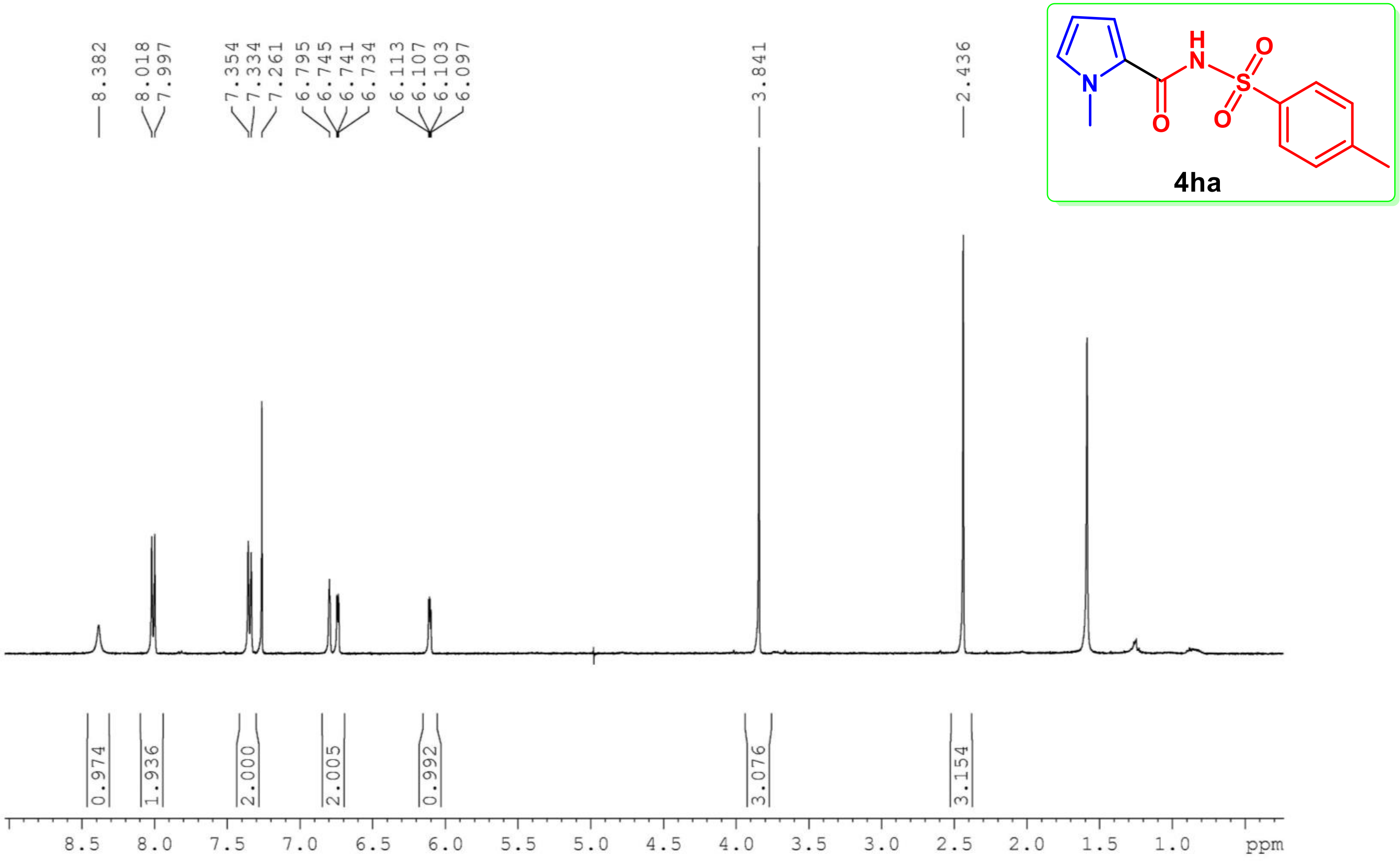

Figure S148. ^{1}H NMR spectrum (400 MHz, $CDCl_3$, 298 K) of the derivative **4ha**.

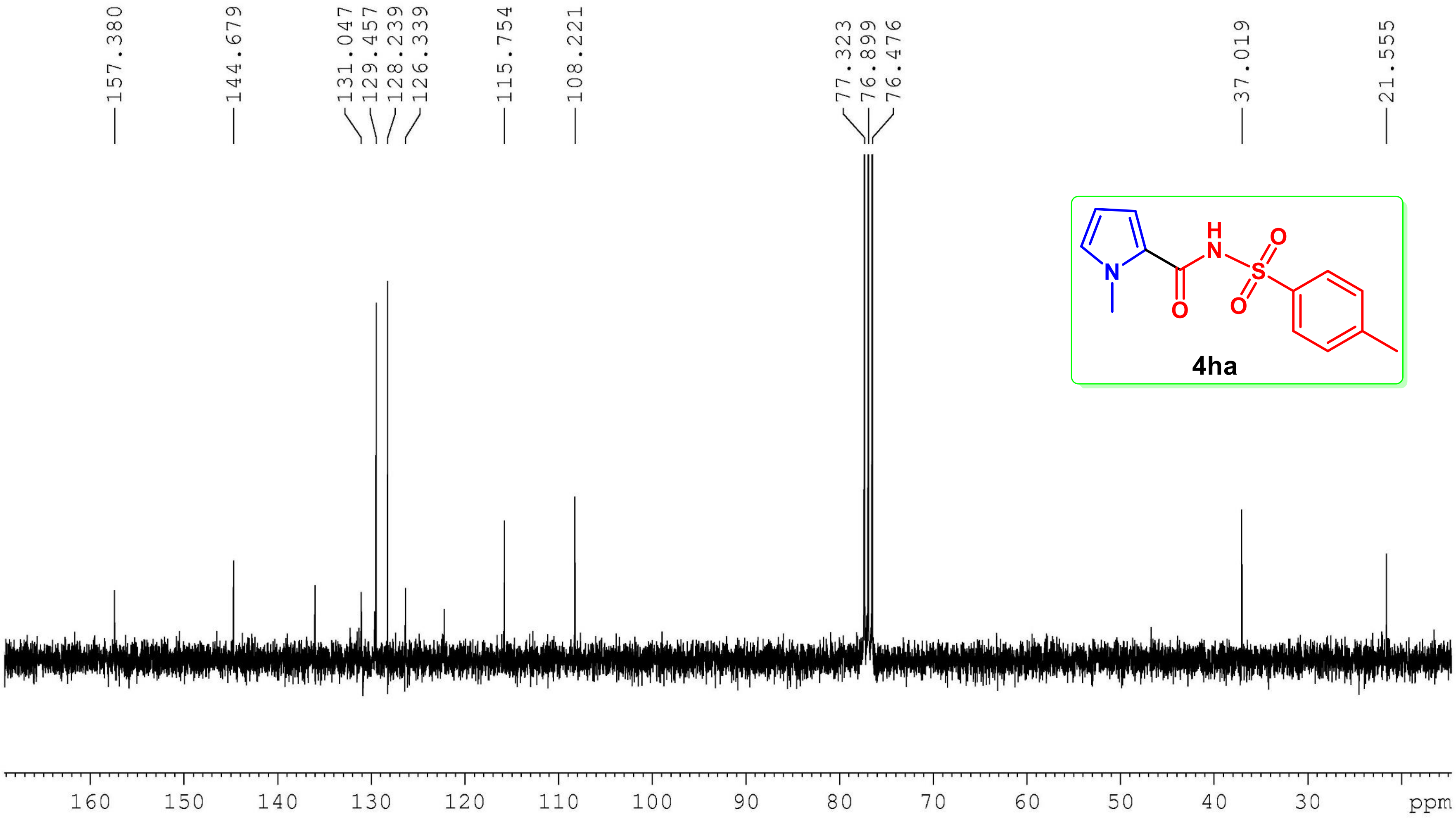

Figure S149. ^{13}C NMR spectrum (75 MHz, $CDCl_3$, 298 K) of the derivative **4ha**

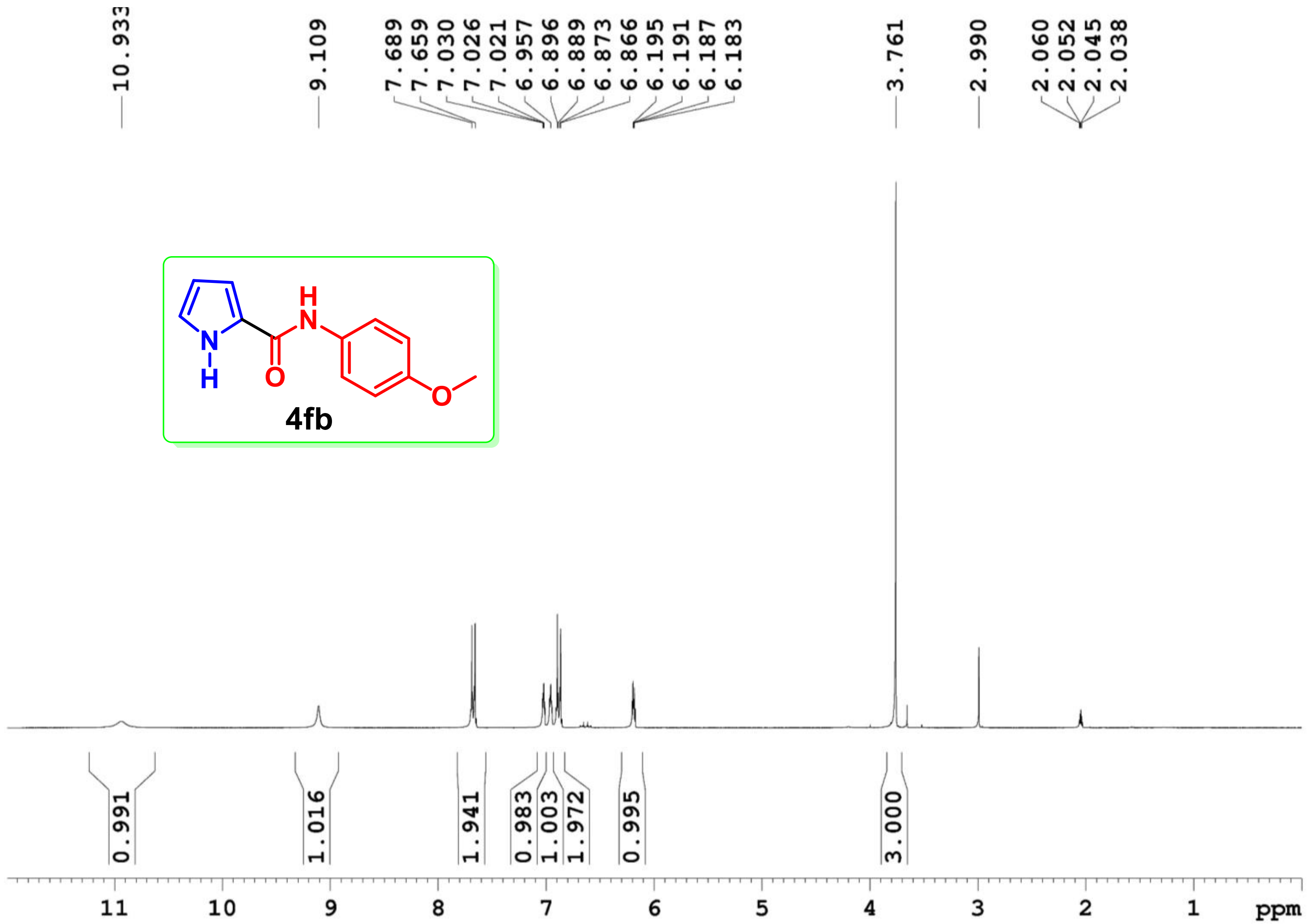

Figure S150. ^{1}H NMR spectrum (300 MHz, acetone-d_6, 298 K) of the derivative **4fb**.

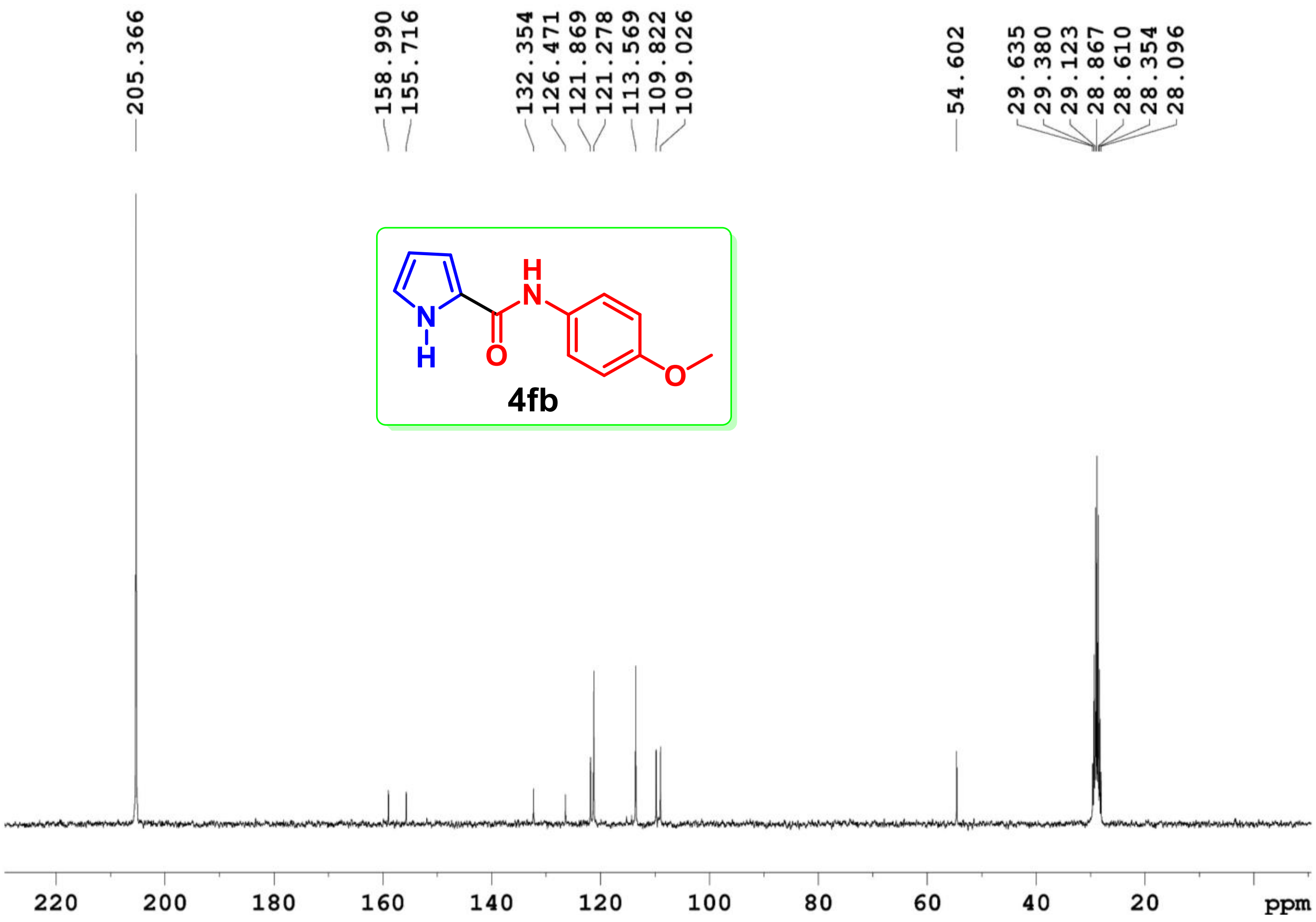

Figure S151. ^{13}C NMR spectrum (75 MHz, acetone-d_6 , 298 K) of the derivative **4fb**.

XIII Hydrolysis test of the phenyl isocyanate 3a

Procedure without capsule C

In a 4 mL glass vial, 1.1 mL of H_2O-saturated $CHCl_3$ was added, followed by isocyanate **3a** (17.8 mg, 162.6 µmol,). The solution was vigorously stirred (1400 rpm) at 50 °C and checked at different times (16, 40 and 92 h).

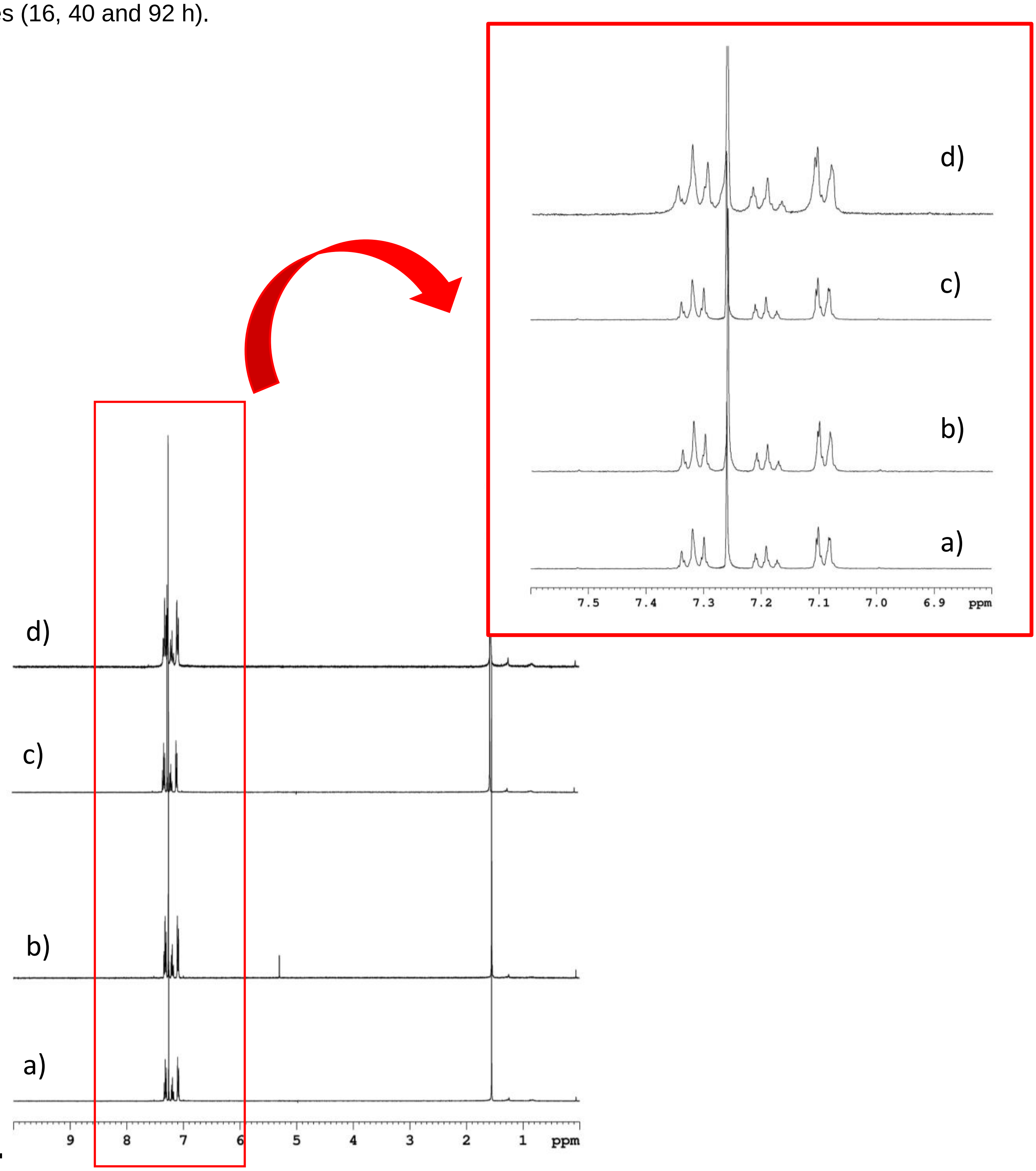

Figure S152. ^{1}H NMR spectrum (400 MHz, $CDCl_3$, 298 K) of phenyl isocyanate **3a** in chloroform-water saturated (at:50 °C) a) after mixing, b) 16 h, c) 40 h and d) 92 h.

Procedure in presence of capsule C

Resorcinarene **1** (281.6 mg, 254.7 µmol) was weighed in a 4 mL glass vial. Then, 1.1 mL of H_2O-saturated $CHCl_3$ was added and the mixture was stirred at 50 °C for 10 min. To this clear, slight yellow solution, isocyanate **3a** (17.8 mg, 162.6 µmol,) was added and the reaction mixture was vigorously stirred (1400 rpm) at 50 °C and checked at different times (16, 40 and 92h).

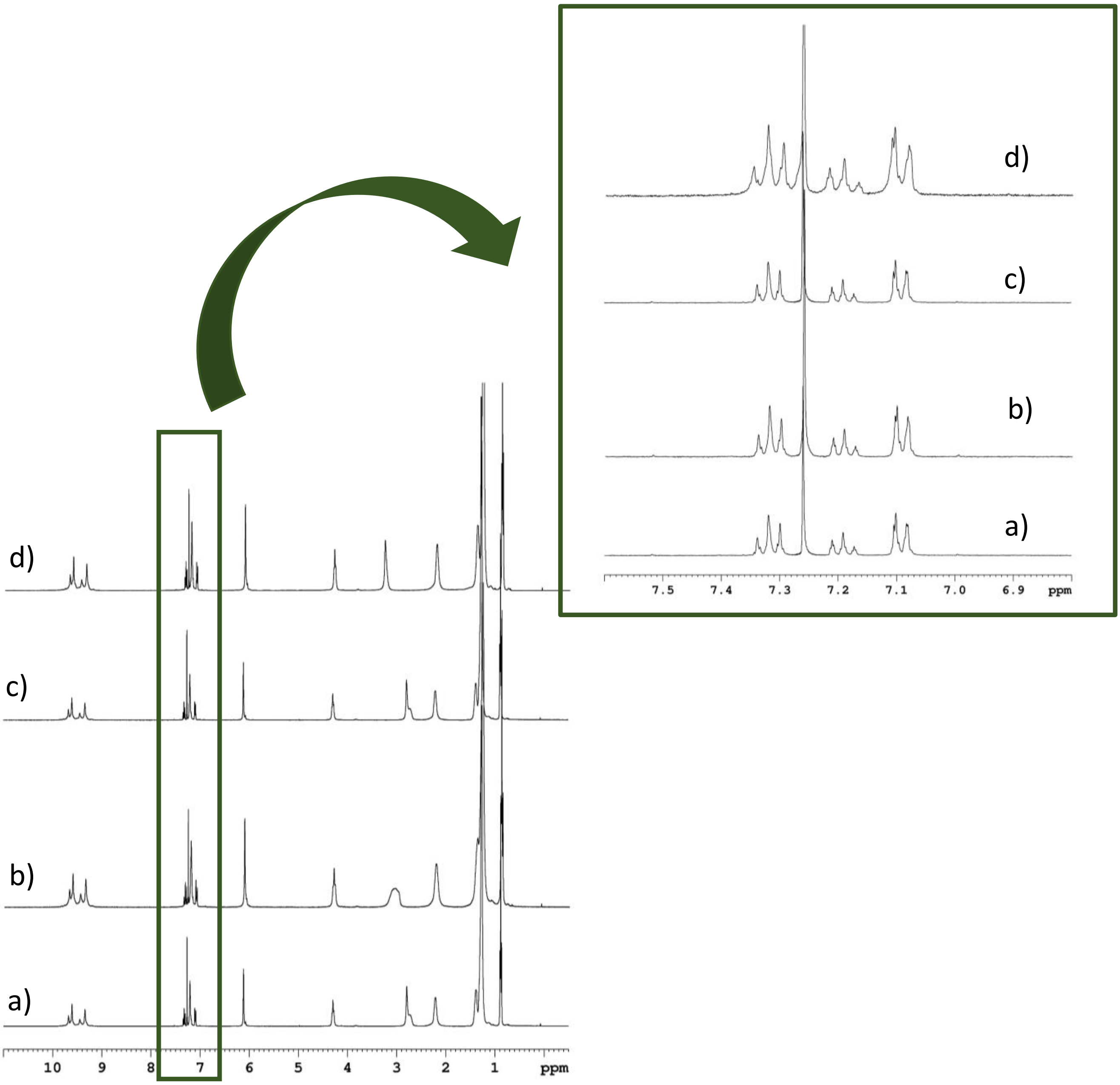

Figure S153. ^{1}H NMR spectrum (400 MHz, $CDCl_3$, 298 K) of phenyl isocyanate **3a** in chloroform-water saturated (at:50 °C) a) after mixing, b) 16 h, c) 40 h and d) 92 h.

XIV. 1 mmol Scale synthesis of 4aa in presence of the hexameric resorcinarene capsule C starting by pyrrole 2a and isocyanate 3a

Resorcinarene **1** (1.72 g, 1.56 mmol, 1.56 equiv) was weighed in a 25mL round bottom flask. Then, 6.6 mL of H_2O-saturated $CHCl_3$ was added and the mixture was stirred at 50 °C for 10 min. To this clear, slight yellow solution, the pyrrole **2a** (4.0 mmol, 4.0 eq.) was added and the solution was stirred at 30 °C for 5 min. Then, isocyanate **3a** (1.0 mmol, 1.0 eq.) was added and the reaction mixture was vigorously stirred (1400 rpm) at 50 °C for 40h. The reaction was stopped pouring the solution into 50 mL 6 Eppendorf conical tube (1.1 mL of solution for tube) and diluting each portion with a 0.13% (v/v) solution of DMSO in *n*-hexane (35 mL). The tubes were placed in a freezer at −20 °C for 3 h and successively centrifugated at 1250 rpm for 8 minutes. The diluted reaction mixture was subjected three times to this process of centrifugation/dilution with *n*-hexane. Finally, the clear solution was removed and concentrated under reduced pressure. The brown oil residue thus obtained was purified by flash chromatography on silica gel using a Hex/AcOEt gradient (from 100:0 to 90:10) to afford the derivative **4aa** (144.8 mg, 0.72 mmol, yield: 72%) as a white solid.

XV. Encapsulation Experiments: NOESY and DOSY Spectra of the Mixture of Pyrrole **2a**, Phenyl isocyanate **3a** and Capsule **C**

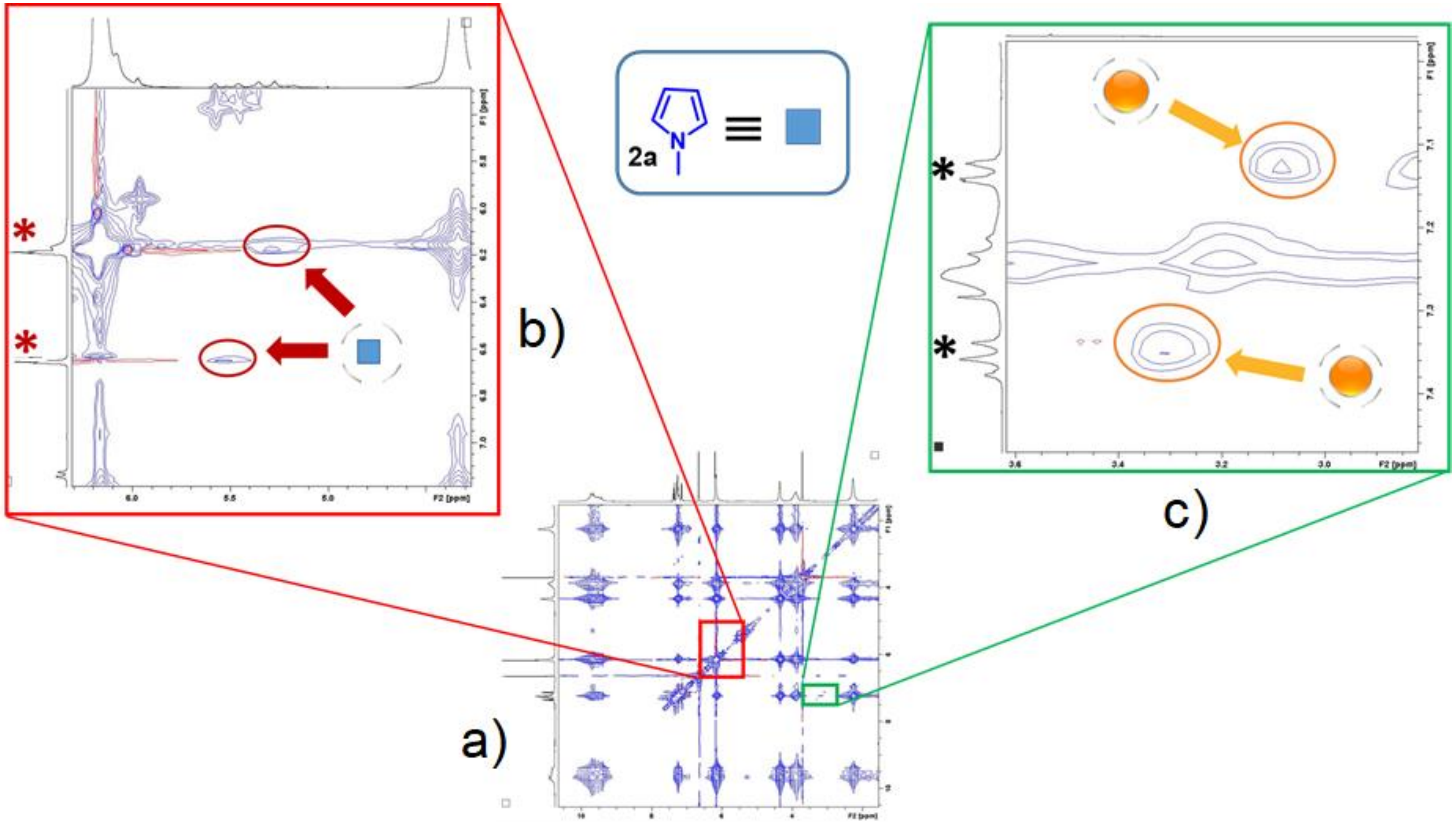

Figure S154. a) EXSY experiment (600 MHz, $CDCl_3$, 298 K, d8 = 700 ms), of the mixture of, capsule **C**, phenyl isocyanate **3a** and pyrrole **2a.** b) Significant portion of the EXSY spectrum in (a), in which exchange peaks were marked between aromatic H-atoms of **2a** outside the capsule (indicated with a brown asterisk) and inside the capsule (indicated with brown ellipses). c) Significant portion of the EXSY spectrum in (a) in which

exchange peaks were marked between aromatic H-atoms of **3a** outside the capsule (indicated with a red asterisk) and inside the capsule (indicated with an orange ellipse).

XVI. References

(**1**). Tunstad, L. M.; Tucker, J. A.; Dalcanale, E.; Weiser, J.; Bryant, J. A.; Sherman, J. C.; Helgeson, R. C.; Knobler, C. B.; Cram, D. J., Host-guest complexation. 48. Octol building blocks for cavitands and carcerands. *J. Org. Chem*. **1989**, *54*, 1305-1312.

(**2**) Hebié, S.; Devillers, C. H.; Fournier, S.; Lucas, D., Direct grafting of free base meso-triarylporphyrins on GC, ITO and Pt via 5-diazonium-triarylporphyrin electroreduction. Reversible zinc(II)-metallation of the resulting materials. *ChemElectroChem* **2016**, *3*, 45-50.

(**3**) Pingen, D.; Müller, C.; Vogt, D., Direct Amination of Secondary Alcohols Using Ammonia. *Angew. Chem. Int. Ed*. **2010**, *49*, 8130-8133.

(**4**) Nagase, Y.; Sugiyama, T.; Nomiyama, S.; Yonekura, K.; Tsuchimoto, T., Zinc-Catalyzed Direct Cyanation of Indoles and Pyrroles: Nitromethane as a Source of a Cyano Group. *Adv. Synth. Catal*. **2014**, *356*, 347-352.

(**5**) Scaggs, W. R.; Scaggs, T. D.; Snaddon, T. N., An enantioselective synthesis of α-alkylated pyrroles via cooperative isothiourea/palladium catalysis. *Org. Biomol. Chem*. **2019**, *17*, 1787-1790.

(**6**) Hong, X.; Tan, Q.; Liu, B.; Xu, B., Isocyanide-Induced Activation of Copper Sulfate: Direct Access to Functionalized Heteroarene Sulfonic Esters. *Angew. Chem. Int. Ed*. **2017**, *56*, 3961-3965.

(**7**) Singh, K.; Kabadwal, L. M.; Bera, S.; Alanthadka, A.; Banerjee, D., Nickel-Catalyzed Synthesis of N-Substituted Pyrroles Using Diols with Aryl- and Alkylamines. *J. Org. Chem*. **2018**, *83*, 15406-15414.

(**8**) La Regina, G.; Bai, R.; Coluccia, A.; Famiglini, V.; Pelliccia, S.; Passacantilli, S.; Mazzoccoli, C.; Ruggieri, V.; Sisinni, L.; Bolognesi, A.; Rensen, W. M.; Miele, A.; Nalli, M.; Alfonsi, R.; Di Marcotullio, L.; Gulino, A.; Brancale, A.; Novellino, E.; Dondio, G.; Vultaggio, S.; Varasi, M.; Mercurio, C.; Hamel, E.; Lavia, P.; Silvestri, R. New Pyrrole Derivatives with Potent Tubulin Polymerization Inhibiting Activity As Anticancer Agents Including Hedgehog-Dependent Cancer. *J. Med. Chem*. **2014**, *57*, 6531-6552.

(**9**) Herr, P.; Glaser, F.; Büldt, A. L.; Larsen, C. B.; Wenger, O. S., Long-Lived, Strongly Emissive, and Highly Reducing Excited States in Mo(0) Complexes with Chelating Isocyanides *J. Am. Chem. Soc*. **2019**, *141*, 14394-14402.

(**10**) Lee, C. K.; Jun, J. H.; Yu, J. S., Synthesis and nuclear magnetic resonance spectroscopic studies of 1-arylpyrroles. *J. Heterocycl. Chem*. **2000**, *37*, 15-24.

(**11**) Deb, I.; Coiro, D. J.; Seidel, D., Decarboxylative formation of *N*-alkyl pyrroles from 4-hydroxyproline. *Chem. Commun*. **2011**, *47*, 6473-6475.

(**12**) Borghs, J. C.; Lebedev, Y.; Rueping, M.; El-Sepelgy, O., Sustainable Manganese-Catalyzed Solvent-Free Synthesis of Pyrroles from 1,4-Diols and Primary Amines. *Org. Lett*. **2019**, *21*, 70-74.

(**13**) Tojo, Y.; Urushibara, K.; Yamamoto, S.; Mori, H.; Masu, H.; Kudo, M.; Hirano, T.; Azumaya, I.; Kagechika, H.; Tanatani, A., Conformational Properties of Aromatic Oligoamides Bearing Pyrrole Rings. *J. Org. Chem*. **2018**, *83*, 4606-4617.

(**14**) G. La Regina, R. Silvestri, M. Artico, A. Lavecchia, E. Novellino, O. Befani, P. Turini, E. Agostinelli, *J. Med. Chem*. **2007**, *50*, 922-931.

(**15**) Yuan, S. –W.; Han, H.; Li, Y. –L.; Wu, X.; Bao, X.; Gu, Z. –Y.; Xia, J. –B., Intermolecular C−H Amidation of (Hetero)arenes to Produce Amides through Rhodium-Catalyzed Carbonylation of Nitrene Intermediates. *Angew. Chem. Int. Ed*. **2019**, *58*, 8887-8892.

(**16**) Vaillard, V. A.; Guastavino, J. F.; Budén, M. E.; Bardagí, J. M.; Barolo, S. M.; Rossi, R. A., Synthesis of 6-Substituted 2-Pyrrolyl and Indolyl Benzoxazoles by Intramolecular O-Arylation in Photostimulated Reactions. *J. Org. Chem*. **2012**, *77*, 1507-1519.

(**17**) La Sorella, G.; Sperni, L.; Strukul, G.; Scarso, A., Supramolecular Activation of Hydrogen Peroxide in the Selective Sulfoxidation of Thioethers by a Self-Assembled Hexameric Capsule. *Adv. Synth. Catal*. **2016**, *358*, 3443-3449.
